# Abstracts of the 20th European Conference on Eye Movements, 18-22 August 2019, in Alicante (Spain) 

**DOI:** 10.16910/jemr.12.7.1

**Published:** 2019-11-25

**Authors:** Susana Martinez-Conde, Luis Martinez-Otero, Albert Compte, Rudolf Groner

**Affiliations:** SUNY Downstate Health Sciences University, New York, USA; Universidad Miguel Hernández, Alicante; Institut d'Investigacions Biomèdiques August Pi i Sunyer (IDIBAPS); University of Bern and Journal of Eye Movement Research

**Keywords:** eye movements, perception, attention, reading, usability, microsaccades

## Abstract

This document contains all abstracts of the 20th European Conference on Eye Movements, August 18-22, 2019, in Alicante, Spain

**Video stream "Glimpses at 20th ECEM":** https://vimeo.com/user43478756

## Abstract Book, www.ecem2019.com



