# Supplementary material for: Abstracts of the 20th European Conference on Eye Movements, 18-22 August 2019, in Alicante (Spain)
Source: J Eye Mov Res. 2019 Nov 25;12(7):10.16910/jemr.12.7.1. doi: 10.16910/jemr.12.7.1 (PMC7917478; doi:10.16910/jemr.12.7.1)
Supplement: Supplementary file 1 [file jemr-12-07-a-SD1-01.pdf]

20th ECEM 2019

European Conference on Eye Movements

18-22 August 2019, Alicante, Spain

Abstract Book

www.ecem2019.com

Symposiums
20th European Conference on Eye Movements

Symposium 1
Sunday, August 18th 16:15 – 18:15 Plenary room

Word processing across space and time during reading: Insights from eye tracking, electrophysiology, and computational modeling

Organizers: Elizabeth R. Schotter & Brennan Payne

Are our insights into the fundamental cognitive processes of reading limited by our methodologies? As disparate fields converge and researchers combine approaches to study the same cognitive processes, open dialogue must guide us forward, bring us in closer alignment, and generate new fruitful collaborations. This symposium aims to convene researchers who study parafoveal processing in reading using (1) gaze-contingent eye tracking paradigms, (2) ERP and fMRI paradigms using RSVP with parafoveal flankers, (3) EEG and eye-tracking co-registration studies, and (4) computational models. Each talk will address one of the 'big questions' that will be the focus of the panel discussion (i.e., questions about the time-course of word recognition, the sub-processes involved, and the relevant linguistic variables), summarize how their research supports their perspective, and discuss open questions that would benefit from integration with other methods. The panel discussion, led by Elizabeth Schotter and Brennan Payne, will spark lively debate based on the presentations and include questions from the audience. Although the focus is on reading processes, the implications of combining methods and approaching questions from different perspectives is of interest to a broad range of ECEM attendees interested in visual attention.

Efficient reading requires constant processing of upcoming words simultaneously with direct fixation on preceding words (i.e., *parafoveal preview*; Schotter, 2018). Eye movement research on this topic raises questions about whether high-level linguistic processing of the currently fixated and subsequent words coincide, or occur in a sequential process (Talk 1: Veldre et al.). By focusing primarily on eye movement measures, which only tap the behavioral consequences of underlying neural processing rather than the timings of the neural processes directly, our inferences about underlying neuro-cognitive processes are limited. However, most electrophysiology studies on the neural processes of reading have used unnatural paradigms (i.e., Rapid Serial Visual Presentation; RSVP) that presented words sequentially at a fixed rate and outside of the readers control, barring the ability to address questions about parafoveal processing. Recent RSVP paradigms that present parafoveal words with foveal words provide new ways address the neural signatures of parafoveal processing (Talk 2: Barber et al.), but still eliminate eye movements, and consequently the ability to directly relate neural processing to overt behavior. The recent development of co-registration studies offers the opportunity to investigate neural reading processes time locked to naturally generated eye movements (Talk 3: Kretzschmar et al.), and how processing of one word changes across fixations (Talk 4: Dimigen & Tischer), but this literature is in its infancy, and there are currently a number of open debates surrounding best practices in the design, measurement, and interpretation of data from these studies. Ultimately, any inferences we make from this literature must be combined into a broad theory of online reading processes, which specifies the relationship between neural processes on a word and the eye movements that bring it from parafoveal vision, into foveal vision, and then move on (Talk 5: Reichle & Schotter). The big questions that will motivate these talks are:

1. Is word recognition best conceptualized as a singular 'magic moment' or a temporally extended process? In what ways do the focuses of different methodologies (e.g., ballistic eye-movements versus continuous EEG) lead to progress in theory development in our understanding of word processing during reading?
2. Does word recognition vary qualitatively across the visual field? Can the same processes occur for a word in the parafovea as in the fovea? Is it possible to deconfound questions about space from questions about the time course of processing multiple words in natural reading?
3. What are the biggest logistical challenges to integrating these methodologies as the field moves forward to integrate largely 'siloed' literatures? In what ways do historical conventions across fields (e.g., favoring lexical frequency in eye tracking studies versus favoring contextual fit and violation paradigms in EEG) influence the nature of the cognitive and linguistic processes we focus on in our research?

1: Aaron Veldre, Roslyn Wong, & Sally Andrews

Plausibility preview effects reveal contextual influences on word identification during online reading

Abstract: Readers extract information from foveal and parafoveal words in order to plan when and where to move their eyes next. Much of the evidence for parafoveal processing comes from studies using the boundary paradigm

in which the preview of a target word is manipulated prior to direct fixation by the reader. This talk reviews recent demonstrations of plausibility preview effects: Skipping and first-pass fixation duration benefits for contextually plausible preview words relative to implausible words. These effects have been shown to be independent of the preview's orthographic or semantic similarity to the target and occur for targets that are low in cloze predictability. These data provide strong evidence that readers extract semantic and syntactic information from parafoveal words. However, the benefit of parafoveal semantic information depends on the fit between the preview and the sentence, rather than the semantic overlap between the preview and target word, implying an early and direct influence on oculomotor planning. Taken together, the findings imply that readers rapidly and incrementally integrate words with the developing representation of the sentence to generate graded predictions about plausible continuations, even in unconstraining contexts. The implications for models of eye movement control are discussed.

2: Horacio A. Barber, Francisco Rivero, Julien Dampuré, Laura Hernández-Ramos, & Enrique García-Marco

Parafoveal word processing: What can we learn from "unnatural reading paradigms"?
Abstract: The electrophysiological correlates of parafoveal word processing have been studied with a modification of the RSVP paradigm (Rapid Serial Visual Presentation; which is standard in EEG and neuroimaging research of reading). In the RSVP-with-flankers paradigm, sentences are presented word by word at fixation, but additionally each word is flanked two degrees bilaterally by other words; on its right by the next word in the sentence and on its left by the preceding word. In this talk I will review previous studies using this paradigm, and describe the results of two experiments (EEG and fMRI) in which we directly compared brain activity associated to sentence reading either presented with the standard RSVP or the RSVP-with-flankers paradigm. ERPs associated to reading target nouns embedded in sentences showed larger N400 amplitudes when being read with the RSVP than with the RSVP-with-flankers paradigm. Consistently, metabolic measures showed an increase of the BOLD signal in the RSVP condition in respect to the RSVP-with-flankers conditions at posterior parietal and occipital areas of the cortex. The activation in these areas increases when no flankers are presented in the RVSP, probably showing the inhibition of a default visual-attention mode during reading that involves parafoveal processing.

3: Franziska Kretzschmar, Erika Mayer, & Adrian Staub

The time-course and source of lexical predictability effects in reading
Abstract: Effects of lexical predictability emerge in both eye movement and ERP data. However, their timing is difficult to reconcile: Effects on word skipping and first fixation duration are earlier than effects on N400 amplitude. One explanation is that the two measures reflect qualitatively different predictability effects: semantic processing in the N400 (Kutas & Federmeier, 2011), and orthographic processing in eye movements (Staub & Goddard, 2019). This division has implications for the interaction between parafoveal processing and predictability. Because orthographic processing begins in parafoveal vision, eye movements should be highly sensitive to the predictability of the parafoveal word. The parafoveal N400, by contrast, should index preview predictability only when the preview undergoes semantic processing, and the foveal N400 should be insensitive to invalid preview. We tested these assumptions in a series of eyetracking/EEG co-registration experiments using the boundary paradigm and manipulating preview predictability and validity. Initial results show that foveal predictability effects in the eye movement record are diminished after invalid preview, while the foveal N400 amplitude is unaffected. We will discuss our findings in terms of the interaction of orthographic bottom-up information with predictions, as well as implications for our understanding of predictability effects on eye movements and ERPs.

4: Olaf Dimigen & Sophia Tischer

Understanding the brain-electric correlates of the saccadic preview benefit
Abstract: Words (and other objects) can often be partially processed in extrafoveal vision, leading to shorter fixations once they are foveated. Although combined eye movement/EEG recordings provide a promising approach to understand how and when information is integrated across saccades, relatively little is still known about the neural correlates of this preview benefit. This talk aims to provide an overview over current findings on EEG preview benefits and new techniques for their measurement (linear deconvolution models, Ehinger & Dimigen, 2018). I will summarize key findings from co-registration experiments on the preview benefit that show that the preview positivity – a reduction of the late N1 component after valid previews –

is a highly reliable phenomenon that can be measured regardless of stimulation paradigm (flanker-RSVP vs. natural reading), writing system (alphabetic vs. symbolic), reading materials (word lists vs. sentences), display change awareness, and stimulus category (words vs. faces). However, I will also highlight some differences between these conditions. I will also describe two experiments using alternating-case and "pixel cloud" previews to investigate the relative contribution of genuine preview benefits (i.e. facilitation), incorrect-preview costs, and display change costs to the effects seen in EEG. Results will be used to discuss the brain-behavior relationship.

5: Erik D. Reichle & Elizabeth R. Schotter

Using simulations to understand parafoveal preview and the nature of lexical processing during reading
Abstract: Two prominent models of eye-movement control in reading, E-Z Reader (Reichle, Pollatsek, & Rayner, 2012) and SWIFT (Schad & Engbert, 2012), posit discrete stages of lexical processing. Is this assumption just a modelling convenience, or does it reflect something meaningful about the nature of lexical processing during reading? If the latter is true, what is nature of the distinction between the early and late stages: (i) form (e.g., orthographic) vs. deeper (e.g., semantic) processing (Reingold & Rayner, 2006); (ii) lexical familiarity vs. recall of lexical information (Reichle & Perfetti, 2003); (iii) a threshold adopted in the service of increasing saccadic programming efficiency vs. lexical identification (Reichle & Laurent, 2006); (iv) or the rise vs. fall in lexical activation (Schad & Engbert, 2012)? A further question is whether both stages can be performed based on parafoveal information, or whether foveal information is necessary. We used E-Z Reader to examine the time course of lexical processing in the fovea and parafovea by simulating data from eye tracking studies using the boundary paradigm (Rayner, 1975). The results of these simulations support a theoretical distinction between an early and late stages of lexical processing and provide new insights into the nature of that distinction.

References:

Ehinger & Dimigen (2019). *PeerJ.*
Kutas, & Federmeier (2011). *Annual Review of Psychology, 62.*
Rayner (1975). *Cognitive Psychology, 7.*
Reichle, & Laurent (2006). *Psychological Review, 113.*
Reichle, & Perfetti (2003). *Scientific Studies of Reading, 7.*
Reichle, Pollatsek, & Rayner, (2012). *Psychological Review, 119.*
Reingold, & Rayner (2006) *Psychological Science, 17.*
Schad, & Engbert (2012). *Visual Cognition, 20.*
Schotter (2018). *Psychology of Learning and Motivation, 68.*
Staub, & Goddard (2019). *Journal of Experimental Psychology: Learning, Memory, and Cognition, 45.*

Authors, Affiliations, & Contact for First Authors

Elizabeth R. Schotter, University of South Florida, *eschotter@usf.edu*
Brennan Payne, University of Utah, *brennan.payne@utah.edu*
Aaron Veldre, University of Sydney, *aaron.veldre@sydney.edu.au*
Roslyn Wong, University of Sydney
Sally Andrews, University of Sydney
Horacio A. Barber, Universidad de La Laguna, *hbarber@ull.edu.es*
Francisco Rivero, Universidad de La Laguna
Julien Dampuré, Universidad de La Laguna
Laura Hernández-Ramos, Universidad de La Laguna
Enrique García-Marco
Franziska Kretzschmar, University of Cologne, *fkretzs1@uni-koeln.de*
Erika Mayer, University of Massachusetts, Amherst
Adrian Staub, University of Massachusetts, Amherst
Olaf Dimigen, Humboldt Universität zu Berlin, *olaf.dimigen@hu-berlin.de*
Sophia Tischer, Humboldt Universität zu Berlin
Erik D. Reichle, Macquarie University, *erik.reichle@mq.edu.au*

Symposium 2
Monday, August 19th 10:30 – 12:30 Room 4

What eyes tell us about child sensorimotor and cognitive development

D.P. Munoz[1], B. Luna[2], A. Hillairet de Boisferon[3], L.M. Schmitt[4], J.L. Kleberg[5]
Organizer: N. Alahyane[3]
1 Centre for Neuroscience Studies, Queen's University, Kingston, ON, Canada
2 University of Pittsburgh Departments of Psychiatry and Psychology
3 Paris Descartes University, Institut de Psychologie, Boulogne-Billancourt, France
4 Division of Developmental and Behavioral Pediatrics, Cincinnati Children's Hospital Medical Center, Cincinnati OH, USA
5 Department of Psychology, Uppsala University / Department of Clinical Neuroscience, Karolinska Institutet, Sweden

From birth, we move our eyes to explore the environment and interact with it. Given that various stimuli surround us, we must select and generate the most appropriate saccadic eye movement among the multiple competing response options, relying upon the environment cues and internal goals. The saccade system allows thus to study not only basic sensorimotor processes but also higher-order processes such as attention, memory, response inhibition, response planning and decision-making, supporting cognitive control and hence voluntary control over behavior. Simple tasks such as prosaccade (look at a peripheral stimulus) and antisaccade (look away from the stimulus) tasks are extensively used to examine these sensorimotor and cognitive controls of behavior. Dynamics of pupil size is another increasing used index of cognitive function but also arousal, this latter influencing behavior and performance. The large-scale oculomotor brain network undergoes dynamic and specific anatomical changes during child development and overlaps with the brain circuits that are impaired in various neurodevelopmental disorders. In sum, eyes represent a unique window into the emergence, development and specialization of the sensorimotor and cognitive processes in the developing brain at both behavioral and neural levels. Eye movements and pupil responses offer a rich repertoire of quantitative measures sensitive to specific developmental changes of these processes. Eyes give thus a unique translational target for investigating behavioral deficits and their neural substrates across development, leading to providing endophenotypes of brain disorders. In turn, studies in populations with neurodevelopmental disorders further our understanding of critical stages of control of behavior and brain maturation.
The aim of the symposium is to present novel research on oculomotor control that employs complementary eye tracking, pupillometry and neuroimaging methods as well as basic and clinical approaches to explore sensorimotor and cognitive controls across child development. The first three talks will present normative development while the last two will discuss saccade control when it is compromised in two neurodevelopmental disorders: autism spectrum disorder and attention deficit/hyperactivity disorder.
Douglas Munoz will show saccade, pupil and eyeblink response data in a large cohort of participants from 5 to 25 years of age, looking at typical development of sensorimotor, cognitive and autonomic functions. **Beatriz Luna** will describe multimodal neuroimaging evidence of changes in brain function and connectivity underlying neurocognitive maturation using saccade tasks, in 8-30 years-olds. **Anne Hillairet de Boisferon** will focus on early development of saccade control. She will present an original experimental protocol testing sensorimotor and cognitive control of internally-triggered saccades in preverbal and preschool children. **Lauren Schmitt** will discuss saccade abnormalities in individuals with autism spectrum disorder (ASD) and how they are familial and relate to potential pathophysiological processes. **Joan Lundin Kleberg** will discuss saccade impairments in children with attention deficit/hyperactivity disorder (ADHD) with attenuated arousal and show how their saccade performance can be improved with environmental cues using saccade tasks and pupillometry. A general discussion will close the symposium.

TALK #1

Saccade, pupil, and eyeblink responses change across development
Douglas P. Munoz, Donald C. Brien, Brian C. Coe, Jeff Huang
Centre for Neuroscience Studies, Queen's University, Kingston, ON, Canada

Video-based eye tracking can offer detailed insight into the development of various cognitive processes through rigorous quantitative analysis of saccade, pupil and blink responses. We have developed a short oculomotor

test battery that consists of 20 minutes of interleaved pro- and anti-saccades trials, followed by 10 minutes of free viewing of dynamic video. This test battery can be completed by participants from ages 5-95 years and is optimized to identify behavioural biomarkers of sensory, motor, cognitive, and autonomic dysfunction in various neurological and psychiatric patients. In the pro-saccade task, participants are instructed to look at a peripheral visual stimulus, while in the anti-saccade task they are instructed to look in the opposite direction. There is now extensive knowledge of the neural circuitry that underlies these behaviours and this knowledge can be used to identify key biomarkers. This presentation will focus on important developmental changes in saccades, microsaccades, pupil responses, and eyeblinks recorded from 260 healthy individuals between the ages of 5 and 25 years. These data provide important insight into developmental milestones and are useful as normative standards for data sets collected from different neurological and psychiatric patient groups.
doug.munoz@queensu.ca

TALK #2

Oculomotor evidence of cognitive maturation through adolescence
Beatriz Luna[1,2,3], Brenden Tervo-Clemmens[2,3], Finn Calabro[1,3], and Ashley Parr[1,3]
University of Pittsburgh Departments of Psychiatry[1] and Psychology[2]
Center for the Neural Basis of Cognition[3]

Adolescence is a time when neurocognitive processes are available but are undergoing significant specialization. Understanding the processes that underlie adolescent specialization is critical for informing dynamic aspects of neurocognitive development in normative and impaired populations. Here we describe findings from a normative accelerated longitudinal cohort of approximately 150 8-30 year olds who performed visually guided (VGS), antisaccade (AS), and memory-guided saccade (MGS) tasks behaviorally and subsets who also performed these tasks during fMRI. Behavioral data indicated significant decreases in latency across all tasks stabilizing through adolescence into adulthood reflecting optimization of information processing. AS and MGS performance continued to improve into adulthood in a curvilinear fashion. fMRI results indicated that prefrontal systems become integrated through childhood and are in place by adolescence. However, engagement of the dorsal anterior cingulate cortex and visual association areas become increasingly integrated into adulthood in parallel with improved performance. Importantly, intra-subject variability decreased with age in association with decreases in the variability of the magnitude of expression of whole brain patterns of cognitive function. Finally, results linking DA and development of cognitive control of eye movement will be discussed. Together, these results provide compelling evidence that adolescence is a period of active specialization of cognitive processes determining adult trajectories.
lunab@upmc.edu

TALK #3

I choose where to look: control of voluntary saccades in the first years of life
Anne Hillairet de Boisferon, Christelle Lemoine-Lardennois, Karine Doré-Mazars, Nadia Alahyane
Paris Descartes University, Institut de Psychologie, Laboratoire Vision Action Cognition, Boulogne-Billancourt, France

Early development of control of voluntary saccades (VS) remains poorly known despite their critical role in active visual perception, cognitive tasks and social interactions. While reactive saccades (RS) are easily elicited notably in preverbal children, VS are generally initiated toward complex scenes or following an instruction to look toward a preexisting stimulus, and based on an endogenous decision. We previously showed that immature RS performance in 7-42 month-old children improve with age (latency) and over the 140 trials (accuracy), suggesting differential effects of brain development and environment. We recently adapted this protocol (Alahyane et al., 2016) to elicit VS without verbal instruction in preverbal children and adults, using an overlap procedure. To prompt a decision, two identical peripheral stimuli appeared simultaneously at 10° eccentricity from a fixation point (140 trials). Results showed that toddlers were able to select and direct their gaze toward one target without any explicit task instruction, and were slower than adults. Compared to RS, children generated VS with longer latency but also higher accuracy, suggesting a benefit from additional time. Characterizing early development of the basic control of voluntary saccades provides a groundwork to better understand typical or atypical development of cognitive and socio-cognitive processes.
anneh2b@gmail.com

TALK #4

20 Years of Oculomotor Control Studies in Autism Spectrum Disorder: What We've Learned and Where We Go From Here

Lauren M. Schmitt[1] & John A. Sweeney[2]

1 *Division of Developmental and Behavioral Pediatrics, Cincinnati Children's Hospital Medical Center, Cincinnati OH, USA*

2 *Department of Psychiatry, University of Cincinnati, Cincinnati OH, USA*

Cerebellar and brainstem abnormalities have been the most consistently documented postmortem and MRI findings in autism spectrum disorder (ASD), consistent with numerous studies reporting emergence of motor abnormalities prior to onset of hallmark clinical features. Over the past two decades, our work and others have highlighted a profile of saccadic and pursuit eye movement supporting cortical-pontine-cerebellar-thalamic-cortical loop involvement in ASD. Additionally, we recently extended our previous findings of a similar profile of abnormal saccadic and pursuit eye movements in unaffected first-degree relatives of individuals with ASD, with novel evidence from our family trio study (62 individuals with ASD (probands), 135 unaffected biological parents, and 76 matched controls) showing specific eye movement abnormalities are not only present in both probands and their unaffected biological parents, but also these deficits were inter-correlated among family members. For example, both altered peak saccade velocity and lateralized predictive saccade latencies each were found to be familial. Taken together, the past two decades of research into eye movements in ASD has offered crucial insights into potential underlying pathophysiological mechanisms. Still bridging the gap between these biomarker studies and translational medicine approaches has remained limited, appealing for the critical need for future research in this area.

lauren.schmitt@cchmc.org

TALK #5

Auditory cues normalize saccadic latencies in children with ADHD

Johan Lundin Kleberg[1,2,3], Matilda A. Frick[1], Karin C. Brocki[1]

1 *Uppsala Child and Baby Lab, Department of Psychology, Uppsala University, Box 1225, 751 42 Uppsala, Sweden*

2 *Department of Clinical Neuroscience, Karolinska Institutet, 171 65 Solna, Sweden*

3 *Child and Adolescent Psychiatry, Stockholm City Council, Stockholm, Norra Stationsgatan 69, 113 64 Stockholm, Sweden*

Attenuated baseline arousal has been suggested to underlie attention deficit/hyperactivity disorder (ADHD) and comorbid externalizing disorder. Individuals with low baseline arousal are often hyperresponsive to warning signals (alerting cues). We examined the effects of auditory warning signals on saccadic latencies in children with ADHD in a combined eye tracking and pupillometry study. In the absence of auditory warning cues, children with ADHD (N = 24) were slower than controls (N = 43) to initiate a saccade to visual targets. Saccadic reaction times in the ADHD group were normalized by auditory cues (brief beeps). The auditory cues led to enhanced pupil dilation, suggesting that the effect on visual attention was modulated by increased locus coeruleus-noradrenergic arousal. In addition, children with ADHD showed increased pupil dilation response to naturalistic auditory cues. This demonstrates that, similar to other populations with attenuated arousal, children with ADHD have an enhanced facilitating effect of auditory warning cues. Comorbid CU traits in children with ADHD were associated with reduced differentiation between naturalistic social sounds and brief beeps in terms of the pupil dilation response. These results contribute to our understanding of visual attention in ADHD and comorbid conditions and have implications for treatment and interventions.

johan.lundin.kleberg@ki.se

REFERENCES

Alahyane, N., Lemoine-Lardennois, C., Tailhefer, C., Collins, T., Fagard, J., & Doré-Mazars, K. (2016). Development and learning of saccadic eye movements in 7-to 42-month-old children. *Journal of vision*, 16(1), 6-6.

Symposium 3
Monday, August 19th 14:00 – 16:00 Room 4

Progress in Neurophysiology and Oculomotor Psychophysics: Contributions and Influence of Jan van Gisbergen (1943-2019)

John van Opstal *(Radboud University Nijmegen, NL),* **Dan Guitton** *(McGill University, Montréal, Québec, Can),* **Doug Munoz** *(Queens University, Kingston, Ontario, Can),* **Pieter Medendorp** *(Radboud University, Nijmegen, NL), and* **Maarten Frens** *(Erasmus University, Rotterdam, NL)*

Neurophysiological studies of the oculomotor system, in particular the saccadic eye-movement system and the vestibular ocular reflex, have yielded unique and detailed insights into the neural mechanisms of how the brain selects, plans, programs and executes goal-directed gaze shifts, and how it maintains stable fixation and spatial awareness despite unpredictable and often complex self-motion trajectories of the head and body through space. In the early seventies, Dave Robinson paved the way for his pioneering approaches in neurocomputational neuroscience, by introducing the systems-theoretical paradigm and quantitative modeling into the oculomotor field. Dave died in November 2016, at the age of 92, but he left behind a large group of oculomotor researchers that have successfully followed this tradition.
Jan van Gisbergen was his postdoc in 1975-1976, and together they published a highly influential paper on the monkey saccadic pulse generator in the oculomotor brainstem (Van Gisbergen et al., 1981), which has inspired many researchers in the eye-movement field ever since. Jan passed away on March 8, 2019, at the age of 75. In this symposium, the contributors will discuss how the neurophysiological and behavioural approaches in their own work, both in the past and present, relate to the legacy of Jan's contributions, and to Robinson's neurocomputational approaches.

Saccadic eye movements serve the purpose to bring the fovea as fast and as accurately as possible on a peripheral target of interest. They are extremely fast (up to 700 deg/s in humans, and 1300 deg/s in monkey), with movement durations well below 100 ms. As a result, their accuracy cannot be controlled by visual feedback, which would be far too slow. Yet, saccades remain accurate to flashed targets in darkness, after intervening eye movements (Hallett and Lightstone, 1976; Sparks and Mays, 1980), and even after midflight perturbations in complete darkness (Keller and Edelman, 1994). In addition, their kinematics are described by the so-called main-sequence (a saturating saccade amplitude - peak velocity relation), which betrays the existence of a nonlinear controller in the system. It has been widely agreed in the oculomotor field that saccades are driven by an internal dynamic feedback circuit, and that the main-sequence kinematics are generated by a central nonlinear pulse generator. The Van Gisbergen et al (1981) model provided a detailed account of the neural mechanisms underlying the feedback control circuit of saccades, in which the excitatory (EBNs) and inhibitory (IBNs) burst neurons in the pons embody the nonlinear pulse generator. According to this seminal model, these burst neurons are driven by a dynamic motor-error signal, which is the ongoing difference between the desired target location (input to the circuit) and current eye position (output of the circuit), and the burst-neuron output reflects the instantaneous velocity of the eye (the saccade pulse).
The input to the brainstem circuitry is a target representation in head-centered coordinates, which reflects the spatial accuracy of saccades to target sequences, and readily incorporates the spatial remapping of targets that is needed after intervening eye movements. The model accurately accounted for many aspects of saccade generation, but a major problem has been its lack for an explicit role of the midbrain Superior Colliculus (SC), which is known to play a central role in gaze shifts, and top control the saccadic brainstem. The SC, however, issues a fixed saccade displacement vector (i.e., in oculocentric coordinates), rather than a desired eye-position signal (in head-centered coordinates). Later work, for example that of Scudder and colleagues (1988) reformulated the original head-centered Van Gisbergen model by including the SC in a neurobiologically plausible way; the Scudder model drives the saccade entirely in oculocentric coordinates. In his later work, Jan van Gisbergen proposed a quantitative mathematical model for the SC motor map, which could also explain how a large population of activated neurons would encode the upcoming saccade vector (Ottes et al., 1986). It also provided a quantitative account for the shape and size of SC movement fields.

John van Opstal will discuss his early and recent work on the midbrain Superior Colliculus, which he already started as his PhD project in Jan's lab in the 80's. He will describe how their psychophysical data and modelling efforts on oblique saccade control led to the concept of the existence of a nonlinear vectorial pulse generator

in the saccadic system, which elegantly explains the strong and systematic cross-coupling observed in oblique saccades, and their remarkably straight trajectories, as an emerging property of the system. We speculated (in 1989) that the SC motor map would be in an ideal position within the system to embed the vectorial pulse generator. However, because at that time SC responses still lacked a clear (cor)relation with the saccade kinematics, let alone their instantaneous kinematics, the idea remained highly controversial. Here he will discuss his more recent work on neural responses in the colliculus during perturbed saccades, which culminated into a better understanding of the saccadic pulse generator, and how the potential kinematic role of the SC could also be beneficial for understanding eye-head gaze control.

Dan Guitton has published many studies on saccadic eye-head gaze shifts, the role of feedback and the SC in controlling these gaze shifts, and co-authored a seminal series of papers on the brainstem EBNs and IBNs ('97) that followed up on the earlier '81 work of Jan and Dave. In this symposium, he will discuss his current work on spatial remapping of visual receptive fields in the visuomotor system. He will argue how the mechanism of target remapping in response to intervening eye movements subserves perceptual stability and perceptual learning across saccades, also in hemidecorticate patients who lack vision in one hemifield, yet respond to visual stimuli presented in that hemifield. He explains how Jan's complex-logarithmic model of the SC motor map may hel to understand this remarkable behaviour.

Doug Munoz probes the monkey saccadic system at different levels, including the basal ganglia, frontal cortex, and the SC. He uses a multimodal approach, in which targeting saccades, anti-saccades, fixation, micro-saccades and pupil dilation together reflect the underlying mechanisms of action selection, task difficulty, and response tasks that involve the processing of emotional stimuli. He will discuss how action selection of saccades incorporates the concerted activity of the cortex and basal ganglia, and how the result of that process modulates the activity in the SC motor map.

Pieter Medendorp works on spatial awareness. In his experiments and computational modeling studies he aims to understand how the vestibular system, in particular the otoliths, stimulated by passive whole-body motion, influence action selection of multiple effectors, including the eyes and hands, to subserve goal-directed spatial behaviour and spatial remapping mechanisms. He collaborated in his vestibular studies with Jan van Gisbergen, and he will discuss how current statistical approaches, like Bayesian inference, and the experimental manipulation of prior information about the environment and about self-motion help to understand the multimodal contributions of different, often noisy, sensory and motor signals in guiding purposeful behavior.

Maarten Frens studies the complex eye-movement patterns in visual search across a cluttered visual display with many competing distracters, as well as arm movement control. Here he will discuss his current cohort studies, in which he quantified the development of visual search strategies of the human oculomotor system as a function of age, and especially how the efficiency of visual search appears to accelerate during adolescence.

References

HALLETT, P. E. AND LIGHTSTONE, A. D. Saccadic eye movements toward stimuli triggered by prior saccades. *Vision Res*. 16: 88–106, 1976

KELLER, E.L. AND EDELMAN, J.A. Use of interrupted saccade paradigm to study spatial and temporal dynamics of saccadic burst cells in the superior colliculus in monkey. *J Neurophysiol* 72: 2754–2770, 1994

MAYS, L.E. AND SPARKS, D.L. Saccades are spatially, not retinocentrically, coded. *Science* 208: 1163–1165, 1980

OTTES, F.P., VAN GISBERGEN, J.A.M., AND EGGERMONT J.J. Visuomotor fields of the superior colliculus: a quantitative model. *Vision Res* 12: 1795–1808, 1986

SCUDDER, C.A. A new local feedback model of the saccadic burst generator. *J. Neurophysiol.* 59: 1455–1475, 1988

VAN GISBERGEN J.A.M., ROBINSON D.A., AND GIELEN C.C.A.M. A quantitative analysis of generation of saccadic eye movements by burst neurons. *J. Neurophysiol*. 45: 417–442, 1981

Symposium 4
Tuesday, August 20th 10:30 – 12:30 Plenary room

From lab-based studies to eye-tracking in virtual and real worlds: conceptual and methodological problems and solutions

Organizer: Dr. R.S. Hessels
Experimental Psychology, Helmholtz Institute, Utrecht University, the Netherlands

Summary

Wearable mobile eye trackers have great potential as they allow the measurement of eye movements during daily activities such as driving, navigating the world and doing groceries. Although mobile eye trackers have been around for some time (e.g. Land & Lee, 1994; Pelz & Canosa, 2001), developing and operating these eye trackers was generally a highly technical affair. As such, mobile eye-tracking research was not feasible for most labs. Nowadays, many mobile eye trackers are available from eye-tracking manufacturers (e.g. Tobii, Pupil labs, SMI, Ergoneers) and various implementations in virtual/augmented reality have recently been released.
The wide availability has caused the number of publications using a mobile eye tracker to increase quickly. Mobile eye tracking is now applied in vision science, educational science, developmental psychology, marketing research (using virtual and real supermarkets), clinical psychology, usability, architecture, medicine, and more. Yet, transitioning from lab-based studies where eye trackers are fixed to the world to studies where eye trackers are fixed to the head presents researchers with a number of problems. These problems range from the conceptual frameworks used in world-fixed and head-fixed eye tracking and how they relate to each other, to the lack of data quality comparisons and field tests of the different mobile eye trackers and how the gaze signal can be classified or mapped to the visual stimulus. Such problems need to be addressed in order to understand how world-fixed and head-fixed eye-tracking research can be compared and to understand the full potential and limits of what mobile eye-tracking can deliver.
In this symposium, we bring together presenting researchers from five different institutions (Lund University, Utrecht University, Clemson University, Birkbeck University of London and Rochester Institute of Technology) addressing problems and innovative solutions across the entire breadth of mobile eye-tracking research. *Hessels et al.* present data on the definitions of fixations and saccades held by researchers in the eye-movement field and argue how they need to be clarified in order to allow comparisons between world-fixed and head-fixed eye-tracking research. *Diaz et al.* introduce machine-learning techniques for classifying the gaze signal in mobile eye-tracking contexts where head and body are unrestrained. *Niehorster et al.* compare data quality of mobile eye trackers during natural behavior and discuss the application range of these eye trackers. *Duchowski et al.* introduce a method for automatically mapping gaze to faces using computer vision techniques and *Haensel et al.* present a novel data-driven method for statistical analysis of mobile eye-tracking data in a dyadic social interaction study. Finally, *Pelz et al.* employ state-of-the-art techniques to map fixations to objects of interest in the scene video and align grasp and eye-movement data in the same reference frame to investigate the guidance of eye movements during manual interaction.
The six presentations in this symposium provide both novel directions for researchers who have already been active in mobile eye-tracking research, and a comprehensive foundation for researchers considering a transition into mobile eye tracking in virtual and real worlds in the near future.

Individual talks

Is the eye-movement field confused about fixations and saccades? A survey among 124 researchers

Roy S. Hessels[1,2], Diederick C. Niehorster[3,4], Marcus Nyström[3], Richard Andersson[5], Ignace T. C. Hooge[1]
1 *Experimental Psychology, Helmholtz Institute, Utrecht University, Utrecht, The Netherlands*
2 *Developmental Psychology, Utrecht University, Utrecht, The Netherlands*
3 *Lund University Humanities Lab, Lund University, Lund, Sweden*
4 *Department of Psychology, Lund University, Lund, Sweden*
5 *Tobii Pro AB, Stockholm, Sweden*
Corresponding author: royhessels@gmail.com

Background: Eye movements have been extensively studied in a wide range of research fields. While new methods such as mobile eye tracking and eye tracking in virtual/augmented realities are emerging quickly, the

eye-movement terminology has scarcely been revised. We assert that this may cause confusion about two of the main concepts: fixations and saccades.
Methods: We conducted a survey among 124 eye-movement researchers to assess the definitions of fixations and saccades held in the eye-movement field.
Results: Eye-movement researchers held a variety of definitions of fixations and saccades, of which the breadth seems even wider than what is reported in the literature. Definitions were not related to researcher background or experience.
Conclusion: We urge researchers to make their definitions more explicit by specifying all the relevant components of the eye movement under investigation: (i) the oculomotor component: whether the eye moves slow or fast; (ii) the functional component: what purposes does the eye movement serve; (iii) the coordinate system used: relative to what does the eye move; (iv) the computational definition: how is the event represented in the eye-tracker signal. This should enable eye-movement researchers from different fields and using different eye trackers (world-fixed or head-fixed) to discuss without misunderstandings.

Supervised Learned for the Classification of Gaze Events Made During Natural Task Execution

Gabriel J Diaz, Rakshit S Kothari, Jeff B Pelz, Zhizhuo Yang, Chris Kanan, Reynold Bailey
Chester F. Carlson Center for Imaging Science, Rochester Institute of Technology, Rochester NY, USA
Corresponding author: Gabriel.Diaz@rit.edu

The study of visual-motor behavior benefits from the classification of gaze events into distinct categories, such as saccade, pursuit, and fixation. Although there are several solutions for the classification of the eye-in-head signal, the field is still in need of solutions for gaze classification contexts in which the head and body are unrestrained. Our approach was to apply supervised learning techniques to a custom dataset of eye+head behavior. Twenty subjects were instrumented with a head-mounted 6-axis 100 Hz inertial measurement unit, a 30 Hz ZED stereo camera, and a 120Hz Pupil labs eye tracker as they made tea, walked down a hallway, performed mobile visual search, or caught a ball. This yielded over 6 hours of recorded behavior, approximately 2 hours 20 mins of which was manually annotated by 5 labelers. A subset of labelled data was used to train a series of machine learning gaze-event classifiers. The performance of our classifier was evaluated using various event based metrics, which revealed near-human level classification levels. Moreover, we found that fixation-pursuit confusion is largely resolved through the incorporation of head movement data.

When glasses slip: Data quality of mobile eye trackers during natural behavior

Diederick C. Niehorster[1,2], Thiago C. Santini[3], Roy S. Hessels[4,5], Ignace T. C. Hooge[4], Enkelejda Kasneci[3], Marcus Nyström[1]
1 *Lund University Humanities Lab, Lund University, Lund, Sweden*
2 *Department of Psychology, Lund University, Lund, Sweden*
3 *Perception Engineering, University of Tübingen, Tübingen, Germany*
4 *Experimental Psychology, Helmholtz Institute, Utrecht University, Utrecht, The Netherlands*
5 *Developmental Psychology, Utrecht University, Utrecht, The Netherlands*
Corresponding author: diederick_c.niehorster@humlab.lu.se

Mobile head-worn eye trackers allow researchers to record eye-movement data while a participant freely moves around and interacts with their surroundings. However, is the data quality of these devices affected by participant activity during natural behavior? To answer this question, we designed testing scenarios mimicking participant natural behaviors that can cause a mobile eye tracker to slip relative to the participant's head. Specifically, for four mobile eye-tracking setups – Pupil Labs hardware with Pupil Capture (3D) and EyeRecToo software, SMI glasses 60Hz and Tobii Pro Glasses 2 at 50Hz – we investigated data quality when participants talk, make facial expressions, adjust the eye tracker, and reposition the eye tracker after it has slipped. Our results show that only the Tobii Glasses 2's gaze estimates remained stable across tasks. All other setups exhibited large errors in recorded gaze position that increased with glasses slip magnitude, and were over one degree even for the small amounts of glasses slip that occurred during speech and facial expressions. We recommend that users of mobile eye trackers perform similar tests with their devices to become aware of their setup's characteristics with an aim to design experiments that are robust to its limitations.

Automatic Face Detection for Applied Eye-Tracking Studies

Andrew T. Duchowski[1], Nina A. Gehrer[2], Michael Schönenberg[2], Krzysztof Krejtz[3]
1 Visual Computing, Clemson University, USA
2 Clinical Psychology & Psychotherapy, University of Tübingen, Germany
3 Psychology, SWPS University of Social Sciences & Humanities, Poland
Corresponding author: duchowski@clemson.edu

Automatic Area Of Interest (AOI) demarcation of facial regions is not yet commonplace in applied eye-tracking research (e.g., during social interaction), partially because face detection methods are prone to error. The current eye-tracking state-of-the-art concerning face scanning relies on manual frame-by-frame labeling of facial AOIs. Although an early study used automated software for face detection (developed at the Fraunhofer Institute for Integrated Circuits), the software was otherwise unnamed. More recent work used OpenFace software to detect 68 facial landmarks from which a Voronoi diagram was constructed. Voronoi cells (polygons) acted as facial AOIs. A limitation of the Voronoi tessellation is that the regions extend to infinity (they are borderless). This may hinder delineation of gaze from falling in a facial region or outside the face.
We present a method built from OpenCV open-source software suite that uses Haar cascaded filters to detect the face, eyes, mouth, and nose. As this approach is prone to error (i.e., a large number of false positives), we employ heuristics derived from art to eliminate regions that are improbable, thus eliminating false positives. The method is fully automatic, appears to be fairly stable, and tracks multiple faces in the scene.

Cultural differences in naturalistic face scanning: a data-driven analysis method for mobile eye-tracking

Jennifer X. Haensel[1], Matthew Danvers[1], Mitsuhiko Ishikawa[2], Shoji Itakura[2], Raffaele Tucciarelli[1], Tim J. Smith[1], Atsushi Senju[1]
1 Department of Psychological Sciences, Birkbeck, University of London, UK
2 Department of Psychology, Kyoto University, Japan
Corresponding author: jhaens01@mail.bbk.ac.uk

Face scanning strategies during social interactions have exclusively been examined using the area-of-interest (AOI) approach, which involves the selection of individual facial regions and spatial pooling of gaze data to compute fixation times separately for each area. Although this represents a statistically sensitive method for detecting differences between groups or conditions, pre-defining the number and locations of AOIs can hinder novel insights. Data-driven methods provide an alternative approach, but such techniques do not currently exist for mobile eye-tracking given that changes in AOI position, size, and angle between scene frames complicate collapsing data across time and/or participants.
We conducted a face-to-face interaction study using mobile eye-tracking to compare face scanning patterns of British and Japanese participants, providing also a solution to perform data-driven analysis. We identified faces semi-automatically and subsequently mapped face regions and corresponding gaze points to a unified coordinate space, allowing fixations to be collapsed across time and participants. Monte Carlo permutation testing was applied to statistically compare the resulting density maps, revealing greater eye scanning in Japanese participants and more mouth looking in British individuals. This demonstrates cultural differences in naturalistic face scanning for the first time and highlights the applicability of data-driven methods for exploratory purposes.

Analysis of Gaze and Grasp Behavior Recorded with a Mobile Eye Tracker and Aligned within a Common Reference Frame

Jeff B Pelz and Gabriel J Diaz
Chester F. *Carlson Center for Imaging Science, Rochester Institute of Technology, Rochester NY, USA*
Corresponding author: pelz@cis.rit.edu

When engaging in visually guided behavior, sequential eye movements gather the task-relevant information related to decision making and the motor-relevant information needed to guide movements of the hands. We present a new technique to study these distinct types of eye movements during natural interaction with 2D printed Objects Of Interest (OOIs), as participants performed basic tasks, such as sorting printed items by indicated numerical value. Gaze behavior was monitored by a head-mounted Pupil Labs eye tracker, which

recorded gaze position at 120 Hz, and the ego-centric view of the scene at 60 Hz. Prior to analysis, a custom algorithm was used to map fixations to OOIs detected in the scene video to a static photomontage of all OOIs used in the study. Similarly, custom computer vision algorithms were used to identify when the hands were overlaid upon an OOI, and used to align grasp data within the same photomontage. The result is gaze and grasp data aligned within a single, static frame of reference, regardless of the original orientation of the 2D printed media in the scene view. Analysis focused on the role of task, salience, and motor-relevance of information sources in guiding eye movements during manual interaction.

References

Land, M. F., & Lee, D. N. (1994). Where we look when we steer. *Nature*, 369(6483), 742- 744.
Pelz, J. B., and Canosa, R. (2001). Oculomotor behavior and perceptual strategies in complex tasks. *Vision research*, 41, 3587-3596.

Symposium 5
Tuesday, August 20th 14:00 – 16:00 Room 4

Recent advances in research on the “looking at nothing” phenomenon

Organizers: Agnes Rosner[1], Roger Johansson[2]
[1]University of Zurich, [2]Lund University

An extensive body of research has shown that memory retrieval promotes eye movements toward locations that have previously been associated with sought-after information. The nature of this “looking at nothing” (LAN) behavior has given rise to a heated debate over the last decade and has stimulated researchers from different fields to explain this intriguing eye movement behavior (cf., Ferreira, Apel, & Henderson, 2008; Richardson, Altmann, Spivey, & Hoover, 2009). A key question is whether LAN is merely an epiphenomenon that can be used to trace internal processing or whether it also serves an active role for memory retrieval. As current research has demonstrated that gaze positions showing compatibility between encoding and recall can increase the likelihood of successful remembering of both visual (Johansson & Johansson, 2014) and verbal information (Scholz, Mehlhorn, & Krems, 2016), there is a compelling case for the latter. But our knowledge is still very limited when it comes to the underlying mechanisms. Nonetheless, research on LAN has over the last years spurred important advances within research on episodic memory, working memory, decision making, language processing, and event cognition. This symposium thus aims to bring together recent research on LAN from top researchers within different research fields and offers a unique platform to discuss the state of the art in research on the LAN phenomenon.

Alper Kumcu studies word memory and outlines how LAN is differentially associated with specific lexico-semantic properties during word retrieval and also shows that LAN more strongly occurred in people who were weak in visuospatial memory ability.

Agnes Rosner studies decision making and investigates LAN in a categorization task with verbal materials. It is demonstrated that LAN more strongly occurred when the material was presented aurally as compared to visually, and that there were no differences in LAN when materials were learned by heart as compared to when it was only being repeatedly presented.

Manon Jones studies visual-phonological associations and presents a new field of application for LAN, where it can be used as a diagnostic tool to study how typical and dyslexic readers differ when recalling word-symbol associations. Additionally, results demonstrate how the influence of LAN upon memory retrieval was highly sensitive to location regularities and repetition effects, but to different extents when comparing typical and dyslexic readers.

Stefan Czoschke studies visuospatial working memory and investigates LAN in relation to short term retention of visual material. Novel results reveal how eye movement control adapts to optimize memory maintenance in the face of interference from competing material.

Roger Johansson studies LAN in relation to episodic memory retrieval and investigates how more complex scanpath components over and above simple gaze locations contribute to episodic remembering of visual scenes and object arrangements. Results demonstrate how a multitude of spatio-temporal components contribute to successful remembering, to different extents, and in different ways depending on the stimulus type.

In the final discussion, lead by Agnes Rosner and Roger Johansson, they will summarize the state of the art in research on LAN and outline directions for future research.

(1) Looking for language in space: eye movements to blank locations when re-membering words

Alper Kumcu[1], Robin Thompson[2]
[1]Hacettepe University, [2]University of Birmingham

People tend to look at uninformative, blank locations in space when retrieving information. This gaze behaviour, known as looking at nothing, is assumed to be driven by the use of spatial indices associated with external information. The aim of our research is to explore looking at nothing in memory for words. In two experiments, we investigated: (1) whether people look at blank locations when remembering visually and simultaneously presented single nouns as demonstrated for other types of visual/verbal information and (2) whether individual differences (e.g., verbal and visuospatial memory span) and lexico-semantic differences between words to be remembered (e.g., imageability) modulate eye movements to blank locations. In both experiments,

participants were presented four nouns on a two by two grid in a yes/no recognition memory paradigm. Results demonstrated more looks to the relevant, blank location, where the probe word had appeared previously, longer than the other blank locations during retrieval. Importantly, participants with better visuospatial memory and thus, richer internal sources relied less on the blank locations when remembering words. Further, more looks to blank locations were revealed during the retrieval of less imageable and abstract words. Results are interpreted within the framework of grounded-embodied approaches to language and memory.

(2) Top-down and bottom-up guidance of visual attention during categorization decision making

Agnes Rosner, Bettina von Helversen
University of Zurich

During categorization decision making, people look at blank spatial locations that have been associated with information about previous category members (exemplars). In this study, we systematically investigated the interaction of the „looking at nothing "-phenomenon (LAN) with the accessibility of information in memory and the presentation format during testing. In two experiments, participants repeatedly decided whether to invite job candidates for interviews. In one condition, participants learned the exemplars' attributes by heart before learning to classify the exemplars, in the other conditions, the exemplars' attributes were learned incidentally during the classification training. Additionally, we varied whether exemplars were presented visually or auditorily (Study 1) and how long the visual presentation lasted (Study 2). LAN during the categorisation of new candidates occurred when exemplars were learned by heart and when only being repeatedly presented. LAN also occurred when presenting information auditorily and when removing visual information from screen after 1.5 seconds. However, when visually presenting new test items without removing information, LAN did not occur. The results shed light on the interaction between visuospatial attention and attention to information in memory during categorisation decision making as well as on the memory strength needed to elicit LAN to exemplar locations.

(3) Episodic traces and statistical regularities: Paired associate learning in typical and dyslexic readers

Manon Jones[1], Jan-Rouke Kuipers[2], Gary Oppenheim[1]
[1]Bangor University, [2]University of Stirling,

Learning visual-phonological associations is a key skill underlying successful reading acquisition. However, we are yet to understand the cognitive mechanisms that enable efficient learning in good readers, and those which are aberrant in individuals with developmental dyslexia. Here, we use a repeated cued-recall task to examine how typical and reading-impaired adults acquire novel associations between visual and phonological stimuli, incorporating a looking-at-nothing paradigm to probe implicit memory for target locations. Cued recall accuracy revealed that typical readers' recall of novel phonological associates was better than dyslexic readers' recall, and it also improved more with repetition. Eye fixation-contingent error analyses suggest that typical readers' greater improvement from repetition reflects their more robust encoding and/or retrieval of each instance in which a given pair was presented: whereas dyslexic readers tended to recall a phonological target better when fixating its most recent location, typical readers showed this pattern more strongly when the target location was consistent across multiple trials. Thus, typical readers' greater success in reading acquisition may derive from their better use of statistical contingencies to identify consistent stimulus features across multiple exposures. We discuss these findings in relation to the role of implicit memory in forming new visual-phonological associations as a foundational skill in reading, and areas of weakness in developmental dyslexia.

(4) Splitting retro-cue activation and foveal attention in visual-spatial working memory

Stefan Czoschke, Sebastian Henschke, Laura Rerko, Elke B. Lange
Max-Planck-Institute for Empirical Aesthetics

Attention is a central concept to describe selection mechanisms when information processing is capacity-limited. Whereas attention often is conceptualized to be directed outward (towards external space), models of working memory posit that attention is also directed inward, e.g. to prioritize memory representations as

measured in the retro-cue design. We present two experiments that investigate, how retro-active covered attention shifts are related to gaze shifts during memory retention. In Exp. 1, we investigated spontaneous gaze behavior in the retro-cue paradigm. Subjects encoded five colors and received a 100% valid cue that marked the to-be-recalled item during retention. Eye movements were strongly inhibited prior to the cue but released afterwards, indicating that saccadic activity might interfere with memory representations before the cue, and are therefore avoided. After the cue, prioritization might shield memory from interference. To test this directly, we introduced an uninformative saccade cue towards item positions, which was presented either before or after the retro-cue (Exp. 2). In agreement with the spontaneous viewing behavior in Exp. 1, saccades before, but not after the retro-cue impaired memory performance for non-saccade targets. Our results demonstrate that the oculomotor system is strongly related to internal attentional processes. Eye-movement control adapts to optimize memory maintenance: inhibition to prevent interference from competing representations, but release of inhibition, once a representation is prioritized for recall.

(5) How scanpath components present in eye movement reinstatements differentially contribute to visuospatial recollections

Roger Johansson[1], Marcus Nyström[1], Richard Dewhurst[2], Mikael Johansson[1]
[1]*Lund University,* [2]*Aarhus University*

An extensive body of research has demonstrated that episodic remembering involves eye movements that largely reinstate the gaze patterns that were established during encoding. Current research has further shown that fixation locations that overlap between encoding and recall promote cortical episodic reconstruction and successful remembering. While such findings indicate an active role for gaze location during episodic reconstruction, virtually nothing is known about how scanpath properties over and above simple gaze locations, such as order, direction, shape, length and duration, contribute to episodic reconstruction. The present study investigated the encoding-retrieval overlap in scanpaths for 60 participants who encoded and recalled 36 images of two types: scenes and object arrangements. By combining subjective ratings of memory quality with a surprise test of forced-choice recognition, we demonstrate how a multitude of spatio-temporal components contribute to successful remembering to different extents, and in different ways depending on the stimulus type. Results indicate that scanpath shape contributes to reconstructing the overall scene structure whereas scanpath position, order and direction contribute to reconstructing the arrangement of individual objects in a spatial context. To our knowledge, this is the first systematic demonstration of how eye movement reinstatements contribute to episodic remembering in a multifaceted way.

References:

Ferreira, F., Apel, J., & Henderson, J. M. (2008). Taking a new look at looking at nothing. *Trends in Cognitive Sciences*, 12, 405-410.
Johansson, R., & Johansson, M. (2014). Look here, eye movements play a functional role in memory retrieval. *Psychological Science*, 25, 236-242.
Richardson, D. C., Altmann, G. T. M., Spivey, M. J., & Hoover, M. A. (2009). Much ado about eye movements to nothing: A response to Ferreira et al.: Taking a new look at looking at nothing. *Trends in Cognitive Sciences*, 13, 235-236.
Scholz, A., Mehlhorn, K., & Krems, J. F. (2016). Listen up, eye movements play a role in verbal memory retrieval. *Psychological Research*, 80, 149-158.

Symposium 6
Wednesday, August 21st 10:30 – 12:30 Room 4

Eye movements during the reading of narrative and poetic text

Organizers: Jana Lüdtke & Arthur M. Jacobs
E-mail corresponding author: jana.luedtke@fu-berlin.de

Participants:
1 Johanna Kaakinen
2 Shuwei Xue, Jana Lüdtke & Arthur M. Jacobs
3 Lynn S. Eekhof, Moniek M. Kuijpers, Myrthe Faber, Xin Gao, Marloes Mak, Emiel van den Hoven, Roel M. Willems
4 Lilla Magyari, Anne Mangen, Anezka Kuzmičová
5 Teresa Sylvester & Jana Lüdtke
6 Marion Fechino & Arthur M. Jacobs

Abstract
Despite a wealth of studies using eye tracking to investigate mental processes during vision or reading, the investigation of oculomotor activity during natural reading of longer texts –be it newspaper articles, narratives or poetry– is still an exception in this field (as evidenced by the program of ECEM 2017 in Wuppertal). Following up on our symposium at ECEM 2017, here we'd like to bring together eye movement research on natural text reading to report recent progress in a coordinated way sharing data, experiences and software skills in this highly complex subfield. More specifically, in this symposium we will address several challenges faced by an eye tracking perspective on the reading of longer texts which involve a surplus of intervening variables and novel methods to analyze the data. In particular, the following issues will be addressed:

- Which text-analytical and statistical methods are best to deal with the myriad of surface and affective-semantic features potentially influencing eye movements during reading of 'natural' texts?
- What are the pros and cons of using machine learning assisted predictive modeling as an alternative to the standard GLM/LMM frameworks?
- Which kind of theoretical models can deal with the level of complexity offered by reading longer natural texts?

The five papers in this symposium all provide answers to these questions which also are of more general interest to the eye tracking community.

1 Johanna K. Kaakinen
University of Turku, Finland
E-mail corresponding author: johkaa@utu.fi

Emotional arousal during listening and reading of fictional narratives: Evidence from concurrent recordings of eye movements and psychophysiological measures

In this study, I examined how the emotional experience induced by fictional narratives is reflected in readers' eye movements. In two experiments, participants listened (Experiment 1, N=37) or read (Experiment 2, N=40) negatively-valenced (n= 12) and neutral excerpts (n=12) from Stephen King short stories. Pupil size, eye movements (only in Experiment 2), electrodermal activity (EDA), and photoplethysmography (PPG) were simultaneously recorded during listening and reading of fictional narratives. Participants rated the valence and arousal induced by each text with SAM (Bradley & Lang, 1994) and filled the transportation scale short form (Appel, Gnambs, Richter & Green, 2015). The results of Experiment 1 showed that negative texts induce a linear increase in arousal, and result in higher transportation than neutral texts. The data analyses of Experiment 2 will reveal the relationship between fluctuation in emotional arousal induced by text and eye movements during reading. The present results will provide novel information about the role of reader emotion in eye movement control during reading. The results will also be discussed in the light of current theories of text comprehension (e.g., Kinstch, 1998), which at present do not adequately address the role of reader emotion in constructing meaning from text.

2 Shuwei Xue, Jana Lüdtke, Teresa Sylvester & Arthur M. Jacobs
Department of Experimental and Neurocognitive Psychology, Freie Universität Berlin
E-mail corresponding author: jana.luedtke@fu-berlin.de

Eye Tracking Shakespeare Sonnets: Combining Quantitative Narrative Analysis and Predictive Modeling

In this study we analyzed the eye movement behavior of participants reading three Shakespeare sonnets as a

function of a multitude of psycholinguistic features extracted via Quantitative Narrative Analysis (QNA). The results of a competitive statistical analysis, comparing different models and highlighting a machine learning-based predictive modeling approach identify a number of important predictors of mean total reading time and fixation probability most of which are novel to this field of research, e.g. the sublexical feature ‘sonority score’ or the lexico-semantic feature ‘aesthetic potential’. We discuss the results with regard to both methodological and theoretical limitations in mainstream eye tracking research and propose ideas for how to tackle these limitations when investigating the reading of longer natural texts.

3 Lynn S. Eekhofa, Moniek M. Kuijpersb, Myrthe Faber[c, d,] Xin Gaoa, Marloes Maka, Emiel van den Hovene, Roel M. Willemsa, [c, f]

a Centre for Language Studies, Radboud University Nijmegen, The Netherlands
b Digital Humanities Lab, University of Basel, Switzerland
c Donders Institute for Brain, Cognition and Behaviour, Radboud University Nijmegen, The Netherlands
d Radboud University Medical Hospital, Department of Cognitive Neuroscience, Nijmegen, The Netherlands
e Center for Cognitive Science, University of Freiburg, Germany
f Max Planck Institute for Psycholinguistics, Nijmegen, The Netherlands
E-mail corresponding author: L.Eekhof@let.ru.nl

Lost in a story, detached from the words: Absorbed readers are less sensitive to word characteristics during narrative reading

Successful reading goes beyond the coding and decoding of words. One important aspect of the pleasure related to reading is being absorbed in a story. Here, we investigated the relationship between basic eye movements during reading and the more subjective aspects of the reading experience. Specifically, we focused on how sensitivity to low-level lexical word characteristics—measured as the effect of these characteristics on gaze durations—was related to narrative absorption and appreciation. Leveraging a large data set consisting of three previous eye tracking experiments in which participants (N = 171) read literary narratives, we first established that, as expected, word frequency, age of acquisition, and orthographic neighborhood size of words influenced gaze durations. More importantly, we found that the degree of sensitivity to these word characteristics was negatively related to self-reported narrative absorption, appreciation and print exposure. We speculate that readers whose reading behavior is less affected by word characteristics have more resources available to build a rich situation model or mental story world. Our study illustrates the importance of linking basic aspects of reading to the subjective reading experience.

4 L. Magyari1,2, A. Mangen3, A. Kuzmičová4,5

1 Department of Cognitive Psychology, Faculty of Education and Psychology, Eötvös Loránd University, Budapest, Hungary
2 Neuroethology of Communication “Lendület” Research Group, Department of Ethology, Institute of Biology, Eötvös Loránd University and Hungarian Academy of Sciences, Budapest, Hungary
3 Norwegian Reading Centre, University of Stavanger, Stavanger, Norway
4 Department of Culture and Aesthetics, Stockholm University, Sweden
5 Institute of Literature, Czech Academy of Sciences, Czech Republic
E-mail corresponding author: lillamagyari@gmail.com

Eye-movement behavior during reading of literary texts with different narrative styles

Based on Kuzmičová’s (2014) phenomenological typology of narrative styles, we studied the specific contributions of mental imagery to literary reading experience and to reading behavior by questionnaires and eye-tracking methodology. Specifically, we focused on the two main categories in Kuzmičová’s (2014) typology, i.e., texts dominated by an “enactive” style, and texts dominated by a “descriptive” style. “Enactive” style texts render characters interacting with their environment, and “descriptive” style texts render environments dissociated from human action. Excerpts from novels were used as stimuli. Our initial analysis of the results showed that ratings on imagery scales were higher for the enactive style texts and reading speed was associated with higher number of verbs on pages with enactive style text. In our presentation for this symposium, we will go into more detail about these findings. In particular, we will focus on the possible sources of differences in reading speed (e.g. shorter fixation vs less backward regressions).

5 Teresa Sylvester & Jana Lüdtke

Department of Experimental and Neurocognitive Psychology, Freie Universität Berlin
E-mail corresponding author: teresa.sylvester@fu-berlin.de, jana.luedtke@fu-berlin.de

When Stephen King picks up Margarita: Eyetracking in self-paced reading of passages from novels

Iser (1972) described reading as a creative act co-determined by features of the text, the reader and the reading situation. Surprisingly, until today only a few studies have used eye tracking during reading of literary texts to study this complex interaction and resulting key phenomena, such as immersion or aesthetic appreciation. Since recent models of literary reading, e.g. the Neurocognitive Poetics Model (NCPM, Jacobs, 2015), offer specific hypotheses about reading behaviour, eyetracking is a valuable asset to ‚objectively' test them. In this study we manipulated the reading task (reading for understanding vs. proof reading) and crossed this factor with the presentation of two excerpts from literary texts differing in text complexity, suspense and overall emotional tone (authors: King vs. Bulgakow). The results of standard multiple regression analysis indicate that textual features, reading task, self-reported reading experiences and readers' personality traits influenced different aspects of reading behaviour, e.g. reading speed. The latter was positively correlated with self-reported immersion but not with text difficulty. We reflect our results in the light of the NCPM and discuss the applicability of new approaches (predictive modeling) to the analysis of features from interlexical levels usually associated with a relatively small sample size.

Iser, W. (1972). The reading process: A phenomenological approach. *New Literary History*, 3(2), 279-299.
Jacobs, A. M. (2015). Towards a neurocognitive poetics model of literary reading, in Towards a Cognitive Neuroscience of Natural Language Use, ed. R. Willems (*Cambridge: Cambridge University Press*), 135–159.

6 Marion Fechino & Arthur M. Jacobs

Aix-Marseille Université and CNRS, France
Department of Experimental and Neurocognitive Psychology, Freie Universität Berlin
E-mail corresponding author: marion.fechino@univ-amu.fr

Following in Jakobson and Lévi-Strauss' footsteps: a neurocognitive poetics investigation of eye movements during the reading of Baudelaire's 'Les Chats'

Following Jakobson and Levi-Strauss famous analysis of Baudelaire's poem Les chats, in the present study we investigate the reading of French poetry from a Neurocognitive Poetics perspective. Our study is exploratory and a first attempt in French, most previous work having been done in either German or English (e.g., Jacobs, 2015a, 2018a,b). We varied the presentation mode of the poem Les chats (verse vs. prose form) and measured the eye- movements of our readers to test the hypothesis of an interaction between presentation mode and aesthetic appreciation of the poem. Guided by Jakobson and Levi-Strauss' qualitative analysis, we looked specifically at the effects of literary foregrounding features on standard eye movement parameters. Our results replicate those from previous English poetry studies in that there is a specific pattern in poetry reading with longer dwell times and more regressions in the verse than in the prose format. Moreover, presentation mode also matters for making salient parts of the poem with a high aesthetic potential. Finally, analysis of readers' questionnaire responses provides interesting new research questions. In general, this study generates promising hypotheses for further research applying quantitative narrative analysis to French poetry and developing the Neurocognitve Poetics Model of literary reading (NCPM; Jacobs, 2015a) into a cross-linguistic model of poetry reading.

Symposium 7
Wednesday, August 21st 14:00 – 16:00 Plenary room

Eye movements in developing readers: From basic research to classroom application.

CONTACT EMAILS

- **Chris Vorstius** *vorstius@uni-wuppertal.de*
- **Ronan Reilly** *ronan.reilly@nuim.ie*
- **Jochen Laubrock** *laubrock@uni-potsdam.de*
- **Jocelyn R. (Jill) Folk** *jfolk@kent.edu*
- **Julie Kirkby** *jkirkby@bournemouth.ac.uk*
- **Alexandra Spichtig** *alex@readingplus.com*

Eye-movement recording has made it possible to achieve a detailed understanding of oculomotor and cognitive behavior during reading and of changes in this behavior across the stages of reading development. Given that many students struggle to attain even basic reading skills, a logical extension of eye-movement research involves its applications in both the diagnostic and instructional areas of reading education. The focus of this symposium is on eye-movement research with potential implications for reading education.

Christian Vorstius will review results from a large-scale longitudinal study that examined the development of spatial parameters in fixation patterns within three cohorts, ranging from elementary to early middle school. The authors will discuss an early development window and its potential influences on reading ability and orthography. Cross-language comparisons will also be discussed.

Ronan Reilly and Xi Fan will present longitudinal data related to developmental changes in reading-related eye movements in Chinese. Their findings are indicative of increasing sensitivity to lexical predictability and sentence coherence. The authors suggest that delays in the emergence of these reading behaviors may signal early an increased risk of reading difficulty.

Jochen Laubrock's presentation will focus on perceptual span development and explore dimensions of this phenomenon with potential educational implications, such as the modulation of perceptual span in relation to cognitive load, as well as preview effects during oral and silent reading --and while reading comic books.

Jocelyn Folk and colleagues will present research related to eye-movements during orthographic and semantic learning involving novel words. Their characterizations of initial reading times and fixation probabilities may provide a basis for enhancing word learning in developing readers. Julie Kirkby and Shannon Babb will present research comparing the eye movements of dyslexic and skilled readers while word-copying. Their research not only provides insight into underlying cognitive processes in dyslexia, but also indicates a need to accommodate the learning styles of dyslexic readers during classroom instruction.

Finally, Alexandra Spichtig and colleagues will describe eye-movement data characterizing reading efficiency differences between proficient and non-proficient readers as identified by their state or local reading assessments, using a large sample of students in grades 2 through 12. These data highlight the usefulness of incorporating eye-movement data within an assessment process in order to gain a more complete picture of a student's reading challenges or success.

As computers and eye-tracking technology continue to improve, it is only a matter of time before the ability to record eye movements will be available in every classroom. Eye-movement researchers are in a position to assure that educators will be able to use this technology to quickly identify and address the needs of struggling students. This includes issues like suboptimal oculomotor control and/or allocation of attention, decoding inefficiencies, impoverished vocabulary, mind wandering, lack in comprehension monitoring etc. In the long run the goal is to develop adaptive forms of instruction focusing on the individual needs of each child. We anticipate that the research presented in this session will help to take us further down the path in this direction.

CONTRIBUTIONS

Title: Spatial attributes of saccades in developing readers: Evidence from longitudinal data

Author: Christian Vorstius[1], Ralph Radach[1], and Christopher J. Lonigan[2]

1 Bergische Universität Wuppertal, Department of Psychology

2 Florida State University, Department of Psychology

Abstract

Over the last decade, a growing number of studies investigated eye movements in developing readers. Extending findings from pioneering studies, a "developmental reading pattern" including decreasing fixation durations, lower

fixation and refixation probabilities, longer saccade amplitudes, and an increasing perceptual span has emerged. However, a detailed analyses of saccade metrics and the interplay of landing position, saccade amplitude, and launch distance is somewhat underrepresented. To this end, the current study examined the development of spatial parameters in fixation patterns from a longitudinal data set with 3 cohorts (1st-3rd grade n=80; 3rd-5th n=98; 5th-7th n=71). All children read English declarative sentences with identical content across grades. Results revealed that for beginning English readers, fixation patterns show a close resemblance to adult patterns with well-behaved saccade landing positions, a linear relationship between launch distance and fixation position, and u-shaped refixation curves. Most development is happening from 1st through 3rd grade and we present the first detailed account of changes within this period. While these findings are thought to be mostly related to visuo-motor constraints, further influences of reading ability and orthographic regularity effects are discussed. Finally, a comparison with German data will highlight important differences and similarities across languages.

Title: Using eye-movement data as a developmental measure in Chinese readers

Authors: Ronan Reilly & Xi Fan

Department of Computer Science, Maynooth University, Maynooth, Ireland

Abstract

We collected longitudinal data from developing readers of Chinese to quantify growth patterns in eye-movement parameters and relate them to lexical, sentential, and text characteristics (e.g., Levy, 2013).

The study involved 100 Grade 4 students (10 to 11 years), comprising three cohorts that differed in reading ability, but had similar IQ as measured by the Raven Standard Reasoning Test (RSRT). Participants read short passages displayed on a computer screen while their eyes were tracked using an SR-Research EyeLink 1000 eye-tracker. The three cohorts were tested twice, one a year apart, using age-appropriate texts with some overlap between texts used in the two data collections.

We use n-gram statistics to calculate surprisal measures of local word predictability (Levy, 2013) and embedding distance to measure sentence and paragraph level coherence (e.g., Sutskever et al., 2014). These are then used as predictors of various eye movement parameters and their change over time. The results show growth in sensitivity to supra-lexical properties such as lexical predictability and sentence coherence. Correlations are also apparent between reading ability and growth rate in some parameters, suggesting that these could be used as early warning of reading difficulty.

References

Levy, R. (2013). Memory and Surprisal in Human Sentence Comprehension. In van Gompel, Roger P. G., editors. *Sentence Processing* (pp. 78–114). Hove: Psychology Press.

Sutskever, I., Vinyals, X, & Le, Q. (2014). Sequence-to-sequence learning in neural networks. arXiv:1409.3215 [cs.CL]

Title: Flexible scheduling and the dynamics of parafoveal processing

Author: Jochen Laubrock

Universität Potsdam, Germany

Abstract

We present a review of our recent work on eye movement markers of processing upcoming information in reading from three complementary perspectives: our longitudinal study on perceptual span development, a comparison of oral and silent reading, and a corpus study of reading comic books. The perceptual span describes the useful field of view during reading. A qualitative jump in its development coincides with the transition from synthetic to word-form based reading. Initially good readers benefited more from this transition, so that the developmental gap widened (a Matthew effect). Our data also indicate "foveal load": momentary modulation of the perceptual span by the difficulty of ongoing cognitive processing. Such flexible and dynamic allocation of attention is further supported by experiments comparing silent and oral reading. Here we used the logographic Chinese writing system, which less directly codes for sound. Translation of text into speech should thus require more resources. Therefore, phonological preview benefit is usually absent in Chinese silent reading. However, when phonological processing was prioritized in oral reading, phonological preview benefit appeared. In a second experiment we show that it can occur at the sublexical level. Finally, our comics corpus results show that similar mechanisms regulate preview of upcoming panels.

Title: What Eye Movements Reveal about Orthographic and Semantic Word Learning

Authors: Jocelyn R. Folk[1], Michael A. Eskenazi[2], & Ashley N. Abraham[1]

[1]Kent State University, USA, [2]Stetson University, USA

Abstract

Vocabulary learning entails more than just acquiring a new word's meaning; it also includes learning a word's

spelling and pronunciation. In the current studies, we investigated individual differences in word learning and their relationship to eye movement patterns, focusing on the relationship between orthographic and semantic learning. We embedded novel words in informative or neutral sentence contexts while participants read silently for comprehension. We varied the number of exposures to the novel words (1, 3, or 6) while monitoring readers' eye movements. The reading sessions were followed by tests of orthographic and semantic learning. Across several experiments, we found evidence that readers were more successful at learning a novel word's meaning when they also learned its spelling. Importantly, this effect was strongest for higher-skilled readers. Word learning was reflected in eye movement patterns as initial reading times decreased on novel words across multiple exposures, and novel word skipping rates increased. Interestingly, word learning was not related to word skipping such that performance on a semantic vocabulary test did not change even if the novel word was never fixated. This was true for both higher- and lower-skill readers. This suggests that skipped novel words are processed enough to support word learning.

Title: Quantifying Dyslexic Performance in Classroom Copying Tasks

Authors: Julie A. Kirkby & Shannon Babb
Psychology Department, Bournemouth University, UK

Abstract
Classroom learning, the bedrock of school education, relies heavily on written information transfer. Seemingly simple typical classroom tasks of copying text are psychologically complex. They involve sequential visual and cognitive processes, including visual-encoding, constructing and maintaining mental representations, and written production. We have previously documented how children and adults cognitively process information during a copying task (Laishley, Liversedge, & Kirkby, 2015). Dyslexic children, in particular, struggle with visual-encoding, potentially causing difficulties with this type of task. In the current study we examined word-copying for dyslexic readers, assessing evidence for lexical or sub-lexical strategies. Dyslexic readers showed word-frequency effects, denoting lexical identification during visual-encoding. However, they required more visual-encoding time, and showed an exacerbated effect of word-length, suggesting a greater need to encode and maintain supplementary sub-lexical representations. We also found dyslexic readers had a higher probability of making a secondary encoding gaze-lift, than skilled readers, particularly for longer, lower-frequency words. We suggest this is due to insufficient activation of a robust lexical representation in long-term memory, indicative of poor lexical quality in dyslexia. The increased demands of word-copying for dyslexic readers should be accounted for in educational practice in order to minimise the potential for missed learning opportunities.

References

Laishley, A. E., Liversedge, S. P., & Kirkby, J. A. (2015). Lexical processing in children and adults during word copying. *Journal of Cognitive Psychology*, 27(5), 578-593.

Title: The relationship between efficiency and proficiency in reading: A cross-sectional eye-movement study

Author: Alexandra N. Spichtig, John D. Ferrara, Jeffrey P. Pascoe, & Elias Tousley
Reading Plus, USA

Abstract
This study compared four measures of comprehension-based silent reading efficiency (reading rate, fixations/100 words, regressions/100 words, and fixation duration) between proficient and non-proficient U.S. students in grades 2-12 (n=977). Students were divided into proficient and non-proficient readers by their state or local reading assessments. Data were obtained by educators using a low-cost eye-movement recording system as part of a national silent reading efficiency norm study (for details see Spichtig et al., 2016). The current study defined efficiency as reading < 150 words per minute (wpm).
Results revealed that reading efficiency played an increasingly important role as students advanced in grades. For example, while 78% of inefficient 2nd-grade students were able to achieve proficient reading levels on their local or state assessments, by grade 6 only 34% of inefficient students were able to do so, and by grade 12, 95% of students who achieved proficient levels of reading on their assessments read at or above 150 wpm.
In addition, it was found that by grade 6 inefficient readers appeared to stagnate, reading 124-130 wpm, making 166-176 fixations/100 words, 32-35 regressions/100 words, and 280-329 millisecond fixations, while their proficient peers were already reading 80 wpm faster, making 26% fewer fixations, 46% fewer regressions and 15% shorter fixations. By grade 12 proficient readers were reading 98 wpm faster, making 38% fewer fixations, 62% fewer regressions than their inefficient peers. Fixation duration differences were minimal.

References

Spichtig, A.N., et al., (2016). The Decline of Comprehension-Based Silent Reading Efficiency in the United States: A Comparison of Current Data with Performance in 1960. Reading Research Quarterly, 51(2), pp. 239-259.

Symposium 8
Thursday, August 22nd 10:30 – 12:30 Room 4

3-D Binocular Motor Control and Health

Authors: Kapoula, Zoi; Chaturvedi, Akshay; Daniel, Francois; Morize, Aurélien; Ward, Lindsey; Eggert, Thomas; Sprenger, Andreas
IRIS, Laboratory of Physiopathology of binocular motor control and vision, CNRS FR2022, University Paris Descartes, France; IRIS, Laboratory of Physiopathology of binocular motor control and vision, CNRS FR2022, University Paris Descartes, France; Ludwig-Maximilians Universität Dept. of Neurology, Germany; University Luebeck, Department of Neurology

Summary:
Purpose: Eye movements are an excellent tool for investigating brain function during development and adulthood. The use of eye movements in health domain is of particular interest. Nowadays, lots of instruments for video recording are available and lots of neuroscience has been built over decades on profound knowledge of neural basis of eye movements. In this context, the purpose of this symposium will be to present you some examples of these studies dealing with eye movement specificities in different populations in connection to cognitive, attentional and multisensory aspects.

Presentation 1: The first talk deals with the properties of eye movements in 3D space, comparing movements to visual versus audio visual targets. This reveals that lot of new insight on eye movement control can be obtained by considering multisensory interaction. The efficiency of vergence neuro-rehabilitation with such targets will be also presented.
Authors: Dr. Zoi Kapoula, Akshay Chaturvedi, Aurélien Morize

Presentation 2: Experimental induction of conflict between vergence and accommodation and measurement of impact of such conflict on reading and cognitive processes. This study clearly shows that cognition and attention are highly dependent on quality of binocular motor control.
Authors: François Daniel, Dr. Zoi Kapoula

Presentation 3: Properties of eye movement control to audio-visual targets in the 3D space for dyslexic adolescents. This presentation will report on multisensory integration via eye movement studies in dyslexia which is an entirely new field. The link with eye movement abnormalities during reading will be also discussed.
Authors: Dr. Zoi Kapoula, Lindsey Ward

Presentation 4: Factors affecting the repeatability of the alternating cover test. Phoria (loss of binocular eye alignment, when binocular vision is interrupted) is of major importance for the functional evaluation of binocular motor control and vision. The authors present an objective method based on eye movements measurements to evaluate phoria. Also, objectively demonstrate, high variability of phoria that is physiologic and not an artefact.
Author: Thomas Eggert

Presentation 5: Compensation of age related performance decrease in the visual and vestibular system. This presentation deals with an important health issue of ageing of the visual and vestibular systems and the compensations used by the CNS.
Author: Andreas Sprenger

Abstract 1: Most of the previous literature regarding eye movements study the effect of visual stimuli alone. However, in real life, visual and auditory stimuli occur together frequently. Auditory stimulus has been shown to facilitate saccades, but no such studies have been conducted to study its effect on vergence and combined eye movements. Therefore, the present study aims to investigate the effects of bimodal stimulation (auditory and visual) on saccades, vergences and combined movements. A total of 14 subjects, 6 women and 8 men (mean age: 22.6 +/- 0.62 years) were recruited for this study. Each type of eye movements was tested in two conditions: one without sound (visual target), another with a sound preceding the onset of the LED visual target (auditory-visual target). Eye movements were recorded with the Eyeseecam device. The results show substantial reduction in the saccade latencies when adding a sound before the visual stimulus. In contrast, for

convergence or divergence the sound did not decrease the latency but it did increase the velocity and reduced the duration of such movements significantly. For combined saccade vergence movements, particularly leftward, the duration of the saccade component decreased and the velocity increased by the sound as well as its latency decreased. These results support the hypothesis of complex saccade-vergence interactions during combined eye movements. The benefit from the sound for all types of eye movements is of theoretical and clinical interest. Its importance for clinical vergence rehabilitation methods will be discussed.

Abstract 2: Interaction mechanisms between cognition and binocular motor control in reading saccades remain unclear. In this study we examine objectively saccades and fixations parameters during the Stroop test, involving three different levels of cognitive demand (reading, color denomination and interference). In addition, we experimentally induce accommodation and vergence conflicts during the different tasks (1). Twenty-one visually normal subjects (age 20.9 ±1.45) performed the Stroop test in three different randomized conditions: a control normal viewing condition, a 16Δ base-out prism condition, and a -2.50D spherical lenses condition. Prisms and spherical lenses induced Vergence-Accommodation conflict. Eye movements were recorded with the Eyeseecam video-oculography device. The results show (1) longer fixation duration in the interference task than in the denomination task, and shorter fixation duration in the reading task; (2) a higher interference effect in the conflict induced conditions compared to the control condition; (3) a lower tolerance to prism induced conflict, with a higher destabilization of the binocular motor control of saccades and fixations. This suggests an interplay between vergence accommodation conflict and cognitive load: tolerance to the conflict seems to be lower in the more cognitively demanding interference Stroop task. The results consolidate the link between cognition and high quality of single binocular vision.

Abstract 3: It has been previously shown that dyslexic children have poor three-dimensional motor control during reading, namely in poor binocular coordination of saccades and fixation instability (7). This study is the first to measure how multisensory stimulation provokes abnormal eye movements in dyslexic teenagers. Using the REMOBI device, a table which provides a visual and auditory target stimulus in three-dimensional space, we measured vergence, saccades, and combined eye movements in 43 dyslexic adolescents aged 10 to 21 (mean age 15.28), in their school environment. Their eye movements were recorded using the Pupil Labs Eye Tracker. We found greater abnormalities in their vergence movements than in their saccades or combined movements. This study demonstrates that eye movement abnormalities are present in dyslexic children outside of reading, even when multisensory stimuli are used. These results are significant in that the visual deficits of the dyslexic adolescent cannot be overcome by providing a more robust stimuli including sound, suggesting their visual deficits are either extremely profound or that there are additional auditory deficits related to their pathology. Future research should focus on comparing multisensory stimuli vs visual-only stimuli in the same population; this will enable better understanding of eye movement abnormalities during reading

Abstract 4: Factors affecting the repeatability of the alternating cover test, **Thomas Eggert**
Using the alternating cover test in within-subject and within-examiner repeated measures designs, measures of heterophoria achieve standard deviations between 0.5 and 0.8 deg. Previous studies have shown that systematic differences between examiners and across time do normally not reach significance with respect to this noise level. However, it is less clear how this total noise is composed of variable errors related to the examiner (measurement noise), to the size of the manifest heterophoria (heterophoria noise), and to the availability of sensory vergence cues such as accommodative cues or residual binocular visual input (stimulus noise).
To address these questions, we developed an automated version of the alternating cover test (based on a system combining VOG and shutter glasses) which minimizes stimulus noise and has a well-defined measurement noise (sd=0.06 deg). In a within-subject design, 19 measures were taken within 1.5 min and multiple of such blocks were repeated either across days, or across 45 min at the same day. Blocks were separated by at least 7 min of binocular viewing. The standard deviation of the manifest heterophoria across blocks from different days or from the same day (sd=0.33 deg) was about 6 times larger than expected based on the standard deviation within the block (0.25 deg).
The results show that about half of the inter-day standard deviation of phoria measurements with the alternating cover test is related to a variability of the manifest heterophoria and not to measurement noise or stimulus noise. About 5/6 of the standard deviation of the heterophoria noise was induced by intermediate binocular viewing periods and did not affect the variability of the phoria observed during the 1.5 min of each alternating cover test.

Abstract 5: Compensation of age related performance decrease in the visual and vestibular system **Andreas Sprenger[1], Rebekka Lencer[2], Christoph Helmchen**

1 Department of Neurology, University of Luebeck/Germany

2 Department of Psychiatry and Psychotherapy, University Muenster/Germany

The process of getting older is mostly accompanied by performance decrease of the visual and vestibular system. Despite of this, humans in the age of 50+ are able to perform high level tasks with major use of visual and vestibular information. Although long-time experience may help to compensate for performance decrease in complex situations, i.e. on a macro level, there are several mechanisms that are used by the visual and vestibular system on the micro level: prediction and anticipation of oncoming events or movements.

To test this, we performed a study on smooth pursuit eye movements with and without target blanking in 45 participants (age 20-75 years). The pursuit target moved either predictable or not predictable in the horizontal plane with different timings of target blanking. Data shows preserved performance even with high age on anticipated movements or predicted movement after blanking. Initial acceleration and smooth pursuit gain in non-predictable situations decreased with age.

In a second study, we tested whether performance decrease in the vestibulo-ocular reflex can be compensated by predictive or active head movements. Eye and head movements were recorded in patients with bilateral vestibular failure (N=31, age 43-78 years) and age-matched healthy participants (N=31, age 44-80 years). Compared to non-predicitive (clinical default) conditions, data revealed increased VOR gain in conditions, which can be predicted by the subjects, even in healthy participants.

To conclude, anticipation and prediction may help to overcome age-related performance decrease in the visual and vestibular systems. These mechanisms are not used at will but subconsciously. Physiological decrease in oculo-motor performance cannot be compensated completely, but reduces deficits to a level that helps to survive demanding situations in real life. This helps to understand why – for example – a linear decrease in visual and vestibular performance is not highly correlated with car crashes.

References:

Daniel F, Kapoula Z. *Induced vergence-accommodation conflict reduces cognitive performance in the Stroop test*. Sci Rep. 2019 Feb 4;9(1):1247. doi: 10.1038/s41598-018-37778-y

Kapoula Z, Lang A, Vernet M, Locher P. *Eye movement instructions modulate motion illusion and body sway with Op Art*. Front Hum Neurosci. 2015 Mar 26;9:121. doi: 10.3389/fnhum.2015.00121. eCollection 2015

Lovegrove WJ, Bowling A, Badcock D, Blackwood M. *Specific reading disability: differences in contrast sensitivity as a function of spatial frequency*. Science. 1980 Oct;210(4468):439-40.

Cornelissen P, Richardson A, Mason A, Fowler S, Stein J. *Contrast sensitivity and c oherent motion detection measured at photopic luminance levels in dyslexics and controls*. Vision Res. 1995 May; 35(10):1483-94.

Bednarek DB, Grabowska A. *Luminance and chromatic contrast sensitivity in dyslexia: the magnocellular deficit hypothesis revisited*. Neuroreport. 2002 Dec 20; 13(18):2521-5.

Stein, John. (2012). *Visual Contributions to Reading Difficulties: The Magnocellular Theory. Visual Aspects of Dyslexia*. 10.1093/acprof:oso/9780199589814.003.0011.

Jainta S, Kapoula Z. *Dyslexic children are confronted with unstable binocular fixation while reading*. PLoS One. 2011 Apr 6;6(4):e18694. doi: 10.1371/journal.pone.0018694.

Daniel F, Morize A, Brémond-Gignac D, Kapoula Z. *Benefits from Vergence Rehabilitation: Evidence for Improvement of Reading Saccades and Fixations*. Front Integr Neurosci. 2016 Oct 20;10:33. eCollection 2016.

Symposium 9
Thursday, August 22nd 14:00 – 16:00 Room 4

Eyes Wide Shut: Gaze dynamics without vision

Symposium Abstract:

The human ability for visualization extends far beyond the physical items that surround us. We are able to dismiss the constant influx of photons hitting our retinas, and instead picture the layout of our kindergarten classroom, envision the gently swaying palm trees of our dream vacation, or foresee the face of a yet-to-be-born child. As we inspect imaginary objects and people with our mind's eye, our corporeal eyeballs latch onto the fantasy. Research has found that our eyes can move as if seeing, even when there is nothing to look at. Thus, gaze explorations in the absence of actual vision have been reported in many contexts, including in visualization and memory tasks, and perhaps even during REM sleep. This symposium will present the manifold aspects of gaze dynamics in conditions when the visual input is impoverished or altogether absent. Presentations will address the characteristics of large and small eye movements during imagined and remembered scenes, the impact of visual field deficits on oculomotor control, and the role of eye movements in the future development of neural prosthetics for the blind.

Organizer: Susana Martinez-Conde

State University of New York, Downstate Medical Center

Speakers and affiliations:

- **Bradley R. Buchsbaum** *(Rotman Research Institute, Canada)*
- **Susana Martinez-Conde** *(State University of New York, Downstate Medical Center, USA)*
- **Fatema F. Ghasia** *(Cleveland VA Medical Center, USA)*
- **Freek van Ede** *(University of Oxford, UK)*
- **Stephen L. Macknik** *(State University of New York, Downstate Medical Center, USA)*

Titles and abstracts:

Eye movement reinstatement and neural reactivation during mental imagery

Bradley R. Buchsbaum

Fifty years ago Donald Hebb theorized that mental imagery is a constructive process that reconstitutes the neural representations that normally accompany direct perception. Hebb argued that neural reactivation and mental imagery benefit from the re-enactment of eye movement patterns that first occurred at viewing (i.e., fixation reinstatement). To investigate these claims, we applied multivariate pattern analyses to functional MRI (fMRI) and eye tracking data collected while healthy participants repeatedly viewed and visualized complex images. The results showed that the specificity of neural reactivation correlated positively with vivid imagery and with detailed memory for the stimulus images. Moreover, the specificity of neural reactivation correlated positively with fixation reinstatement – even when analyses were constrained to fMRI signal from the occipital lobe. These findings support the conception of mental imagery as a simulation of perception, and provide evidence of the supportive role of eye-movement in neural reactivation.

Microsaccade generation requires a foveal anchor

Jorge Otero-Millan, Rachel E. Langston, Francisco Costela, Stephen L. Macknik, Susana Martinez-Conde

Visual scene characteristics have the ability to affect various aspects of saccade and microsaccade dynamics. For example, blank visual scenes are known to elicit diminished saccade and microsaccade production, compared to natural scenes. Similarly, microsaccades are less frequent in the dark. Yet, the extent to which foveal versus peripheral visual information contribute to microsaccade production remains unclear: because microsaccade directions are biased towards covert attention locations, it follows that peripheral visual stimulation could suffice to produce regular microsaccade dynamics, even without foveal stimulation being present. Here we determined the characteristics of microsaccades as a function of foveal and/or peripheral visual stimulation, while human subjects conducted four types of oculomotor tasks (fixation, free viewing, guided viewing and passive viewing). Foveal information was either available, or made unavailable, by the presentation of both solid and blurred simulated scotomas. We found foveal stimulation to be critical for microsaccade production, and peripheral stimulation, by itself, to be insufficient to yield normal microsaccades. In each oculomotor task, microsaccade production decreased when scotomas blocked foveal stimulation. Across comparable foveal stimulation conditions, the type of peripheral stimulation (static versus dynamic) moreover affected microsaccade production, with dynamic backgrounds resulting in lower microsaccadic rates than static backgrounds. These results indicate that a foveal visual anchor is necessary for normal

microsaccade generation. Whereas peripheral visual stimulation, on its own, does not suffice for normal microsaccade production, it can nevertheless modulate microsaccadic characteristics. These findings can have valuable implications for the diagnosis and treatment of ophthalmic conditions that degrade central vision, such as age-related macular degeneration.

Miniscule eye movements play a major role in amblyopia

Fatema Ghasia

Amblyopia, commonly called 'lazy eye' is a childhood disorder that if untreated early, will cause permanent visual impairment. Amblyopia is a neurodevelopmental disorder that occurs due to de-correlated binocular input to the visual cortex, but the eye structure itself is normal. It causes alteration in the organization and function of visual cortex. A variety of investigations have studied effects of amblyopia on sensory aspects of visual system with little known regarding its effects on visual efferent system. Macaque models of amblyopia have shown shifts in the ocular dominance, binocularity losses, impaired cortical acuity, and contrast sensitivity of the affected eye. Recent literature has reported extensively on the important functional role of microsaccades in visual perception. Microsaccades are essential for prevention of visual fading and effective visualization of complex scene. They are thought to provide an optimal sampling method for the brain while viewing finer objects, sharp edges, and contrast details. There is increasing evidence that the visual cortex signals microsaccade while the superior colliculus drives such input to generate microsaccades. Limited ability to separate small closely spaced visual objects, a phenomenon called crowding, is the hallmark of amblyopia. Studying microsaccades in amblyopia represents a unique opportunity to delineate their role in visual processing in disease states. Amblyopic patients have impaired fixational stability in both the amblyopic and the fellow eye. We have found a reduced frequency of microsaccades with increase disconjugacy and intrasaccadic drift in amblyopic patients during a simple fixation task. We found that amblyopic patients had difficulties performing visual search tasks during both fellow and amblyopic eye viewing. These difficulties were more pronounced in children with fusion maldevelopment nystagmus – a signature eye movement marker of disruption of binocularity in early infancy. In amblyopic patients without nystagmus, we found reduced microsaccade production during a visual search task, which could contribute to the crowding phenomenon. Thus, microsaccades can serve as an oculomotor disease biomarker for amblyopia assessment. Such investigations of FEMs before, during, and after treatment of amblyopia will be pivotal in understanding the role of microsaccades in visual processing, crowding phenomenon and efficacy of treatment.

Human gaze tracks the focusing of attention within the internal space of visual working memory

Freek van Ede, Sammi R Chekroud, Anna C Nobre

There is considerable overlap in the brain areas that control overt shifts of gaze and covert shifts of attention. In visual perception, these two functions are naturally linked because information sampled at covertly attended locations often informs where to look next. We ask whether the brain's oculomotor system also participates in attentional focusing when there is no incentive for overt shifts of gaze: when attention is voluntarily directed to one out of multiple visual items held internally within the spatial layout of visual working memory. Paradoxically, we showcase this participation through gaze behaviour itself. We demonstrate that selecting an item from visual working memory leads to an increased propensity of micro saccades in the direction of the memorised location of that item – even when there is nothing to look at and even when location memory is never asked about. Building on this key observation, we further show that this retrospective 'gaze bias' is specific to cases where the probed memory item is not already in the focus of attention, and predicts the performance benefit associated with such focusing (experiment 2); that externally-induced gaze shifts of similar magnitude are insufficient to place memory items into the focus of attention (experiment 3); and that this gaze bias generalises across the selection of different visual features (orientation and colour; experiment 4). We conclude that the oculomotor system also plays a key role in attentional focusing within the internal space of visual working memory, and that such 'internal focusing' can be studied through the eyes.

The role of eye movements in cortical prosthetics for the blind

Stephen L. Macknik, Olivya Caballero, Jordi Chanovas, Manuel Ledo, Daniel Cortes-Rastrollo, Azadeh Yazdan-Shahmorad, Shiming Tang, Nozomi Nishimura, Chris Schaffe, Yu-Ting Chen, Laurie Bizimana, Mitch Pender, John Reynolds, Michael Avery, Anirvan Nandy, Edward Callaway, Peichao Li, Eyal Seidemann, Yuzhi Chen, John Carpenter, Westley Hayes, David Heeger, Jose-Manuel Alonso, Susana Martinez-Conde

There are no current methods for generating optimized spatiotemporal cortical patterns that evoke naturalistic visual responses and perception. In addition, there are no cortical prosthetics that account for the effects of eye movements. Our prior research indicates that most spikes in V1 are driven by eye movements and that eye-movement-driven extraretinal inputs shape those neural responses. We present a novel prosthetic approach that integrates eye-movement-driven inputs to mimic naturalistic neural responses. We have transfected adeno-

associated viral (AAV) optogenes into macaque LGN using a recently described convection enhanced delivery procedure that enables large deep brain injections, using neurosurgical navigation techniques. Our approach is to optogenetically stimulate LGN neuronal boutons in layer 4 of V1 and identify the ON/OFF and ocular dominance preference of each LGN input at each retinotopic stimulation position. This will optimize targeting of the appropriate inputs and achieve maximal contrast at the highest attainable acuity, with stereoscopic fidelity. We will build a computational model of how LGN inputs spatiotemporally activate V1 when viewing a stimulus in a functioning visual system, and then optogenetically stimulate the LGN inputs to achieve the same perceptual response. To account for the effects of eye movements, we will use eye-tracking in conjunction with a novel model of oculomotor influence on early visual responses.

Oral Presentations
20th European Conference on Eye Movements

Session 1

Sunday, August 18th

Face Perception & Emotions (Room 4 | 16:15 – 18:15)

You literally cannot pay me to look at poop: The astounding lack of habituation to disgust as measured through oculomotor avoidance

PRESENTING AUTHOR
Edwin S. Dalmaijer
CO-AUTHORS
Alex Lee
Thomas Armstrong
AFFILIATIONS
MRC Cognition and Brain Sciences Unit, University of Cambridge, UK
Whitman College, WA USA
EMAIL
edwin.dalmaijer@mrc-cbu.cam.ac.uk

ABSTRACT
Disgust is a basic emotion that is typically triggered by polluting or offensive stimuli, and is often thought of as a disease-avoidance mechanism. Disgust is less susceptible to extintion or cognitive restructuring than other basic emotions like fear. This cognitive impenetrability can be a challenge for people who deal with disgusting material through their jobs, and presents a challenge for patients who suffer from disorders that disgust contributes to (e.g. OCD, specific phobias, and PTSD). Here, we investigate whether disgust might be reduced through the non-cognitive process of habituation. In each of three experiments, we presented participants with two images per trial, one disgusting (fecal matter) and one neutral (buttons or scarfs), while we collected gaze data. In the first experiment (N=104), participants looked at the disgusting image for less time compared to the neutral image, and this tendency remained over 28 trials that repeated the same images. In our second experiment (N=99), we replicated the non-habituating disgust avoidance from experiment 1, and showed that oculomotor differences between fearful and neutral images did habituate. In our final experiment (N=93), we encouraged participants to look at disgusting over neutral images by paying them $0.25 for every 4-8 seconds of disgust-gazing. Participants looked at the disgusting image while they obtained gaze-contingent rewards, and almost completely ignored the neutral image. While the manipulation did subtly reduce disgust ratings, participants returned to oculomotor avoidance directly after rewards were discontinued. We conclude that habituation to disgust did not occur, even after successfully encouraging prolonged exposure.

Recognition of dynamic emotions between deaf and hearing individuals

Izabela Krejtz
SWPS University of Social Sciences and Humanities, Warsaw, Poland
Krzysztof Krejtz
SWPS University of Social Sciences and Humanities, Warsaw, Poland
Katarzyna Wisiecka
Polish-Japanese Academy of Information Technology, Warsaw, Poland
Marta Abramczyk
Polish Association of the Deaf, Warsaw, Poland
Michał Olszanowski
SWPS University of Social Sciences and Humanities, Warsaw, Poland
Andrew T. Duchowski
School of Computing, Clemson University, Clemson, USA

Previous research showed either anger or happiness superiority effects in visual search paradigms (e.g., Savage et al., 2015). The present study examined dynamics of visual attention while deaf and hearing individuals decoded emotions from videos morphing from a neutral expression to either happy, sad or angry. The enhancement hypothesis (Sidera et al., 2017) posits that deaf people may have developed a greater visual sensitivity to facial expressions and therefore they may recognize facial emotions more effectively than hearing individuals.

By means of the K coefficient measuring ambient and focal visual attention (e.g., Krejtz et al., 2016), we explored strategies of gaze dynamics during emotion recognition. In line with prioritizing positivity effect, happy faces were more accurately and more quickly recognized than angry or sad faces. Both groups produced longer average fixation durations when decoding happiness in comparison to anger and sadness. Finally, before a decision about an emotion was made, deaf participants exhibited more focal viewing of happy faces than negative faces. Results support preferential decoding of happiness over anger and sadness from dynamically evolving stimuli.

Krejtz, K., Duchowski, A., Krejtz, I., Szarkowska, A., & Kopacz, A. (2016, May). Discerning Ambient/Focal Attention with Coefficient K. *ACM Transactions on Applied Perception*, 13 (3), 1–20. http://doi.org/10.1145/2896452
Savage, Becker, Lipp (2015). Visual search for emotional expressions: Effect of stimulus set on anger and happiness superiority. *Cognition & Emotion*. 30. 1-18. 10.1080/02699931.2015.1027663.
Sidera, F., Amadó, A., & Martínez, L. (2017). Influences on facial emotion recognition in deaf children. *Journal of Deaf Studies and Deaf Education*, 164-177. http://doi.org/10.1093/deafed/enw072

Eye Movement Study on the Own Age Effect of Children with Autism Spectrum Disorder in Face Processing

Ling Wei, Youyi Yu, Renhao Liu
Department of Applied Psychology, School of Humanities, Fujian Medical University

Many studies have found that Autism Spectrum Disorder (ASD) children have specificity in facial processing (Stoesz & Jakobson, 2018). However, not all studies reached the consistent conclusion possibly due to the age of the faces used in the experimental material. Typically Developing (TD) individuals process and recognize faces of their own age more accurately than faces of another age (the own age effect, Wright & Stroud, 2002). There is no study explored the effect directly on the ASD children.
Experiment 1 used 21 6-9 years old ASD children and 25 TD children as subjects. Their gazes on 24 pictures of neutral faces of the own age (children) and another age (youth) were recorded .The results showed that only the ASD children had significantly greater fixation duration and frequency towards the own age faces than another age faces. Experiment 2 explored the influence of facial emotion on the own age effect of the ASD children by using 48 faces of joy, anger and fear. The results showed that ASD children had significantly longer gaze duration at the faces of fear and anger of the own age, but not the joy; The first gaze latency and first fixation duration of the eyes and mouth showed the own age effect just in ASD children. The results of this study indicated that ASD children had the own age effect on the face processing and were influenced by the facial emotions. Overall, TD children did not show the own age effect of facial processing.

Key words: autism spectrum disorder, own age effect, face processing, emotion.

Eye movements to tilted faces reveal a strong upper eye bias

Davidenko, Nicolas
University of California, Santa Cruz

When we look at a face, we tend to automatically fixate on features like the eyes and mouth that provide the most useful information for the task at hand (e.g. identity recognition, expression judgments, speech perception, etc.). In addition, most of us are susceptible to a left gaze bias, wherein the left side of a face (from the perspective of the observer) elicits more attention and fixations than the right side. However, it is unknown whether tilted faces also elicit a left gaze bias, or whether the vertical displacement of the two eyes in tilted faces leads to a different type of bias. In a series of three eye-tracking studies, participants judged the expressions of faces presented upright or tilted in-plane by +/- 45 degrees (in studies 1 and 3) and broader range of angles (in study 2). Eye movements were monitored using a 60-Hz GazePoint eye tracker, and first and second fixations to each face were analyzed by independent coders. Across the three studies, participants exhibited a strong bias to fixate the upper eye in tilted faces. This "upper eye bias" was strongest when faces were tilted by about +/- 45 degrees, and it quickly outweighed the left gaze bias with tilts as small 11.25 degrees. I will discuss potential neural mechanisms of the upper eye bias in tilted faces and consider some implications for research in social cognition.

Wild lab - Characterizing face-selective ERPs under more natural conditions

Gert, Anna Lisa ; Ehinger, Benedikt V.; Timm, Silja; König, Peter *; Kietzmann, Tim C. *

Institute of Cognitive Science, Osnabrück University; Donders Institute for Brain, Cognition and Behaviour, Radboud University; Institute of Cognitive Science, Osnabrück University; Institute of Cognitive Science, Osnabrück University; MRC Cognition and Brain Sciences Unit, Cambridge University

Over more than two decades, neural mechanisms of face processing have been studied in the lab using highly constrained experimental conditions. Typical paradigms include passive fixation tasks, randomized image sequences, and adjusted low-level stimulus features. These controls help overcoming technical challenges in subsequent analyses, but at the same time yield paradigms that strongly contrast natural vision. Do known properties of face selective ERPs generalize to this situation?

Here we overcome these limitations by combining a set of novel analysis techniques. We record visually responsive ERPs in an unrestricted viewing paradigm while participants freely explore natural scenes. We use non-linear deconvolution in a mass-univariate analysis framework to separate neural responses to previous and current fixations, as well as other confounding variables (like saccade amplitude or the fixation position). Focusing on fixation-locked ERPs in a 2x2 design (previous/current fixation, face/background) we find a main effect of current fixation, reproducing the classic N170. Furthermore, our analyses reveal that fixation responses are strongly modulated by fixation history, in particular by the presence of a previously fixated face. To conclude, we here use a combination of novel analysis techniques to embed the classical N170 paradigm in an ecological more valid experimental setup. Replicating and extending classic lab-based findings, this project paves the way for future experimental investigating using more natural, less constrained paradigms.

Manifestation of the other race effect during spectatorship of paintings

Trawinski, T.[1*], Zang C.[2], Liversedge, S. P. [3], & Donnelly, N. [4]

1 Department of Psychology, University of Southampton, Southampton, United Kingdom
2 Academy of Psychology and Behavior, Tianjin Normal University, Tianjin, China
3 School of Psychology, University of Central Lancashire, Preston, United Kingdom
4 Department of Psychology, Liverpool Hope University, Liverpool, United Kingdom

British and Chinese participants classified as naive beholders of art were asked to encode images of paintings for recall in a test phase. The test phase occurred 30 minutes after encoding and participants discriminated half of the images shown at encoding from foils. The set of paintings shown to participants consisted of the 100 Western or East Asian paintings. Each painting was split into face (area covered by face(s)) and context (the rest of the paintings excluding face(s)) regions of interest. Behavioral responses at test and eye movements at encoding and test phases were analyzed. Ethnicity influenced both overall performance and eye movements. Behavioural measures showed Chinese participants to be less accurate than British participants but only with respect to Western paintings. With respect to eye movements, British participants made greater and longer fixations to faces in Western paintings than Chinese participants at encoding. In contrast Chinese participants made greater and longer fixations to faces in East Asian paintings than did British participants. The implications of ethnicity on the viewing of faces in paintings is considered.

Clinical Research (Room 5 | 16:15 – 18:15)

Affective features of psychopathy predict reduced eye contact in incarcerated offenders: A study of eye movements during live social interaction

Nina A. Gehrer[1], Andrew T. Duchowski[2], Aiste Jusyte[3], Michael Schönenberg[1]

1 *Department of Clinical Psychology and Psychotherapy, University of Tübingen*

2 *Visual Computing, Clemson University*

3 *Department of Psychiatry and Psychotherapy, University Hospital Tübingen*

The eyes and gaze of an interaction partner convey crucial nonverbal information about the individual's internal state. For example, deficient eye contact during childhood is assumed to have detrimental effects on the development of social cognition including empathy and may contribute to the development of psychopathy. Indeed, previous studies have linked psychopathic personality traits to impaired attention orienting to the eyes while categorizing facial stimuli. However, to date, no study has examined the ecological validity of these findings and it is still unclear which facet of psychopathy is driving this effect, i.e., interpersonal, affective, lifestyle or antisocial features. In the present study, we assessed eye contact in a sample of incarcerated offenders (N=30) during live face-to-face interaction via a mobile eye tracking headset. Psychopathic traits were measured by means of Hare's Psychopathy Checklist-Revised. For the analysis of eye movements, we developed a method to automate video frame Areas of Interest labelling (e.g., eye region). We employed linear mixed effect models and, while controlling for the effects of age and activity (listening vs. talking), higher affective psychopathy was found to predict reduced eye contact. No other facet of psychopathy was linked to the gaze patterns. Thus, the present study provides evidence linking impairments in attention orienting to the eyes to a lack of empathy, an incapacity of feeling guilt or remorse, and shallow affect (i.e., affective psychopathy) in incarcerated offenders. Further, we conclude that these deficits extend to naturalistic settings such as live social interaction and therefore, have behavioral relevance.

Effect of visual tracking intervention on attention and behavior of Attention Deficit Hyper Activity children

Ebrahim, Pishyareh ; Shiva, Janmohamadi; Haghgoo, Hojjat Allah

University of Social Welfare and Rehabilitation Sciences, Tehran, Iran.; University of Social Welfare and Rehabilitation Sciences, Tehran, Iran.; University of Social Welfare and Rehabilitation Sciences, Tehran, Iran.

Abstract

Attention deficit - hyperactivity disorder is characterized by several behavioral and cognitive problems such as inattention and impulsivity, abnormal control of saccadic eye movements and visual fixation, smooth pursuit movements, disturbed saccadic eye movements and eye movement relocation, and problems in inhibition and control on eye movements. Hence, the effects of eye tracking intervention was studied on improvement in sustain attention in these children.

Method

Thirty-nine boys, 6 to 10 years, matched in terms of intelligence, recruited and randomized to receive conventional occupational therapy referred as control group, or occupational therapy and eye tracking exercises, referred as experimental group. They evaluated using Conner's rating scale, Continuous performance task-2, and Test of Visual- Motor Skills- Revised before and after intervention.

Result

Significant improvements in the mean scores of cognitive problems (F=9/22), coping behavior (F=6.03) and hyperactivity (F=9.77) detected in the post-test between the two groups ($p<0.05$). furthermore, in the Continuous Performance Test scores, detectability (F=5.68), omission error (F=17.89), commission errors (F=19.45), reaction time (F=8.95), variability (F=7.07), and preservation (F=6.33) showed significant differences between control and experimental groups ($p<0.01$).

Conclusion

Intervention of pattern based eye movement tracking is able to improve cognitive functions and coping behaviors in these children.

Eye Tracking and reading disabilities in a sample of Portuguese children

Norberto Cardoso Pereira[1,2,4], Maria Armanda Martins da Costa[2], Manuela Guerreiro[3]
[1]Faculty of Letters, Lisbon – CLUL | [2]NeuroCog | [3]Faculty of Medicine, Lisbon – IMM | [4]Faculty of Sciences – IBEB

Abstract
This study investigated the neuropsycholinguistic profiles of typical and atypical reading children with the aim of finding which cognitive, linguistic and eye tracking measures have the most predictive diagnostic value to develop dyslexia or ADHD, and which of them are more suitable to explain measures of reading fluency. The sample is composed of 59 Portuguese children, 9 years old, native speakers of European Portuguese (L1) attending the 4th grade of primary education. The sample was divided in three groups, a group of 21 ADHD children, 19 children with dyslexia and 19 control children. Procedure: eye movements were recorded with the SMI IVIEW X™ HI-SPEED eye tracking system (SensoMotoric Instruments) while performing a reading task consisting of a descriptive-informative text with 264 words, with control of lexical properties, namely word-frequency and word-length. Multinomial logistic regression analyses suggested that some measures revealed an adequate sensitivity for the early diagnosis of dyslexia and ADHD. Having the control group as a reference, the strongest variables to predict the development of dyslexia were: verbal working memory, phonological awareness and rate of fixations of long low-frequency words. As far as ADHD is concerned, the risk factors include deficits in: visuospatial working memory, lexical memory, processing speed, visual attention and oculomotor coordination. Moreover, linear regression models enabled to conclude that the better predictors of reading speed are "diagnosis" and "second pass reading time" of short .

Keywords: predictors of reading disabilities; eye tracking; reading fluency

Oculomotor Measures as Endophenotypes for Attention Deficit Hyperactivity Disorder and Autism Spectrum Disorder.

Seernani D.[1], Damania K.[2], Ioannou C.[1], Hill H.[3], Anderson N.[6], Boccignone G.[4], Foulsham T.[5], Bishof, W.[6], Kingstone, A. [6], Biscaldi M.[1], Ebner-Priemer U.[3], Klein C.1,[7].

1. *Department of Child and Adolescent Psychiatry, University of Freiburg.*
2. *Amazon.com*
3. *Institute of Sports and Sports Sciences, Karlsruhe Institute of Technology.*
4. *Department of Computer Science, University of Milan.*
5. *Department of Psychology, University of Essex.*
6. *Brain, Attention and Reality Lab, University of British Columbia*
7. *Department of Child and Adolescent Psychiatry, Medical Faculty, University of Cologne.*

Background: Endophenotypes are intermediate variables in the hypothetical causal chain from observed behavior of a clinical disorder to its underlying genotype. Recent literature trends point to the potential etiological overlap between Attention Deficit Hyperactivity Disorder (ADHD) and Autism Spectrum Disorder (ASD). The present study aims to systematically compare these groups, by studying the potential endophenotypes of ASD (local-global processing; social cueing) and ADHD (Intra-Subject Variability (ISV)) in tandem.

Methods: Three tasks, namely visual search, copying down and gaze cueing were administered to directly examine ASD- (ASD without co-morbid ADHD), ADHD and ASD+ (ASD with co-morbid ADHD) groups, in comparison to a typically developing (TD) group (N=100). Step-by-step process analysis and scan-path models were employed to analyze the oculomotor and behavioural data collected.

Results: Results from the visual search task show that groups with ADHD symptoms (ADHD and ASD+) have increased intra-subject variability, whereas only the ASD- group showed signs of superior performance. Fixation durations during the copying down task can differentiate ASD- and ADHD groups on the strategies used. The gaze cueing task, shows the ASD+ group to use different strategies as compared to TD, and have slower and more variable saccadic RTs as compared to ADHD and ASD-.

Conclusion: The present study gives evidence for a double dissociation between ADHD and ASD when no comorbid symptoms are present, on paradigms of local-global processing and social cueing. Oculomotor paradigms and analysis have successfully teased apart this interaction in the present study and can aid greatly in the quest for these endophenotypes.

The effect of levodopa on saccades–Oxford Quantification in Parkinsonism study

Chrystalina Antoniades, Zhongjiao Lu, Tim Buchanan, James FitzGerald

Objectives

The evaluation of novel disease modifying drugs requires biomarkers that are simultaneously sensitive to disease state but resistant to the effects of background symptomatic treatment. Saccadic eye movement parameters have been proposed as a neurophysiological biomarker for Parkinson's disease (PD) and so it is important to know how they are affected by dopaminergic medication. Studies to date are conflicting: some have concluded that medication prolongs saccadic latencies while others suggest they are shortened. We aimed to characterise the effects of antiparkinsonian medication on prosaccadic and antisaccadic parameters in a large cohort of PD patients and age matched healthy controls.

Methods

We studied saccades both off and on medication in 38 PD patients and 34 controls. Latencies, amplitudes, velocities, and directional errors were evaluated, using a published standardised protocol. We then combined this study and previously published literature in a meta-analysis of the effects of antiparkinsonian medication on prosaccadic latency.

Results

Prosaccadic latency is significantly prolonged by dopaminergic medication in PD. The effect size is comparable to the difference between PD off dopaminergic therapy and healthy control values. There was no statistically significant change in any other saccadic parameter with medication. Of particular note, antisaccadic latency was almost exactly the same on and off medication, while being almost 20% longer in PD patients compared to healthy controls.

Conclusion

Many saccadic parameters appear to be resistant to the effects of dopaminergic medication. Antisaccadic latency is particularly interesting because it shows a large disease effect with no medication effect.

The pupil accommodative response as a communication tool in ALS patients.

Roatta S., Lorenzo Villalobos A. E., Giusiano S., Musso L., Calvo A, Moglia C., de'Sperati C.

The "voluntary" control of pupillary constriction during accommodative tasks (PAR) has been recently tested in healthy subjects and proposed as a communication tool for paralyzed patients (Ponzio et al, 2019). Whether pupil functionality and PAR is preserved in a progressively paralyzing disease such as amyotrophic lateral sclerosis (ALS) is not known and is here addressed with a longitudinal and a cross sectional study.
The pupil light reflex and PAR have been investigated in a group of 19 ALS patients, consecutively enrolled at the time of diagnosis and followed for 15 months, and in 10 ALS patients, in an advanced stage of the disease (all artificially ventilated). The PAR was tested by asking the patient to shift the gaze from a far (>3m) to a near (40 cm) visual target. In 4 patients of the latter group, a communication protocol was tested, requiring a PAR as input to answer "YES/NO" to a series of 10 questions with known answers. Pupil size was bilaterally monitored (EyeTribe).
Pupil functionality appeared to be stable in the follow-up and still present in (9 out of 10) advanced stage patients (constriction magnitude: PAR: 19 ± 8 %; light reflex: 40 ±8 %). The accommodative task was easily understood and could effectively be used to answer the posed questions (accuracy 87.5 %).
For the first time, the PAR is shown to be well preserved in advanced ALS patients and suitable to be employed as a binary communication, without requiring any previous learning or training session.

Applications: Education (Room 10 | 16:15 – 18:15)

Applications of Gaze Analyses in the Study of Pedagogical Expertise in Music

Robert A. Duke, PhD*, Center for Music Learning, The University of Texas at Austin*
Laura K. Hicken, PhD*, Department of Music, Towson University*
Travis Marcum, PhD*, Austin Classical Guitar*

Data from various applications of eye-tracking have revealed important information about human attention in perceptual and motor tasks that involve explicit or implicit decision making. Available eye-tracking technologies have yet to be deployed in analyzing the allocation of attention in expert music pedagogy. We developed an analysis procedure that facilitates the identification of teachers' ongoing selection and prioritization of behavioral goals, and thus defines the performance outcomes that guide teachers' moment-to-moment behavior during the course of instructional interchanges.

We report on 4 experiments that reveal aspects of visual attention in expert and novice music teaching. In Experiment 1, we analyzed the gaze of string instrument teachers in the process of teaching individual learners. To determine the effects of an auditory signal on teachers' allocation of attention (Experiment 2), we compared the gaze of expert string teachers observing a solo violinist with and without hearing the sound of the performer's instrument. In Experiment 3, we observed the gaze of string teachers at different stages of experience and expertise, and noted attentional foci in relation to goal-setting, instructional directives, and feedback. In Experiment 4, we analyzed the gaze of performing musicians and teachers as they viewed multiple video recordings of individuals performing music (including the observers' principal instruments) and three other motor tasks (juggling, dancing, and batting in baseball). Our results reveal aspects of attention allocation that inform pedagogical decision making and facilitate the prioritization and accomplishment of proximal instructional goals.

Interactive multimedia keep students focused. Impact of interactive educational tools on dynamics of attention and learning effectiveness.

Krejtz, Krzysztof ; Borkowska, Olga; Duchowski, Andrew
SWPS University of Social Sciences and Humanities, Warsaw, Poland; SWPS University of Social Sciences and Humanities, Warsaw, Poland; School of Computing, Clemson University, SC, USA

Abstract:
Interactive multimedia learning materials present knowledge in verbal and visual representations allowing students to manipulate and play with the content. Advances in technologies make such tools available for massive use during formal and informal education settings. There are also growing expectations among education practitioners towards such tools. To our best knowledge, however, the impact of interactive multimedia tools on learning outcomes and its attentional mechanism are still not well understood. For example, Krejtz et al. (2016) showed that the interactive simulation elicited more careful visual investigation of the learning material but no learning effects were reported. Others argue that multimedia materials split attention, increase cognitive load, and reduce transfer learning (Austin, 2009).
In the present study we used eye tracking to examine how interactive materials affect the attention allocation and ambient-focal dynamics during learning and how they are related to short and long learning effects. The participants' (N=27) task was to learn two science problems of biology and mathematics using interactive or static multimedia learning materials. Two knowledge tests were performed measuring short and long term learning outcomes. Working memory capacity was controlled. The results revealed more focal visual attention and stronger focus on textual descriptions while using interactive tools. These effects were strongly related to better learning effectiveness. We provide the evidence for effectiveness of interactive multimedia in learning and more importantly shed a light on attentional mechanism related to such effects. The results and their interpretation contribute to Cognitive Theory of Multimedia Learning (Mayer, 2002).

Investigating the relationships between teachers' gaze, classroom practices and teacher self-efficacy belief

Skuballa, Irene ; McIntyre, Nora; Barza, Lydia; Von Suchodoletz, Antje
Open University of the Netherlands; University of Sheffield; Zayed University; New York University Abu Dhabi

The present study investigates the associations between teachers' gaze behaviors in real-time teaching situations, their teaching quality, and self-efficacy beliefs, as suggested by a preliminary framework on professional vision in teaching (Lachner, Jarodzka, & Nückles, 2016). Participants were 56 kindergarten teachers from the UAE. Teaching quality was assessed by three domains from a standardized observation manual: emotional support, classroom organization, and instructional support (CLASS; Pianta, La Paro, & Hamre, 2008). Teacher self-efficacy belief was measured through a self-report scale (Tschannen-Moran & Hoy, 2001). Teachers' gaze was recorded by mobile eye-tracking glasses (SMI, 60 Hz). Gaze events were defined as visual intakes between two saccades. For analyses, we used a semantic coding scheme where we determined children as AOIs. Two indicators were of relevance: duration of visual intake per visit in total, which represented general information processing of the classroom (i.e., teacher visually taking in classroom information), and duration of visual intake per visit on a child as an indicator of communicative gaze (e.g., teacher-student eye contact while teacher gives information). Multivariate regression analyses demonstrated self-efficacy to be a significant predictor of information-processing gaze but not communicative gaze. Next, multivariate multiple regression analysis found information processing gaze to consistently predict teaching quality variables, whereas, communicative gaze only near-significantly predicted teaching quality. While communicative gaze seemed weak in its association with teacher self-efficacy and teacher effectiveness, both gaze types in combination were required for a significant mediation of teacher self-efficacy's prediction of teaching quality.

Towards a software prototype for the identification of the eye movements associated with dyslexia through eyetracking

Meza-Garcia, Eduardo Ariel ; López-Orozco, Francisco; López-Nájera, Abraham; Quiñonez-Domínguez, César; Loya-Sánchez, Carlos Esteban; Alarcón-Osollo, Adrián

Universidad Autónoma de Ciudad Juárez; Universidad Autónoma de Ciudad Juárez; Universidad Autónoma de Ciudad Juárez; Universidad Autónoma de Ciudad Juárez; Universidad Autónoma de Ciudad Juárez; Universidad Autónoma de Chihuahua

This research is presented in three folds. First, a review of state of art of eye movement patterns associated to dyslexia was done and in a second stage a develop of a software prototype for the identification of eye movements patterns with dyslexia through eye-tracking technology is done. Finally, in order to test and validate our prototype, an eye-tracking experiment was conducted with 104 participants. Stimuli texts were presented during the experiments with different configurations changing some parameters as text content, font type and size, text and background colors, line spacing, column width and others (Rello et al., 2014; Tseng, 2012; Dickinson et al., 2012). During this testing phase, promissory results were obtained. 19 participants (18%) presented some tendency that could be representative of dyslexia. While those who presented several trends were 4 (3% of the total sample). Both percentages are in accordance with the percentage of people with dyslexia in Mexico (between 3% and 10%) (El Universal Diario, 2010). This allows us to see that the sample taken meets characteristics consistent with those of the country's population. However, the study should be continue in order to generalize our results toward the construction of a complete software system for dyslexia detecting.

Tracing attentional anchors: Idiosyncratic repetitive eye-movements in the control of prospectively mathematical sensory-motor routines

Anna Shvarts[1], Dor Abrahamson[2], Arthur Bakker[1]
[1]Utrecht University, The Netherlands; [2]University of California, Berkeley, USA
a.y.shvarts@uu.nl

Whereas the idea of scanpaths as idiosyncratic repetitive patterns for visual analysis of stimuli was introduced decades ago, the cognitive role of these patterns is still under consideration. Of note, prior investigations were concerned mostly with static images of everyday objects. In this presentation, we draw attention to the emergence of idiosyncratic repetitive patterns of eye-movements in a variety of dynamical embodied educational designs. These tasks comprise motor-control problems, where students are required to move objects on a touchscreen in an attempt to discover a rule determining a regimen of color feedback from the technological system. We report on the iterative eye-movements that were found in designs for proportion, parabolas, and trigonometry. In all these cases, the students appear to trace isomorphic points on the manipulated objects, however, the particular eye-movement patterns varied from subject to subject. Interestingly, the fixation points did not bear any information that would require foveal analysis. Rather, they reflected attentional anchors, namely perceptual patterns that emerge as an efficient means of enacting target movements (Duijzer, Shayan, Bakker, Van der Schaaf, & Abrahamson, 2017). Based on qualitative analysis of students' multimodal utterances, we track how the invoked attentional anchors are later involved in explicit discourse about formal mathematical content.

Duijzer, C. A. C. G., Shayan, S., Bakker, A., Van der Schaaf, M. F., & Abrahamson, D. (2017). Touchscreen Tablets: Coordinating Action and Perception for Mathematical Cognition. *Frontiers in Psychology*, 8, 144.

A methodological synthesis of eye-tracking research in second language acquisition and bilingualism

Godfroid, Aline
Michigan State University

Eye-tracking research has taken hold in Second Language Acquisition (SLA) and bilingualism. The number of eye-tracking publications with non-native speakers and bilinguals has increased exponentially since the early 2000s, and there are now several discipline-specific reference works (Conklin, Péllicer-Sánchez, & Carrol, 2018; Godfroid, 2019; Godfroid, Winke, & Gass, 2013). In this talk, I present the findings of a synthetic review of the L2 eye-tracking literature, focusing on both substantive and methodological features.
An online search of academic databases and 16 SLA and bilingualism journals resulted in the identification of 52 text-based studies and 32 visual studies. Across the two modalities, studies on grammar representation and processing constituted the largest research strand (k = 19), followed by work on lexical processing and acquisition (k = 17), and the real-time effects of language instruction (k = 8). In the visual world paradigm, prediction research accounted for over half of all published studies (k = 17). The survey also revealed innovative uses of eye tracking to study multimodal processing (k = 4), language assessment (k = 4), and oral interaction and spoken language production (k = 5). Reading researchers analyze an average of 3.3 measures per study, but their analyses are still heavily focused on durational measures.
I argue that eye-movement registration with L2 learners or bilingual participants is coming of age. While building on a strong research tradition in psychology, L2 eye-tracking researchers are also actively adapting and innovating their methods to rise to the challenges that come with a new discipline.

Conklin, K., Pellicer-Sánchez, A., & Carrol, G. (2018). *Eye-tracking. A guide for applied linguistics research.* Cambridge: Cambridge University Press.
Godfroid, A. (in press, 2019). *Eye tracking in second language acquisition and bilingualism: A research synthesis and methodological guide*. New York: Routledge.
Godfroid, A., Winke, P., & Gass, S. (2013). (Eds.). Eye-movement recordings in second language research [Special issue]. *Studies in Second Language Acquisition*, 35(2), 205–212.

Session 2

Monday, August 19th

Reading Models I (Plenary room | 10:30 – 12:30)

Integrative Mechanisms in Sight-Reading and Silent Reading

Michel Cara, Esteban Yima, Álvaro Hurtado
Pontificia Universidad Católica de Valparaíso, Chile

Introduction
The relation between sight-reading and reading texts was studied in a group of university students. Through the study of sight-reading tasks, we attempt to search for differences and similarities in the integrative mechanisms underlying music reading and verbal text reading. We are searching for new insights about music learning in adults.
Questions
Are musicians who are more successful in terms of reading scores equally effective in reading verbal texts? Is the sight-reading task relevant to study of the relationship between music and language at the level of information integration processes?
Design
In the **first experiment**, different music reading tasks were applied as well as a verbal comprehension task. In the **second experiment** a sight-singing task and a verbal comprehension task were applied to participants. The students (n = 17, first experiment and n = 15, second experiment) were musicians with at least 3 years of music reading experience (formal training), all students at Pontificia Universidad Católica de Valparaíso. In the second experiment, eye-tracking data were collected.
Results
In the first experiment, we found negative correlations between the music reading efficacy (numbers of errors) and the score on the verbal comprehension test ($r = -.55$, $p = .043$) while other tasks were not correlated with the verbal comprehension task.
In the second experiment, we found links between integrative mechanisms in music reading and verbal text reading. A Principal Components Analysis led us to study the relationship between the different variables (e.g., regressive fixations at local and global level, music flow)

Navigating the Narrative: Readers' Strategies When Reading Comic Page Layouts

Kirtley, Clare ; Murray, Christopher; Vaughan, Phillip; Tatler, Benjamin
University of Aberdeen; University of Dundee; University of Dundee; University of Aberdeen

The order of viewing when presented with images is much less strict compared to the order of viewing when reading text. However, in stimuli such as comics, the reader must follow a narrative in which both text and image contribute to the information. While comic layouts typically follow the reading order of the culture in which they are written, artists may use more irregular, unique layouts, which the reader must still follow correctly to understand the story presented. Previous work on comic reading has found that a large contributor to navigation decisions is the external structure, or outlines, of the panels with little contribution from the panel content. However, other studies have shown that panel content (particularly text) can influence reading order. The present studies aimed to investigate this contribution further, using eye-tracking to examine readers' online navigatory decisions. In Experiment 1, participants were presented with comics in which the layout was manipulated into one of six types. The influence of the external structure was replicated, but an effect of text location was also found for one layout type. Experiment 2 focused on variations of this particular layout, manipulating the location of text within critical panels. It was found that panel content was a consistent effect for all variations, suggesting that, while most navigation decisions are the result of using the external structure, content is a key aspect for readers when resolving ambiguous layouts, and should therefore be considered as part of the navigation strategy.

Shorter fixations before regressions? Evidence for two qualitatively different types of regressive eye movements during reading

Weiss, Anna Fiona
Philipps-University of Marburg

Although both increased fixation durations and a higher number of regressions have been assumed to reflect processing difficulties during reading, there is also evidence that these two measures do not just ‘sum up’ each other, but have to be functionally distinguished. For example, it has been shown that fixations preceding regressions tend to be shorter than fixations preceding progressive saccades (Altmann et al., 1992). A model that may explain these on the first glance counterintuitive findings is the Information Gathering Framework (IGF; Weiss 2017). However, the IGF also assumes that there exist two different types of regressive eye movements: (A) Regressions due to integration difficulties which may occur all over the sentence and (B) regressions due to missing evidence which occur mostly at the end of a sentence. More importantly, the IGF predicts that shorter fixations durations should only occur before regressions of type A but not before regressions of type B. In order to test this hypothesis, we reanalyzed data from a large-scale eye tracking study of 92 English subjects reading 99 English sentences of varying syntactic structure (Weiss et al., 2017). We identified all saccades and compared the preceding fixation durations by using linear mixed-effects models. Our results show significantly shorter fixations before regressions than before progressions in all sentence regions ($t > |2|$), with exception of the last region ($t=1.30$). These findings are fully in accordance with the predictions of the IGF and provide more evidence that two functionally different types of regressions may exist.

Integrating syntactic expectations with parafoveal visual information during reading

Sturt, Patrick ; Cutter, Michael; Martin, Andrea
University of Edinburgh; University of Edinburgh; Max Planck Institute for Psycholinguistics, Nijmegen, The Netherlands

It is known that fixation times on a word N can be affected by visual features of the next word, N+1. However, there is currently little experimental evidence that such parafoveal-on-foveal effects can also be affected by higher-level linguistic properties of word N+1.
In three eye-tracking experiments, we presented participants with sentences containing either subject or object relative clauses, in which the overt argument inside the relative clause was a capitalized proper noun (e.g., "The tall lanky guard who [alerted Charlie/Charlie alerted] to the danger was young"). We hypothesized that we would observe early relative clause effects (i.e., inflated fixation times for object vs. subject relative clauses) while readers were still fixated on the pre-disambiguating words (i.e. "guard who"), given that capitalization of the next word ("Charlie") is a salient visual cue for its syntactic class, and thus the structure of the sentence. This prediction was confirmed in Experiments 1 and 3, and in a Bayesian analysis combining all three experiments. Further manipulations (involving fully upper-cased control conditions in Experiment 1, and non-valid preview conditions in Experiment 2), suggested that these effects are likely to be due to noun capitalization rather than lexical processing. Thus, we suggest that participants detected that they were processing an object relative clause on the basis of visual features of a parafoveal word, leading to increased difficulty of syntactic integration, observable as a parafoveal-on-foveal effect.
We discuss our findings in relation to theories of sentence processing and models of eye movement control during reading.

A tale about differences in eye movements while children and adults read comics and texts using a coherence paradigm.

Martin-Arnal, L. A., León, J. A., Olmos, R.
Universidad Autónoma de Madrid

The aim of this study dives into the relation between age, coherence and format, exploring differences in eye movements while children/adults read text/comic stories. Many processes are involved in reading comprehension, allowing us to elaborate a complete mental representation (León & Escudero, 2017) to maintain the coherence of stories. These processes influence eye movements when something does not compute (e. g., Clifton, Staub & Rayner, 2007). In the last decade, several experiments aimed to describe cognitive processing through eye tracking (e.g. König et al., 2016) and a small number (e.g., Leinenger & Rayner, 2017) found differences in reading comprehension patterns between sentences/images and children/adults. 63 participants volunteered for the experiment: 32 adults (18-24) and 31 children (10-12), who read up to 20 short texts and up to 20 comics and chose between both endings (coherent VS incoherent) on each. An eye-tracker Tobii-x120 to register data and a multilevel analysis were used. Results showed more fixations for children than for adults, being this difference higher on texts; fixations were longer on comics, being the difference higher for children. Besides, there were more and longer fixations and more “regressions out” from coherent stimuli, more “regressions between” than “out” on comics and vice-versa on texts, and adults made fewer regressions than children on comics; besides, children made more “regressions between”. Consequently, both, children and adults, reached the same level of comprehension, supporting on different strategies: while children needed to use an additional comparative strategy, facilitated by images, adults only needed a confirmatory approach.

Does substituting words by emoji affect reading? Evidence from word recognition, sentence comprehension, and text reading

Nik Kharlamov, Alexander F. Povlsen and Kasper H. Ravnkilde
Aalborg University

Emoji are graphic symbols that represent emotions and objects and are often used in textual communication, such as instant messaging. While using emoji at the end of sentences as emotional accents is not new, smartphone software has recently incorporated the possibility to substitute individual words in sentences with emoji, providing suggestions for the user to do so. Such substitution creates sentences that mix words and symbols in traditionally alphabetic languages such as English and Danish, thus introducing a logographic component.

We present evidence from three experiments using word-emoji substitution with adult native speakers of Danish with no known reading difficulties. Experiment 1 used single word or emoji recognition task, and measured recognition errors and times. We also used remote eye tracking in reading and comprehension tasks, with different emoji positions in sentences (Experiment 2), and word-emoji substitutions in sentences and short texts (Experiment 3). Measures included fixation durations, skipping and back tracking, as well as overall reading speed and comprehension.

Our findings suggest that word-emoji substitution does not markedly change the core properties of reading process. We discuss the implications of these results in context of E-Z Reader model of eye movement control during reading, and the apparent robustness of alphabetic reading process to inclusion of logographic symbols.

Scene Perception (Room 10 | 10:30 – 12:30)

Beyond the Screen's Edge Part 1: Eye and Head Movements While Looking at Rotated Scenes in VR

NICOLA C. ANDERSON, WALTER F. BISCHOF, and ALAN KINGSTONE, *University of British Columbia*

We examined the extent to which image shape (square vs. circle), image rotation, and image content (landscapes vs. fractal images) influenced eye and head movements. Both the eyes and head were tracked while observers looked at natural scenes in a virtual reality (VR) environment. In line with previous work, we found a horizontal bias in saccade directions, but this was affected by both the image shape and its content. Interestingly, when viewing landscapes (but not fractals), observers rotated their head in line with the image rotation, presumably to make saccades in cardinal, rather than oblique, directions. We discuss our findings in relation to current theories on eye movement control, and how insights from VR might inform traditional eye-tracking studies.

Beyond the Screen's Edge Part 2: Eye and Head Movements While Looking at Rotated Panoramic Scenes in VR

WALTER F. BISCHOF, NICOLA C. ANDERSON, MICHAEL T. DOSWELL and ALAN KINGSTONE, *University of British Columbia*

Observers looked at panoramic, 360 degree scenes using VR goggles while eye and head movements were tracked. Fixations were determined using IDT (Salvucci & Goldberg, 2000) adapted to a spherical coordinate system. We then analyzed a) the spatial distribution of fixations and the distribution of saccade directions, b) the spatial distribution of head positions and the distribution of head movements, and c) the relation between gaze and head movements. We found that, for landscape scenes, gaze and head best fit the allocentric frame defined by the scene horizon, especially when taking head tilt (i.e., head rotation around the view axis) into account. For fractal scenes, which are isotropic on average, the bias toward a body-centric frame gaze is weak for gaze and strong for the head. Furthermore, our data show that eye and head movements are closely linked in space and time in stereotypical ways, with volitional eye movements predominantly leading the head. We discuss our results in terms of models of visual exploratory behavior in panoramic scenes, both in virtual and real environments.

CA/C ratio is a key parameter for adaption to stereoscopic viewing

Pascaline Neveu[1], Corinne Roumes[2], Charles-Antoine Salasc[1], Philippe Fuchs[3] and Anne-Emmanuelle Priot[1,4]

1 *Institut de recherche biomédicale des armées (IRBA), 1 place Général Valérie André, BP 73, 91223 Brétigny-sur-Orge CEDEX, France*
2 *Direction centrale du service de santé des armées (DCSSA), 60 boulevard du général Martial Valin 75015 Paris, France*
3 *Mines ParisTech (ENSMP), 60 Boulevard Saint-Michel, 75272 Paris CEDEX 06, France*
4 *INSERM U1028, CNRS UMR 5292, Lyon Neuroscience Research Center, 69500 Bron, France*

Stereoscopic displays challenge the neural cross-coupling between accommodation and vergence by inducing a constant accommodative demand and a varying vergence demand. There is now some evidence that stereoscopic viewing can induce discomfort and changes in oculomotor parameters. However, the adaptability of oculomotor system to this conflict is still a subject of debate. Stereoscopic viewing calls for a decrease in the cross-coupling through the CA/C ratio, helping the accommodative system to remain in focus by reducing vergence accommodation. The present study sought to investigate its adaptability when using a stereoscopic viewing exposure.
Cross-coupling (CA/C and AC/A ratios) and tonic components of vergence and accommodation were assessed in twelve participants with good stereoscopic vision before and after a 20-minute exposure to stereoscopic viewing. Vergence responses were measured during the exposure using an eye-tracker. During stereoscopic viewing, vergence demand oscillated from one to three meter angles along a virtual sagittal line in sinusoidal movements, while accommodative demand was fixed at 1.5 diopters.
Results showed a significant decline in the amplitude of the in-depth oscillatory vergence response
over time and a decreased CA/C ratio, with no change in the AC/A and tonic components. Our findings demonstrate for the first time that the CA/C ratio can exhibit adaptive adjustments at short term. The small decrease in CA/C suggests an incomplete adaptive process as shown by the progressive decline of the vergence amplitude.

Object search in real-world scenes with central and peripheral scene degradation

Anke Cajar, Ralf Engbert, & Jochen Laubrock

When searching for an object in a scene, central and peripheral vision are best suited for different tasks. Searchers use coarse-grained peripheral vision for selecting new scene regions that potentially contain the target object, and fine-grained central vision for identifying objects. Here we investigated how the attenuation of high or low spatial frequencies (i.e., fine-grained or coarse-grained information) in central or peripheral vision affects object search in color and grayscale real-world scenes. To this end, gaze-contingent low-pass or high-pass filters attenuating high or low frequencies, respectively, were applied to scenes which viewers searched for an object that was either present or absent. Results showed that, compared with an unfiltered control condition, peripheral filtering and central high-pass filtering hardly affected search accuracy. However, accuracy dropped dramatically with central low-pass filtering, even to chance level when searching grayscale scenes. Decomposing search times revealed that peripheral filtering increased scanning time (i.e., the time until first fixating the target). On the other hand, central filtering increased target verification time (i.e., the time from first fixating the target to responding). Verification time increased only slightly with central high-pass filtering, but increased strongly with central low-pass filtering, where viewers often relied on near-central peripheral vision for target identification. We conclude that attenuating spatial frequencies in peripheral vision hampers object localization, thus increasing search times. Attenuating high frequencies in central vision severely hampers the verification of object identity, especially when color is absent, whereas attenuating low frequencies in central vision hardly impairs search.

Across the Scene Depth Plane: Differing impacts of foreground and background information

Monica S. Castelhano

When you walk into a room, you perceive visual information that is both close to you and further in depth. Researchers have postulated that scene representations consist of background elements that provide a scaffold for more detailed foreground elements (Castelhano, Fernandes & Theriault, 2018; Davenport & Potter, 2004). Here, we investigated how processing is affected by information across scene depth.
First, we examined the role of foreground and background information on initial scene perception using Chimera scenes (images with foreground and background from differing scene categories). Across three experiments, we found a Foreground Bias, in which foreground information strongly influenced initial scene interpretation. It persisted when fixation position was modified and when task emphasized overall scene information. We conclude that the Foreground Bias arose from an initial prioritization of information closer to the observer.
Second, we investigated whether initial processing differences (i.e., Foreground Bias) could result in search efficiency differences. Again, Chimera scenes were used and targets could appear either in the foreground or background. Results showed participants had shorter response times, fewer fixations to the target, and a more direct scan path to foreground targets. This pattern was not explained by target size or sematic consistency of information at specific depths. Together, these studies suggest processing differences across scene depth, which have implications for scene and depth processing.

References

Castelhano, M. S., Fernandes, S., & Theriault, J. (2018). *JEP: LMC*,in press.
Davenport, J. L., & Potter, M. C. (2004). *PsychSci*,15(8),559–564.

Foveal vs. extrafoveal processing of object semantics during scene viewing: fixation-related N400 effects

Moreno I. Coco[ad], Antje Nuthmann[b] and Olaf Dimigen[c]

a *School of Psychology, The University of East London, London, UK*
b *Department of Psychology, Christian-Albrechts-Universität Kiel, Kiel, Germany*
c *Department of Psychology, Humboldt-Universität zu Berlin, Berlin, Germany*
d *CICPSI, Faculdade de Psicologia, Universidade de Lisboa, Lisboa, Portugal*

The processing of object-scene consistency continues to be a hotly debated issue. Results from eye-movement studies have been mixed, ranging from rapid processing of object-scene semantics in peripheral vision to a lack of evidence for extrafoveal semantic processing. In this study, we aimed to bring new evidence to this debate by simultaneously recording eye-movement behavior and associated EEG activity as participants inspected images of real-world scenes (e.g. a bathroom) in which the consistency of a target object (e.g. toothpaste vs. flashlight) was manipulated. The eye-movement data showed that inconsistent objects were prioritized over consistent objects (looked at earlier) and were more effortful to process (looked at longer). Crucially, the analysis of fixation-related brain potentials (FRPs) evoked by the first fixation on the target object (t) and the preceding fixation (t-1) demonstrated a frontocentral N400 effect initiated during the pre-target fixation t-1, which continued throughout fixation t. Our results demonstrate that the N400 triggered by object inconsistency generalizes from steady-fixation paradigms to unconstrained viewing of naturalistic scenes. Moreover, the results suggest that the extraction of object semantics already began in extrafoveal vision. Importantly, we did no find a significant effect of object-scene consistency on the probability of immediate target fixation and on the ERP aligned to scene onset, which suggests that object-scene semantics was not immediately accessed. Taken together, the results emphasize the usefulness of combined EEG/eye-movement recordings for understanding high-level scene processing.

Cognition I (Room 5 | 10:30 – 12:30)

Talking about what we see: Eye movements in dynamic scenes are not under the control of linguistic stimuli

Roberto G. de Almeida
(roberto.dealmeida@concordia.ca)
Department of Psychology, Concordia University
Montreal, QC, Canada
Julia Di Nardo
(jdinardo79@gmail.com)
Department of Psychology, Concordia University
Montreal, QC, Canada
Caitlyn Antal
(caitantal@gmail.com)
Department of Psychology, Concordia University
Montreal, QC, Canada

As Macnamara (1978) once asked, how can we talk about what we see? We report on a study manipulating realistic dynamic scenes and sentences aiming to understand the interaction between linguistic and visual representations in real-world situations. Specifically, we monitored participants' eye movements as they watched video clips of everyday scenes while listening to short sentences describing these scenes. We manipulated two main variables: (a) verb semantic class with causatives (e.g., break) and perception/psychological verbs (e.g., notice); and (b) the action/motion of the agent in the unfolding event—towards a target object (always the referent of the verb-complement noun), away from it, or neutral (when they remained performing a given activity such as cooking). Scenes and sentences were synchronized such that the verb onset corresponded to the first video frame of the agent motion towards or away from the object. Results show effects of agent motion but weak verb-semantic restrictions: causatives draw attention to potential referents of their grammatical complements faster than perception verbs, but only when the agent moves towards the target object. Crucially, we did not find anticipatory verb-driven eye movements toward the target object, contrary to studies using non-naturalistic and static scenes. We propose that linguistic and visual computations in real-world situations occur largely independent of each other during the early moments of perceptual input, but rapidly interact at a central, conceptual system using a common, propositional code. Implications for language use in real world contexts and the so-called 'visual world' experimental paradigm are discussed.

Using pupil size and eye-blink rate to investigate brain processes underlying cognitive flexibility.

Pajkossy Péter, Szőllősi Ágnes, Demeter Gyula, Racsmány Mihály
Institute of Cognitive Neuroscience and Psychology, Research Centre for Natural Sciences, Hungarian Academy of Sciences
Department of Cognitive Science, Budapest University of Technology and Economics

Pupil size and eye-blink rate index the activity of noradrenergic (NA) and dopaminergic (DA) neurotransmitter systems (see e.g. Eckstein et al, 2016). As commercial eye-trackers are capable off measuring these variables, eye-tracking can be used to assess how these neurotransmitter systems underlie information processing. We demonstrated this in four experiments. Because both NA and DA are involved in adapting to environmental changes (Ashton-Jones & Cohen, 2005; Cools & D'Esposito, 2011), we investigated how pupil size and eye-blink rate change during tasks requiring flexible shifting of attention (Exp 1-3) or altering knowledge about potential rewards (Exp 4). In each experiment, two stimuli with different features were presented several times. One of them was rewarded according to a rule. Based on feedback received for previous choices, participants had to choose the rewarded stimulus. Crucially, the rule changed periodically, and after rule shifts, participants had to figure out the new rule. In line with our predictions, the measured psychophysiological variables changed after rule shifts: pretrial, tonic pupil size increased (Exp 1-4), whereas task-evoked, phasic pupil dilation decreased (Exp 3-4). Eye-blink rate also increased after rule shifts (Exp 2-3), but only if new stimuli were introduced. These results are in line with theories linking NA and DA to regulating trade-offs between opposing information-processing demands (NA: exploration vs. exploitation; DA: maintenance vs. updating of working memory representations). Thus, assessing NA and DA levels indirectly with eye-tracking technology is a nonintrusive and cost-effective method to investigate how NA/DA activity underlies higher level cognitive functions.
Keywords: pupillometry, eye-blink rate, attentional set shifting, reversal learning, noradrenalin, dopamin

References:

Eckstein, M. K., Guerra-Carrillo, B., Singley, A. T. M., & Bunge, S. A. (2017). *Beyond eye gaze: What else can eyetracking reveal about cognition and cognitive development?* Developmental cognitive neuroscience, 25, 69-91.

Aston-Jones, G., and Cohen, J. D. (2005). *An integrative theory of locus coeruleus-norepinephrine function: adaptive gain and optimal performance*. Annu. Rev. Neurosci. 28, 403–450. doi: 10.1146/annurev.neuro.28.061604.135709

Cools, R., and D'Esposito, M. (2011). *Inverted-u-shaped dopamine actions on human working memory and cognitive control*. Biol. Psychiatry 69, e113–e125. doi: 10.1016/j.biopsych.2011.03.028

What the pupils tell us about aesthetics (or not)

Sophie G. Elschner and Wolfgang Gaissmaier

As a theory of aesthetic appreciation, processing fluency postulates a general preference for easy to process stimuli. Among others, it has been demonstrated that paintings that are low on abstractness – therefore easier to process – elicit greater pupil response than highly abstract ones (Elschner et al., 2018; Kuchinke et al., 2009). This contradicts literature on cognitive load reporting stronger pupil dilation under greater effort (e.g. Steinhauer et al., 2004). Thus, we aimed to further identify whether pupil dilations mirror effects of fluency. In three experiments, we investigated whether these effects already occur at faster responses that preceed conceptual stages: Partcipants completed a categorization task where they either judged whether a cloud of dots was symmetric or asymmetric (Experiment 1), or whether paintings were expressionist or cubist (Experiment 2). In Experiment 3, we adapted the task used in Elschner et al. (2018), where participants indicated the moment of content recognition when viewing paintings, but included a time constraint. Fluency was either operationalized as symmetry category (Experiment 1) or as abstractness (Experiments 2 & 3). Preference ratings on a 7-point scale, response times and peak dilations were recorded. Concordant with fluency theory, preference ratings were highest and response times generally fastest for easy to process stimuli in all experiments. Results for PDs were mixed: While they were generally greater for hard to process stimuli in the first two experiments (concordant with cognitive load literature), no effects were found in the third. The utility of the employed methods for aesthetic research is discussed.

No effect of negation in counterfactuals: Evidence from eye-tracking in the visual world paradigm

Isabel Orenes (1), Juan A. García-Madruga (1), Orlando Espino (2) & Ruth Byrne (3).
(1) Universidad Nacional de Educación a Distancia (UNED)
(2) Universidad de La Laguna
(3) Trinity College Dublin, University of Dublin, Ireland

Counterfactuals such as 'if she had arrived early, she would have bought roses" require people to envisage two possibilities, the conjecture, 'she arrived early and she bought roses' and the presupposed facts, 'she did not arrive early and she did not buy roses'. We report two eye-tracking studies to examine how people process these two possibilities and whether they are equally accessible. In Experiment 1, people heard affirmative counterfactuals while looking at four printed words on a computer screen, e.g., 'roses', 'no roses', 'carnations', 'no carnations'. The results showed that participants first looked at 'roses' and then its implicit negation, 'carnations'. In contrast, for negated counterfactuals, 'if she had not arrived early, she would not have bought roses', they looked only at 'roses'. In Experiment 2, we corroborated these results for counterfactuals in a binary context (there were roses or carnations in the shop) and multiple contexts (there were roses or carnations or lilies or daisies). We discuss how the understanding of counterfactuals and negation affects the accessibility of the two possibilities.

Saccade suppression depends on scene context

Zimmermann, Eckart
Institute for Experimental Psychology, University of Düsseldorf

One of the most frequently occurring selective blindnesses is saccade suppression. Visual sensitivity decreases drastically while we perform saccade eye movements. This effect lasts for a period of about 100 ms around saccade initiation. Here, I investigated whether trans-saccadic motion perception is context-sensitive. In this view, saccade suppression strength should be flexibly variable depending on the current scene context.
Performing saccades in a structured environment yields a persistent intrasaccadic feedback that contains high variance. In order to create an experimental environment in which intrasaccadic motion information could be systematically varied, a horizontal grating was used as a background on which horizontal saccades had to be performed.
Observers executed many saccades in context trials after which their current saccade suppression strength was tested in separate test trials.
In sessions where that background was kept stationary - thus inducing negligible intrasaccadic motion - a minimal amount of saccade suppression was observed.
Then, in other sessions, the grating was shifted upwards as soon as a saccade was detected. Consistent with context-sensitivity, saccade suppression magnitude was very strong in the following test test trials.
Further experiments revealed that the context-sensitive suppression of motion was direction-specific. Discrimination was hampered only if the motion in the test trials had the same direction as that in the context trials. These data suggest that the sensorimotor system selectively weighs down motion information depending on the recent intrasaccadic reliability of vision.

Task-dependent eye-movement behavior in head unrestrained scene viewing

Daniel Backhaus, Lars O.M. Rothkegel, Ralf Engbert, & Hans A. Trukenbrod
University of Potsdam

The generalizability of theoretical implications derived from the static picture viewing paradigm has been questioned (Tatler et al., 2011) because of its typical experimental procedures (e.g., no explicit task, use of a chin rest, sudden image onset). Therefore, a paradigmatic shift to more natural tasks has been proposed. Here we investigated eye movements from participants standing in front of a projector screen while exploring images under head unrestrained viewing with mobile eye-tracking. For the analysis, head-centered gaze data were transformed into image-centered coordinates after the experiment. Our results show differences in temporal and spatial eye movement parameters induced by the tasks. For example, we found task-dependent differences in the tendency to fixate images near the center (Fig. 1). Our findings demonstrate the feasibility to study fine-scaled task dependencies with current mobile eye-trackers in a highly controlled experimental setting that permit more natural viewing behavior than typical experiments implementing the static picture viewing paradigm.

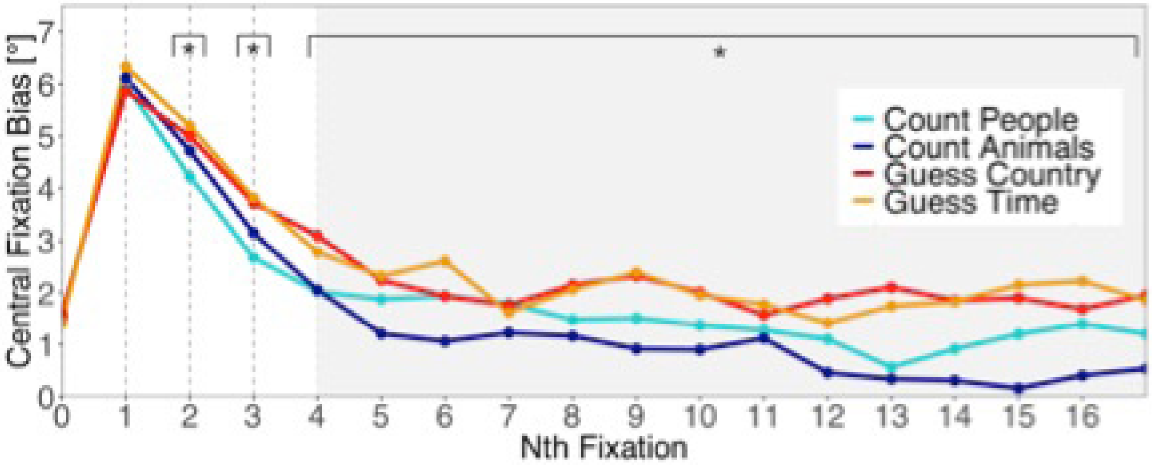

Fig. 1: Temporal evolution of the central fixation bias measured as the average distance to image center. Each line corresponds to one of the four instructions. The abscissa provides the expected distance to center, if fixations were uniformly placed on an image. Level of significance: * < 0.05

Tatler, B. W., Hayhoe, M. M., Land, M. F., & Ballard, D. H. (2011). Eye guidance in natural vision: Reinterpreting salience. *Journal of Vision*, 11(5):5, 1–23. DOI: 10.1167/11.5.5.

Session 3

Attention I (Room 10 | 14:00 – 16:00)

Is covert visual search biologically constrained by the effective oculomotor range?

Daniel T. Smith; Soazig Casteau
Durham University

We typically move attention around the environment with overt eye-movements. However, it is also possible to orient attention covertly, such that the 'spotlight' of attention is moved independently of the locus of gaze. While there is enduring debate about the relationship between the mechanisms controlling covert and overt attention, one influential view is that shifts of covert attention are achieved by planning (but not executing) and overt eye movement. We have previously shown that placing stimuli at locations beyond the effective oculomotor range (EOMR) interfered with covert, exogenous orienting to peripheral cues but not covert endogenous orienting driven by symbolic cues (e.g. Smith et al., 2012; Smith & Casteau 2019). Here, we examined whether the association between exogenous attention and the EOMR observed in cueing tasks generalised to visual search tasks. We first measured the EOMR for each participant, then presented search arrays that were either within or beyond their EOMR. Feature search was significantly slower when stimuli were presented beyond the range of eye-movements whereas conjunction search was unaffected. In a second study we parametrically varied the location of the array to better establish how well changes in search performance mapped onto the range of eye-movements. The results confirmed that preattentive search was restricted to the locations within the range of eye-movement. We conclude that optimal covert, exogenous orienting of attention depends on the oculomotor system, consistent with an Oculomotor Readiness hypothesis of Exogenous Orienting.

Casteau, S., & Smith, D. T. (2018a). Covert attention beyond the range of eye-movements: Evidence for a dissociation between exogenous and endogenous orienting. *Cortex*. doi:https://doi.org/10.1016/j.cortex.2018.11.007

Smith, D. T., Schenk, T., & Rorden, C. (2012). Saccade preparation is required for exogenous attention but not endogenous attention or IOR. *J Exp Psychol Hum Percept Perform*, 38(6), 1438-1447. doi:10.1037/a0027794

Presaccadic attention modulates visual responses via response gain changes

Marisa Carrasco, Jasmine Pan & Hsin-Hung Li
NYU

Attention is an essential neural and cognitive process enabling the brain to selectively process information. Characterizing the dependency of attention on stimulus contrast is essential for understanding the computations underlying attentional modulations. Whereas the contrast dependency of covert attention (attention without concurrent eye movements) has been extensively studied (Carrasco, 2011), we know far less about the contrast dependency of overt attention (attention with concurrent eye movements). Here, we investigated the contrast dependency of presaccadic attention, a form of overt attention that is deployed to the saccade target just before saccade onset. In three psychophysical experiments, we found that presaccadic attention enhanced visual performance for the stimulus at the saccade target location compared to a neutral baseline in which no saccade was executed. Concurrently, presaccadic attention suppressed performance at a non-target location. Measuring enhancement and suppression across various stimulus contrasts and fitting the data with Naka-Rushton functions, revealed that both enhancement and suppression are mediated via response gain changes (asymptotic performance), instead of contrast gain changes (semi-saturation contrast). Moreover, these response gain changes were robust even with increased location uncertainty of the targets. Model simulations underscored the differences between our results and the predictions of the Reynolds-Heeger normalization model of attention, a computational model that explains perceptual modulations by covert attention. Beyond experimental dissociations between presaccadic and covert attention in previous studies (eg, Li, Barbot, Carrasco, 2016), the present results suggest that the computation of perceptual modulations by these two types of attention cannot be unified under the same framework.

1- **Carrasco**, *Vision Research*, 2011
2- **Reynolds & Heeger**, *Neuron*, 2009
3- **Zhao, Gersch, Schnitzer, Dosher & Kowler**, *Vision Research*, 2012
4- **Li, Barbot & Carrasco**, *Current Biology*, 2016
5- **Li, Pan & Carrasco**, *Scientific Reports*, 2019

The influence of spatial priors and visual processing time on attentional selection and oculomotor control

Luca Wollenberg[1,2], **Heiner Deubel**[2]

1. *Allgemeine und Experimentelle Psychologie, Department Psychologie, Ludwig-Maximilians-Universität München, Munich, Germany*
2. *Graduate School of Systemic Neurosciences, Ludwig-Maximilians-Universität München, Munich, Germany.*

In order to efficiently explore the environment via saccadic eye movements, our oculomotor system relies on attentional mechanisms for saccade target selection. However, when multiple nearby stimuli compete for attentional selection, one can frequently observe so called averaging saccades landing at an intermediate location between the stimuli. In a recent study (Wollenberg, Deubel, & Szinte, 2018, PLoS biology) we observed that visual attention is not deployed at the endpoint of averaging saccades but rather equally distributed across two competing saccade targets, suggesting that oculomotor averaging arises from unresolved saccade target selection. Here, we wanted to investigate how oculomotor competition is resolved under conditions where participants have prior knowledge about target or distractor locations. Participants had to saccade towards a target, presented together with a nearby distractor, and discriminate localized probes during saccade preparation. Importantly, we systematically varied the degree of preknowledge about target and distractor locations, and introduced delayed go-signals to differentially increase visual processing time. As expected, saccade accuracy improved with preknowledge and increasing visual processing time. At short latencies, visual attention was facilitated at both target and distractor locations. Across time, however, attentional performance was selectively sustained at the target while it decayed at the distractor. These data suggest that oculomotor competition among targets and distractors is reflected in an initial attentional capture at the competing visual onsets and resolved only later via active attentional target selection. Moreover, oculomotor averaging seems to disappear at the time when attentional selection of the target is resolved.

This work was supported by grants of the Deutsche Forschungsgemeinschaft to HD (DE336/5-1 and RTG 2175)

Featural attention in the development of category learning

Ingmar Visser, Maartje Raijmakers, Daan van Renswoude
University of Amsterdam

Under what circumstances and with what stimuli similarity based versus rule based strategies are used has been a contested issue for a long time (eg Kemler-Nelson, 1984, versus Milton, Inkster, Wills, 2014). Kloos & Sloutsky (2008) present an experiment (1c) where both children and adults prefer similarity based category learning using dense categories under unsupervised learning conditions. In the current study we replicate that experiment in a large sample of children (n=106) and adults (n=82) to facilitate studying individual differences using latent class analysis. To validate the findings from the latent class analysis, we performed a second experiment that uses eye-tracking to study the features that participants focus on during category learning and during the generalization phase. The results of both experiments lead to the same conclusion: a large majority (60-70%) of participants were found to use a single dimensional strategy, whereas only a small minority (8-13%) possibly uses an overall similarity strategy, and the remaining participants have an inconsistent or guessing strategy. The fixation patterns align precisely with the behavioral data: most participants show fixations to a single feature only whereas a minority pays attention to multiple dimensions of the stimuli after learning has completed. Examining eye-movements in this task has provided novel evidence to a long-standing dispute about the cognitive processes underlying (the development of) category learning.

Kloos & Sloutsky, 2008, *JEPG*.
Wills, Inkster, & Milton, 2015, *CogPsy*.
Kemler-Nelson, 1984, *JVLVB*.

Meaning or Salience? What drives attention over real-world scenes in infants and adults?

Daan van Renswoude, Ingmar Visser, Maartje Raijmakers
University of Amsterdam; University of Amsterdam; VU Amsterdam

What factors drive infants' and adults' gaze behavior over complex real-world scenes? In adults, scene viewing is characterized as an interplay between low-level perceptual salience (e.g., contrast, color and orientation of pixels) and higher order top-down information such as meaningful objects and context. This interplay between exogenous and endogenous factors develops in infancy and scene viewing is a suitable paradigm to quantify how infants' visual attention develops into more adult like attention. In this presentation data of three different scene viewing studies with both infants (3 – 18-month-olds, N = 150) and adults (N = 100) is discussed. Together these studies provide evidence for a slight developmental shift from more salience-based towards more meaning-based attention. However, the most striking finding is the overall similarity between general eye movement patterns in the infant and adult data. Similar to adults, infants have a center and horizontal bias, they are much more likely to fixate objects than the background. Moreover, infants also fixate objects in the center and have longer fixation durations on meaningful objects than on the background. In the adult literature these findings are often interpreted as evidence for more meaning-based attention. The fact infants show similar tendencies challenges the meaning-based attention account, as this would imply that objects in complex real-world scenes are already meaningful for infants. A more likely conclusion to draw from this set of studies is that infant and adult fixations are driven by similar underlying low-level processes.

Computational Modeling, Data Analysis & Quality (Room 5 | 14:00 – 16:00)

Temporal control in modelling eye fixations (TIME)

Casimir J.H. Ludwig, Hans A. Trukenbrod, Ralf Engbert
University of Bristol; University of Potsdam; University of Potsdam

In natural vision, processing difficulty will change periodically during sequences of fixations. To ensure adaptive behaviour, it is important that fxation duration is adjusted to systematic changes in difficulty. We aim to understand these temporal control mechanisms by developing and fitting computational models to extended sequences of fixations. Observers generated large sequences of fixations in a gaze-contingent paradigm where the fixated item cued the direction of the next saccade. Items were embedded in visual noise, which changed periodically to increase or decrease processing difficulty. The key features of our data are: fixation duration adapts immediately to an increase in processing difficulty; adaptation to increasing difficulty initially "overshoots", but then decreases again to a new steady state; adaptation of fixation duration to decreasing difficulty tends to be delayed (but not always). Here we report simulations and Bayesian fits of a model based on evidence accumulation to a threshold. The model has three key mechanisms that operate on different timescales: (i) during the first fixation after any change in difficulty, foveal inhibition transiently reduces the drift rate; (ii) the drift rate itself varies with the quality of evidence presented during a given fixation; (iii) the decision threshold adjusts over the course of several fixation in response to the recently experienced processing difficulty. These mechanisms themselves are symmetric with respect to processing difficulty. However, their interaction over different timescales explains the asymmetry in adaptation between increasing and decreasing difficulty, as well as the initial overshoot in fixation duration after an increase in difficulty.

popEye - An integrated R package to analyse eye movement data from reading experiments

Sascha Schroeder
University of Goettingen
sascha.schroeder@psych.uni-goettingen.de

popEye is an R package for the analysis of eye-tracking data from reading experiments. A unique feature of popEye is that it allows to analyse data collected using different eye tracking devices (SR research, SMI, etc.) and software packages (Experiment Builder, EyeTrack, etc.) within the same workflow. In addition, popEye aims at analysing reading data from all levels of reading and using different paradigms (e.g., text reading, single sentence reading, boundary paradigm, fast priming paradigm).
popEye reconstructs the stimulus input as shown by the display software used in the experiment, preprocesses the data, and assigns fixations to various linguistic units on different levels (letters, lines, words, sentences, screens, texts). In addition, flexible interest areas on the sub-lexical (e.g., morphemes) or super-lexical level (phrases or paragraphs etc.) can be defined. All processing is done directly in terms of linguistic units and, as a consequence, the hierarchical structure of language can be exploited during analysis. This allows popEye to solve some problems that have typically haunt reading research (e.g., automatic detection of return sweeps, align fixations to lines). Moreover, popEye provides several options for data preprocessing, context-sensitive plots, and flexible cleaning functions that greatly ease data analysis and make it more transparent.
In this talk, I will introduce popEye, demonstrate how it is used, and explain how to contribute to the package using the projects' GitHub site (https://github.com/sascha2schroeder/popEye).

Disentangling cognition and eye movements in EEG using generalized additive mixed models

Radha Nila Meghanathan[1,2,] **Andrey R. Nikolaev**[1]**, Cees van Leeuwen**[1,2]

1 *Laboratory for Perceptual Dynamics, Brain & Cognition Research Unit, KU Leuven - University of Leuven, Leuven, Belgium*

2 *Technical University Kaiserslautern, Kaiserslautern, Germany*

When EEG is co-registered with eye movements in free-viewing tasks, EEG response to cognitive processes of interest maybe confounded with that to sequential eye movements (Dimigen et al 2011; Nikolaev et al 2016). These two can be disentangled using generalized additive mixed models (GAMM) with eye movement parameters and experimental conditions as predictor variables. Since GAMM can account for non-linearity in data, it is particularly suited to modeling EEG. GAMM was used to investigate the role of visual saliency in saccade guidance in an EEG eye movement co-registration study. Participants were asked to search, during an 8s interval, for a contour formed by 7 collinear Gabor elements embedded in a field of similar but randomly oriented distractors. Participants were then asked to indicate whether the contour was present, which was the case in half of the trials. Since the interval preceding saccade onset (presaccadic interval) involves saccade planning to the next fixation location, EEG epochs in this interval were modeled using GAMM with visual saliency of the subsequent fixation location and perisaccadic eye movement parameters as predictors. Besides the effect of fixation duration and saccade size on pressaccadic EEG, low presaccadic EEG amplitude was associated with high saliency at the next fixation location in both contour-present and contour-absent conditions (van Humbeeck et al 2018). This reveals the role of bottom-up saliency in saccade guidance. Using GAMM, the effects of visual saliency on saccade guidance were successfully isolated from the effect of eye movements on EEG.

Small eye movements cannot be reliably measured by video-based P-CR eye-trackers

Kenneth, Holmqvist ; Pieter, Blignaut
Regensburg Universität; University of the Free State

For evaluating whether an eye-tracker is suitable for measuring microsaccades, Poletti and Rucci (2015) advocate that a measure called 'resolution' be used rather than the more established root-mean-square of the sample-to-sample distances (RMS-S2S). Many open questions exist around the resolution measure, however. Resolution needs to be calculated using data from an artificial eye that can be turned in very small steps. Furthermore, resolution has an unclear and uninvestigated relationship to the RMS-S2S and STD (standard deviation) measures of precision (Holmqvist and Andersson, 2017, p. 159-190), and there is another metric by the same name (Clarke et al., 2002), which instead quantifies errors amplitude measurements. In this paper, we present a mechanism - the Stepperbox – for rotating artificial eyes in arbitrary angles from 1 (arcmin) and upward. We then use the Stepperbox to find the minimum reliably detectable movement in 10 videobased eye-trackers (VOGs) and the DPI. We find that resolution correlates significantly with RMS-S2S and to a lesser extent also with STD. In addition, we find that although most eye-trackers easily detect small eye movements, the movements of amplitudes between 1 and 5° are frequently erroneously measured by video-based eye-trackers. We show evidence that the corneal reflection (CR) feature of these eye-trackers is a major cause of the erroneous measurements. Our data furthermore suggest that some results from research where the amplitude of small eye movements have been measured with past or current video-based eye-trackers may need to be reconsidered. This includes, for instance, research on microsaccades, reading and vergence.

A computational model of rhythmic auditory attention predicts the pupillary response to music

Lauren Fink[1], Brian Hurley[1], Joy Geng[1], Elke Lange[2], Petr Janata[1]
1 University of California, Davis
2 Max Planck Institute for Empirical Aesthetics

Changes in pupil diameter are known to reflect sub-second changes in attentional state related to locus-coeruleus noradrenergic functioning (Berridge & Waterhouse, 2003; Reimer et al., 2016). In three experiments, we examine the continuous pupillary signal while participants listen to dynamically changing musical scenes. We use a linear oscillator model to predict how each musical stimulus will elicit rhythmic fluctuations in attention. We test our predictions against 1) participants' perceptual thresholds for detecting intensity deviants (dB SPL) adaptively inserted into musical scenes 2) participants' pupil dilation response (PDR) to deviants and 3) participants' continuous pupil signal while listening to both simple rhythmic or complex 'real-world' instrumental music. The model successfully predicted participants' perceptual thresholds, with higher thresholds for moments of lower predicted attentional salience and vice versa. The PDR was evoked for both increment and decrement deviants (and is therefore not a function of stimulus amplitude); maximum evoked pupil size was a significant predictor of whether a deviant was detected or missed on any given trial, as was output from our computational model. Compared to the pupil signal during silent fixation or with shuffled stimulus labels, there was significantly more phase coherence between the model-predicted and recorded pupil signals for each stimulus. Coherence between the pupil and music was correlated with participants' feelings of being 'absorbed in the music' and not with other ratings, like valence or arousal. We conclude that the continuous pupillary signal can reveal psychologically relevant, fine-grained information about an attended auditory stimulus.

Keywords: auditory, attention, pupillometry, perceptual threshold, computational modeling

Do pupil-based binocular video eye trackers reliably measure vergence?

Ignace T.C. Hooge[1], Roy S. Hessels[2] & Marcus Nyström[3]
1 *Experimental Psychology, Utrecht University, Utrecht, the Netherlands*
2 *Experimental Psychology, and Developmental Psychology, Utrecht University, Utrecht, the Netherlands*
3 *Lund University Humanities Lab, Lund, Sweden*

Distance to the binocular fixation point, vergence angle and fixation disparity are useful measures e.g., to study visual perception, binocular control in reading and attention in 3D. Are binocular pupil-based video eye trackers accurate enough to produce meaningful binocular measures? Recent research (Wyatt et al. 2010; Wildenmann & Schaeffel, 2013; Drewes et al., 2014) revealed large idiosyncratic systematic errors due to pupil-size changes. We investigated whether the pupil-size artefact in the separate eyes may cause the eye tracker to report apparent vergence changes when the eyeballs do not rotate. To evoke large and small pupils, observers continuously fixated a dot on a screen that slowly changed from black to white in a sinusoidal manner (0.125Hz). Gaze positions of both eyes were measured with an EyeLink 1000 plus. We obtained vergence changes up to 2° in the eye tracker signal. Inspection of the corneal refection signals confirmed that the reported vergence change was not due to actual eye rotation. Due to the pupil-size artefact, pupil-CR or pupil-only video eye trackers are not accurate enough to determine vergence, distance to the binocular fixation point and fixation disparity.

Applications: Medicine (Plenary room | 14:00 – 16:00)

How many cues does it take to find every cancer?

Dr Damien Litchfield
Edge Hill University
damien.litchfield@edgehill.ac.uk
Dr Tim Donovan
University of Cumbria
tim.donovan@cumbria.ac.uk

Understanding how observers interpret complex medical images and detect pathology is important as errors have serious health and economic implications. Visual search in this applied setting is typically compared between experts and novices to establish what processes experts optimise for high performance, e.g., more efficient eye movements (Donovan & Litchfield, 2013) or exploiting the first glimpse of the scene (Litchfield & Donovan 2016). Yet despite extensive training, experts still miss cancers (4-30%) and rarely achieve 100% cancer detection in experiments. Taking a novel experimental approach, novices were provided with increasing number of cues to establish what it would take to achieve 100% cancer detection. A key factor when finding 'lung nodules' in chest x-rays is that these targets have a variety of shapes and sizes, and their features can be mistaken for normal anatomy. Specifying the target-template so observers know what features precisely to find in images should increase performance, as should presenting this cue in the context of the image. Without cues novice accuracy is typically 50% whereas experts achieve 80%-90% (Donovan & Litchfield, 2013). Presenting 30 novices the precise visual depiction of cancer on 36 images (using a 1-pixel cropped border of the target) yielded 65% accuracy (1 novice achieved 100%). A separate group of 30 novices shown the target cue with a 100-pixel border (including surrounding spatial information), yielded 86% accuracy (and 4 achieved 100%). We discuss how observers make use of these cues and why cancer is still hard to find even when shown onscreen.

How Prior Knowledge Affects Problem-solving Performance in a Medical Simulation Game: Using Game-logs and Eye-tracking

Lee, Joy Yeonjoo ; Donkers, Jeroen; Jarodzka, Halszka; Van Merriëboer, Jeroen
Maastricht University; Maastricht University; Welten Institute; Maastricht University

Computer-based simulation games provide an environment to train complex problem-solving skills. Yet, it is largely unknown how the in-game performance of learners varies with different levels of prior knowledge. Based on theories of complex skill acquisition (e.g., 4C/ID (Van Merriënboer & Kirschner, 2018)), we derive four performance aspects that prior knowledge may affect: (1) systematicity in approach, (2) accuracy in visual attention and motor reactions, (3) speed in performance, and (4) cognitive load. This study aims to empirically test whether prior knowledge affects these four aspects of performance, using game-logs and eye-tracking in combination. A medical simulation game for resuscitation skills training was employed as the task environment. Participants were 24 medical professionals (experts, with high prior knowledge) and 22 medical students (novices, with low prior knowledge). After pre-training, they all played one scenario, during which game-logs and eye-movements were collected. A cognitive-load questionnaire ensued. During game play, experts demonstrated a more systematic approach, higher accuracy in visual selection and motor reaction, and a higher performance speed than novices. Their reported levels of cognitive load were lower. These results indicate that prior knowledge has a substantial impact on performance in simulation games, opening up the possibility of using our measures for performance assessment.

Van Merriënboer, J. J., & Kirschner, P. A. (2018). *Ten steps to complex learning: A systematic approach to four-component instructional design*: Routledge.

Eye tracking control improves functionality of a retinal prosthesis for the blind

Caspi, Avi
Jerusalem College of Technology, Second Sight Medical Products Inc., Wilmer Eye Institute

Visual restoration for the blind by electrical stimulation of the remaining cells in the optical pathway is feasible. For the Argus II retinal prosthesis, which is approved to treat blindness in many countries worldwide, electrical stimulation is based on an image captured by a head-mounted camera. Visual scanning with the prosthesis is achieved only by head movements.
The perceived location of an elicited phosphene, in world coordinates, is a function of the location of electrical stimulation on the retina, orientation of the eye within the head, and orientation of the head. Patients naturally integrate the eye's position with the retinotopic percept from the implant. The eye tracker measured gaze positions in real-time in order to adjust the region of interest (ROI) that was sent to the implant within the wide field of view (FOV) of the scene camera. The user was able to use combined eye-head scanning: shifting the camera by moving their head and shifting the ROI within the FOV by eye movement.
An eye tracker based on field-programmable-gate-array (FPGA) logic was used to develop a mobile and low power device. An eye trackers, based on PC and FPGA, integrated into the Argus II system were used to shift the line-of-sight based on eye position. We demonstrated with ten blind Argus II implantees that a scanning mode utilizing eye movements with this device improved pointing precision and reduced head movements in a localization task.

Eye movements in the formally blind

Paul Zerr[1,2], Jose Ossandon[1], Idris Shareef[3], Stefan Van der Stigchel[2], Ramesh Kekunnaya[3], Brigitte Röder[1]

1 *Biological Psychology and Neuropsychology, Hamburg University, Germany*

2 *Experimental Psychology, Helmholtz Institute, Utrecht University, the Netherlands*

3 *Child Sight Institute, Jasti V Ramanamma Children's Eye Care Center, LV Prasad Eye Institute, Hyderabad, India.*

Sensitive periods have previously been identified for several human visual system functions, such as global motion perception and face perception. Goal directed eye movements are crucial for efficient visual perception. Non-human primates and cats which had been visually deprived from birth have previously been shown to recover some oculomotor control following visual experience. The present study investigated whether sensitive periods exist in the development of human oculomotor control. Eye movements in individuals born with total bilateral cataracts were, at least one year after sight-restoration, compared to the eye movements of sight-restored late blind individuals, individuals with strong pathological nystagmus and controls with typical vision. Congenital cataract reversal individuals regained the ability to make systematic, purposeful gaze shifts, even after decades of blindness. The typical strong nystagmus of congenital cataract reversal individuals caused distorted eye movement trajectories, but measures of latency and accuracy were as expected from their prevailing nystagmus, that is, not worse than in individuals with a pathological nystagmus due to other reasons than a period of blindness. By contrast, saccade velocity was lower in congenital cataract reversal individuals than in any of the control groups. This first study on basic characteristics of oculomotor control in cataract reversal individuals has demonstrated a remarkable recovery of goal directed gaze shifts despite some remaining impairments in oculomotor control. Thus, the more severe visual impairments observed in this group for higher visual functions cannot be fully explained by a lack of basic goal directed eye movements.

Looking to collaborate: using mobile eye-trackers to explore interprofessional collaboration features in stroke care simulations

MacKenzie, D.E.[1], Neyedli, H.F.[2], Westwood, D.A.[2], Creaser, G.[3], Sponagle, K.[4], Hickey, E.[5], Merritt, B.K.[1], Miller, S.G. [6], Gubitz, G.[7], Dithurbide, L.[2], & Picketts, L.8

[1]Dalhousie Unviersity – Occupational Therapy; [2]Dalhousie Unviersity – Health and Human Performance; [3]Dalhousie Unviersity – Physiotherapy; [4]Dalhousie Unviersity - Pharmacy; [5]Dalhousie Unviersity – Communication Science and Disorders; [6]Dalhousie Unviersity – Emergency Medicine; [7]Dalhousie Unviersity – Neurology; [8]Dalhousie Unviersity – Center for Centre for Collaborative Clinical Learning and Research

Background: Stroke is the leading cause of long-term disability in North America and best practice care requires effective interprofessional collaboration (IPC). Situational awareness, communication, and coordination of services are critical elements of IPC and patient safety, but assessment of IPC is challenging and often relies solely on communication analysis. Little research has focused on how differences in attention allocation amongst team members might relate to effective IPC in the stroke care context.

Methods: In this pilot study we explored whether eye movements may provide additional insight into effective IPC in simulation. Sixteen participants (students enrolled in Medicine, Pharmacy, Occupational Therapy, Physiotherapy, Speech/Language Pathology, and Nursing) participated in an interprofessional module and were randomly assigned to four interprofessional teams. Participants wore SMI mobile eye-trackers during a simulation consisting of a 25-minute team meeting followed by a 25-minute simulated patient assessment. SMI BeGazeTM software was used for eye movement analysis (saccades, fixations, and area-of-interest analyses). Team performance was ranked separately for IPC and stroke competency by a panel of trained experts.

Findings: Preliminary results suggest the teams ranked higher on IPC had shorter fixation durations, higher frequency of fixations/sec, less variability in saccade velocity, and higher areas of interest (AOI) frequencies on the patient's chart. Next steps include event-based analyses of "critical incidents" identified by experts to determine if looking patterns at pivotal moments distinguish high from low ranked teams. Results will be compared to audio recording qualitative analysis and self-ranked IPC scores. Further results to be shared at the conference.

Stereoscopic eye tracking with an aspheric model of the cornea

J. Goossens
Cognitive Neuroscience Dept., Donders Institute for Brain, Cognition and Behaviour, Radboud University Nijmegen Medical Centre

A critical hurdle for accurate eye tracking in clinical populations has been the need for individual calibration of the tracker through a series of precise eye fixations of visual targets – a task many people with visual, oculomotor and/or cognitive impairments cannot perform accurately. To circumvent these complications, we recently developed a high-speed stereoscopic eye-tracker with two USB3 cameras that can estimate the orientation of the eye's optical axis directly from the stereo images of the pupil and corneal reflections of two infrared light sources (Barsingerhorn et al., Behav Res Methods, 2018). This greatly simplifies calibration to a one-point fixation procedure. However, stereoscopic eye-tracking methods have thus far assumed that the cornea is a sphere with only one refractive surface whereas the human cornea is slightly aspheric and has two refractive surfaces. This simplification causes significant errors in the resulting gaze estimates (Barsingerhorn et al., Biomed Opt Express, 2017). To address this problem, we developed new gaze reconstruction algorithms for our stereo eye-tracker that incorporate an aspheric model of the cornea (Navarro et al., J Opt Soc Am, 1985). Interestingly, these algorithms do not require additional calibration data; the cornea asphericity parameter (Q) can be estimated from spontaneous eye movements. Simulation results and empirical data show that these novel methods are both accurate and noise robust. We conclude that these techniques may be helpful in improving the quality of eye tracking in clinical populations and otherwise challenging subjects (e.g., young children).

Session 4

Tuesday, August 20th

Sentence and Text Processing I (Room 10 | 10:30 – 12:30)

On the segmentation of incremental words during Chinese reading

Li, Xingshan ; Zhou, Junyi
Institute of Psychology, Chinese Academy of Sciences; Fujian Normal University

In Chinese, there are some incremental words, in which multi-character words (e.g., [illegible], means landlady) contain a subset of characters that constitute another word (referred to as the embedded word, e.g., [illegible], means boss). In the current talk, we will introduce some recent progress on experimental and modelling studies on how Chinese readers segment words during natural sentence reading. In one of these studies, Chinese readers read sentences with some incremental words while their eye movements were monitored. We manipulated the plausibility of incremental words and their embedded words. Results showed an interaction between the plausibility of the whole words and the plausibility of the embedded words on the reading times on the target word region. When the whole incremental word was plausible, the plausibility of the embedded words did not affect reading time on the target region. However, when the whole incremental word was implausible, reading times on the implausible embedded word was longer than plausible embedded word. These results suggest that Chinese readers tend to segment incremental word as a whole, but they can adjust their segmentation strategy very quickly once they found the segmentation is incongruent with sentence context.

Key words: Chinese reading, word segmentation, eye movements

References:

Li, X., Rayner, K., & Cave, K. (2009). On the segmentation of Chinese words during reading. *Cognitive Psychology*, 58, 525-552.

Shen, W., Li, X., & Pollatsek, A. (2018). The processing of Chinese compound words with ambiguous morphemes in sentence context. *The Quarterly Journal of Experimental Psychology*, 71, 131-139 .

Yang, J., Staub, A., Li, N., Wang, S., & Rayner, K. (2012). Plausibility effects when reading one- and two-character words in Chinese: evidence from eye movements. *Journal of Experimental Psychology. Learning, Memory, and Cognition, 38(6)*, 1801–9.

Zhou, J., Ma, G., Li, X., & Taft M. (2018). The time course of incremental word processing during Chinese reading. *Reading and Writing, 31*(3), 607-625.

A large-scale eye-movement study of Chinese reading: Implications for models of reading

1. **Lili Yu** (*Department of Cognitive Science & Macquarie University Centre for Reading, Macquarie University, Australia*);
2. **Yanping Liu** (*Department of Psychology, Sun Yat-sen University, China*)
3. **Erik D. Reichle** (*Department of Psychology & Macquarie University Centre for Reading Macquarie University, Australia*)

In this talk, we will report the results of a large-scale eye-movement study of native Chinese speakers reading sentences in their native language. These sentences contained two-character target words that varied in terms of their frequency (high vs. low) and the frequency of their first characters (high vs. low), but otherwise matched for second-character frequency, character complexity, predictability, and plausibility. Our analyses of the overall corpus (i.e., all of the words) and target words replicated many previously reported findings (e.g., word-based frequency effects; Li, Bicknell, Liu, Wei, & Rayner, 2014), but also showed discrepancies between the corpus-based versus experimentally manipulated analyses (e.g., character-frequency effects in the latter but not the former). We will discuss the theoretical implications of our results for current and future models of Chinese reading (e.g., Reichle & Yu, 2017).

Older Adults Make Greater Use of Word Predictability in Chinese Reading

Sainan Zhao[1], Lin Li[1], Qianqian Xu[1], Jingxin Wang[1], Kevin B. Paterson[2]
1. *Tianjin Normal University, China*
2. *University of Leicester, UK*

An influential account of aging effects on reading holds that, compared to young adults (18-30 years), older adults (65+ years) make greater use of context to predict upcoming words (Rayner et al., 2006). However, supporting evidence is scarce. Accordingly, we conducted two eye movement experiments to investigate adult age differences in word predictability effects in Chinese reading, as this language has characteristics that may promote such effects. Experiment 1 used sentences containing a two-character target word with high or low cloze predictability. A predictability effect in reading times for words (emerging in gaze durations) was larger for the older adults. However, while both young and older adults skipped more predictable target words more often, this effect did not differ across age groups.
With Experiment 2, we used one-character target words to promote parafoveal processing and increase word-skipping. As in Experiment 1, predictability effects on reading times for words were greater for older adults. However, no interaction effect was observed in word-skipping, despite increased rates compared to Experiment 1. Together the findings provide novel evidence that older readers make greater use of context predictability to identify words, while casting doubt on their greater use of this information during parafoveal processing. We discuss our findings in the context of aging effects on reading in Chinese and alphabetic languages.

Reference

Rayner, K., Reichle, E.D., Stroud, M.J., Williams, C.C., & Pollatsek, A. (2006). *The effect of word frequency, word predictability, and font difficulty on the eye movements of young and older readers*. Psychology and Aging, 21, 448-465.

Saccade-Targeting is Not Word-Based in Chinese Reading: Evidence from Parafoveal Masking

Jingxin Wang[1],Min Chang[1], Kuo Zhang[2], Xue Zhang[1], Lin Li[1], Sha Li[3], Simon P. Liversedge[4], & Kevin Paterson[5]
1. *Tianjin Normal University, China.*
2. *Nankai University, China.*
3. *Fuzhou Normal University, China*
4. *University of Central Lancashire, UK.*
5. *University of Leicester, UK.*

Words in Chinese often comprise two or more adjacent characters althoughword boundaries are not delineated using spaces or visual cues. Whether Chinese readers make use of parafoveal word length information for eye-guidance is therefore unclear. On one view, this information is used flexibly so that, when parafoveal word length information is available, saccades are targeted towards the center of the next word so it can be recognized efficiently, often in a single fixation (Yan et al., 2010). However, when this information is unavailable, saccades are targeted towards the beginning of the next word, which then must be read by making multiple fixations. Another view holds that saccade-targeting is not word-based and that this pattern simply reflects aword recognition advantage when saccadeshappens to land at an optimal intra-word location (Li et al., 2011). The present study compared these accounts using a gaze-contingent paradigm in which words to the right of fixation were shown normally, or masked so that word length information was unavailable. Reading was slower when parafoveal information was masked. However, landing position effects for words receiving one or multiple first-pass fixations were identical in normal compared to masked displays, supporting the view that saccade-targeting is not word-based.

Li, X., Liu, P., & Rayner, K. (2011). *Eye movement guidance in Chinese reading: Is there a preferred viewing location?* Vision Research, 51, 1146-1156.
Yan, M., Kliegl, R., Richter, E.M., Nuthmann, A., & Shu, H. (2010). *Flexible saccade-target selection in Chinese reading*. Quarterly Journal of Experimental Psychology, 63, 705-725.

Lack of Character Frequency Effects in Reading Chinese Compound Words

Jukka Hyönä[1]; Lei Cui[2]; Jue Wang[2]; Yingliang Zhang[2]; Fengjiao Cong[2]; Wenxin Zhang[2],

1 *University of Turku, Finland*

2 *Shandong Normal University, China*

In two eye-tracking studies, reading of two-character Chinese compound words was examined. In both experiments, first and second character frequency were orthogonally manipulated to examine the extent to which Chinese compound words are processed via the compon ent characters. In Experiment 1, first and second character frequency were manipulated for compound words of relatively high in frequency, whereas in Experiment 2 it was done for infrequent compound words. Fixation time and skipping probability for the first and second character were affected by its frequency in neither experiment, nor in their pooled analysis. Lack of character frequency effects is unlikely to be a result of insufficient power, as we had in the pooled analysis 18400 observations per frequency condition. In Experiment 2 fixation durations on the second character showed longer fixation durations when a high frequency character was presented as the first character compared with when a low frequency character was presented as the first character. This result is explained by a constraint hypothesis, according to which fixation time on the second character was longer when the second character was semantically not closely related to the one constrained by the prior context. All in all, it is concluded that frequent Chinese compound words are processed holistically, whereas with infrequent compound words there is some room for the characters to play a role in the identification process.

Saccades (Room 5 | 10:30 – 12:30)

Saccadic adaptation to temporarily delayed stimuli

Ilja Wagner[1], Christian Wolf[2], & Alexander C. Schütz[1,3]
1 *Experimental & Biological Psychology, University of Marburg (Germany)*
2 *General Psychology, Westfaelische Wilhelms-Universität Münster (Germany)*
3 *Center for Mind, Brain and Behavior, University of Marburg (Germany)*

Saccadic adaptation is an adaptive learning mechanism that ensures saccade accuracy. Earlier studies demonstrated that saccades to perceptually-irrelevant stimuli are only adapted when the post-saccadic error occurs shortly after saccade offset (e.g. Shafer, Noto, & Fuchs, 2000). Here, we test the hypothesis that saccades to perceptually-relevant stimuli can be adapted even when the error occurs long after saccade offset. Participants were instructed to saccade to a perceptually-irrelevant saccade target that was displayed at a horizontal eccentricity of 10°. Two seconds after saccade offset, a Gabor patch appeared for 150 ms. The Gabor patch was shown randomly 4° above or below the center of the saccade target. Two seconds after Gabor offset, a reference stimulus was displayed at the saccade target position and participants had to judge if the orientation of the reference and the Gabor matched. Vertical saccade amplitudes showed robust trial-to-trial changes in the direction of the Gabor. The adaptation magnitude was comparable to a control experiment, in which the perceptually-relevant Gabor was shown for 150 ms directly after saccade onset.
Our findings demonstrate that saccades to perceptually-relevant stimuli can be adapted even when an error occurs long after saccade offset. Such a long temporal evaluation window might be necessary under natural conditions, when the saccade target is temporarily occluded after the saccade or when saccades guide and anticipate actions (Land & Hayhoe, 2001), whose consequences arise only later on.

The spatial programming of multiple saccadic eye movements

McSorley, Eugene ; McCloy, Rachel; Gilchrist, Iain
University of Reading; University of Reading; University of Bristol

We make multiple saccadic eye movements everyday but little work has taken place examining the control of these and it is unclear the extent to which the spatial aspects of the saccadic responses are programmed in parallel. In order to examine this we asked participants to saccade to multiple visual targets and, while they shifted their gaze around the display, we displaced select targets. We reasoned that visual targets closer to current fixation would be programmed to a greater depth and be less susceptible to changes in target locations than those further removed. In both experiments we found evidence of parallel programming of saccades with saccades being executed with shorter response latencies as more visual targets are made available for pre-programming. However this came with an accuracy cost showing that the new target position was factored into the programming of the saccadic response executed to all targets, even those which are the subject of the next fixation. We also found that having more information about the sequence path, hence the overall shape or Gestalt of the path, influenced saccade landing position with saccades to original target locations being less affected by relocations where there is less information about the sequence path. This suggests that while parallel programming may occur across multiple target locations, the spatial aspect of the underling visuo-motor programming of the saccadic response is still sensitive to changes made to future target locations.

Trans-saccadic perception of filled-in information

Alexander C. Schütz & Alejandro H. Gloriani
Allgemeine und Biologische Psychologie, Philipps-Universität Marburg, Marburg, Germany

A small star at the night sky can disappear from our vision when we fixate it directly, because the fovea does not contain rod photoreceptors. Recently, we showed that this scotopic foveal scotoma is filled-in with information from the immediate surround and that humans trust this inferred information more than veridical information from the periphery (Gloriani & Schütz, 2019). Here we investigate how this filled-in information is treated after a saccade when veridical peripheral information was available before the saccade in trans-saccadic perception. We presented gratings under scotopic conditions with independently oriented center and surround and observers had to discriminate the orientation of the center. We replicated the filling-in observation because observers were not able to discriminate the center in the fovea and consistently reported the surround orientation. In trans-saccadic conditions however, observers correctly reported the center orientation based on peripheral information and were not affected by the filled-in foveal information. Simulating scotomata under photopic conditions showed that perception was not influenced by filled-in information, except when the center was still identifiable as a separate object, distinct from the surround. In that case, observers showed a bias for the (false) information from the simulated foveal scotoma.
These results show that trans-saccadic perception and the weighting of peripheral and foveal information is not limited to mandatory low-level image fusion, but relies on a sophisticated matching of objects in peripheral and foveal information. This is reminiscent of causal inference in multisensory integration (Körding et al., 2007).

Acknowledgements: This work was supported by the European Research Council (ERC) under the European Union's Horizon 2020 Research and Innovation Program (grant agreement no. 676786).

Rapid spatial updating across saccades is malleable

Artem V. Belopolsky & Jonathan van Leeuwen
Department of Experimental and Applied Psychology, Vrije Universiteit, Amsterdam, The Netherlands

The human eye movement system is equipped with a sophisticated updating mechanism that can adjust for large retinal displacements produced by saccadic eye movements. Previous studies have demonstrated that updating can occur very rapidly and is initiated before the start of a saccade. In the present study we used saccade adaptation to demonstrate that rapid updating is also malleable. Participants made a sequence of horizontal and vertical saccades while ignoring an irrelevant distractor. Curvature of the second saccade relative to the distractor was used to estimate the time-course of updating. During the first saccade both saccade targets were displaced on 80% of trials, which induced shorter or longer saccades depending on a block of trials (Experiment 1). Critically, since the distractor was left stationary, successful saccade adaptation meant that on some trials after the first saccade distractor appeared in a different hemifield than expected. After adaptation, second saccades curved away only from the "new" distractor location starting at 80ms after the first saccade. When on the minority of trials (20%) the targets were not displaced, saccades again first curved away from the "new" expected location, but then quickly switched to curving away from the "old" location. When on some trials the distractor was removed after the first saccade, saccades curved away only from the "new" expected location (Experiment 2). The results show that updating of locations across saccades is not only fast, but is malleable, based on learned sensorimotor contingencies.

Transsaccadic perception depends on object-based updating

Tas, Caglar ; Hollingworth, Andrew
University of Tennessee - Knoxville; University of Iowa

The present study investigated mechanisms of transsaccadic object perception. Specifically, we asked whether the pre- and the post-saccadic representations of saccade targets are integrated or maintained separately. In addition, we tested the hypothesis that post-saccadic representations tend to overwrite pre-saccadic representations when transsaccadic continuity is achieved. Participants executed a saccade to a colored disk. During the saccade, the target's color was changed by 0°, 15°, 30°, or 45° in color space. Further, we manipulated object continuity by means of a target blanking manipulation (no post-saccadic blank or 200-ms blank). Participants either reported the pre- or post-saccadic color value using the continuous report paradigm. Response distributions were fit with probabilistic mixture models. There were two major findings. First, for color change trials, response distributions were generally fit better by a bimodal model than a unimodal model, indicating that there was minimal integration of color from pre- and post-saccadic sources. Second, when reporting the pre-saccadic color, we observed robust overwriting (incorrect report of the post-saccadic color value). This effect was most pronounced under conditions promoting object continuity (no blank, small color discrepancy) and was systematically reduced under conditions that should have disrupted object continuity (blank, larger color discrepancy). The results challenge recent evidence suggesting that color information is integrated across saccades, and they support an object-based model in which more recently encoded features of an object representation tend to overwrite previously encoded features.

Do readers integrate phonological codes across saccades? A Bayesian meta-analysis and a survey of the unpublished literature.

Martin R. Vasilev, Mark Yates, Timothy J. Slattery
Bournemouth University, Department of Psychology; University of South Alabama, Department of Psychology; Bournemouth University, Department of Psychology

It is commonly accepted that phonological codes can be activated parafoveally during reading and later used to aid foveal word recognition- a finding known as the phonological preview benefit. However, a closer look at the literature shows that this effect may be less consistent than what is sometimes believed. To determine the extent to which phonology is processed parafoveally, a Bayesian meta-analysis of 27 experiments and a survey of the unpublished literature were conducted. While the results were generally consistent with the phonological preview benefit (>90% probability of a true effect in gaze durations), the size of the effect was small. Readers of alphabetical languages obtained a modest benefit of only 4 ms in gaze durations. Interestingly, Chinese readers showed a larger effect (6-14 ms in size). There was no difference in the magnitude of the phonological preview benefit between homophone and pseudo-homophone previews, which suggests that the modest processing advantage is indeed related to the activation of phonological codes from the parafoveal word. Simulations revealed that the results are relatively robust to missing studies, although the effects may be 19-22% smaller if all missing studies found a null effect. The results suggest that while phonology can be processed parafoveally, this happens only to a limited extent. Because phonological priming effects in single-word recognition are small (10-13 ms; Rastle & Brysbaert, 2006) and there is a loss of visual acuity in the parafovea, it is argued that large phonological preview benefit effects may be unlikely.

Oculomotor Dysfunction I (Room 4 | 10:30 – 12:30)

Effect of eye movement reactivation on visual memory among individuals with traumatic brain injury (TBI)

Eli Vakil, Yishai Deitcher and Yaron Sachar
Bar Ilan University; Bar Ilan University; Loewenstein Rehabilitation Hospital

Previous research has shown that when individuals are asked questions referring to visual stimuli seen before, their eye movements spontaneously return to the visual area where the stimuli were first seen. This recurring eye movement phenomenon has been shown to assist the memory retrieval of visual images. It is thus possible that oculomotor dysfunction can account for visual memory deficits, due to their diminished tendency to retrieve the same eye movement pattern. Considering patients with traumatic brain injury (TBI) characteristically suffer from visual memory deficits, also typical among this population, can partially account for this memory impairment.
In this study, 27 healthy individuals and 27 patients with TBI from the Lowenstein Rehabilitation Hospital participated in a memory task. Participants were first exposed to stimuli and were then asked questions about the displayed stimuli. The testing session was conducted for each participant under two conditions: (1) while eyes were free to move over the screen; (2) while eyes were fixated.
Study findings show that the control group significantly benefitted from the free-viewing condition in comparison to the fix-viewing condition, while this effect was absent among the TBI group. This was corroborated by the eye tracking data showing that participants with TBI showed a minimal tendency to effectively reactivate eye movements as among the healthy group. The research findings expand our comprehension of visual memory among the TBI population and present rehabilitation health clinicians with new directions in understanding visual memory deficits.

Fixation dynamics as a function of PRL eccentricity with a simulated-scotoma

Francisco M Costela[1,2]; Susana Martinez-Conde[3]; Russell L Woods[1,2]

1. *Department of Ophthalmology, Harvard Medical School, Boston, MA, United States.*
2. *Schepens Eye Research Institute, Boston, MA, United States.*
3. *State University of New York (SUNY)*

When foveas are damaged by retinal disease, people often adopt an alternative preferred retinal locus (PRL) to conduct everyday visual and visuomotor tasks. Individuals with normal vision can be trained to develop a PRL over a relatively short time period, via the use of a simulated foveal scotoma. We found that, as the simulated scotoma size increased, the PRL's location with respect to the fovea remained largely unchanged. We also wondered about microsaccade production as a function of PRL eccentricity. Microsaccades are involuntary, small-magnitude saccadic eye movements made during attempted visual fixation. We expected that microsaccade size would vary with PRL eccentricity. Moreover, we hypothesized that, instead of being mostly horizontal (as during normal vision), microsaccades produced with a PRL would be oriented towards the location of the former fovea. We trained eight subjects with normal vision to develop a PRL, by having them produce saccades, fixations, and smooth pursuits while exposed to a simulated foveal scotoma of 4˚ diameter. Subsequently, subjects were exposed to scotoma sizes ranging from 4˚ to 24˚ diameter. During fixation tasks, microsaccades got smaller with increasing scotoma sizes. Conversely, saccadic intrusions got larger and more frequent as a function of scotoma size. In addition, some subjects showed a moderate microsaccade direction bias towards the location of the former fovea, suggesting that the oculomotor system did not (and may not ever) completely reference itself to the PRL, despite showing substantial flexibility in adapting to the expansion of the scotoma size during fixation.

Reading with a simulated central vision loss: a comparison of dynamic and static text formats.

Farah Akthar and Robin Walker
Department of Psychology
Royal Holloway, University of London, UK

Background: Dynamic text formats, such as horizontally scrolling and rapid serial visual presentation (or RSVP) have been recommended as methods to enhance reading in people with macular degeneration and central vision loss (Harvey and Walker, 2014; Chung, 2011). Here, we examined reading performance using an artificial scotoma paradigm to simulate central vision loss.
Method: Fifteen participants read passages of text presented as: single horizontally-scrolling lines, single word rapid serial visual presentation (RSVP), single static lines and paragraph format. An Eyelink 1000 eye-tracker controlled a gaze-contingent central scotoma. The main measures of interest were reading comprehension, accuracy and eye movements (adherence to the eccentric viewing (EV) position).
Results: Reading performance (comprehension and accuracy) was significantly better with single horizontally-scrolling lines and poorest with RSVP. The paragraph format yielded better comprehension than single static lines, but accuracy was reduced. Although participants showed better adherence to maintaining an EV position during reading with RSVP, this did not result in improvements in their comprehension. An examination of eye-movement data indicated a difference of strategies adopted whilst reading with each text display, which may account for the observed differences in reading performance across conditions.
Significance: This study highlights the potential benefits of using scrolling text to enhance the ability to maintain adaptive strategies and improve reading performance in people with a central vision loss.

Chung, S.(2011). *Improving reading speed for people with central vision loss through perceptual learning*.IOVS, 52(2).
Harvey H, Walker R(2014). *Reading with peripheral vision: a comparison of reading dynamic scrolling and static text with a simulated central scotoma*. Vision Res.

Rapid adaptation of fixation distribution during sentence reading with central field loss and its role in reading performance : a mediation analysis

Castet, Éric ; Aguilar, Carlos; Matonti, Frédéric; Calabrèse, Aurélie
CNRS-AMU : LPC UMR 7290 Marseilles; CNRS-UNS : BCL UMR 7320 Nice; Monticelli-Paradis Ophthalmology Center : Marseilles; CNRS-AMU : LPC UMR 7290 Marseilles

Age-related Macular Degeneration patients (288 million people worldwide by 2040) are blind in their central visual field and have thus great difficulty to read text. Reading with central field loss (CFL) relies on peripheral vision and requires adaptation of visuo-attentional and oculo-motor processes. It is still an open question whether reading performance depends on the systematic (or at least frequent) use of one specific portion of peripheral vision (often referred to as the Preferred Retinal Locus - PRL). To address this question, forty-four normally-sighted subjects read French sentences with a gaze-contingent artificial scotoma (a 10° disk centred on the fovea). They had to read with the “self-paced reading” technique allowing to display only one word (N) at a time while masking all other words with ‘x’ strings: in order to read, subjects had to unmask the adjacent word (N+1 or N-1) by using keyboard presses. Several experimental blocks were run over a period of about 2 hours. For each subject and block, a fixation heatmap was created by plotting fixation locations with respect to each word’s centre. Reading speed was regressed on several characteristics of fixation maps (centroid, size, ….). Results show that reading speed correlates with the vertical coordinate of the distribution’s centroid. Importantly, a statistical mediation analysis shows that improvement in reading speed between the first and the last block is partly mediated by this vertical coordinate. Consequences of these results for designing novel visual rehabilitation protocols are discussed.

Cerebellar control of saccades by the size of the active population in the caudal fastigial nucleus

Laurent Goffart, Julie Quinet and Clara Bourrelly
Aix Marseille University, CNRS, INT, Marseille, France
contact: laurent.goffart.int@gmail.com

The caudal fastigial nucleus (cFN) plays a crucial role in the ability to foveate a static or moving visual target. Its unilateral pharmacological inactivation impairs the horizontal velocity of pursuit eye movements and the horizontal amplitude of saccades. After cFN inactivation, more endpoint variability than normal saccades has been reported and interpreted as an altered planning and/or signal dependent motor noise (Eggert et al. 2015). From the demonstration that larger current enhances the size and velocity of saccades evoked by cFN microstimulation (Quinet & Goffart 2015), we propose that the variability of saccade endpoints actually reflects the variable size of the active population. Indeed, if the muscimol injection is not exactly centered in cFN, the number of neurons which are inactivated should increase as the inhibitory drug diffuses, resulting in a dysmetria that increases with time. We will show examples of cFN inactivation experiments performed in head-restrained monkeys where the size of dysmetria does not change with time, and other examples where it increases with time. Thus, the variability of saccade amplitude seems to be the consequence of the number of cFN neurons which are involved in saccade execution. More generally, our observations support the hypothesis that the cerebellar control of the ability of foveate and pursue a visual target consists of adjusting the number of active neurons in the cFN and their firing rate.

Impacts of heterophoria on binocular advantages in reading

Stephanie Jainta
University of Applied Sciences and Arts Northwestern Switzerland, Institute of Optometry

Reading with two eyes necessitates efficient processes of binocular vision, which provide a single percept of the text. These processes come with a binocular advantage: binocular reading shows shorter average fixation durations and sentence reading times when compared to monocular reading. In Jainta and Jaschinski (IOVS; 2012; N = 13), we showed that binocular advantages critically relate to the individual heterophoria (the resting state of vergence) and in the present, large-scale replication we set out to quantify heterophoria impacts on binocular advantages in reading. We collected binocular eye movements (Eyelink II) for 94 participants who read 20 sentences monocularly and 20 sentences binocularly. Further, individual heterophorias were determined using three different, optometric standards: objective eye tracking (EyeLink II at 60 cm), Maddox wing test (at 30 cm) and MKH measures (at 5.5 m). Binocular eye movements showed typical pattern in monocular reading (larger saccades and changed binocular coordination) and impacts of increased heterophoria on binocular coordination in binocular reading. More importantly, we replicated (1) binocular advantages of about 25 ms for average fixation durations and (2) a reduction in binocular advantages when heterophoria increased. The latter was true only when heterophoria was identified by EyeLink II or Maddox wing measures (MKH measures of heterophoria did not affect binocular advantages in reading). In detail, for large heterophorias (3 degrees and larger) binocular reading even turned into a disadvantage. Implications for effect estimations through LME modelling and optometric treatments based on different heterophoria measures will be discussed.

Session 5

Attention II (Plenary room | 14:00 – 16:00)

Eye movements and actions during typing with an on-screen keyboard

Foulsham, Tom
University of Essex

In real-world tasks, eye movements and attention are deployed as part of a sequence of actions. However, tracking gaze during naturalistic actions remains complex and methodologically difficult. I will describe three experiments that use a common, realistic task – typing with an on-screen keyboard – to investigate the coordination between eye and hand movements. Participants selected keys with a mouse in order to copy text, name pictures, or in response to a spoken word. Eye fixations preceded typing actions in a systematic way, across all tasks, showing that participants tended to look ahead in the sequence of required key presses. These look-aheads reveal sequential planning of the individual letters making up the required output. I will discuss other effects on the eye-mouse span in this task, as well as the effect of word complexity on looks back to the stimulus. There is a long history of studying eye movements in copytyping. The present findings demonstrate that studying attentional deployment over an on-screen keyboard can reveal much about sequential tasks as well as about theories of language production.

Changes in strategies during mouse- and gaze-foraging are associated with variations in oculomotor dynamics

Jérôme Tagu & Árni Kristjánsson
Icelandic Vision Laboratory, School of Health Sciences, University of Iceland

In foraging tasks where observers select targets by tapping them with their fingers, observers change their strategy according to the crypticity of the targets. During feature-based foraging involving two target types (e.g., red and green dots) among two distractor types (e.g., yellow and blue dots), where crypticity is low, observers select the two target types in random order. However, in a more cryptic conjunction-based task (e.g., green dot and red square targets among red dot and green square distractors), observers mainly select targets in two long runs, finishing one entire target category before turning to the second. But some observers forage randomly even during conjunction-foraging (so-called "super-foragers"). We recorded eye movements of observers during foraging where they cancelled items either with mouse-click or eye-fixation (mouse- or gaze-foraging), testing whether oculomotor dynamics would vary according to the foraging strategy and the effector type that is used. The behavioral and eye movement data indicate that the ability of "super-foragers" to switch between the two target types in conjunction-search is linked to 1) anticipating the next target and 2) organization of the visual exploration. We report intra- and inter-individual differences in foraging strategies and eye movement dynamics between mouse- and gaze-foraging and between feature-based and conjunction-based foraging. Our novel gaze-foraging paradigm, along with the finger-foraging task, provides important insights into how observers orient in complex visual search tasks and about optimal strategies for efficient foraging.

Adding Informational Masking against Energetic Masking further Reduces the Accuracy and Speed of Visual-Search Performance: An Eye Movement Study

Haibo Yang[1] **Lili Niu**[1] **Liang Li**[2]
1 *Academy of Psychology and Behavior，Tianjin Normal University*
2 *School of Psychological and Cognitive Sciences, Peking University*

Visual search for targets becomes difficult when targets are surrounded by masking objects. To investigate the mechanisms underlying the informational masking, in this study both the performance accuracy to determine whether one of four lettersdiffered from the other and the eye movements during the performance were examined when the target letters were masked by one of the 4 types of maskers: randomly positioned and oriented letters, letter fragments, random-phase noise, and random-pixel noise. The results showed that the search performance was poorer and the eye-fixation/gazing time was longer when the masker was composed of recognizable objects (letters or letter fragments) than when it was either random-phase or random-pixel noise, indicating that when the masker became informational it interfered more with the visual search and induced more perceptual loads than the energetic noise maskers with the same spatial frequency profile or contrast. Moreover, performance was poorer and the total fixation time was longer for the letter masker than for the letter-fragment masker, suggesting that the letter masker, which elicits the lexical activity at higher-order processing stages, interferes more with the visual search and induces more perceptual loads than the letter-fragment masker, which elicits the graphemic activity. Thus, the lower accuracy and speed of visual-search performance under informational masking are caused by an increase in perceptual load, demanding longer eye-fixing/gazing times.

Gao,Y.Y., Schneider, B., &Liang Li. (2017). The effects of the binocular disparity differences between targets and maskers on visual Search. *Attention, Perception,& Psychophysics. 79*:459-472.

What determines where we look: The individual differences approach

Pertzov, Yoni; Guy, Nitzan
Psychology department The Hebrew Universit

Vision is not a passive process but rather an active process in which our high-resolution center of gaze is repeatedly shifted. People often differ in the way they perceive and explore the world around them. Indeed, when two observers view the same picture, they typically study the image following different scan paths. When seeing the same image again, individuals repeat their specific scanning characteristics in a consistent manner. Most previous studies have treated these individual differences as noise and studied the common factors of gaze behavior by pooling the data across observers. Here, I posit that these reliable individual differences are of great interest. I will describe evidence that such difference reflect variability between observers in their visual processing skills (e.g. faster processing leads to shorter fixations and a higher rate of saccades). Moreover, differences in gaze behavior reflect also variation in high-level factors between people (interest in certain visual categories may differ between subjects. The importance of this research derives not only from the need to modify an influential theoretical framework and understand the almost uncharted territory of individual differences in gaze behavior. It also stems from the considerable influence of gaze behavior on ones attitudes and behavior. Our gaze behavior characteristics determine the visual input that enters our brain and, in turn, our memories, cognition and traits. Thus, consistent differences in gaze behavior may not only reflect our traits but also lead to consistent differences in how we perceive and understand the world.

Learning Goal-directed Fixations

Gregory Zelinsky, Yupei Chen, and Seoyoung Ahn
Stony Brook University, USA

Most models of attention control predict fixations from priority signals learned either via supervised training with an object category or directly from the to-be-predicted behavior. However, neither object labels nor previous behavior is typically available for training, making neither method a realistic model for attention control. Recently, we introduced ATTNet, a deep network that uses reinforcement learning (RL) to predict the fixations made during the search for a target category goal. ATTNet also has rough biological plausibility in that it has a foveated retina and an architecture that broadly maps onto known structures in the primate brain's ATTention Network. Through the application of reward upon fixating a target category in an image, ATTNet learns to shift its fovea to locations that maximize expected reward—essentially, learning to prioritize space. We tested ATTNet using separate "microwave oven" and "analog clock" search tasks. Images were kitchen scenes (Microsoft COCO) depicting both a microwave and a clock (target present) or no instance of the target category (target absent). Any difference in the model's behavior could therefore only be due to the target-specific learning of reward. Similar to the eye movements observed from 60 participants searching the same scenes for the same targets, ATTNet preferentially fixated only the rewarded object category. Analysis of target-absent behavior also revealed clear scene context effects. There is now computational evidence that the fixations made during goal-directed attention control can be modeled simply as the pursuit of expected reward using movements of a fovea.

Unlike saccades, quick phases of optokinetic nystagmus are not preceded by shifts of attention

Nina M. Hanning[1,2] and Heiner Deubel[1]
1 Ludwig-Maximilians-Universität München, Munich, Germany;
2 Graduate School of Systemic Neurosciences, Munich, Germany
hanning.nina@gmail.com

Quick phases of optokinetic nystagmus are often equated to saccades. Indeed, their trajectories resemble the saccadic 'main sequence', and both saccade and quick phase control involve the same brainstem circuitry. However, whether and to what extend higher cortical areas associated with saccade programming (e.g. lateral intraparietal area, frontal eye fields) also participate in the control of OKN quick phases remains unknown. One hallmark of saccadic eye movements is the premotor shift of attention towards the saccade target, which is assumed to rely on overlapping networks for attention and oculomotor control in these higher cortical areas. Hence, the question arises if quick phases, like saccades, also draw attention towards their endpoint.
We measured the spatiotemporal distribution of visual attention during OKN induced by a leftward or rightward moving full-field dynamic pink noise stimulus. At a random time and position, the moving noise stimulus contained a brief (50 ms) orientation signal which participants had to discriminate. Taking visual orientation sensitivity as a proxy of attention, this paradigm allowed us to determine how attention is deployed during quick and slow phases of OKN, without the presence of object-like visual structures.
Our results show that visual attention is not enhanced at the endpoint of OKN quick phases. Instead, visual attention during OKN is predominantly allocated to the current gaze position. This suggests that OKN quick phases are reflexive gaze resetting movements controlled by low level rather than higher, attention-related centers. OKN quick phases are not saccades.

Parafoveal Preview (Room 10 | 14:00 – 16:00)

Parafoveal Processing of Repeated Words during Reading

Denis Drieghe & Rob Chan Seem
School of Psychology, University of Southampton

Raney and Rayner (1995) investigated the effects of lexical repetition on High Frequency (HF) and Low Frequency (LF) target words in passages of text. The initial presentation of target words showed that there was a frequency effect such that HF were read faster than LF words but repetition attenuated fixation durations and the size of the frequency effect over presentations. By the third presentation a floor effect emerged where repetition exerted no further effect over fixation durations and frequency effects were absent. In the current study we implemented a display change on the target words such that they were either presented correctly or misspelled before the eyes landed on them. We observed significant main effects of frequency (HF words were read faster than LF words), preview (reading times were faster on the target word when the preview was correct compared to misspelled) and repetition (words were read faster as they were encountered more often). The interaction between frequency and repetition observed by Raney and Rayner (1995) was replicated such that frequency effects were reduced with repeated encounters. Our analysis showed that this was almost exclusively due to faster reading times of the LF target word when repeated. Crucially, there was no interaction between preview and repetition indicating that parafoveal processing was not influenced by repetition. Consistent with this conclusion, skipping ratios which are also influenced by parafoveal processing did not show any interactions with preview.

The use of parafoveal syllable information for early Finnish readers

Häikiö, Tuomo ; Mäkinen, Tiina
University of Turku

It has been established that early Finnish readers utilize syllable information in the foveally fixated word. However, when the syllable information is explicitly cued by hyphens at syllable boundaries (e.g., en-ter), reading is slowed down due to attention being drawn to single syllables at a time. In the present study, we were interested in whether early Finnish readers extract syllable information from the parafoveally viewed word. Furthermore, we assessed whether seeing explicit syllable structure information only in the parafovea can be used to jump start word processing once the word in question is fixated. To this extent, 1st and 2nd grade Finnish children read sentences with an embedded critical target word while their eye movements were recorded. The target word either contained or did not contain a hyphen denoting the syllable structure (e.g., en-ter). The preview type (hyphen vs. no hyphen) and the display change (change, hyphen to no hyphen or vice versa, vs. no change) were manipulated. The results showed that both 1st and 2nd grade children extracted syllable information from the parafovea. However, seeing explicit syllable structure information only in the parafoveal word did not give rise to enhanced word processing as the cost associated with the display change ate up the facilitating effect of the explicit syllable boundary. In sum, our study shows that early readers in a shallow orthography allocate their attention also to the upcoming word and that they extract sublexical parafoveal information.

Effects of reading goals on parafoveal processing during reading

White, S.J.[1], Warrington, K.L. [1], Sidhu, K. [2], McGowan, V.A. [1], Soltan, M. [3], Gopikrishna, D. [1], Kolodziejczyk, K.Z. [4] & Paterson, K.B. [1]

1 *University of Leicester*
2 *NHS East Midlands Deanery*
3 *NHS West Midlands Deanery*
4 *Leiden University*

Two experiments employed the moving window gaze contingent change technique (McConkie & Rayner, 1975) to assess how reading goals (reading vs. skimming) modulate parafoveal processing. Single sentences were presented with either no mask, or all words masked except for the fixated word, the fixated word and one word to the right, or the fixated word and two words to the right. Outside of the moving window words were replaced with visually similar letters. In both experiments there were interactions between reading goal and window size for first-pass reading measures. Parafoveal preview benefit for fixated words is likely to be limited during skimming as longer saccades result in more eccentric (visually degraded) previews. Window size also significantly affected skipping rates, with fewer skips when parafoveal words were masked. Crucially, for Experiment 1 (simple sentences) there were interactions between reading goal and window size for reading rate, such that accurate parafoveal previews were especially important in maintaining reading rate for skimming compared to reading for comprehension. Accurate parafoveal previews enable high skipping rates, which in turn enable high reading rates for skimming. There was no reliable interaction for reading rate in Experiment 2 (sentences with more complex concepts), likely because skipping and reading rates were lower for these more difficult sentences. The implications for how eye movement control mechanisms may be modulated by reading goals will be discussed. Comparisons with between-participant examinations of effects of reading speed on the perceptual span will also be considered (Rayner, Slattery & Bélanger, 2010).

References

McConkie, G. W., & Rayner, K. (1975). The span of the effective stimulus during a fixation in reading. *Perception & Psychophysics*, 17, 578-586.
Rayner, K., Slattery, T.J., & Bélanger, N.N. (2010). Eye movements, the perceptual span, and reading speed. *Psychonomic Bulletin & Review*, 17, 834-839.

The impact of uninformative parafoveal masks on L1 and late L2 speakers

Fernandez, Leigh B. ; Scheepers, Christoph; Allen, Shanley E.M.
Technische Universität Kaiserslautern; University of Glasgow; Technische Universität Kaiserslautern

Reading research has found an orthographic, phonological, morphological, and (potentially) semantic parafoveal preview benefit (Schotter et al., 2012). However it has been argued that invalid parafoveal previews lead to preview costs that interfere with reading (Hutzler et al., 2013; Vasilev & Angele, 2017). In the current study we manipulated the type of preview using a gaze contingent boundary paradigm. Native English speaking participants (n=51) and late L2 English speakers (L1 German; n=51) read sentences with the preview of the critical word (rock) manipulated such that an invalid preview (that shared no features with the critical word) was previewed and changed to the critical word upon making a saccade across an invisible boundary (*), (Table 1). The identical form served as the baseline and was compared to the invalid previews, and sliding contrasts were used. English and German non-word masks were used to test whether participants were sensitive to language specific sub-lexical orthographic information and were based on two language specific orthographic-measures (Schröter et al., 2016). First-fixation and total duration were greater for non-word masks relative to the identical mask, but there were no differences across language groups or due to sub-lexical orthographic information. For total duration, t-values increased (p-values decreased, all <0.001) as the mask became less word-like (ENW: t=4.20, GNW: t=4.26, Illegal: t=5.04, X: t=9.05, Blank: t=11.63; Figure 1). This suggests that L1 and L2 speakers are similarly impacted by uninformative masks, and that the less word-like the masks become, the greater the impact when foveally fixated.

Table 1: Example stimuli

	Identical	English non-word (ENW)	German X	Illegal	X	Blank
The geologist found th*e	rock	mish	mand	nbpl	xxxx	

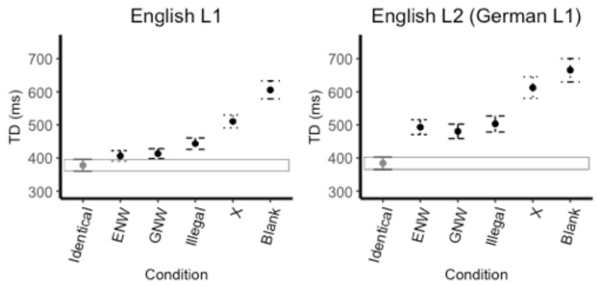

Figure 1. Total duration across parafoveal mask types (error bars indicate 95% confidence intervals; box encompasses the confidence interval of the identical condition).

Hutzler, F., et al. (2013). Parafoveal X-masks interfere with foveal word recognition: Evidence from fixation-related brain potentials. *Frontiers in Systems Neuroscience*, 7, 1-10.
Schotter, E. et al. (2012). Parafoveal processing in reading. *Attention, Perception, & Psychophysics*, 74, 5–35.
Schröter, P. & Schroeder, S. (2018). Exploring early language detection in balanced bilingual children: The impact of language-specificity on cross-linguistic nonword recognition. *International Journal of Bilingualism, 22*, 305-315.
Vasilev, R. M., & Angele, B. (2017). Parafoveal preview effects from word N+1 and word N+2 during reading: A critical review and Bayesian meta-analysis. *Psychonomic Bulletin and Review*, 24, 666-689.

Eye movements in the parafoveal processing of noun phrases: A return to the word group hypothesis

Ralph Radach, Laura Schwalm & Albrecht Inhoff
Bergische Universität Wuppertal, Department of Psychology

There has been substantial research on the extrafoveal processing of words located two positions right of the current fixation. From a recent meta-analysis of studies on such N+2 effects, it is apparent that there is a lack of work on syntactical parafoveal processing (Vasilev & Angele, 2017). Also, there is clearly a need for more experiments with systematic variation of N+1 word length. The present work sought to addresses both issues. We asked participants to read German sentences with nominal phrases like the following: ‘gibt die/das Sache’ and ‘sieht der/die rote Figur’. Articles were always a syntactically correct continuation of the sentence. However, an invisible boundary before the articleserved to create a mismatch so that the article could be incorrect with respect to grammatical gender of the following noun. Results indicate robust syntactical N+2 preview effects, but only marginal N+3 preview effects when the sentence contained an additional adjective between adjective and noun.

Interestingly, the noun phrase appeared to provide a unified target region for the incoming saccade, providing support for the word group hypothesis (Radach 1996, Drieghe, Pollatsek, Staub & Rayner, 2008). When the eyes reached the noun phrase, viewing times were a function of fixation positions within individual words. Apparently, word groups may provide a unified visual target, but the meaning-carrying article is being processed as an independent linguistic unit. Implications for theories of eye movement control in reading will be discussed with an eye on subtle differences between noun phrases in English and German.

Parafoveal Pre-processing in English Children: The Importance of External Letters

Sara V. Milledge, Hazel, I. Blythe, and Simon P. Liversedge
University of Southampton; University of Southampton; University of Central Lancashire

Although previous research has demonstrated that for adults external letters of words are more important than internal letters for lexical processing during reading, no comparable research has been conducted with children. This experiment explored, using the boundary paradigm during silent sentence reading, whether parafoveal pre-processing in English is more affected by the manipulation of external letters or internal letters, and whether this differs between skilled adult and beginner child readers. Six previews were generated: identity (e.g., monkey); the beginning three letters of the word substituted (external orthographic manipulation; e.g., rackey); the middle three letters of the word substituted (internal orthographic and phonological manipulations; e.g., machey, mochiy); the end three letters of the word substituted (external orthographic manipulation; e.g., monhig); and a control condition (e.g., rachig). Results indicate that both adults and children undertook pre-processing of words in their entirety in the parafovea, and that the manipulation of external letters in preview was more harmful to participants' parafoveal pre-processing than internal letters. These results not only provide further evidence for the importance of external letters to parafoveal processing and lexical identification for adults, but also demonstrate that such findings can be extended to children.

Detection and Gaze (Room 5 | 14:00 – 16:00)

Can word processing difficulty explain individual differences in inconsistency detection?

Annina Hessel, Sascha Schroeder
Department of Educational Psychology, University of Goettingen

Readers differ in their discourse processing skills, as evidenced for example in varying abilities to re-analyse inconsistent information (Connor et al., 2015). Arguably, such individual differences result from word processing difficulties (Perfetti & Stafura, 2014), especially in second language readers (Hopp, 2016). So far, however, this relationship has not been tested experimentally.
We thus conducted an eye-tracking experiment with a within-subject manipulation of inconsistency crossed with word processing difficulty, manipulated on the text level. We wanted to see whether and how difficulty influences re-analysis of inconsistencies in German learners of English as a foreign language (EFL). We predicted that when reading texts containing difficult words, EFL readers would adapt their rereading less clearly to inconsistencies than when reading texts containing easier words, indicating reduced re-analysis of inconsistent information.
Sixty-three adult EFL learners read 48 short expository texts containing inconsistencies created through mismatches between pre-targets such as sugar and targets such as salt, or consistent controls containing matched pre-target and target. Word processing difficulty was manipulated by inserting either shorter and higher-frequency words such as wait, or longer and lower-frequency words such as linger.
Preliminary results show that EFL learners read inconsistent targets and difficult words longer than consistent targets and easier words. Furthermore, higher word processing difficulty reduced inconsistency re-analysis during rereading, as evidenced in smaller adaptions to inconsistencies in texts containing difficult words.
This result shows that word processing difficulty reduces inconsistency re-analysis similarly to individual differences in language proficiency, but only during later processing. The finding will be discussed regarding theories of individual differences in discourse processing.

Compensation of ceiling effects through eye tracking

Fehringer, Benedict C.O.F. ; Münzer, Stefan
University of Mannheim

Ceiling and floor effects of items in performance testing occur if these items are solved by all participants or by none of the participants. In such cases, the items are not able to differentiate between participants with different abilities. Gaze patterns of participants obtained with eye tracking techniques are an additional source of information for visual items. The present study (N = 48) tested the potential to differentiate between participants by using simple fixation-based measures (number of fixations) as well as complex measures (parameters of a Hidden Markov Model, HMM) with a self-developed spatial thinking test. The items of this test are grouped into six distinct difficulty levels. Preliminary studies found that the easiest and the most difficult level produce ceiling and floor effects for participants from the tested population. Ability was indicated by the score of the complete test. The results showed for the easiest level that a certain parameter of the analyzed HMM (2-state HMM, one per level and participant) was able to differentiate between participants with high and low ability. The parameter significantly increased the explained variance in a hierarchical regression model over and above accuracy and reaction times. The parameter might be interpreted as an indicator for the participant's awareness of the item's structure. It is concluded that eye-tracking-based measures are able to deliver diagnostic information beyond accuracy and reaction times.

Event Detection Algorithms and Data Quality

Dare, Zoya ; Brinkman, Hanna; Mikuni, Jan; Kawabata, Hideaki; Leder, Helmut; Rosenberg, Raphael
University of Vienna; University of Vienna; Keio University; Keio University; University of Vienna; University of Vienna

Eye Tracking research is heavily dependent on event detection algorithms. There are numerous algorithms, using different approaches, and they partly produce very diverse results. While it is well documented that different algorithms can produce dramatically different results (see, for example, Nystrum and Holmqvist, 2011), often, little attention is paid to algorithms in research, with most studies conducted using the proprietary algorithm packaged with the eye tracker. This makes eye tracking research difficult to compare and reliably replicate.
This talk will present a comparison of various event detection algorithms used in regard to still scene viewing. We used data from a study about the cultural influences on art perception, performed by the Lab for Cognitive Research in Art History at the University of Vienna and the Aesthetic Science Laboratory at the Keio University (Tokyo). The aim of the study was the comparison of an Austrian and a Japanese group. However, it turned out that the results of the comparison are highly dependent on the event detection algorithm. We therefore compared one algorithm by Marcus Nystrum and Kenneth Holmqvist (Nyström and Holmqvist 2010) and two by researchers at the Perception Engineering Group at the University of Tubingen (Tafaj et al., 2012) based on our own research and previous studies done on algorithm comparison (Anderson et al, 2017). The talk will address methods of comparison, qualities of each algorithm, and the drastic differences in data they produced.

Andersson, R., Larsson, L., Holmqvist, K. et al. Behav Res. (2017) “One algorithm to rule them all? An evaluation and discussion of ten eye movement event-detection algorithms.” *Behavior Research Methods 2*, 49: 616–637.
Nyström, Macrus and Kenneth Holmqvist. Eye Tracking: A comprehensive guide to methods and measures. Oxford: *Oxford University Press*, 2011.
Nyström, Macrus and Kenneth Holmqvist (2010) “An adaptive algorithm for fixation, saccade, and glissade detection in eyetracking data” *Behavior Research Methods 1*, 42: 188-204.
Tafaj, Enkelejda, Gjergji Kasneci, Wolfgang Rosenstiel, Martin Bogdan. “Bayesian Online Clustering of Eye-Tracking Data.” *Eye Tracking Research and Applications (ETRA 2012)*: 285-288.

Improving the state of the art in eye movement event detection via trainable label correction

Startsev, Mikhail; Dorr, Michael
Technical University of Munich

Modern computational methods such as machine learning can improve eye movement detection. Several deep learning approaches have recently emerged that further pushed the boundaries of algorithmic classification. To tackle the detector quality assessment not just as a numerical problem, but to rather estimate how suitable the detected events are for subsequent analyses, event-level instead of sample-level evaluation metrics have been developed. In order to filter out unlikely or impossible events, current algorithms often rely on hand-crafted heuristics. In contrast to this, we propose a post-processing step to automatically learn the constraints via simple statistical modelling with hidden Markov models. Our approach learns from data to correct errors in algorithmic labelling, automatically adapting to the analysed labels. We tested this approach on large-scale data sets of free-viewing gaze data from dynamic natural scenes and in application to the outputs of a number of classical and state-of-the-art eye movement classifiers. Even though these performed very well on sample-level classification already, event-level metrics were noticeably improved. For example, we considered a state-of-the-art sequence-modelling deep learning eye movement classifier (Startsev et al., BRM, 2018) that already used a large temporal context. In principle, it thus could learn sequential inter-dependencies in the labels directly. However, our trainable correction yielded noticeable improvements in all event-level quality measures for all eye movement types: F1 scores, for instance, showed an absolute increase of 1.5%, 3%, and 5% for fixations, saccades, and smooth pursuits, respectively. The implementation is publicly available at https://github.com/MikhailStartsev/hmm_error_correction/.

Reading pie-graphs: the influence of top down and bottom up information in multimodal comprehension

Dos Santos Rodrigues, Erica ; Carvalho Ribeiro, Antonio João; Fernandes, Natália

Pontifical Catholic University of Rio de Janeiro - PUC-Rio; Fundação de Apoio à Escola Técnica - FAETEC- RJ; Pontifical Catholic University of Rio de Janeiro - PUC-Rio

The comprehension of graph-text constellations involves the integration of linguistic and visual information (Acartürk & Habel, 2012; Rodrigues, Fragoso & Ribeiro, 2018). We conducted a 2x2 within-subjects eyetracking experiment, with 20 undergraduate students, in order to investigate the role of the title and the visual display during the reading of pie-graphs. The independent variables were (i) type of title: directive title, which emphasizes the largest slice of the pie (The alcoholic beverage most consumed by adolescents is beer), vs. non-directive title, which does not refer to any particular sector of the graph (Alcoholic beverages most consumed by adolescents), and (ii) the position of the legend: legend to the right vs. legend to the left of the pie-graph. Eye movements were recorded by means of a Tobii Pro X3-120 eyetracker and the participants saw a total of 32 trials: 16 targets (pie-graphs) and 16 fillers (bar and line graphs). The total fixation time, the number of fixations, the average fixation duration and the number of visits were analysed for 3 areas of interest (AOIs): the title, the graph and the legend. There was significant effect of the title only when the legend was to the left of the graph, with directive titles generating longer fixations in the area of the graph. Regarding the position of the legend, left position generated higher values for number of fixations and total fixation time in the 3 AOIs. Top-down and bottom up processing costs in multimodal comprehension are discussed in the light of these results.

Session 6

Wednesday, August 21st

Word Recognition (Room 5 | 10:30 – 12:30)

Linguistic variables modulate where the eyes move regardless of word boundaries: Evidence against word-based eye-movement guidance during reading.

Françoise Vitu[1], Claire Albrengues[2], Frédéric Lavigne[2], Carlos Aguilar[3], Eric Castet[1]
1 Aix-Marseille Univ, CNRS, LPC (Laboratoire de Psychologie Cognitive), Fédération de Recherche 3C
2 Université de Nice Sophia-Antipolis, CNRS, BCL (Bases, Corpus, Langage)
3 Amaris Research Unit, Biot

It has long been assumed that saccadic eye movements during reading are driven in a top-down word-based manner. According to this hypothesis, saccades are invariably programmed towards the center of peripheral target words, as selected based on the (expected) needs of ongoing processing, while the variability in within-word landing positions exclusively reflects systematic and random errors. Here we challenged the word-based view by testing one of its strong predictions: that linguistic variables should only influence the likelihood of word skipping, but not where in the words the eyes land. Using a large corpus of eye-movement data collected while forty native-French speakers read a total of 316 pairs of sentences, we showed that the frequency and the predictability of words only mildly affected eye-movement behavior in comparison with word length and saccadic launch-site distance to the words' beginning. Yet, frequency and predictability overall influenced where the eyes initially landed regardless of word boundaries, affecting either the likelihood of word skipping or within-word landing positions, all depending on the words' length and eccentricity. Shorter and nearer words were more often skipped, and even more so as their frequency or their predictability increased. However, as word length increased, word-skipping rate became both smaller and less strongly affected by word frequency/ predictability, while within-word landing positions, closer to the words' center, showed increasingly greater language-related variations. These findings provide a strong case against top-down word-based eye-movement guidance, by revealing that ongoing word-identification processes only modulate default saccade amplitude, as determined by bottom-up, non-word-based, visuo-motor processes.

Reversing reading direction increases precision of eye fixations

Chandra, Johan ; Krügel, André; Engbert, Ralf

Universität Potsdam; Universität Potsdam; Universität Potsdam

For high-acuity visual information processing during reading, saccadic eye movements shifts the reader's line of sight from one word to another. While experimental and theoretical reading models demonstrate that readers aim at word center, the oculomotor accuracy is low compared to other saccadic tasks. As the result, distribution of saccadic landing positions contains large random errors and systematic, distant-dependent, over- and undershoots of word centers (McConkie et al, 1988). This study (N=32) shows that reversing reading direction (i.e. from right to left in German) can simultaneously reduce both random and systematic error components of saccadic landing position distributions. These results are inline with the predictions of a Bayesian model of saccade planning. According to this model, our findings suggest that reading against normal reading direction increases the precision of the sensory identification of word centers.

Reading for comprehension and skim reading: Effects of word frequency and plausibility

Warrington, Kayleigh ; Wang, Xiaotong; Paterson, Kevin; Sui, Xue; White, Sarah
University of Leicester; Liaoning Normal University ; University of Leicester; Liaoning Normal University ; University of Leicester

Three experiments are presented that examine effects of reading goals (reading for comprehension vs. skim reading) on word and sentence processing. Experiments 1 and 2 examined effects of word frequency. In Experiment 1 there was an interaction such that first-pass effects of frequency were smaller during skimming compared to reading for comprehension. This finding builds on previous work examining effects of reading goals on lexical processing (Fitzsimmons, Weal, & Drieghe, 2014; White, Warrington, McGowan, & Paterson, 2015), and indicates that reading goals can modulate lexical processing of fixated words during first-pass reading. Experiment 2 employs distributional analyses to further reveal the time course of effects of reading goals on lexical processing for fixated words. These experiments also provide a strong test of how reading goals might modulate the use of parafoveal lexical information in determining which words are fixated. Experiment 3 examined effects of plausibility by presenting sentences including a critical word that was either plausible or anomalous given the preceding context. The results demonstrated that while effects of plausibility on gaze durations were similar for both reading goals, effects were larger for reading compared with skimming in later measures. This is consistent with the suggestion that standards of coherence (van den Broek, Risden, & Husebye-Hartmann, 1995) may be more superficial during skimming, such that integration of words into sentence context is more limited than during reading for comprehension. The implications for effects of reading goal on lexical processing and integration within models of eye movement control will be discussed.

References

Fitzsimmons, G., Weal, M.J., & Drieghe, D. (2014). *Skim reading: An adaptive strategy for reading on the web.* In, WebSci '14 Proceedings of the 2014 ACM Conference on Web Science, Bloomington, US, 23 - 26 Jun 2014. ACM, 211-219.

van den Broek, P., Risden, K., & Husebye-Hartmann, E. (1995). *The role of readers' standards for coherence in the generation of inferences during reading*. In R. F. Lorch, Jr., & E. J. O'Brien (Eds.), Sources of coherence in text comprehension (pp. 353-373). Mahwah, NJ: Erlbaum.

White, S.J., Warrington, K.L., McGowan, V.A., & Paterson, K.B. (2015). *Eye movements during reading and topic scanning: Effects of word frequency.* Journal of Experimental Psychology: Human Perception and Performance, 41, 233-248.

The time-course of lexical and semantic effects in derived word recognition: a combined EEG and eye-tracking study

Gaisha Oralova, Rober Boshra, Daniel Schmidtke, John Connolly and Victor Kuperman

Abstract

Schmidtke and Kuperman (2018) highlighted an apparent paradox in the estimates of the time-course of word recognition where neural activity for morphological and semantic effects reported in the literature tends to lag behind the timeline of behavioural results (see Dimigen et al., 2011). By using a non-parametric technique of survival analysis, they established the upper temporal bounds at which these effects are expected to emerge in brain activity. In the present study, we co-registered EEG and eye-tracking signals to further explore the reported temporal discrepancy. Native speakers of English read a series of English derived words (e.g., government) shown in sentences and in isolation (lexical decision). Word frequency and semantic transparency were orthogonally manipulated. Eye-tracking results showed a reliable frequency effect in the mean total fixation times when target words were read in sentences (443 ms vs 511 ms) as well as in isolation (868 ms vs 903 ms). Critically, survival analysis showed consistent divergence between high- and low-frequency word lists at 223 ms for words read in sentences, and at 679 ms for words showed in isolation. Fixation-related potential (FRP) analyses demonstrated a word frequency effect peaking at 400 ms with a centro-parietal distribution for words in isolation, and a smaller response peaking at 300-400 ms in sentence reading. FRP analysis is intended to confirm or deny the apparent paradox of whether lexical/semantic effects on eye movement behaviour precede brain activity. Further analysis of semantic transparency and its interaction with frequency will be reported. Methodological implications will be discussed.

References:

Dimigen, O., Sommer, W., Hohlfeld, A., Jacobs, A. M., & Kliegl, R. (2011). Coregistration of eye movements and EEG in natural reading: analyses and review. *Journal of experimental psychology: General, 140*(4), 552.

Schmidtke, D., & Kuperman, V. (2018). A paradox of apparent brainless behavior: The time-course of compound word recognition. *Cortex*.

Reading development between high school years and young adulthood.

Raymond Bertram & Rosa Salmela
University of Turku & Åbo Akademi University, Finland

Over the last decade a large amount of data has been gathered with respect to reading development throughout the elementary school years. Among other things, it has been found that the perceptual span really starts to increase between the 2nd and 3rd grade (Sperlich et al., 2015), and that it reaches adult size during the 6th grade (Rayner, 1986). Yet, reading studies in which 6th graders have been compared with young adults between 20-30 years (Häikiö et al., 2009; Schröter & Schroeder, 2017) have found a clear difference in reading speed across groups, so supposedly processing efficiency still increases in between 12 and 20 years of age. There are no studies investigating reading development during these years though. In the current eye movement study we investigated whether 16-year old Finnish high school students were already at par with young Finnish adults in terms of lexical and complex morphophonological processing. Both groups read sentences including words in three conditions: monomorphemic nouns (lääkäri 'doctor'), inflections (aamu+lla 'in the morning') and inflections with stem alteration (illa+lla [from ilta] 'in the evening'). The results showed that all 3 conditions elicited longer fixation durations, more regressions and shorter saccades for high school students than for young adults. Moreover, an enlarged processing delay was observed for the morphophonological condition for the high school students only. These results indicate that there is still substantial ground to gain at different linguistic levels in terms of reading efficiency between high school years and young adulthood.

References

Häikiö, T., Bertram, R., Hyönä, J., & Niemi, P. (2009). Development of the letter identity span in reading: Evidence from the eye movement moving window paradigm. *Journal of Experimental Child Psychology, 102*, 167-181.

Rayner, K. (1986). Eye movements and the perceptual span in beginning and skilled readers. *Journal of Experimental Child Psychology, 41*, 211–236.

Schröter, P., & Schroeder, S. (2017). The Developmental Lexicon Project: A behavioral database to investigate visual word recognition across the lifespan. *Behavior Research Methods, 49*, 2183-2203.

Sperlich, A., Schad, D.J., & Laubrock, J. (2015). When preview information starts to matter: Development of the perceptual span in German beginning readers. *Journal of Cognitive Psychology, 27*, 511-530

The role of look-backs in processing written sarcasm

Henri Olkoniemia, Eerika Johandera, & Johanna K. Kaakinena,b
a Department of Psychology, University of Turku
b Turku Institute for Advanced Studies, University of Turku,

Previous eye-tracking studies suggest that when resolving the meaning of sarcastic utterances in a text, readers often initiate fixations that return to the sarcastic utterance from subsequent parts of the text. We used a modified trailing mask paradigm to examine both the role of these look-back fixations in sarcasm comprehension and whether there are individual differences in how readers resolve sarcasm. Sixty-two adult participants read short paragraphs containing either a literal or a sarcastic utterance while their eye movements were recorded. The texts were presented using a modified trailing mask paradigm: sentences were initially masked with a string of x's and were revealed to the reader one at a time. In the normal reading condition, sentences remained visible on the screen when the reader moved on to the next sentence; in the masked condition, the sentences were replaced with a mask. Individual differences in working memory capacity (WMC) was measured. The results showed that readers adjusted their reading behaviour when a mask prevented them from re-examining the text content. Interestingly, the readers' compensatory strategies depended on spatial WMC. The present study suggests that look-backs are driven by a need to re-examine the text contents but that they are not necessary for the successful comprehension of sarcasm. The strategies used to resolve sarcasm are mediated by individual differences in WMC.

Sentence and Text Processing II (Room 10 | 10:30 – 12:30)

Effects of Word Predictability on Eye Movements during Arabic Reading

Maryam A. AlJassmi[1,2], Kayleigh L. Warrington[2], Victoria A. McGowan[2], Sarah J. White[2], Kevin B. Paterson[2]
1 *Zayed University, UAE*
2 *University of Leicester, United Kingdom*

The predictability of a word from the prior sentence context has an important influence on both the probability and duration of eye fixations on words in Latinate alphabetic languages like English and German that are read from left to right. However, it is unknown whether a word's predictability has a similar influence on when and where to move the eyes during reading for a Semitic language like Arabic, which is read from right to left and has a very different orthography and morphology. Such knowledge is nevertheless important for establishing the generality of mechanisms of eye movement control across different writing systems. Accordingly, we investigated the influence of word predictability on the eye movements of native, skilled Arabic readers in two experiments. In both experiments, reading times (including first-fixation durations) were shorter for words that were more rather than less predictable from the prior sentence context, consistent with findings for Latinate languages. Predictability did not influence the likelihood of word-skipping for longer Arabic words (ranging from 4-8 letters) that varied in morphological complexity (Experiment 1). However, an effect of predictability on word-skipping was observed for short words (3-4 letters long) with simple morphological structures (Experiment 2). The results are in line with other evidence for low word-skipping probabilities in Arabic reading, and additionally suggest that effects of predictability on word-skipping in this language depend crucially on the length and morphological structure of words. We discuss these findings in relation to the parafoveal processing of upcoming word information during Arabic reading.

Discourse effects on children and adults' online processing of relative clauses

Macdonald, Ross ; Serratrice, Ludovica; Brandt, Silke; Theakston, Anna; Lieven, Elena
University of Manchester; University of Reading; Lancaster University; University of Manchester; University of Manchester

When listening to relative clauses (RC) children show anticipation for a subject (SRC) rather than object relative clause (ORC). Research has suggested that changes to discourse interfere with this SRC bias (Yang, Mo & Louwerse, 2012), however others have argued these findings were due to effects of lexical priming, rather than true discourse effects (Forster & Sicuro Corrêa, 2017). We investigated discourse effects on RC interpretation using ambiguous RCs and preamble sentences with no direct reference to the agents in the target sentence. For example, the target "The man saw the nurse [NP1] with the boy [NP2] *who was very tired*" was employed after one of these preambles:
"It was a long day…
(1) …at the hospital" [NP1-priming]
(2) …at the school" [NP2-priming]
(3) …that Tuesday" [Neutral]
Forty-eight children (aged 4-6) and 30 adults saw pictures of NP1 and NP2 as they listened to the target sentence and their eye movements were monitored. We found no evidence of the preambles influencing online processing, and a strong bias for NP2 anticipation, suggesting that syntax guided the processing for children and adults while discourse did not. We later used unambiguous sentences with varying morphological cues ("The man saw the nurse(s) [NP1] with the boy(s) [NP2] *who was/were very tired*") on adults and found that these cues influenced online interpretation with interference from syntax but not discourse. We are currently testing 4- and 8-year-olds with these new stimuli to investigate the development of the interplay between the influence of morphology, syntax and discourse on RC processing.

Negation and semantic relatedness in eye-tracking-while-reading

Mayer, Erika ; Staub, Adrian; Dillon, Brian
University of Massachusetts Amherst; University of Massachusetts Amherst; University of Massachusetts Amherst

In an influential ERP study, Fischler et al. (1983) found that the amplitude of the N400 response is modulated by the semantic relatedness of a target word to an earlier word, but not by the presence or absence of negation, which should modulate the fit of the target word in the sentence. We investigated whether eye movements in reading show similar insensitivity to negation. Target propositions adapted from Fischler et al. (in square brackets in the example below) were embedded in carrier sentences. Subjects answered comprehension questions. 47 participants read 20 test sentences in a 2x2 design crossing the presence of negation and the semantic relatedness of the critical word (in bold). For the embedded proposition, the semantically related word was true in the affirmative condition while the unrelated word was false, while these truth values were reversed in the negation condition. For example:
The politician said that [a sparrow {is a / is not a} **{bird/bear}**] last Wednesday after the press conference.
We found that go past time on the critical and spillover regions was significantly shorter when preceded by negation, and semantically related critical words led to reduced first fixation, go past, and total time in the critical region. There was no evidence for an interaction between relatedness and negation; readers' eye movements were insensitive to the truth of the embedded proposition. Our results extend Fischler et al.'s findings to eye-tracking: eye movements, like ERPs, suggest that negation does not reverse expectations about an upcoming noun.

Processing of intralingual subtitles by native and non-native speakers: Influence of mismatches between spoken and written words

Walter J. B. van Heuven, *University of Nottingham, UK*
Jack Taylor, *University of Glasgow, UK*

This study investigated the processing of same-language (intralingual) subtitles when people watch a movie. Viewers do not need to read intralingual subtitles in order to understand the movie. However, non-native speakers might process intralingual subtitles to a greater extent when watching a movie in their less proficient language. To investigate viewers' attention to intralingual subtitles, we created mismatches between subtitles and the spoken language by changing (control) words (e.g., GREAT) in 10% of the subtitles to pseudohomophones (e.g., GRAIT) that matched with the spoken word but mismatched with the spelling and to synonyms (e.g., SUPERB). In total 33 participants (15 native and 18 non-native English speakers) watched the first hour of an English spoken and subtitled animated movie ("Spirited Away") while their eye movements were recorded. Afterwards participants also had to indicate whether words presented on screen were said at any point during the film or not (old-new task). The eye movement data revealed longer total fixation durations on subtitles with pseudohomophones and synonyms than on subtitles with matched (control) words. Interestingly, the eyetracking data of the native and non-native speakers were very similar, although only the data of the native speakers showed significantly longer total fixation durations on pseudohomophones than on synonyms. The results of the old-new task revealed for both groups better memory for spoken words that were accompanied in the subtitles with their pseudohomophones than with their synonyms. Overall, the data revealed that viewers read intralingual subtitles even when they are not needed to comprehend the movie.

The processing costs of subordinating and coordinating conjunctions in Brazilian Portuguese: evidence from eye movement data

N. Teixeira, Elisangela ; P. Dantas, Antonio; Arruda de Souza, Brenda
Universidade Federal do Ceara; Universidade Federal do Ceara; Universidade Federal do Ceara

In Rummer, Engelkamp & Konieczny (2003), it was observed that the processing of subordinate clauses is less costly than the coordinate ones in tasks of memorization, as suggested that the displacement of the subordinate clause influences the costs of the processing. As teachers at school, we observed that students could have more difficulties in reading subordinate clauses due to the lack of knowledge of the logical relation served by the conjunction. We conducted three experiments in Brazilian Portuguese: (i) the first one investigated the cost of reading processing of subordinate clause displacement; (ii) the second one compared the reading process of coordinate and subordinate clauses; (iii) and the third one observed the importance of four different conjunctions in coordinate clauses. 42 students and 40 adult readers participated in the experiment. For experiment one, an interaction between group and displacement was found ($F=4,06$, $p = 0.04$). For experiment two, main group effects were found for the first pass ($p=0.04$) and total time ($p<0.001$). In the study of coordinate conjunctions, we observed an significative difference for each conjunction in the first pass, but not in the total time.The results suggest that the students read in a different way and more slowly than the adults, but this results is not significative to coordinate and subordinate clauses. However, the displacement of the subordinate clause to the front position of the sentence generates a higher cost of processing for the students, suggesting that learning this topic is relevant for the schoolers.

Reference

RUMMER, R.; ENGEELKAMP, J.; KONIECZNY, Lars. (2003) The subordination effect: evidence from self-paced reading and recall. *Europen Journal of Cognitive Psychology*, v. 15, n. 3, p. 539-566.

False memories of enriched sentences in context: Evidence from an eye-tracking probe recognition paradigm

Caitlyn Antal
(caitantal@gmail.com)
Department of Psychology, Concordia University
Montreal, QC, Canada
Roberto G. de Almeida
(roberto.dealmeida@concordia.ca)
Department of Psychology, Concordia University
Montreal, QC, Canada

A sentence such as '*We finished the paper*' is indeterminate regarding what we finished doing with the paper. By hypothesis, these sentences require 'semantic enrichment'. These sentences constitute a test case for two major issues on language comprehension. First, whether or not semantic composition is simple ('classical') or enriched with content supposedly provided by the lexical complement 'paper' (a procedure known as 'coercion'). And, second, the nature of the linguistic and cognitive resources that help us interpret the event that the sentence conveys. We conducted an eye-tracking study to investigate whether indeterminate sentences embedded within biasing contexts would trigger contextually driven (though false) event interpretations. Following Sachs (1967, 1974), we developed a probe recognition paradigm whereby participants (N=36; 18 in the current analysis) were presented with short discourse contexts (7 to 13 sentences in length), followed unexpectedly by one of three recognition probe types: the identical sentence (e.g., 'Lisa began the book on Saturday'), a contextually-licensed (but false) probe (...began reading the book...) and a contextually-unlicensed probe (...writing the book...). Probes were presented 0 seconds (s) after having read the indeterminate sentence (always the fourth sentence in the context), or following an additional 25s of neutral discourse. We found statistically significant differences in probe reading time (on the target 'book') for both presentation times only in right-bounded duration (identical > licensed = unlicensed; $F(2, 34) = 3.38$, $p = .046$, $\eta p2 = .166$). We suggest that true and enriched but false propositions linger during reading of indeterminate sentences in context.

Attention III (Plenary room | 10:30 – 12:20)

Pupil size reflects exploration and exploitation in visual search (and it's like object-based attention)

Sebastiaan Mathôt & Franzi Regnath
Department of Psychology, University of Groningen, The Netherlands

The adaptive-gain theory (AGT) posits that there are two modes of behavior: exploitation and exploration. During exploitation, you are calmly focused on a single task; during exploration, you are distractible and rapidly switch from one task to another. The AGT further posits that pupil size reflects these modes of behavior, such that the pupil is larger during exploration than during exploitation; these (light-independent) differences in pupil size are believed to reflect activity in the Locus Coeruleus (LC), such that higher LC activity is accompanied by larger pupils. Here we test the predictions of the AGT in a visual-search task. Participants searched for a single target among 224 distractors. Crucially, the search display was divided into four regions of different colors. These regions were randomly generated and uninformative for the search task. Nevertheless, we found that participants tended to search within regions: They made far fewer between-region eye movements, and far more within-region eye movements, than expected by chance, a result that is reminiscent of studies on object-based attention. Furthermore, we found that participants' pupils dilated just before between-region eye movements, as compared to (matched) within-region eye movements. Taken together, our results suggests that diverting gaze away from one region (or object) towards another region (or object) is a form of "micro-exploration" that is accompanied by pupil dilation and, presumably, increased tonic activity of the LC.

Assessing the functional link between saccades and covert attention by oculomotor plasticity.

Judith Nicolas[1,2,3], Denis Pélisson[1,3] and Aurélie Bidet-Caulet[2,3]

1 *Integrative Multisensory Perception Action & Cognition Team (ImpAct), INSERM U1028, CNRS UMR5292, Lyon Neuroscience Research Center (CRNL), 69000 Lyon, France*

2 *Brain Dynamics and Cognition (Dycog Team), INSERM U1028, CNRS UMR5292, Lyon Neuroscience Research Center (CRNL), 69000 Lyon, France*

3 *University Claude Bernard Lyon 1, Université de Lyon, 69000 Lyon, France*

Saccadic eye movements and shifts of attention permit, respectively, to overtly and covertly scan our visual environment and select meaningful information therein. Previous studies revealed that saccadic adaptation (SA), a well-studied model of oculomotor plasticity, relies on cortical networks overlapping in the parietal cortex with those involved in attention orienting (see references in 1). Whether this anatomical overlap entails a functional interaction between SA and covert orienting of attention is not yet demonstrated.

To decipher whether SA affects covert attention orienting2, we designed behavioral studies in humans, measuring endogenous and exogenous attention shifts in Posner-like cueing tasks3 before and after inducing SA by the double-step target paradigm4. In a voluntary modality study (n=36 subjects), we induced backward adaptation (amplitude shortening) of voluntary saccades and found a significant facilitation of endogenous attention shifts, specifically after adaptation of leftward, and not rightward, saccades. In a reactive modality study (n=18 subjects), we induced backward or forward adaptation (amplitude lengthening) of leftward reactive saccades and found a significant facilitation of exogenous attention shifts, specifically after backward, and not forward, adaptation.

These results demonstrate that oculomotor plasticity boosts covert orienting of attention in both modalities. They fit within a conceptual framework where the right posterior parietal cortex has a dominant contribution in controlling covert attentional shifts through priority maps which are shared with saccades and activated by SA. Further studies are required to disclose possible cross-modality effects, i.e. from adaptation of reactive saccade to endogenous attention or from adaptation of voluntary saccade to exogenous attention.

References :

1. **Nicolas, J.** et al. Saccadic Adaptation Boosts Ongoing Gamma Activity in a Subsequent Visuoattentional Task. *Cerebral Cortex* (2018). doi:10.1093/cercor/bhy241
2. **Habchi, O.** et al. Deployment of spatial attention without moving the eyes is boosted by oculomotor adaptation. *Frontiers in Human Neuroscience 9,* (2015).
3. **Posner, M. I.** Orienting of attention. *Quarterly Journal of Experimental Psychology* 32, 3–25 (1980).
4. **McLaughlin, S. C.** Parametric adjustment in saccadic eye movements. *Perception & Psychophysics 2*, 359–362 (1967).

An ultrafast FIBER fMRI experiment helps in uncovering mechanisms behind ambient and focal modes of gaze behavior

Vadim L. Ushakov[1*], Boris M. Velichkovsky[1,2], Sebastian Pannasch[2], Jens R. Helmert[2], Anastasia N. Korosteleva[1], Boris B. Velichkovsky[3], Maxim G. Sharaev[4]
1 National Research Center "Kurchatov Institute", Moscow, Russia
2 Technische Universität Dresden, Dresden, Germany
3 Lomonosov Moscow State University, Moscow, Russia
4 Skolkovo Institute of Science and Technology, Skolkovo, Moscow Region, Russia
**presenting author, E-mal tiuq@yandex.ru*

Studies of active vision in naturalistic scenes show the existence of two classes of eye movements manifested in ambient and focal visual fixations (Ito et al., 2017; Pannasch, Velichkovsky, 2009; Velichkovsky et al., 2002, 2005). This finding seems to corroborate with the separation of two "streams" of visual processing related either to localization (dorsal system) or to identification of objects (ventral system). A direct verification of this connection proved to be difficult due to an insufficient resolution of the noninvasive brain imaging. Another hypothesis recently attributed the same observation to the lateralization of global and local attention modes in the right and left hemispheres, correspondingly (Mills et al., 2017). Thus, there are two hypotheses that explain changes of eye movement patterns in the process of free image viewing. Using a combination of ultrafast fMRI scanning with the fixation-based event-related (FIBER) method, we measured the dynamics of brain functional activity in its relation to ambient and focal visual fixations in free viewing complex images. Unexpectedly, both hypotheses were confirmed. In line with our early proposal, ambient fixations were accompanied by activation of the structures associated with the dorsal visual pathway, while focal fixations with that of the ventral pathway. At the same time, the second hypothesis also proved to be correct: the activated structures of the dorsal pathway were localized in the right hemisphere and those of the ventral stream mainly—albeit not exclusively—in the left hemisphere. (This research was partially supported by the NRC "Kurchatov Institute", grant 1378/23.08.2017).

Recency effects emerge from the interplay between visual attention and working memory during a delayed match-to-sample task

Marta Balagué, Natàlia Conesa, Laura Dempere-Marco
Bioinformatics and Medical Statistics Research Group
University of Vic-Central University of Catalonia

Abstract: Working memory (WM) allows us to hold and manipulate information when it is no longer present. When stimuli are displayed sequentially, recency effects (i.e. stimuli presented later in the sequence are more likely held in WM) have been reported in visual WM. The main hypothesis that guides this study is that visual attention modulates the codification of visual information during the encoding phase of WM even when stimuli are shown simultaneously, thus affecting which stimuli enter and are maintained in WM. A total of 54 subjects (Age, 36,314,3 years, 31 females) participated in the experiment; a computerized free-view DMS task in which colored squares were presented in arrays of 6 items during 500 ms. Throughout the experiment, the subjects' gaze was monitored by means of an eye-tracker (Tobii X120). In those trials (26% out of 84 trials/subject) in which exhaustive searches (fixations fully contained on ROIs of size 3x3 visual deg centered at the items) were encountered, the last 2 items which were attended during the search were recognized significatively better than those items visited earlier on (Last vs 2nd-last 2=0.713 p=0.3983; 2nd-last vs 3th-last 2=112,081, p<0.05; 3th-last vs 4th-last 2=61,727, p<0.05). This recency effect emerges from the allocation of visual attention to the different items during the stimulation phase of the task, thus illustrating the important role of the interplay between visual attention and WM.

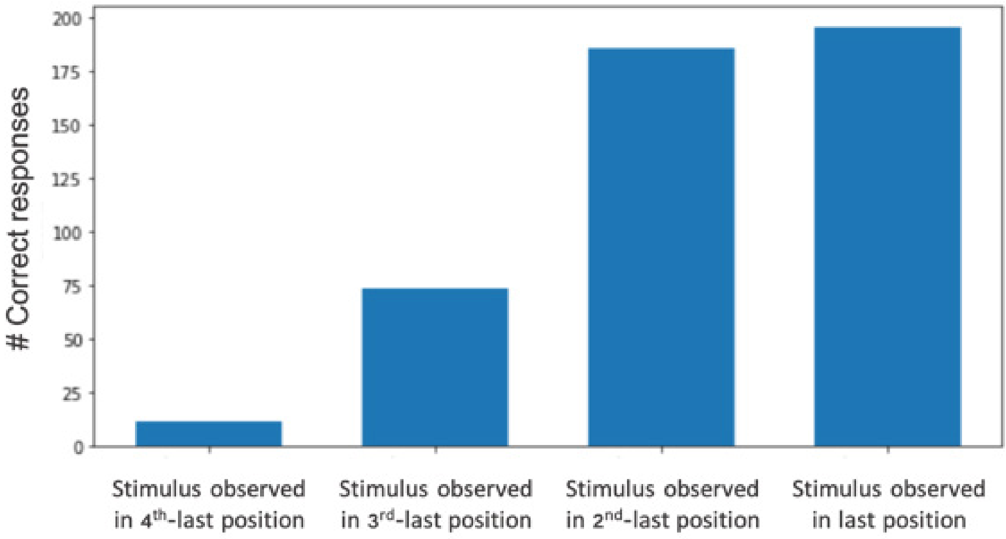

Fig. 1 Recency effect derived from visual attention deployment over the memory set during the encoding phase of the Delayed Match-to-Sample (DMS) task

Eye movements and mental attentional demand in children and adults

Valentina Bachirina[1] **Marie Arsalidou** [1,2*]
1 Neuropsylab, National Research University Higher School of Economics; marsalidou@hse.ru
2 Faculty of Graduate Studies, York University; arsalido@yorku.ca

Mental attentional capacity is the central maturational component of working memory and refers to the amount of information that we can simultaneously maintain and operate (1, 2). Working memory performance has been linked to most key cognitive abilities (3). Despite the fact that vast number of studies have attempted to find connections between eye movements and cognitive performance, very few of them have looked at the developmental perspective and fewer still investigated eye movements directly during task of variable cognitive demand. The purpose of this study was to investigate the relation between eye-tracking indices (e.g., number and duration of fixations) and mental attentional demand in children and adults. Data from adult participants (N = 35, 17 males, mean age = 22.8+/-4.1) showed significant differences between both number and duration of fixations per trial for different levels of mental attentional demand, showing a gradual increase in number of fixations and a complimentary decrease in duration with increase in difficulty. Interestingly, preliminary data with children (N = 15, 4 males, aged 8-9) show a different trend with a drastic increase in number of fixations followed by a plateau in the number and duration of fixations for the more difficult levels, while accuracy of performance was very similar for both age groups.

1] **Pascual-Leone, J.** (1970). *A mathematical model for the transition rule in Piaget's developmental stages.* Acta Psychologica, 32, 301–345.
[2]**Arsalidou, M., Pascual-Leone, J., & Johnson, J.** (2010). *Misleading cues improve developmental assessment of working memory capacity: the color matching tasks*. Cognitive Development, 25(3), 262-277. [
[3]**Simmering V. R., Perone S.** (2013). *Working memory capacity as a dynamic process*. Front. Psychol. 3:567 10.3389/fpsyg.2012

Support is gratefully acknowledged from the Russian Science Foundation (#17-18-01047). The article was prepared within the framework of the Basic Research Program at the National Research University Higher School of Economics (HSE) and supported within the framework of a subsidy by the Russian Academic Excellence Project "5-100."

Fluctuations in pupil size reflect lack of external attention

Jaana Simola[a], Mahiko Konishi[b,c], Satu Palvaa,[d], Jonathan Smallwood[b] & Matias Palvaa,[d,e]

a *Helsinki Institute of Life Science, Neuroscience Center, University of Helsinki, Finland*
b *Department of Psychology / York Neuroimaging Centre, University of York, UK*
c *Laboratoire de Sciences Cognitives et de Psycholinguistique, Dept d'Etudes Cognitives, ENS, PSL University, EHESS, CNRS, Paris, France*
d *Centre for Cognitive Neuroimaging, Institute of Neuroscience and Psychology University of Glasgow, UK*
e *Department of Neuroscience and Biomedical Engineering, Aalto University, Finland*

Unusually small or large pupil dilations are associated with momentary lapses in attention. Here, we examined whether scale-free fluctuations as evidenced by long range temporal correlations (LRTCs) in pupil size time-series are affected by the external task focus. Participants (N=24) saw pairs of shapes (Non-Targets, NTs) followed by a target stimulus. In the 0-back task, they responded which shape matched the presently perceived shape, while the NTs were irrelevant for the task. In the 1-back task, participants responded depending on which side the target was on the previous trial, and they must attend on the NTs. Pupil size were recorded in two sessions consisting of alternating task blocks. Focus of attention was measured by task performance and self-reports collected at the end of the sessions. We used detrended fluctuation analysis (DFA) to obtain the LRTC scaling exponents that quantify the strength of long-range correlations of pupil size time-series. The LRTC scaling exponents were stronger in the 0-back relative to the 1-back task and correlated negatively with the reported levels of detail in thought. Slower response times were further associated with increased levels of self-referential thinking. Our data indicated stronger LRTCs in pupil size when attention was less constrained by the task as well as an association between scale-free pupil size fluctuations and the form of self-generated thought. These results suggest that pupil size fluctuations could be used as an objective marker of the degree of task focus.

Session 7

Oculomotor Behavior (Room 4 | 14:00 – 16:00)

Contrasting gaze-based interaction vs. spontaneous gaze behavior: EEG, MEG and fMRI studies

Sergei L. Shishkin[1], Anatoly N. Vasilyev[1,2], Yuri O. Nuzhdin[1], Ignat A. Dubynin[1], Eugeny P. Svirin[1,3], Anna V. Butorina[4], Darisy G. Zhao[1], Bogdan L. Kozyrskiy[1], Denis G. Malakhov[1], Vadim L. Ushakov[1,5], Alexei E. Ossadtchi[6], Tatyana A. Stroganova[4], Boris M. Velichkovsky[1,7]

1 *National Research Center Kurchatov Institute, Moscow, Russia;*
2 *M.V. Lomonosov Moscow State University, Moscow, Russia;*
3 *University of Würzburg, Würzburg, Germany;*
4 *Moscow State University of Psychology and Education, Moscow, Russia;*
5 *National Research Nuclear University MEPhI, Moscow, Russia;*
6 *National Research University Higher School of Economics, Moscow, Russia;*
7 *Technische Universität Dresden, Dresden, Germany*
E-mail: sergshishkin@mail.ru

Unlike most human behaviors, eye movements with similar overt characteristics can be either automatic or intentional and consciously controlled. Contrasting these two types of eye movements may help to study mechanisms of intention and to search for the “markers” of intention in brain signals accompanying gaze-based interaction with a computer. We review our studies that employed this approach. Samples of gaze-based interaction and spontaneous gaze behavior were collected in participants who played “gaze-controlled” games. The data included EEG recorded under interaction using dwells (Shishkin et al., 2016) and smooth pursuit (Zhao et al., submitted), as well as MEG+EEG and (separately) fMRI recorded under interaction using dwells. Gaze dwells/pursuits were selected for analysis using a set of criteria maximizing contrast on the “intentionality” scale and minimizing influence of irrelevant factors. The EEG related to gaze interaction but not to spontaneous gaze behavior exhibited a component likely related to the feedback expectation, in line with Protzak et al. (2013). The MEG and the fMRI showed signs of brain activity that were unrelated to the feedback expectation and presumably more intention-specific. We are currently using contrasting interaction-related vs. spontaneous gaze behavior to develop an interface that on-the-fly classifies gaze events based on the neural markers, which we call the eye-brain-computer interface (EBCI). Such an interface may enable particularly fluent human-machine interaction.

The study was partly supported by the Russian Science Foundation, grant 18-19-00593 (EEG & MEG studies), and by the NRC Kurchatov Institute, order 1378/23.08.2017 (fMRI studies).

Characterization of natural eye and head movements driving retinal flow

Paul R. MacNeilage, Christian Sinnott, Peter Hausamann
University of Nevada, Reno; Univeristy of Nevada, Reno; Technical University of Munich

In the absence of moving objects, retinal flow is determined by eye velocity relative to the environment as well as by the structure of the environment. Eye velocity in space is the sum of head-in-space and eye-in-head velocity. To gain a better understanding of head and eye velocity driving retinal flow, we developed a system to measure both head and eye velocity during everyday behaviors outside the lab. The system consists of a Pupil Labs eye tracker with an inertial measurement unit (IMU) rigidly attached to the world camera. Head velocity is reconstructed using a computer vision algorithm known as simultaneous localization and mapping (SLAM). The SLAM estimate is supplemented by data from the IMU. Head and eye velocity were recorded for participants walking around campus. Not surprisingly, participants tend to fixate features of the stationary environment, and robust oculomotor stabilization leads to retinal flow that is minimal near the fovea. Linear components of retinal flow are driven by linear velocity of the head. Angular components, however, do not depend strongly on angular head velocity because angular optic flow is largely cancelled by compensatory eye movements. Instead, angular components of retinal flow are driven by compensation for linear optic flow at fixation, which depends on fixation eccentricity relative to the heading direction as well as distance to the scene. Consequently, we observe that retinal flow is driven most strongly by three factors: 1) linear head velocity, 2) fixation direction and distance, and 3) the structure of the environment.

Refixation control and information acquisition at refixations: an EEG-eye movement coregistration study

Andrey R. Nikolaev [1], **Radha Nila Meghanathan** [1, 2], **Marcello Giannini** [1], **Cees van Leeuwen** [1, 2]
1) KU Leuven-University of Leuven, Leuven, Belgium
2) Technical University Kaiserslautern, Kaiserslautern, Germany

Eye tracking research has revealed that refixations (returns to previously visited locations) serve to recover information lost or missed during scanning (Gilchrist and Harvey, 2000; Zelinsky et al., 2011). However, the neural mechanisms of human refixation control are unexplored. We combined eye tracking with EEG registration in a visual search task. EEG epochs related to ordinary fixations and refixations were analyzed for saccade planning effects in the presaccadic interval and for information acquisition effects in the postsaccadic interval. To control for overlapping brain responses in a saccade sequence, EEG epochs were matched between conditions on all relevant eye movement characteristics (Dimigen et al., 2011; Nikolaev et al., 2016). To control for the effect of time, EEG epochs were matched on fixation rank within trials. EEG in the presaccadic interval differed between refixation and ordinary fixation during the shift of attention to the next saccade target. Thus, refixation control operates on saccade planning (Nikolaev et al., 2018). In the postsaccadic interval, EEG differed between refixation and ordinary fixation only in lambda activity over occipital areas, which is associated with perception at fixation. This suggests that early information acquisition is distinct in refixations. Ordinary fixations to locations that were later revisited differed during the entire postsaccadic interval from ones that were not. This suggests attenuation not only of perception but also encoding, which results in loss of information that a subsequent refixation has to recover.

Dimigen O, Sommer W, Hohlfeld A, Jacobs AM, Kliegl R (2011) *Coregistration of eye movements and EEG in natural reading: analyses and review*. J Exp Psychol Gen 140:552-572.
Gilchrist ID, Harvey M (2000) *Refixation frequency and memory mechanisms in visual search*. Curr Biol 10:1209-1212.
Nikolaev AR, Meghanathan RN, van Leeuwen C (2016) *Combining EEG and eye movement recording in free viewing: Pitfalls and possibilities*. Brain Cogn 107:55-83.
Nikolaev AR, Meghanathan RN, van Leeuwen C (2018) *Refixation control in free viewing: a specialized mechanism divulged by eye-movement related brain activity*. J Neurophysiol 120:2311-2324.
Zelinsky GJ, Loschky LC, Dickinson CA (2011) *Do object refixations during scene viewing indicate rehearsal in visual working memory?* Mem Cognit 39:600-613.

Does increasing orthographic transparency modulate word frequency, length, and predictability effects? An eye-movements investigation of processing Arabic diacritics

Hermena, Ehab ; Bouamama, Sana; Liversedge, Simon; Drieghe, Denis
Zayed University; University of Southampton; University of Central Lancashire; University of Southampton

Diacritics are glyph-like marks that add vowel information to Arabic which features a predominantly consonantal script. By virtue of disambiguating the pronunciation of consonants, diacritics make Arabic orthography transparent, and disambiguate the pronunciation of the almost ubiquitous Arabic heterophonic-homographs. Diacritics are typically omitted from print except for the relatively common situations where a particular homograph is not sufficiently disambiguated by the surrounding context (e.g., Hermena, Liversedge, & Drieghe, 2016). In three experiments we investigated whether the presence of diacritics on homographs modulates word frequency, length, and predictability effects. In addition to increased processing costs for comparatively longer (Exp. 1), low-frequency (Exp. 2), and low-predictability targets (Exp. 3), adding diacritics to target words independently prompted the readers to be slower in processing these words. Specifically, diacritized words attracted longer and more fixations from first-pass reading and well into later processing, presumably to perform phonological analyses of the printed diacritics. In addition, the readers re-visited prior regions to the diacritized target word for significantly longer durations (go-past time), to reexamine whether and how the target word, with its particular pronunciation as indicated by the diacritics, fits with the sentence representations constructed by the readers during first pass reading. These findings were true of high- and low-frequency target words (Exp. 1), short and long words (Exp. 2), and low-predictability words (Exp. 3). As such, any potential benefits from diacritics-based increase of orthographic transparency is arguably offset by the costly reading and re-reading strategies that readers adopt upon encountering diacritics on a particular word.

Binocular plasticity occurs in adults with multiscale benefits along its time-course, when elicited by double-step paradigm

Aurélien Morize [1], Botao Peng [1], Dominique Brémond-Gignac [2], Zoï Kapoula [1]
(1) IRIS FR2022, CNRS, France
(2) Necker-Enfants malades, APHP, France

The double step saccade paradigm has been extensively used to demonstrate ocular motor neuroplasticity[1] Vergence disorders are frequent and traditionally treated with 12-18 weekly sessions of visual therapy involving pencil push ups, exercise with prisms, stereograms etc. Kapoula et al. 2016[2], introduced the use of the double step paradigm for rehabilitating vergence to multisensory visual-acoustic targets (REMOBI, US8851669). This study investigates the time course of the vergence neuroplasticity.
Fourteen subjects with vergence disorders (20-77 years) were compared to eight healthy. Their vergence performances were tested before and after rehabilitation with a single step vergence protocol run on the REMOBI device, using an overlap temporal arrangement. Eye movements were recoded with the Eyeseecam device. Subjects with vergence disorders underwent 4-REMOBI rehabilitation sessions containing 2880-double-step vergence for the first-group (N=8), at least twice the quantity manually elicited by traditional visual-therapists but within reduced time-course. The second-group (N=4) participated to a shorten double-step convergence (1800-double-step), while others participated to traditional visual-therapy.
Significant effects occurred after 1-session, namely increase of the peak velocity and decrease of variability, for all parameters, particularly for the gain. Intergroup/subgroups comparisons confirmed persistence of the these results 1-8 months later, and better results than participants who undergone traditional visual therapy. Peak velocity increased particularly in patients who before showed the slower values.
Thus, the time course of vergence rehabilitation can be very fast and advantageous for the patient and the clinician. REMOBI stimulates vergence neuroplasticity efficiently as it enables multiple repetitions in a short time, with well controlled visual-acoustic stimulations.

1. Alahyane-Pélisson(2005).Learn.Memory,12(4),433-443.
2. Kapoula-Morize-Daniel-Jonqua-Orssaud-Bremond-Gignac.Transl.Vis.Sci.Techn.,5(2),8-8

Associations of Age and Sex with Oculomotor Performance in the Rhineland Study

Annabell Coors[1], Ulrich Ettinger[2], David Ward[1], Natascha Merten[1] & Monique M.B. Breteler[1,3]

1 *Population Health Sciences, German Center for Neurodegenerative Diseases (DZNE), Bonn, Germany*

2 *Department of Psychology, University of Bonn, Germany*

3 *Institute for Medical Biometry, Informatics and Epidemiology (IMBIE), Faculty of Medicine, University of Bonn, Germany*

Introduction: Aging processes affect eye movements but previous studies have revealed heterogeneous results about the effects of aging on different eye movement outcomes. In addition to the effects of age, sex differences in eye movements are also not well characterised.

Aim: To quantify the cross-sectional associations of age and sex with oculomotor performance across the adult lifespan. Moreover, we aimed to examine whether any relation between age and performance differed as a function of sex.

Method: Our analysis is based on data from the first 3,000 participants of the Rhineland Study, a community based cohort study in Bonn, Germany, that includes people aged 30 years or older (range 30-95). The 8-hour examination protocol includes eye-tracking examinations (a horizontal "step" antisaccade and prosaccade task, a fixation task and a smooth pursuit task). Performance was analysed with multiple regression models, with age and sex as independent variables and education and visual acuity included as covariates.

Results: Older age was associated with worse performance in pro- and antisaccade latency, antisaccade costs and error rate, prosaccade accuracy, smooth pursuit gain, spatial error and number of saccades in the fixation task. We found no effect of age on pro- and antisaccade peak velocity and antisaccade accuracy. We also observed small sex differences in antisaccade error rate, smooth pursuit gain and number of saccades in the fixation task.

Conclusion: Most oculomotor performance measures gradually decline with increasing age. Men have on average slightly better inhibition abilities in the antisaccade task and faster smooth pursuit movements.

Expertise (Room 10 | 14:00 – 16:00)

Eye Movements Reveal Satisfaction of Search Effects in Proofreading

Barach, Eliza ; Gloskey, Leah; Sheridan, Heather

University at Albany, State University of New York; University at Albany, State University of New York; University at Albany, State University of New York

Subsequent search misses (SSMs) are a type of multiple-target search error in which the discovery of one target reduces the detectability of another target (Cain, Adamo, Mitroff, 2013). SSMs are a major source of errors in radiology, but also occur in non-medical tasks (Fleck, Samei, & Mitroff, 2010), such as baggage scans. To further assess the generalizability of SSMs, we used eye tracking to examine SSMs in proofreading for the first time. To understand the source of these errors, we contrasted the perspective that the discovery of an initial typo results in a less thorough search for additional typos (i.e., the "satisfaction of search" (SOS) account) with the alternative perspective that the discovery of an initial typo consumes working memory resources that otherwise could have aided in the subsequent search (i.e., the "cognitive resource depletion" account). Across two studies, we monitored eye movements while participants either proofread for typos in paragraphs (Experiment 1) or proofread lists of words that were arranged in a straight line or scrambled in random locations across the screen (Experiment 2). We document an SSM effect where detection of a high-salience typo (easy-to-detect; e.g., "carpeb" instead of "carpet") reduced the detectability of a low-salience typo (difficult-to-detect; e.g., "mitsake" instead of "mistake"). Moreover, in support of the SOS account, the detection of the high-salience typo resulted in a less thorough search, as revealed by shorter reaction times, as well as shorter fixation times and fewer refixations on the low-salience target word.

Expertise effects on memory for complex visual search targets: Evidence from eye movements

Kinnera S. Maturi & Heather Sheridan
University at Albany, State University of New York

Across two experiments, we investigated experts' and novices' memory representations for complex visual search targets. Specifically, we monitored the eye movements of expert musicians (≥10 years of music reading experience) and novices (who could not read music) while they searched for a complex search target (i.e., a target bar of piano sheet music) within a search array (i.e., a larger music score that contained the target bar). In Experiment 1, the search target was displayed above the search array throughout the trial, such that participants could refer back to it as needed to support their search. As evidence that experts and novices adopted qualitatively different search strategies, experts had long initial dwells on the search target at the start of the trial, whereas novices exhibited more frequent but shorter dwells on the search target. To further explore this pattern of results, in Experiment 2, we contrasted trials in which the search target and the search array were presented simultaneously (as in Experiment 1) with a condition in which the search target was briefly displayed prior to the presentation of the search array (i.e., the sequential condition). As evidence that novices have a less precise memory representation for the search target, the sequential condition lowered the accuracy of the novices, without impacting the experts' accuracy. Also, the experts' dwell durations revealed that they were rapidly detecting relevant information during their search. Our results coincide with findings from other domains, including radiology and chess, and our results provide support for chunking/template theory.

The perceptual specificity of expertise in music reading: Evidence from eye movements

Abigail L. Kleinsmith *(University at Albany, State University of New York)* and **Heather Sheridan** *(University at Albany, State University of New York)*

To investigate the perceptual specificity of visual expertise in music reading, we monitored the eye movements of experts (>10 year of experience) and novices (who could not read music) during a music-related variant of the flicker paradigm. Specifically, across two experiments, we asked participants to rapidly locate a single note that was changing within a music score. In Experiment 1, we manipulated the visual complexity of music scores to test the prediction of chunking and template theories that experts' domain-specific knowledge would allow them to compensate for increases in visual complexity. In support of this prediction, novices showed larger visual complexity effects than experts across a variety of eye movement measures (e.g., saccade amplititudes, number of fixations, fixation durations). In Experiment 2, we manipulated the familiarity of the music's visual configurations by contrasting upright (i.e., typically oriented) music scores with those that were rotated by 90°. In support of chunking and template theories' prediction that expertise is perceptually specific, we did not observe any expertise effects on eye movements in the rotated music score condition, despite observing robust expertise effects in the upright condition. Building on similar findings in other domains, including radiology and chess, music reading experts display perceptual advantages that are remarkably perceptually specific. Taken together, the present experiments support chunking and template theory's assumption that experts acquire large mental vocabularies of domain-related visual patterns.

Cross-modal music integration in expert memory: Evidence from eye movements

Véronique Drai-Zerbib & Thierry Baccino
University of Bourgogne; University Paris 8

The study investigated the cross-modal integration hypothesis for expert musicians using eye tracking. Twenty randomized excerpts of classical music were presented in two modes (auditory and visual), at the same time (simultaneously) or successively (sequentially). Musicians (N = 53, 26 experts and 27 non-experts) were asked to detect a note modified between the auditory and visual versions, either in the same major/minor key or violating the key. Analyses of eye-movement metrics were carried out at a global level (i.e. testing the effects of modification over the whole score), and at a local level (i.e. focusing only on pre-target, target and post target AOIs), Several main effects of expertise, presentation and note modification were found in the global and local analyses. For most of the metrics (errors, FFD, DT), the results show that experts carried out the task faster (shorter FFD and DT) and with greater accuracy (fewer errors) than non-experts. Sequential presentation was more difficult than simultaneous (longer fixations and higher error rates) and the modified notes were more easily detected when violating the key (fewer errors), but with longer fixations (speed/accuracy trade-off strategy). Experts detected the modified note faster, especially in the simultaneous condition in which cross-modal integration may be applied. These results support the hypothesis that the main difference between experts and non-experts derives from the difference in knowledge structures in expert memory built over time with practice, and cross-modal integration capacities for experts. (e.g. Drai-Zerbib & Baccino, 2014; Drai-Zerbib & Baccino, 2018; Ericsson & Kintsch, 1995).

Extrafoveal perception of complex stereometric shapes in novices and experts

Dreneva, Anna ; Chumachenko, Dmitry; Krichevets, Anatoly; Shvarts, Anna
Lomonosov Moscow State University; Lomonosov Moscow State University; Lomonosov Moscow State University; Utrecht University

Extrafoveal vision has been revealed to contribute greatly to visual perception. However, its definite role is challenging to detect. Here, we investigate extrafoveal processing input in solving a conceptual search task.
The sample included two groups: 13 “experts” being the students and graduates from mathematical faculties and 16 “novices” from psychological ones. The stimuli comprised 3-, 4-, 5- and 6-sided pyramids rotated by different angles (one of each kind was always present on the screen). There were 9 series with 16 trials. Additionally, at the first and the last series the eye movements away from the center were prohibited. The participants had to find either 4- or 5-sided pyramid. To prove extrafoveal processing, we calculated the amount of the visited stimuli before reaching the target.
The results revealed this task to be so difficult that the number of attended stimuli before the target exceeded the random search one. However, the intriguing finding was that in the trials with the eye movements prohibited, we observed an increased performance rate (70% mean value – significantly higher the random guess). We also failed to detect any difference in performance between two groups.
The findings suggest that although extrafoveal processing is barely involved in detecting the complex three-dimensional shapes, it becomes effective in the restricted conditions indicating a flexible shift of behavioral acts in the complex system of perceptual action. The absence of difference between mathematicians and non-mathematicians shows that the skill of identifying stereometric stimuli should require abilities independent of mathematical specialization.

Identifying factors that influence attaining native-like proficiency in L2 speakers of English

Nisbet, Kelly ; Bertram, Raymond; Erlinghagen, Charlotte; Pieczykolan, Aleksandra; Kuperman, Victor
McMaster University; University of Turku; University of Wuerzburg; University of Wuerzburg; McMaster University

This study examines reading behavior in native L1 and highly-proficient L2 readers of English. We investigate whether the L2 disadvantage arises because L2 readers are less proficient in component skills of English reading, or because of an overall processing penalty that depends on the linguistic distance between their specific L2 and English. Parallel eye-tracking experiments were conducted with L1 Canadian (33), and highly-proficient L2 (Finnish (33) and German (28)) university-level readers of English in respective countries. The task was sentence reading in English for comprehension plus a battery testing English language component skills (vocabulary, spelling and print exposure). We found a large main effect of language, with Finnish readers being slower than German readers and both being slower than native English speakers. We utilized a method of statistical prediction to generate hypothetical Finnish and German participants that are assigned maximum native scores in all component skills of English proficiency. This procedure pointed to those skills that give the most leverage to the reading speed of L2 readers and estimated the processing penalty that their L2 imposes. We found that the greatest benefit for German readers of English would come from improved vocabulary knowledge, and for Finnish ones from improved spelling. With native-like mastery of component skills German readers will show the native reading speed. Finnish readers will remain disadvantaged even with maximum component skills. This is due to a much smaller linguistic distance between English and German compared to Finnish. Additional data from Hebrew and Mandarin will be reported.

Cognition II (Room 5 | 14:00 – 16:00)

On the relationship between fixations and perceptual confidence

Emma E. M. Stewart & Alexander C. Schütz
Allgemeine und Biologische Psychologie, Philipps-Universität Marburg, Marburg, Germany

Humans use saccades to gather information about their visual environment, and it is currently debated what sort of memory they retain about fixated objects in a scene, and whether they can remember their scan-path. If humans retain a representation of how much information they have acquired about objects in a scene across fixations, there should be a link between fixations, perceptual confidence and perceptual performance.
We presented observers with an array of everyday objects for two seconds, and obtained perceptual confidence and perceptual performance for those objects afterwards. We measured perceptual confidence by allowing observers to choose two objects they wanted to report. Subsequently, they had to report the orientation of a chosen or a non-chosen object. We compared observers' choices to their fixation patterns, the amount of information gained during fixations, and low-level object properties such as salience that may have contributed to the selection of saccade targets. The results indicate a link between perceptual confidence and fixations: observers were more confident in judgements of objects they previously fixated. Confidence was linked to performance, as perceptual performance was higher for chosen than non-chosen objects. Both confidence and fixations were linked to low-level object properties, as participants tended to both fixate and choose more salient objects.
These results suggest that humans do retain some representation of how much information they have gained about different objects and that they can use this information to improve their perceptual discrimination performance. This ability might include some implicit memory of their fixation behaviour.

Differences in learning between global and vector-specific saccadic adaptation

Carlos Cassanello[1], Florian Ostendorf[2], Martin Rolfs[1,3]
1 *Department of Psychology, Humboldt-Universität zu Berlin, Germany*
2 *Charité Universitätsmedizin Berlin, Germany*
3 *Bernstein Center for Computational Neuroscience Berlin, Berlin, Germany*

Rolfs and collaborators (2010) studied saccadic adaptation using a version of the double-target-step paradigm, in which the initial target step could take any direction and the second (intra-saccadic) step had fixed inward size. Saccade-amplitude gain decreased globally, rapidly and efficiently across trials. This global adaptation could not be modeled assuming superposition of vector-specific adaptation across visited directions. Thus, global adaptation may involve mechanisms different from those responsible for vector-specific adaptation and may actually have faster onset. Using a similar paradigm in which the second step varied sinusoidally in size between -25% and 25% of the pre-saccadic target amplitude, we adapted saccades either along horizontal (Two-way) or random (Global) directions. We fitted a family of generative models, extracted learning parameters and performed statistical selection to identify which model was most likely to generate each adaptation type. Global adaptation responded obligatorily to the last experienced error and transferred across directions with a timescale of the order of 30-50 trials. Evolution of saccade-amplitude gain in Two-way adaptation was better fitted if the learning algorithm included a second sampling of the feedback error. This double error-based correction facilitated extraction of stimulus variations over a longer number of trials and extended the integration window of the learning model about tenfold. Based on these results, we will discuss possible origins of vector-specific adaptation.

Rolfs M, Knapen T, Cavanagh P. Global saccadic adaptation. *Vision Research 50*: 1882–1890, 2010.

This work was supported by the DFG (grants RO 3579/2–1, RO 3579/3-1, RO 3579/8-1).

Eye blinks increase local field beta activity in visual cortex during a visual task

Franz Aiple[1], Armin Brandt[1], Julie Blumberg[2], Peter Reinacher[3], Markus Kern[4], Andreas Schulze-Bonhage[1]
1 *Epilepsy Center, Univ.Medical Center, Freiburg, Germany*
2 *Dept of Neuropediatrics, Univ.Medical Center, Freiburg, Germany*
3 *Dept of Stereotactic Neurosurgery, Univ.Medical Center, Freiburg, Germany*
4 *Translational Neurotechnology Lab, Dept of Neurosurgery, Univ.Medical Center, Freiburg, Germany*

Epilepsy patients were implanted with depth electrodes in temporal and occipital brain regions for diagnostic purposes. Intracortical local field potentials (LFPs) were recorded continuously while participants performed a visual task. Images were presented for 500ms, followed by inter stimulus intervals of 1330ms. Eye blinks were detected on the basis of EOG signals. Two classes of trials - with or without eye blinks - and 2 classes of eye blinks - inside or outside of trials - were analyzed separately. Eye blinks in trials were mostly time locked to stimulus onset (STON), occurring mainly between 700 and 1100ms after STON. LFPs were analyzed with respect to stimulus onset and eye blink occurrence times, respectively, for different frequency bands in the delta to gamma range (0.5-256Hz). STON-aligned averaging revealed visual evoked potentials (VEPs), blink-aligned averaging revealed blink related potentials (BRPs) in many electrodes. Coupling of blinks to trial timing confounded the two potentials and had to be resolved by analyzing amplitude and sharpness of peaks. In occipital electrodes (areas V1, V2, FG) beta activity (11-32Hz) increased shortly after blinks in 4 of 5 patients, more strongly for blinks within than outside of trials. Otherwise STON was followed by a decrease in beta activity. Modulation of beta activity during visual tasks was discussed in the literature in the context of attention, with less attention given to the contribution of eye blinks. Here, we report that beta activity is decreased after the appearance of visual stimuli and increased after eye blinks.

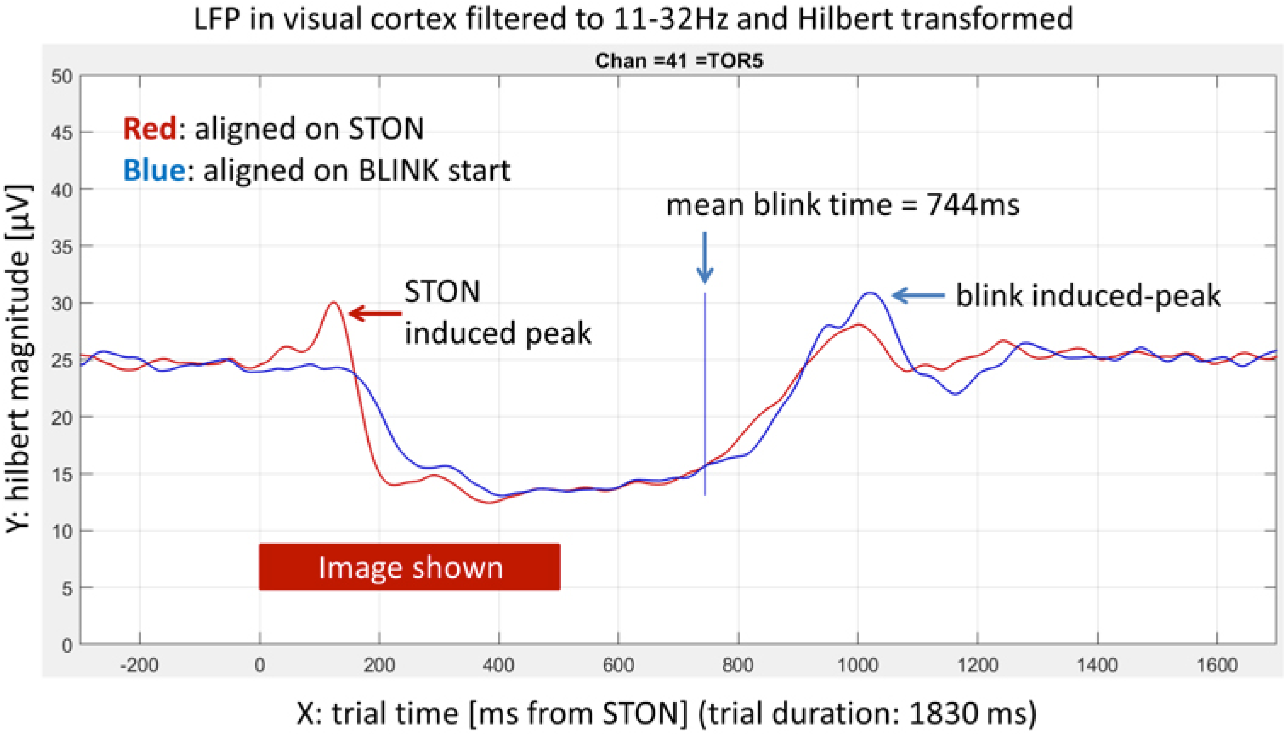

As the red peak is bigger than the blue at ~130 ms we conclude that it is mainly induced by the appearance of the image (STON). As the blue peak is bigger than the red at ~1000 ms we conclude that it is mainly induced by the blink happening shortly before (blue vertical line)

Nothing to lose? An exploration of eye-movements and the zero effect in risky choice

Jonas Ludwig[1,2], Alexander Jaudas[1,3], Anja Achtziger[1]

1 *Department of Political and Social Sciences, Zeppelin University Friedrichshafen*

2 *Department of Psychology, University of Education Weingarten*

3 *Laboratory for Social and Neural Systems Research, Department of Economics, University of Zurich*

Decision makers presented with lottery choices typically prefer safe wins over risky gambles, even if the latter promise potentially higher payoffs than the former. This deviation from expected utility theory is usually explained by the certainty effect, whereby probabilities of zero or one are interpreted accurately but intermediate probabilities are distorted by diminishing sensitivity. We explored an alternative explanation for deviations from expected utility theory that are in line with the certainty effect. Based on earlier work on the zero effect in risky choice, we examined the role of zero-outcomes (i.e., outcomes with zero utility) in risky lottery choices. We proposed that decision makers exhibit a strong aversion to receiving zero-outcomes, rather than being attracted to certainty (i.e., probability of one), and that this could explain typical deviations from expected utility theory.

We conducted an experiment (N = 35) in which we measured eye-movements and pupil dilations to investigate visual attention as a key component of the zero effect. The findings suggested that gambles were avoided when a zero-outcome was included. Eye-movements indicated that zero-outcomes were substantially less fixated than other outcomes. We concluded that decision makers were strongly motivated to avoid zero-outcomes and that this translated into processes of predecisional information search. Zero-outcomes were generally eschewed, suggesting that they invoked heuristic processing based on an affective reaction elicited by the zero utility of such outcomes. Pupillometry further supported the idea that affect mediated the role of zero-outcomes in decisions under risk.

Key words: Decisions under risk, Risky choice, Zero effect, Eye-tracking, Pupillometry

Perceptual complexity of face stimuli modulates gaze following in old age

Fernandes, Eunice G., *University of Aberdeen, School of Psychology*
Phillips, Louise, *University of Aberdeen, School of Psychology*
Slessor, Gillian, *University of Aberdeen, School of Psychology*
Tatler, Benjamin W., *University of Aberdeen, School of Psychology*

From an early age, we show a clear tendency to attend to the locations that we see others attending to. This gaze following response seems to decline as we move into old age, but why this might be is not well understood. At present evidence for reduced gaze following in old age comes mainly from work that does not include measures of eye movements, or that does, but does not use photographic faces as the gaze-cue-providing stimuli (e.g., Kuhn et al., 2015). We recorded eye movements of old and young adults as they completed a gaze cuing paradigm in which we manipulated the time between gaze cue onset and target onset (SOA: 100, 300, 600, 1000 ms) and the type of face stimulus (schematic, photograph of a young face, photograph of an old face). We found more gaze following responses (saccades in the same direction as the gaze cue) in young participants than in old participants. Contrary to previous studies, we found no evidence of an own-age bias in gaze following when participants were presented with photographic stimuli. Crucially, however, gaze cueing was larger for older participants, when they were presented schematic relative to photographic faces. These findings are consistent with the suggestion that decline in gaze following in old age might arise from perceptual decline, with preserved gaze following only for the schematic faces, which provide more perceptually salient and easy to discriminate cues.

Session 8

Thursday, August 22nd

Oculomotor Dysfunction II (Room 10 | 10:30 – 12:30)

Quality assessment of nystagmus data from eye tracker recordings

William Rosengren*, Marcus Nyström*, Björn Hammar*, Mar-kus Rahne*, Linnéa Sjödahl* & Martin Stridh*
** Lund University, Lund, Sweden*

Pathological nystagmus is a condition causing involuntary oscillating eye movements, which are often investigated using data recorded from an eye tracker. A common problem when analysing these data is to discard seg-ments containing recording artefacts, and include only the clinically rele-vant oscillations. Nystagmus signals are modelled using a harmonic sinus-oidal series. The normalised segment error (NSE) is used to separate oscil-latory from non-oscillatory segments based on differences in energy be-tween the observed signal and the model estimated signal reconstruction. Simulations of nystagmus signals corrupted by disturbances are analysed using the proposed error metric. A binary classification threshold is de-rived by optimising the receiver operator characteristics (ROC) for detec-tion of the introduced disturbances. The threshold is applied to recordings from nystagmus patients, recorded with an EyeLink 1000 Plus. For simu-lated data, the true positive rate and false positive rate of detected disturb-ances were 0.95 and 0.1, respectively. The performance of the NSE for re-al signals was in good agreement with results from the simulated signals. The NSE is a useful metric to identify segments with genuine nystagmus oscillations, and may be used as a pre-processing step for further analyses or modelling of the data.

Computational discrimination between natural images based on gaze during mental imagery

Xi Wang, Kenneth Holmqvist, Marc Alexa
TU Berlin; Regensburg University; TU Berlin

The term "looking-at-nothing" describes the phenomenon that humans move their eyes when looking in front of an empty space. Previous studies showed that eye movements during mental imagery while looking to nothing play a functional role in memory retrieval[1][2]. However, they are not a reinstatement of the eye movements while looking at the visual stimuli[2][3] and are generally distorted due to the lack of reference in front of an empty space[2][4]. So far it remains unclear what the degree of similarity is between eye movements during encoding and eye movements during recall.

We studied the mental imagery eye movements while looking at nothing in a lab-controlled experiment. 100 natural images were viewed and recalled by 28 observers, following the standard looking-at-nothing paradigm[1][2]. We compared the basic characteristics of eye movements during both encoding and recall. Furthermore, we studies the similarity of eye movements between two conditions by asking the question: how visual imagery eye movements can be employed for computational image retrieval. Our results showed that gaze patterns in both conditions can be used to retrieve the corresponding visual stimuli. By utilizing the similarity between gaze patterns during encoding and those during recall, we showed that it is possible to generalize to new images. This study quantitatively compared the similarity between eye movements during looking at the images and those during recall them, and offers a solid method for future studies on the looking-at-nothing phenomenon.

[1] **Laeng B, Bloem IM, D'Ascenzo S, Tommasi L** (2014) Scrutinizing visual images: The role of gaze in mental imagery and memory. *Cognition 131*(2):263 – 283.

[2] **Johansson R, Holsanova J, Holmqvist K** (2006) Pictures and spoken descriptions elicit similar eye movements during mental imagery, both in light and in complete darkness. *Cognitive Science 30*(6):1053–1079.

[3] **Scholz A, Mehlhorn K, Krems JF** (2016) Listen up, eye movements play a role in verbal memory retrieval. *Psychological research 80*(1):149–158.

[4] **Johansson R, Holsanova J, Dewhurst R, Holmqvist K** (2012) Eye movements during scene recollection have a functional role, but they are not reinstatements of those produced during encoding. *Journal of Experimental Psychology: Human Perception and Performance 38*(5):1289–1314

Retinal disorders related nystagmus

Ping Wang Dongsheng Yang
Shandong Purui Eye Hospital
Pediatric Eye Disease and Ocular Motility Institution of Jinan Purui

Introduction: To better understand the mechanisms of the nystagmus waveform, we report a special type of nystagmus waveform and attempt to establish a relationship with retinal disorders.
Methods: We collected data from 136 patients who had a special waveform featured with pendular low amplitude and high frequency (PLAHF), or had an additional PLAHF component on top of their large nystagmus eye movement. All of these patients were sent to do ERG and OCT tests. Meanwhile, data for BCVA and fundus photography were also collected. All patients were recommended to have genetic tests done. Among 136 patients, 52 patients had the genetic test done.
Results: 1. All 136 patients were diagnosis to have retinal disorders based on ERG and OCT test results. 2. The 52 patients who did the genetic tests, 46 of them got positive genetic mutations pointing to retinal disorders, negative results were returned in another 6 patients. 3. Analysis on eye movement recording (EMR) revealed that 13 patients had pure PLAHF waveform. The amplitude was a 2~3 degree high and the frequence was 5~10Hz. The PLAHF waveform were superimposed on top of large horizontal or vertical jerk nystagmus in 23 patients. In 16 patients, INS nystagmus waveform combined with interval of pure PLAHF or superimposed waveforms were identified.
Conclusions: We hypothesized that the PLAHF waveform may related to retinal disorders since protein mutations were identified to be on the retina.Patients with such waveform should be referred for further electroretinographic investigation as well as gene tests.

Traditional analysis of VOR nystagmus provides weak diagnostic features – Segment-based analysis is more robust and unmasks potential non-linearities.

H.L. Galiana, Professor and **Heather LE Smith,** Research Assistant
Department Biomedical Engineering, McGill University, Montreal, Canada
Email: Henrietta.galiana@mcgill.ca

Traditional approaches to the analysis of ocular nystagmus focus on the slow phases of reflex nystagmus to find a slow-phase (SP) 'envelope' of eye velocity. This is the standard analysis for evaluating function during the vestibulo-ocular reflex (VOR), optokinetic nystagmus (OKN), or target pursuit (TP) velocity. Here we focus on the VOR to illustrate analytical issues caused by nystagmus (switching in neural circuits). For example:
To characterize the VOR, nystagmus velocity is computed, and a fit is produced through all slow-phase segments (ignoring fast phases) to parameterize the dynamics (gain/phase or gain/time constant) of the VOR response during step or harmonic passive head rotation.
These approaches are only valid if the assumption of a perfect 'neural integrator' (NI) in the oculomotor pathway (Robinson, 1981) is satisfied. With small NI time constants (<3s), the envelope metrics are very labile and fast-phases affect the slow-phase time profile. For example, during constant head rotation speed, the eye velocity 'envelope' can decay with variable time constants, ranging from 4s to 30s, just by changing the alertness level of a test subject, and with it, the fast phase frequency and end-points. Hence the estimated 'velocity-storage time constant', Cohen & Raphan) is a weak indicator of VOR health. We recommend instead that slow-phase velocities be examined in the context of their local data (initial conditions, head velocity) while fitting individual slow-phase segments. This approach accounts for the contribution of fast phases, and isolates the true VOR dynamics and non-linearities of the vestibular sensors despite variations in fast phases. Examples will be provided from simulated and experimental data. This approach provides robust metrics, unless the reflex itself has changed its slow phase dynamics with context (FP rate, vestibular NL, range of head velocity) through practice or rehabilitation. With this approach, new norms for controls in nystagmus will need to be re-evaluated, but they should be in a much narrower range than from current envelopes. Similarly, differences with patients will be amplified, providing for more sensitive detection of deficits. The same arguments against envelope methods apply to all ocular nystagmus responses (VOR, OKN, pursuit), and to any movement trajectory that imbeds two modes of dynamics during tracking (head, hand reaching…). Simply said, the envelope analysis of nystagmus evaluates the total effect, on average, of both sensory contributions and fast-phase contributions that optimize the ocular set-point in the orbit with current sensory drive. To isolate the sensory contribution, a segment-based fit must be used.

Oculomotor control in children with Posterior Fossa Tumors: eye tracking evidence

Shurupova, Marina ; Anisimov, Victor; Kasatkin, Vladimir; Karelin, Alexander; Latanov, Alexander
Moscow State University, Russian Federation; Moscow State University, Russian Federation; Dmitry Rogachev National Research Center of Pediatric Hematology, Oncology and Immunology, Russian Federation; Dmitry Rogachev National Research Center of Pediatric Hematology, Oncology and Immunology, Russian Federation; Moscow State University, Russian Federation

Posterior fossa tumors (PFC) such as medulloblastoma, astrocytoma, ependymoma affect cerebellum and its connections that are associated with eye movements. Children with PFC also show massive impairments with movement control and cognitive functions. Analysis of saccades and gaze stability could be applicable for evaluation oculomotor system condition and related brain functions. 61 patients with PFC (M=12.80; SD=2.66) and 50 healthy children (M=12.96; SD=2.46) participated in our study. We tested gaze stability, timed visually-guided saccades and visual search in these two groups. Eye movements were recorded with Arrington Research Eye Tracker (60 Hz). We found a significant decrease of gaze stability in patients, presumably related to weak inhibition of intrusive saccades during fixation and unwanted distracting saccades to other stimuli. Further, we revealed a significant amount (20,4%) inaccurate hypermetric saccades in patients in visually-guided saccades test comparing to healthy children (1,4%). Such hypermetric saccades often adjust by corrective saccades and lead to worse performance and increase a test time. Finally, patients demonstrated increased total execution time, fixations number and scanpath length in visual search test. Overall, patients with PFC show weak oculomotor control and visual processing, apparently because of the lesion in vermal and hemispheric oculomotor centers in cerebellum and wide toxic effects on the brain after cancer therapy. Eye tracking method provides a non-invasive reliable assessment of oculomotor and cognitive functions in such patients.

Neural Modelling of Antisaccade Performance of Healthy Controls and Huntington Disease Patients

Vassilis Cutsuridis[1,*], Matt J. Dunn[2], James Brawn[2], Anne Rosser[3], Jonathan T. Erichsen[2]

1 School of Computer Science, University of Lincoln, Lincoln, U.K.

2 School of Optometry and Vision Sciences, Cardiff University, U.K.

3 Division of Psychological Medicine and Clinical Neurosciences, School of Medicine, Cardiff University, U.K.

**Corresponding author: vcutsuridis@lincoln.ac.uk*

In the antisaccade paradigm, subjects are instructed to fixate a central stimulus and then perform an eye movement directly away from a peripheral visual stimulus. The paradigm requires the parallel programming of two decision processes: the suppression of an erroneous prosaccade towards the peripheral stimulus and the initiation of a volitional antisaccade to the mirror opposite position. Although healthy controls (CTL) typically make few errors, patients suffering from Huntington's disease (HD) make more errors and display increased and more variable latencies of error prosaccades and antisaccades. Deficits in the antisaccade performance of these patients are generally interpreted as an impaired top-down inhibitory signal that fails to suppress the erroneous responses. Neural network models with mutual inhibition implementing non-linear accumulation of information prior to decision making (eye movement) are presented. Two decision signals representing the volitional antisaccade from the Frontal Eye Fields and the reactive prosaccade from the Lateral Intraparietal Area are integrated in a competitive neural network of the superior colliculus. The model accurately reproduces the error rates and latency distributions of error prosaccades, antisaccades and corrected antisaccades of CTL and HD cohorts of subjects. The model predicts that antisaccade performance of HD patients is due to a noisier rate of accumulating information, although they are less confident about their decisions than the controls. Finally, competition between the two decision processes in the superior colliculus, and not a third top-down inhibitory signal that suppresses the erroneous response, accounts for the antisaccade performance in healthy and HD subjects.

Sentence and Text Processing III (Room 5 | 10:30 – 12:30)

Comparison of eye-movement patterns during reading across scripts and languages: MECO, the Multilingual Eye-Movement Corpus

Kuperman, Victor ; Siegelman, Noam McMaster; *Haskins Laboratories*

This presentation introduces the new Multilingual Eye-movement COrpus (MECO). MECO is a collection of eye-tracking data registered during reading of text passages in the native language of the participant and in English. Currently, data collection is underway in (Palestinian) Arabic, (Mandarin) Chinese, English, Finnish, German, Hebrew, Russian, (Argentine) Spanish, and Turkish in samples of 50 university students. Several other languages will be added later. The MECO-L1 component of the corpus reports native reading of 12 passages for comprehension (~150 words each). Five passages are matched across languages through translation from English, and additional seven are non-translated texts on the same set of topics. The eye-movement data are supplemented by comprehension questions and a battery of verbal skill tests administered in the participants' native language. The MECO-L2 component consists of 12 texts in college-level English each followed by comprehension questions: it is supplemented by an extensive battery of component skills in English (spelling, word- and non-word reading, vocabulary size, motivation, reading habits, language history questionnaire etc).
In line with Liversedge et al. (2016), MECO-L1 enables a direct comparison of the reading behavior across different (alphabetic, abjad, logographic) writing systems and languages. We present comparative data on saccadic and fixation patterns at the level of character, word, sentence and passage both for subsets of typologically similar scripts (Semitic consonantal abjads; agglutinative alphabetic Finnish and Turkish; and Roman and non-Roman alphabetic) and across all scripts. We also demonstrate which component skills contribute the most to the reading comprehension level in different native languages.

Good-enough processing while reading under no-noise and visual-noise conditions

Anastasiya Lopukhina, Anna Laurinavichyute, Svetlana Malyutina
National Research University Higher School of Economics, Russia
University of Potsdam, Germany
alopukhina@hse.ru

Sentence comprehension relies not only on algorithmic parsing of grammatical structure but also on superficial good-enough processing (Ferreira et al., 2002). That is, we tend to guess relations between words based on their meaning and our real-world knowledge, without building accurate syntactic representations. Previous studies have shown that uncertain input can trigger good-enough parsing (Levy, 2011), however the evidence is very limited. We aim to investigate whether visual linguistic noise disturbs sentence processing and induces greater reliance on lexico-semantic cues.
Participants (N = 58, MAge = 23) read sentences and answered binary-choice comprehension questions while their eyes were tracked. Half of the stimuli were presented in visual noise (short phrases that appeared for 300-400 ms at random positions on the screen). Stimuli sentences (N = 56) were either plausible or implausible: their syntactic structure either matched or contradicted the semantic relations between words. We hypothesized that the decrease of processing speed and comprehension accuracy in the implausible condition should be greater in visual-noise condition.
Surprisingly, this is not the case: participants show a comparable accuracy decrease in implausible sentences both in visual-noise and no-noise conditions. We found no evidence that participants slow down while reading implausible sentences in the presence of noise. On the contrary, in noisy conditions, readers generally speed up, which is reflected in first run dwell time (95% CrI=[-20, -6] ms), total reading time (95% CrI=[-42, -21] ms), and rereading measures (95% CrI=[-39, -11] ms).
The study was supported by RFBR grant № 18-012-00640.

Ferreira, F., Bailey, K. G., Ferraro, V. 2002. *Good-enough representations in language comprehension*. Current directions in psychological science, 11(1), 11-15.
Levy, R. 2011. *Probabilistic Linguistic Expectations, Uncertain Input, and Implications for Eye Movements in Reading*. Studies of Psychology and Behavior 9(1):52–63.

The time course of emoji processing during reading: Evidence from eye movements

Sheridan, Heather ; Barach, Eliza; Feldman, Laurie B.

University at Albany, State University of New York; University at Albany, State University of New York; University at Albany, State University of New York, Haskins Laboratories

Due to the quick rise in popularity of emojis – with close to 3000 emojis listed in the official standard code at the time this abstract was submitted – emojis could provide a new frontier for studying the role of ideograms and non-verbal symbols during reading. In the present study, we used eye tracking to examine the time course of emoji processing during sentence reading. Specifically, we monitored participants' eye movements while they read sentences containing a target word (e.g., "cookies" in the sentence "These homemade cookies are delicious"). The sentences either contained no emoji, or there was an emoji located immediately after the final word of the sentence. The emoji was either congruent with the target word (e.g., the cookie emoji), or incongruent with the target word (e.g., the pie emoji). This emoji congruency manipulation produced a rapid and long-lasting effect on the eye movement record. As evidence that emoji processing begins in the parafovea, the incongruent emojis were skipped less often than the congruent emojis. Also, analogous to the disruption caused by semantically incongruent words during reading, the incongruent emojis were fixated for longer than the congruent emojis, and the incongruent emojis also elicited more regressions and longer overall sentence reading times. We discuss the implications of our results for models of eye movement control during reading, which could be extended to accommodate emojified text.

Do Readers Skip Predictable Words More than Unpredictable Words in Syntactically Illegal Positions: An Eye-tracking Study

Michael G. Cutter *(University of Edinburgh)*, **Andrea E. Martin** *(Max Planck Institute for Psycholinguistics)*, & **Patrick Sturt** *(University of Edinburgh)*.

During reading, people skip predictable words more than unpredictable words (e.g. see Fitzsimmons & Drieghe, 2013). In an eye-tracking study we examined whether this effect occurs even when these words appear in syntactically illegal positions. Participants read sentences in which a target word became predictable by a certain point (e.g. “bone” is 92% predictable in a cloze task given “The dog buried his…”), with the next word actually being an intensifier (e.g. “really”), which a noun cannot follow. The target noun remained predictable to appear later in the sentence, being provided at some point in 89% of sentence completions beyond “really”. We used the boundary paradigm (Rayner, 1975) to present the predictable noun or an alternative unpredictable noun (e.g. “food”) directly after the intensifier, until participants moved beyond the intensifier. Participants also read sentences in which predictable or unpredictable nouns appeared in syntactically legal positions. Preliminary analysis (52 subjects; OSF pre-registration commits to 78) using a Bayesian linear-mixed model suggests that readers skipped the predictable noun 5.2% more than the unpredictable noun when it appeared in a legal position, while there was a smaller 3.8% effect of predictability when the nouns appeared in illegal positions. We will discuss our findings in relation to the nature of lexical prediction during reading.

References

Fitzsimmons, G., & Drieghe, D. (2013). How fast can predictability influence word skipping during reading? *JEP:LMC, 39*, 1054-1063.
Rayner, K. (1975). The perceptual span and peripheral cues in reading. *Cognitive Psychology, 7*, 65-81.

Effects of reading goals on the eye movements of young and older adult readers

McGowan, V.A., Paterson, K.B., Warrington, K.L., & White, S.J.
University of Leicester

Determining how eye movement behaviour during reading changes across the adult lifespan is crucial for understanding how the mechanisms underlying reading are affected by age. Previous studies have demonstrated that older adults (aged 65+ years) read more slowly and show a different pattern of eye movement behaviour to young adults (aged 18-30 years) when reading normally for comprehension. Yet little is known about whether these age effects remain stable across different reading goals. In this experiment we recorded the eye movements of young and older adult readers during normal reading, skim reading, and careful reading. Sentences included a target word manipulation of word frequency or word predictability. When reading normally, a typical pattern of age differences was observed, and so older readers read more slowly than their younger counterparts, and made more and longer fixations, and more regressions. For both young and older readers careful reading produced longer reading times, more fixations and regressions, and shorter forward saccade lengths than normal reading. In contrast, skim reading produced shorter reading times, fewer and shorter fixations, fewer regressions, and longer forward saccade lengths than normal reading. Crucially effects of skim reading were more pronounced for young than older readers, but both age groups showed similarly attenuated effects of word frequency and predictability in this condition. Overall, the results indicate that young adults are more flexible than older adults in modifying their eye movement behaviour in response to current reading demands, but this is not attributable to differences in lexical access.

Fixation positions during reading reflect visual sampling strategies

Hautala, Jarkko ; Loberg, Otto; Leppänen, Paavo
Niilo Mäki Institute; University of Jyväskylä; University of Jyväskylä

Progressive reading saccades are mainly guided by word spaces, which allow targeting to optimal viewing position of word center. Suboptimal landing positions resulting from oculomotor error are often rapidly corrected by glissades. In addition, long words and post-skipping words are landed towards word beginning, presumably in preparation to make a progressive refixation. The latter findings may indicate planning of saccadic sequences in order to optimize sampling of visual information. However, due to the standard usage of monospaced font in research, it is actually not well understood to which extent saccadic behavior in reading is governed by letters and to which extent spatial width of the words. Few eye tracking studies have approached this question by orthogonal manipulations of number of letters contained and spatial width of a word. The results of these studies suggest that all reading saccades including refixations and word skips are predominantly guided by spatial width of the words and to a lesser degree by number of letters contained. Linked mixed modelling analyses of fixation positions were used to estimate the influence of 1) launch site and its oculomotor error, 2) spatial width and number of letters of successive words, 3) parafoveal preview and 4) reading fluency on saccadic control during reading. Implications for theories of eye movement control in reading will be discussed.

Session 9

Reading Models II (Room 5 | 14:00 – 16:00)

Readers can identify the meanings of words before looking at them: Evidence from eye movements and re-reading

Elizabeth R. Schotter & Anna Marie Fennell
University of South Florida

Processing linguistic information from words parafoveally leads to faster reading (Schotter et al., 2012), but does that mean that words are identified before they are fixated? Fixation times are not only shorter when the preview is valid (i.e., identical or similar to the fixated target; Vasilev & Angele, 2018), but also when it is *plausible*, regardless of its similarity to the target (Schotter & Jia, 2016; Veldre & Andrews, 2016). This latter finding suggests that readers may identify the meanings of words without looking at them, based entirely on the preview, and that representation is may not be overridden by information obtained when a different target word is subsequently fixated. We tested whether readers maintain the meaning of the preview with a *gaze-contingent display change study* (Rayner, 1975) and subsequent plausibility manipulation. Both the preview and target were plausible when encountered and in an immediately following region, and we manipulated the end of the sentence so that the different preview was either rendered implausible (in *critical* sentences) or maintained its plausibility (in *neutral* sentences). Regressions from the end of the sentence increased when the preview was rendered implausible, indicating that the preview's meaning had been encoded. These data add to a growing body of research suggesting that semantic information is obtained during preview, to the point where word meaning is accessed. Furthermore, these findings extend prior work to suggest that the meaning of the fixated target word does not always replace the meaning that was accessed from the parafoveal preview.

Orthographic, Phonological and Semantic Preview Benefit for Chinese Children aged 7-8, 8-9, 9-10 and 10-11 Years

Yan, Guoli ; Liu, Min; Zhu, Meng; Sainan, Li; Yongsheng, Wang; Liversedge, Simon, P.
Academy of Psychology and Behavior, Tianjin Normal University ; Academy of Psychology and Behavior, Tianjin Normal University ; Academy of Psychology and Behavior, Tianjin Normal University ; Academy of Psychology and Behavior, Tianjin Normal University ; Academy of Psychology and Behavior, Tianjin Normal University ; School of Psychology ,University of Central Lancashire

Parafoveal preprocessing of upcoming words is a key aspect of fluent reading.
As a logographic language, Chinese has unique orthographic characteristics different from alphabetic languages. Given that information is more densely packed in Chinese than in alphabetic languages, more information may be available for processing to the right of fixation in Chinese, and therefore, the nature of parafoveal processing in Chinese children may be different from that in children reading alphabetic languages. Currently, evidence regarding the developmental pattern of parafoveal preprocessing in Chinese is limited.
In the present study, three boundary paradigm experiments were conducted to investigate the development of parafoveal orthographic, phonological and semantic processing in Chinese readers. Eye movements of primary school students and college students were recorded as they read sentences. In each experiment (orthographic, phonological and semantic) we adopted a mixed design in which we manipulated Preview Type (identical, related or unrelated preview) across School Grade (2-5).
The results were as follows: (1) Orthographic preview benefits were obtained by children from Grade 2 and older; (2) Phonological preview benefit occurred for children from Grade 4 and older; and (3) only children in Grade 5 showed a semantic preview benefit. The results suggest that the nature of parafoveal preview benefit changes with age. It appears that younger children exclusively engage in relatively shallow levels of parafoveal processing, whilst in older children parafoveal processing occurs to a deeper level of linguistic processing.
Keywords: parafoveal processing, reading development, orthographic effects, phonological effects, semantic effects

Preview effects in reading of Chinese two-constituent words and phrases

Chuanli Zang[1,2], Hong Du[2], Xuejun Bai[2], Guoli Yan[2] & Simon P. Liversedge[1]
([1] University of Central Lancashire; [2] Tianjin Normal University)

A considerable amount of research has investigated whether words are lexically processed serially or in parallel during reading of spaced alphabetic languages like English. In contrast, Chinese is a character-based, unspaced language. An intriguing question concerns how lexical processing in Chinese is operationalized across words, phrases or other linguistic units. Particularly, we were interested in processing of frequently occurring Multi-Constituent Units (MCUs), that is, linguistic units comprised of more than a single word, that might be represented lexically. This possibility might offer an explanation regarding why on some occasions lexical processing appears to operate serially, and on others words appear to be identified in parallel.
We manipulated the linguistic category of a two-constituent Chinese string (a word, a MCU, or a phrase), and the preview of its second constituent (identical or psedocharacter) using the boundary paradigm (Rayner, 1975) with the boundary located before the two-constituent string. For the first constituent analyses, a preview effect was obtained with shorter fixations for identical than pseudocharacter previews. Importantly, this effect was robust when the second constituent alongside the first formed a word or a MCU, but not a phrase. For the second constituent analyses, reading times were shorter for words and MCUs than phrases. These results suggest that frequently occurring MCUs are lexicalized and processed parafoveally as a single unit during reading. Apparently, lexical processing in Chinese can be operationalized over linguistic units that are larger than the word. The implications of these findings for current eye movement models will be considered.

Trilingual reading: The effect of cognates, “false friends”, and language proficiency

Angele, Bernhard ; Freitas Pereira Toassi, Pâmela; Slattery, Timothy J.; Nogueira Teixeira, Elisangela
Bournemouth University; Federal University of Ceará; Bournemouth University; Federal University of Ceará

There is ample evidence that multilinguals process cognates faster than non-cognates (Lemhöfer, Dijkstra, & Michel, 2004). However, it is not clear whether this facilitation effect is due to orthographic similarity or due to the semantic overlap between the representations of the cognate in each language. We present an eye-tracking study in which Brazilian Portuguese/Spanish/English multilinguals of different proficiency levels read English sentences containing cognates (e.g. “error”), “false friends” which appear to be cognates but have a different meaning (e.g. English “exit” and Portuguese/Spanish “êxito/éxito”, meaning “success”), and non-cognate control words. In order to force readers to rely more on top-down language knowledge, we introduced a condition in which the sentences were visually degraded. We found shorter gaze durations on the target word when it was either a cognate or a false friend compared to the non-cognate control, but no significant difference between cognates and false friends, suggesting that, in early processing, visual familiarity with the cognate/false friend was responsible for the facilitation. However, there was a significant difference between cognates and false friends in total viewing time, indicating that readers are sensitive to the semantic mismatch in later processing. This effect was stronger the higher a participant’s proficiency in English and Spanish was. Finally, there was a main effect of the visual noise manipulation, but it did not interact with the cognate manipulation.

References

Lemhöfer, K., Dijkstra, T., & Michel, M. (2004). Three languages, one ECHO: Cognate effects in trilingual word recognition. *Language and Cognitive Processes*, 19(5), 585–611. https://doi.org/10.1080/01690960444000007

What is the effect of foveal load on preprocessing the upcoming word?

Stefan Hawelka
Centre for Cognitive Neuroscience
University of Salzburg

In a recent study (Findelsberger, Hutzler & Hawelka, 2019), we re-investigated the effect of foveal load on parafoveal preprocessing the upcoming word (i.e., the foveal load hypothesis; Henderson & Ferreira, 1990). We induced different levels of foveal load by high and low frequency pretarget words and – with the boundary paradigm (Rayner,1975) – we manipulated the preview of the target word in two different ways. In Experiment 1, we used the traditional letter masks. In Experiment 2, we visually degraded the preview of the target word (e.g., Marx et al., 2016, 2017). Contrary to expectation, we did not find a LOAD by PREVIEW interaction with traditional letter masks, but with visually degraded previews. However, the effect size of the significant interaction in Experiment 2 was small. Furthermore, the visual degradation of the previews in Experiment 2 elicited a unusually high awareness of the display change which could be a confounding factor. In a next study we followed-up the mixed evidence for the effect of foveal load with two objectives in mind. First, we aimed at reducing the display change awareness for the visual degradation. This we tried to achieve with lowering the stimulus-background contrast. Second, we hypothesized that manipulating word frequency in the native tongue of the participants is too weak a manipulation for inducing notable differences in foveal load. Thus, we had native German readers read English sentences which should induce much higher levels of load. A preliminary analysis of the data of this ongoing investigation indicates that neither the reduction of the display change awareness nor the induction of higher levels of load results in compelling evidence for the foveal load hypothesis. The findings will be discussed in the light of other recent investigations of the foveal load hypothesis (e.g., Veldre & Andrews, 2018).

References

Findelsberger, E., Hutzler, F. & Hawelka, S. (2019). Spill the load: Mixed evidence for a foveal load effect, reliable evidence for a spillover effect in eye-movement control during reading. Attention, Perception & Psychophysics.
Marx, C., Hutzler, F., Schuster, S., & Hawelka, S. (2016). On the development of parafoveal preprocessing: Evidence from the incremental boundary paradigm. *Frontiers in Psychology, 7*, 1-13.
Marx, C., Hawelka, S., Schuster, S., & Hutzler, F. (2017). Foveal processing difficulty does not affect parafoveal preprocessing in young readers. *Scientific Reports, 7*, 1-11.
Rayner, K. (1975). The perceptual span and peripheral cues in reading. *Cognitive Psychology, 7*, 65-81.
Veldre, A., & Andrews, S. (2018). How does foveal processing difficulty affect parafoveal processing during reading? *Journal of Memory and Language, 103*, 74-90.

Visual behavior in real and virtual scenarios (Plenary room | 14:00 – 16:00)

Concealed information revealed by involuntary eye movements on the fringe of awareness in a mock terror experiment

Gal Rosenzweig and Yoram S Bonneh
University of Haifa; Bar-Ilan University

The involuntary eye movements during fixation are typically inhibited following stimulus onset (Oculomotor Inhibition, OMI), depending on its saliency and anticipation. We have recently reported on early and prolonged OMI in response to barely visible familiar faces in passive viewing (Rosenzweig & Bonneh, 2019). Here we show that this OMI effect can be used for a concealed information test (CIT). In a "mock terror" experiment, 13 volunteers made a choice, hidden from the experimenters, of a "terror target" (one-of-eight), which they learned (face, name and residence) watching text and videos (~20min), while 7 "innocent" volunteers pre-learned nothing. All participants were then passively exposed to ~20 min of repeated (1Hz) brief presentations of barely visible (masked) stimuli, that included the 8 potential "terror targets", as well as a universally familiar face among unfamiliar faces to obtain an "individual recognition pattern". High-resolution eye-tracking data were analyzed for microsaccade and blink inhibition, as well as for pupil size and eye position modulations. We found that a microsaccade OMI analysis that incorporated the individual recognition pattern and all three identifying items (face, name and residence), yielded 12/13 (92%) correct identification of the terror target, which improved to 100% with the additional oculomotor parameters. There was a lower success rate of 85% (no false-alarms, 3 misses) in detecting the "terrorists" among all 20 participants using all parameters. Our results suggest a novel approach to CIT, based on involuntary oculomotor response to barely visible stimuli, individually tailored, and with high accuracy and theoretical resistance to countermeasures.

Gaze behaviour of experienced and inexperienced beach lifeguards – An in situ study

First author: **Pieter Vansteenkiste**
Co-authors: **Matthieu Lenoir Jan Bourgois**
Ghent University, Department of Movement and Sports Sciences
E-mail : Pieter.vansteenkiste@ugent.be

For lifeguards, recognising a swimmer in trouble is not only the start, but also the key factor in the rescue process. Due to task and environmental constraints, spotting a drowning person on time is a very challenging task. Although multiple reports show that lifeguards outperform non-lifeguards in their surveillance task, this has not been reflected in a different visual behaviour so far. However, the visual behaviour of lifeguards has only been tested in a lab environment, and never 'in-situ'. The current study is the first to describe the natural gaze behaviour of experienced and non-experienced beach lifeguards while they were on active duty. The gaze behaviour of six inexperienced and seven experienced lifeguards was recorded for 45 minutes while they were on active duty at the beach. Results showed that fixation duration of experienced lifeguards was longer and more variable than that of inexperienced lifeguards, and that experienced lifeguards adapted their gaze behaviour more to the distribution of patrons. No significant differences were found in general attention distribution, nor for the average duration each zone was left unattended. Results are in line with mechanisms of perceptual learning and suggest that experienced lifeguards process visual information at a higher level (top-down), while inexperienced lifeguards monitored the scene in a more generic way (bottom-up). These findings provide further insights into the perceptual processes of beach lifeguards and could lead to improved lifeguard training programs.

Gaze Synchrony
A Measure of Gaze Overlap in Multiple Mobile Eye Tracking

Enrique Garcia Moreno-Esteva[1], Jessica F. A. Salminen-Saari[1], Miika Toivanen[2], Markku S. Hannula[1]
1) University of Helsinki
2) See True Technologies

Measuring gaze overlap or joint visual attention between two or more individuals has been challenging, and up until now, the solutions that exist provide aggregate information or information which requires interpretation, such as visual aids. Furthermore, such measures have been limited to two participants. Here we discuss a new measure of joint visual attention or gaze overlap, called gaze synchrony, which (1) is quantitatively precise and consistent across data sets; (2) is dynamic - a function of time; (3) works well with two or more participants. We have tested the method with hours of gaze tracking data gathered in classrooms.
To compute the measure, dwell data sequences are synchronized and a raw score of overlap for the participant group is computed in a sliding window of about 10 second for each synchronization point; the longest common sequence length of the data in the group of windows is computed. A mean for the same number of randomly generated data is also computed, and the standard score of the raw score relative to the mean provides the gaze synchrony measure.
We evaluate the effectiveness of the measure in a case study exploring the dynamics of collaborative behavior with a group of students wearing mobile eye tracking devices and working on an open-ended, non-routine, mathematics problem solving session in a classroom. Throughout the episode in which three participants wear mobile gaze trackers, high gaze synchrony correlates with significant events related to the problem session activity related, in particular, with the verification of solutions.

Human Agent Interaction in Virtual Reality: An Experimental Investigation of Deictic Gaze in a Joint Attention Setting

Acarturk, Cengiz ; Yilmaz, Efecan; Fal, Mehmetcan; Semin, Gun R.

Orta Dogu Teknik Universitesi; Orta Dogu Teknik Universitesi; Orta Dogu Teknik Universitesi; ISPA Instituto Universitário, Lisbon

Language and vision are two complementary modalities that employ deixis in communication. Verbal deictic expressions are necessarily accompanied by other modalities, such as pointing by gestures or pointing by gaze. The presence of multiple modalities facilitates disambiguating spatial reference to objects in the environment. We report an experimental study that investigated the deictic function of gaze in avatar agents. For this, we designed a virtual reality (VR) environment, which provided a joint attention setting between human participants and avatars. The participants responded to a set of questions that involved explicit or implicit referring expressions about the spatial locations of objects on a table, while their eye movements were recorded. The explicit statement of referring expressions, such as “what is the object at the left bottom?” revealed different patterns in terms of accuracy, response times and gaze allocation of the participants, compared to the implicit statements of referring expressions, i.e. “what is the object there?” that was accompanied by deictic gaze. The findings also showed that the avatar morphology might have a significant influence on the results. Overall, our findings show that the study of deictic gaze in virtual reality environments has the potential to expand our knowledge about mechanisms that underlie human spatial cognitive abilities as well as our knowledge about the interaction between humans and agents in VR.

Investigation of Quiet Eye in Biathlon

Hansen, Dan Witzner ; Heinrich, Amelie; Cañal-Bruland, Rouwen
IT University Copenhagen; nsitute of Sports Science,Martin Luther University Halle-Wittenberg; Institute of Sport Science, Friedrich-Schiller-University Jena

The duration of the "Quiet-Eye"(QE) – the final fixation before the initiation of a critical movement – seems to be linked to better perceptual-motor performances. The aim of this study was to examine the effect of QE on shooting performance in biathlon. Gaze and performance data (shooting accuracy) of 19 members of the German national junior and senior teams (21.14 +- 2.71) were collected during performance test protocols. The tests consisted of roller skiing on a treadmill (four increasing intensity levels) and shooting series of five shots after each level. A dedicated gun-mounted eye tracker was developed as a means to obtain reliable gaze data without significantly influencing the athletes. The eye tracker did not need user calibration or required the athletes to wear the eye tracker. The combination of sensors for eye tracking, gaze estimation and measuring trigger force ensured minimal interference with normal testing procedures and enabled precise measures of the final action (trigger) and fixation duration. A binomial GLM did, contrary to previous finding, not yield support of the importance of a long fixation duration on shooting accuracy in biathlon. Since a long fixation duration seems not to influence performance further studies are needed to fully understand the importance of vision in biathlon.

Visual attention during online grocery shopping

Gidlöf, Kerstin ; Holmberg, Nils; Annika, Wallin
Aarhus University; Lund University; Lund University

The environment in which we make our decisions influences our decision process, our perception of the options and our visual attention. Visual attention is known to greatly impact consumers' choices, and naturally, supermarkets, brand owners and product designers go to great lengths in order to gain consumers' visual attention. In a previous study we used mobile eye trackers in order to monitor consumers' visual attention in supermarkets. We found that salient features did get consumers visual attention, but in particular so if the consumer goods also fit consumers' product preferences. Here we present a different study in which we ask consumers to buy similar products in a real online store compared to the physical store in our previous study. Previous research indicates that consumers transfer their shopping behaviour from physical stores to online ones. We find, however, that consumers' visual attention works surprisingly different online compared to the physical store. This despite the fact that we asked consumers in both studies to use the same amount of money to buy products in the same categories, and despite the fact that the encountered the same options and package designs in both environments. In particular, saliency did not attract consumers' attention the same way in the online compared to the physical store. Understanding the interaction between visual attention and the visual environment in food purchases is especially interesting since the visual domain is so important and influential in these decisions. Understanding how the context affects these decisions is therefore of great importance.

Memory (Room 10 | 14:00 – 16:00)

How holding an item in visual working memory affects pre-saccadic shift of attention.

Soazig Casteau, Charlotte Bush, Mary Chalkley, Natalie Rogerson and Daniel T. Smith
Durham University

Biased competition theory assumes that shift of attention is biased towards visual stimuli that matched the content of the visual working memory (VWM) and this bias might be due to a competition between eye-movement motor plans and memory object location. Here, we measured the effect of holding an item in VWM on pre-saccadic shift of attention by measuring attention when the goal of an eye-movement is congruent or incongruent with the location of an object sharing properties with the VWM content. Participants performed an eye-movement towards a previously specified location (Saccade Target, ST) and make a discrimination decision (Discrimination Target, DT). They also had to retain a visual feature in VWM for the whole duration of the trial (Memory Target; MT). There were four different conditions: 1) MT congruent with ST and DT, 2) MT congruent with DT but incongruent with ST, 3) MT congruent with ST but incongruent with DT, 4) MT incongruent with ST and DT. As expected, pre-saccadic attentional facilitation was greatest when the VWM content, the saccade direction and the discrimination location were all congruent, and abolished when ST, DT and MT were at different locations. Critically, when the ST and DT were in the same location but the MT at a different location pre-saccadic attentional facilitation was significantly reduced, suggesting competition between the saccade goal and the MT. Our results suggest that, the attention bias linked to the content of VWM can be understand as a competition between different saccade goals in the oculomotor system.

Verbal working memory load affects fixations duration during visual search

Velichkovsky, Boris
Moscow State University

Introduction. Visual search involves eye movements to scan the display. The distribution of fixation durations can reveal important information about the visual search process.
Method. 16 subjects (14 female) aged 20 to 25 years with normal vision took part in the study. They searched for humans in photographs of urban scenes in two conditions counterbalanced across participants. In the WM load condition subjects maintained four random digits in WM during the search task. No load on verbal WM was imposed in the other condition. Eye movements were registered with the Eyelink 1000 system at 250 Hz.
Results. Mean fixation durations didn't differ significantly between conditions ($t(15)=-0,39$, $p>0,05$). Individual fixation duration distributions in every condition were fitted to the exponential-gaussian distribution using the GAMLSS package of the R computing environment. Verbal WM load increased the mean of the Gaussian component of the fitted distributions (mu, $t(15)=-2,33$, $p<0,05$) and their dispersion (sigma, $t(15)=-3,95$, $p<0,05$). These results suggest a general increase in the complexity of the visual search task under WM load. WM load decreased the tau parameter characterizing the extent of the right tail of the exponential component of the fitted distribution ($t(14)=2,34$, $p<0,05$). This suggests a lower probability of "long" fixations (over 500 ms) under verbal WM load.
Conclusions. Imposing a load on verbal WM leads to substantial changes in the dynamics of fixations during visual search task suggesting the reliance of eye gaze control on shared cognitive resources.

This research was supported by Russian Science Foundation, grant no. 19-18-00477.

Effects of prior knowledge on active vision and memory in younger and older adults

Jordana S. Wynn, Jennifer D. Ryan, & Morris Moscovitch
Rotman Research Institute/ University of Toronto

Real-world visual search relies on schemas, knowledge networks built up from multiple repeated experiences (Gilboa & Marlatte, 2017), to guide viewing behavior (Loftus & Mackworth, 1978; Henderson, 2017), and the ability to inhibit those schemas when their predictions fail (Vo & Wolfe, 2013). Relative to younger adults, older adults have difficulty inhibiting schemas, even when that knowledge is detrimental to the task at hand (Hasher & Zacks, 1988; Umanath & Marsh, 2014). Yet, the consequences of these age-related changes for visual search remain unclear. In the present study, we used eye movement monitoring to investigate whether over-reliance on prior knowledge (i.e., schemas) alters the gaze patterns and performance of older adults during visual search. Younger and older adults searched for target objects in congruent or incongruent locations in real-world scenes. As predicted, targets in congruent locations were detected faster than targets in incongruent locations, and this effect was enhanced in older adults. Analysis of viewing behavior revealed that prior knowledge effects emerged early in search, as evidenced by initial saccades, and continued throughout search, with greater viewing of congruent regions by older relative to younger adults, suggesting that schema biasing of online processing increases with age. Finally, both younger and older adults showed enhanced memory for the location of congruent targets and the identity of incongruent targets, with schema-guided viewing during search predicting poor memory for schema-incongruent targets on both tasks. Our results provide novel evidence that older adults' over-reliance on prior knowledge has consequences for both active vision and memory.

Gilboa, A., & Marlatte, H. (2017). Neurobiology of schemas and schema-mediated memory. *Trends in Cognitive Sciences*, 21(8), 618-631. doi:10.1016/j.tics.2017.04.013
Hasher, L., & Zacks, R. T. (1988). Working memory, comprehension, and aging: A review and a new view. The Psychology of Learning and Motivation: *Advances in Research and Theory*, 22, 193–225.
Henderson, J. M. (2017). Gaze control as prediction. *Trends in Cognitive Sciences*, 21(1), 15-23. doi:10.1016/j.tics.2016.11.003
Loftus, G., & Mackworth, N. (1978). Cognitive determinants of fixation location during picture viewing. *Journal of Experimental Psychology-Human Perception and Performance, 4*(4), 565-572. doi:10.1037//0096-1523.4.4.565
Umanath, S., & Marsh, E. J. (2014). Understanding how prior knowledge influences memory in older adults. *Perspectives on Psychological Science, 9*(4), 408-426. doi:10.1177/1745691614535933
Võ, M. L., & Wolfe, J. M. (2013). The interplay of episodic and semantic memory in guiding repeated search in scenes. *Cognition, 126*(2), 198-212. doi:10.1016/j.cognition.2012.09.017

The hippocampus supports binding of visual information across gaze fixations

Zhong-Xu Liu[1,2,3], **R. Shayna Rosenbaum** [2,3,4], **& Jennifer D. Ryan** [2,3,5,6]

1 *Department of Behavioral Sciences, University of Michigan-Dearborn, Dearborn, Michigan, USA*

2 *Rotman Research Institute, Baycrest, Toronto, Ontario, Canada;*

3 *Vision: Science to Applications (VISTA) Program and 4 Department of Psychology, York University, Toronto, Ontario, Canada;*

5 *Department of Psychology and 6 Department of Psychiatry, University of Toronto, Toronto, Ontario, Canada*

We use gaze fixations to freely explore the visual world1, and the amount of visual exploration is related to subsequent memory2. The hippocampus plays a crucial role in forming memories. An open question for investigation is whether the hippocampus binds visual information across individual gaze fixations3. We recently found that the hippocampus was activated more strongly when an increasing number of fixations were deployed to face stimuli, and this relationship between hippocampal activation and visual exploration was weaker in older adults, who also showed weaker memory effects. These findings suggested that the hippocampus may form memory representations by binding visual information from individual fixations4,5. However, given that participants' eye movements were measured in natural viewing tasks in previous studies4,5, the evidence to date is correlational in nature. To experimentally manipulate the relationship between visual exploration and hippocampal activity, in the current fMRI study we asked participants to either freely explore scenes or fixate on a single location. We found that the hippocampus was activated more strongly, and the memory performance was better, in the free than fixed viewing condition, and differences between the conditions were stronger when participants were processing meaningful scenes compared to meaningless scrambled images. In this talk, I will describe our recent studies that support the idea that the hippocampus binds visual memory representations online, across gaze fixations. This line of research calls for researchers to consider individual gaze fixations, instead of individual stimuli, as the basic unit of information on which the (hippocampal) memory system works.

References:

1. **Henderson, J. M.** Human gaze control during real-world scene perception. *Trends in Cognitive Sciences 7*, 498–504 (2003).
2. **Chan, J. P. K., Kamino, D. & Ryan, J. D.** Can changes in eye movement scanning alter the age-related deficit in recognition memory? Front. *Psychology 2, 92* (2011).
3. **Voss, J. L., Bridge, D. J., Cohen, N. J. & Walker, J. A.** A closer look at the hippocampus and memory. *Trends in Cognitive Sciences 21*, 577–588 (2017).
4. **Liu, Z.-X., Shen, K., Olsen, R. K. & Ryan, J. D.** Visual sampling predicts hippocampal activity. *J. Neurosci. 37*, 599–609 (2017).
5. **Liu, Z.-X., Shen, K., Olsen, R. K. & Ryan, J. D.** Age-related changes in the relationship between visual exploration and hippocampal activity. *Neuropsychologia 119*, 81–91 (2018).

Behavioural and pupillometric evidence for feature-specific resources in visual short-term memory

Doug JK Barrett[1] & Michael Pilling[2]

1. *Department of Neuroscience, Psychology and Behaviour, University of Leicester, LE1 7HA, UK.*
2. *Department of Psychology, Health and Professional Development, Oxford Brookes University, OX3, 0BP.*

Independent-resource models of visual short-term memory (VSTM) predict an inverse relationship between recall accuracy and the number of values for separate visual features (e.g., Wang et al., 2017). Slot models in contrast, predict all-or-none recall of separate features for objects that are encoded in VSTM (e.g., Luck & Vogel, 1997). The current study contrasted these predictions using a change detection task for objects that varied in their colour and orientation. Observers were cued to attend colour or orientation and the distribution of the attended and unattended feature was varied across objects in the display. Observers' accuracy to change on each dimension was equated using a staircase procedure. Comparisons of d' indicated change-discriminability was sensitive to the distribution of the attended but not the unattended feature. Pupil dilation during the retention period was i) larger on attend-colour than attend-orientation trials, ii) sensitive to the distribution of the attended feature across objects and iii) negatively associated with d'. These findings are consistent with independent-resource models of VSTM. The data also demonstrate dependencies between changes in pupil dilation during the retention of non-spatial features, and signal theoretic indices of change-discriminability, for objects that vary on attended and unattended features.

Revealing concealed information via eye movements: the promise of the short- term memory task

Dayan, Oryah chen ; Nahari, Tal; Ben-Shakhar, Gershon; Pertzov, Yoni
Hebrew Univeristy of Jerusalem

The concept of the eyes as a window to an inner world is commonly acknowledged, but only recently has it started to gain a scientific justification. Through a series of studies, we provide a new framework for such an interplay between personal experience and eye movements, across different tasks and diverse types of stimuli. In all of these studies, the visual design of the paradigm was similar (i.e., a parallel display of four items, one of them personally familiar to the participant). Yet, we manipulated the task demands and the instructions to the participants. Our results indicate that task demands are a crucial factor when formulating the interaction between familiarity and gaze behavior; when participants freely viewed the display, they preferentially gazed at the familiar item. However, when they were asked to memorize the items, this preference tendency was evident only at the beginning of the trial, followed by a strong tendency to avoid the familiar item. Task demands also influenced the ability to voluntarily control the visual exploration; while instructions to deploy gaze equally to all faces led to a diminution of the preference effect, the avoidance effect was robust even under such instructions. By using this pattern of preference and avoidance, we could accurately classify between familiar and unfamiliar stimuli. This classification ability is highly needed in the fields of security and law enforcement. Accordingly, this line of research provides a unique opportunity to utilize theoretical knowledge regarding memory guided-gaze for solving real-world problems.

Poster Presentations
20th European Conference on Eye Movements

Poster Session I (Monday, August 19th. 16:00 – 18:00)

APPLIED RESEARCH I

Poster 01

Don't Rely on the Pupils: Cognitive load indication in student and expert dentists

Nora Castner, *University of Tübingen, Sand 14, 72076 Tübingen, Germany, castnern@informatik.uni-tuebingen.de*
Tobias Appel, *University of Tübingen, Sand 14, 72076 Tübingen, Germany, tobias.appel@uni-tuebingen.de*
Thérése Eder, *Leibniz-Institute für Wissensmedien, Schleichstr.6, 72076 Tübingen, Germany, tf. eder@iwm-tuebingen.de*
Juliane Richter, *Leibniz-Institute für Wissensmedien, Schleichstr.6, 72076 Tübingen, Germany, j.richter@iwm-tuebingen.de*
Constanze Keutel, *University Hospital Tübingen, Osianderstr.2-8, 72076 Tübingen, Germany constanze.keutel@med.uni-tuebingen.de*
Katharina Scheiter, *Leibniz-Institute für Wissensmedien, Schleichstr.6, 72076 Tübingen, Germany k.scheiter@iwm-tuebingen.de*
Enkelejda Kasneci, *University of Tübingen, Sand 14, 72076 Tübingen, Germany, enkelejda.kasneci@uni-tuebingen.de*
Fabian Hüttig, *University Hospital Tübingen, Osianderstr.2-8, 72076 Tübingen, Germany fabian.huettig@med.uni-tuebingen.de*

Expert behavior extends from rapid information processing abilities [1], which relates to more structured working memory designated for their domain-specific tasks [2] and reflects cognitive load. Much of the literature has used pupil dilation changes as an indicator of cognitive load [3] in an n-back task. Our aim was to see how generalizable the pupil diameter is to cognitive load for expert dentists and students during dental X-Ray examination.
First year dental students (n = 50) and experts (n = 26) in the university clinic viewed panoramic radiographs with instructions to find areas that could be indicative of pathologies (see [4] for methods). We measured percent pupil dilation change from baseline from both groups as well as fixation and saccade behavior from the left eye after comprehensive pre-processing. Median pupil percent change and variance were then used as features for 10 different machine learning classifiers with a cost function for expert misclassification .
With pupil information alone, the average accuracy for all classifiers was 64.93%. However, for the same classifiers with the gaze feature in combination with the pupil dilation change were trained, the average accuracy was 80.52%, with one exception .
Therefore, using pupil dilation alone was not able to distinguish experts from students with high accuracy. These results stress that pupil diameter alone may not be a strong indicator or cognitive load [5], and that other features may be needed for better classification.

REFERENCES

[1] **Gegenfurtner, A., Lehtinen, E., & Säljö, R.** (2011). *Expertise differences in the comprehension of visualizations: A meta-analysis of eye-tracking research in professional domains*. Educational Psychology Review, 23(4), 523-552.
[2] **Ericsson, K. A., & Kintsch, W**. (1995). *Long-term working memory*. Psychological review, 102(2), 211.
[3] **Appel, Tobias, Christian Scharinger, Peter Gerjets, and Enkelejda Kasneci**. *"Cross-subject workload classification using pupil-related measures."* In Proceedings of the 2018 ACM Symposium on Eye Tracking Research & Applications, p. 4. ACM, 2018.
[4] **Castner, N., Kasneci, E., Kübler, T., Scheiter, K., Richter, J., Eder, T., ... & Keutel, C.** (2018, June). *Scanpath comparison in medical image reading skills of dental students: distinguishing stages of expertise development*. In Proceedings of the 2018 ACM Symposium on Eye Tracking Research & Applications (p. 39). ACM.
[5] **Van Gerven, P. W., Paas, F., Van Merriënboer, J. J., & Schmidt, H. G.** (2004). *Memory load and the cognitive pupillary response in aging*. Psychophysiology, 41(2), 167-174.

1. Due to the uneven class sizes.
2. The quadratic discriminant analysis had an accuracy of 54.79%

Poster 02

Eye tracking-based assessment of oculomotor and executive functioning in patients with advanced amyotrophic lateral sclerosis (ALS)

Elisa Aust[1], Sebastian Pannasch[2], Sven-Thomas Graupner[2], Markus Joos[3], Daniel Liebscher[3], Katharina Linse[1,4], & Andreas Hermann[1,4,5,6]

1 *Dept. of Neurology, Technische Universität Dresden, Dresden, Germany*
2 *Engineering Psychology and Applied Cognition, Technische Universität Dresden, Dresden, Germany*
3 *Interactive Minds Research, Dresden, Germany*
4 *German Center for Neurodegenerative Diseases (DZNE), Research Site Dresden, Germany*
5 *Center for Transdisciplinary Neurosciences Rostock (CTNR), University Medical Center Rostock, University of Rostock, Rostock, Germany*
6 *Translational Neurodegeneration Section „Albrecht-Kossel", Department of Neurology, University Medical Center Rostock, University of Rostock, Rostock, Germany*
Email: elisa.aust@uniklinikum-dresden.de

For patients with advanced ALS, eye motions often remain the last preserved voluntary movements. Therefore, eye tracking devices are used as means for communication and to control the environment. They can also enable (neuro)psychological assessment but various oculomotor dysfunctions have been reported for ALS. Investigating oculomotor changes in ALS patients allows important insights into the neurodegenerative processes outside the motor neurons. Furthermore, it helps to better understand the cognitive deficits, which affect about one third of the patients and most frequently executive functions [1]. However, particularly for advanced disease stages only few findings on oculomotor and cognitive functioning exist. One major reason for this lack is that neuropsychological tests are based on verbal or motor output.

We aim to access oculomotor and executive functions in advanced ALS by using eye tracking technology. We therefore developed smooth pursuit, reactive pro- and anti-saccades tasks and for measuring executive functioning eye-tracking versions of the Trail making Test (eTMT) and Wisconsin Card Sorting Test. Results in the latter are interpreted based on norms. Influence of disease characteristics on performance on all tasks will be reported, as well as associations between parameters of oculomotor and cognition. Additionally, oculomotor parameters will serve as evaluation criteria of the validity of reaction time-based eTMT-results.

[1] **Proudfoot, M., Menke, R. A. L., Sharma, R., Berna, C. M., Hicks, S., Kennard, C., . . . Turner, M. R.** (2016). Eye-tracking in amyotrophic lateral sclerosis: A longitudinal study of saccadic and cognitive tasks. *Amyotroph Lateral Scler Frontotemporal Degener, 17*(1-2), 101-111.

Poster 03

Eye-movements in endoscopic ear surgery: Comparison of 2D and 3D endoscopes

Laura Niederhauser[1], MSc; Lukas Anschuetz[2], MD; Wilhelm Wimmer[4], PhD; Abraam Yacoub[2], MD; David Weibel[3], PhD; Fred Mast[3], PhD; Marco Caversaccio[2], MD

1 *Swiss Distance Learning University, Brig, Switzerland*

2 *Department of Otorhinolaryngology, Head & Neck Surgery, Inselspital, University Hospital and University of Bern, Switzerland*

3 *Department of Psychology, University of Bern, Switzerland.*

4 *ARTORG Center for Biomedical Engineering, University of Bern, Switzerland*

Examination of surgeons' eye motions showed that gaze patterns differ between levels of expertise and various kinds of tasks (Hermens, Flin, & Ahmed, 2013). However, little research has looked into differences between visualization techniques, such as endoscopes. Therefore, the aim of this study was to compare eye-movements of ear surgeons operating with an established 2-dimensional endoscope and a relatively new 3-dimensional endoscope. Sixteen participants performed two kinds of simulated ear surgery on a cadavric specimen, once with a 2D endoscope and once with a 3D endoscope. Thus, all subjects (5 specialists and 11 residents) performed four surgeries in total. The order of the surgeries was randomized and participants were wearing eye-tracking glasses at all times (SMI, Berlin, Germany). Results showed that fixation duration differed significantly between the two surgical tasks. Whereas this difference was the same for residents in 2D and 3D, it increased for specialists using the 2D endoscope. Thus, specialists had longer fixation durations while performing the surgical task with a 2D endoscope, yet they could not apply the same gaze strategy with the 3D endoscope. This suggests that even well adapted specialists require a learning curve when adopting a new technology.

Hermens, F., Flin, R., & Ahmed, I. (2013). Eye movements in surgery: A literature review. *Journal of Eye Movement Research, 6*(4), 1–11. https://doi.org/10.16910/jemr.6.4.4

Poster 04

Gender effects in viewing advertising banners on eye movements

Petružálek, Jan ; Šefara, Denis; Franěk, Marek; Mehliš, Honza
University of Hradec Králové
jan.petruzalek@uhk.cz, denis.sefara@uhk.cz, marek.franek@uhk.cz

There is evidence of gender differences in eye movement activity while observing visual stimuli. However, these findings come mainly from studies, where various kinds of erotic stimuli have been used (Lykins et al., 2007). There is only a limited amount of studies (e.g., Hwang et al., 2017) that documented gender differences in visual perception in online shopping. The objective of the present investigation was to examine whether banners that contain either image of male or image of the female are visually processed by men and woman in a different way. We supposed that males are more attracted than females by images of the opposite sex on a banner advertisement. In the present experiment series of banner advertisement were presented and gaze behavior was measured (N = 102, 52 females). Eye movements were recorded using Tobii X2-60 eye tracker. As predicted, it was found that males had a significantly higher mean a number of fixations and significantly higher mean fixation durations on the location of the banner, which contained the image with a woman. Simultaneously, they took less attention to the banner section that carried proper advertising information. A similar trend was observed in women participants, but the differences were not significant. These findings provide further evidences related to gender differences in eye-movements activity. Males are attracted more than females to opposite-sex figures. Implications for applying more efficient marketing strategies are discussed.

Poster 05

Hypervigilance to hostile cues dynamic scenes bullying: An eye tracking study

Mannan, Sabira
Kingston University

The aim of the current study was to examine whether individuals who were victims of childhood bullying differ in their overt attention to hostile cues to non-victims. Multiple lines of evidence suggest that individuals who are anxious are more vigilant to environmental threats than non-anxious individuals, in accordance with hyper-vigilance theory (Eysenck, 1992). In an extension to this theory it was hypothesised that childhood victims of bullying would fixate more quickly on bullies in a dynamic visual scene than non-victim individuals and would exhibit a higher dwell time on bullies compared to non-victims. Forty participants took part in an eye tracking study consisting of the viewing of 12 dynamic scenes (4 of direct bullying, 4 of indirect bullying and 4 neutral scenes). Videos were viewed without sound and ranged from 4 and 29 seconds in duration. Participants also completed the Interpersonal Reactivity Index (Davis, 1980) and the Friendship-Behaviour questionnaire (Crick & Grotpeter, 1995). There was no relationship between childhood victimisation and time to fixate bullies or victims within scenes. There was also no relationship between victimisation scores and total dwell time on bullies or victims. Participants who scored more highly on Personal Distress fixated victims more quickly and showed longer dwell times on victims. Participants with higher scores on perspective taking also exhibited longer dwell times on victims. The results are discussed in relation to social information processing theory.

Poster 06

Microsaccade suppression during antisaccade generation in the posner cueing paradigm

Sofia Krasovskaya[1], Arni Kristjansson[1,2], Joseph MacInnes[1]
1 *National Research University Higher School of Economics*
2 *University of Iceland*

The functional purpose of microsaccades is still debated. Spatial cueing paradigms (Posner, 1980) typically require fixational control, but this does not eliminate all oculomotor activity associated with the preparation of saccades in the cued direction (Engbert & Kliegl, 2003). During the antisaccade task (Halett, 1978) observers have to make saccades in the opposite direction to the onset of a cue. Planning and execution are therefore separate processes. We thus hypothesise that microsaccades will be reduced during the execution of antisaccade as compared to prosaccade trials. Twenty-two participants performed saccades or antisaccades in blocked or mixed trials. They participated in three blocks: a fixed saccade block, a fixed antisaccade block, and a mixed saccade - antisaccade block. In the saccade trials, a green fixation cross was displayed at screen centre, whereas during antisaccade trials the fixation cross was red, allowing participants to prepare the appropriate response (but not direction) prior to the target. The results of the study show a large latency cost for antisaccades compared to prosaccades and an additional cost of mixed blocks. In the blocked antisaccade trials the observers made fewer microsaccades. We believe this may be due to participants having enough time to prepare the top-down control of the oculomotor system, which leads to a predictable pattern for each participant. We also predict that in the mixed block participants have less time to prepare the top-down microsaccade suppression and test this by comparing data between the saccade, the antisaccade and mixed blocks.

References

Engbert, R., & Kliegl, R. (2003). Microsaccades uncover the orientation of covert attention. *Vision Research, 43*(9), 1035–1045.
Hallett, P. E. (1978). Primary and secondary saccades to goals defined by instructions. *Vision Research, 18*(10), 1279–1296.
Posner, M. I. (1980). Orienting of attention. *Quarterly journal of experimental psychology, 32*(1), 3-25.

Poster 07

Scene Cut Influence on Blinks and Saccades

Ioannis Agtzidis[1], Colin Clifford[2], Tamara Watson[3], Inga Meyhöfer[4], Markus Lappe[4], Rebekka Lencer[4], Michael Dorr[1]

1Techincal University of Munich, 2University of New South Wales, 3 Western Sydney University, 4 University of Münster

Several times per minute, blinks introduce abrupt changes to the visual input. Here, we use scene cuts in Hollywood movies to model such changes and investigate how they affect our visual behaviour. Specifically, we studied the time course of saccades and blinks after scene cuts in the studyforrest data set [Henke et al., 2016] which comprises of data from 30 subjects watching the Forrest Gump movie and includes manually annotated scene cuts. Blinks and saccades were automatically detected with the sp_tool [Agtzidis et al., 2016], which achieves close to human performance. For our analysis we used the original positions of the 868 abrupt movie cuts together with a randomly shuffled representation of those as baseline. The used metric was the timing of the first event after a scene cut.

Our results show that both saccades and blinks are similarly affected by scene cuts, but the time course differs for the two event types. In both cases, cuts initially lead to an inhibitory period, with a processing latency of about 100ms for saccades and 250ms for blinks. After about 40ms, an excitatory period leads to an increased number of saccades to reorient the gaze on the new stimulus content for about 300ms. For blinks, a less pronounced excitation-after-inhibition occurs after about 250ms and lasts for about a second. Afterwards, both blink and saccade rates return to normal.

To summarize, we here described the time course of the visual system's information maximization strategy in response to a change in the visual input.

References:

Hanke, Michael, et al. "A studyforrest extension, simultaneous fMRI and eye gaze recordings during prolonged natural stimulation." *Scientific data 3* (2016): 160092.

Agtzidis, Ioannis, Mikhail Startsev, and Michael Dorr. "Smooth pursuit detection based on multiple observers." *Proceedings of the ninth biennial acm symposium on eye tracking research & applications.* ACM, 2016.

Poster 08

Skilled players control the span of visual spatial attention effectively during anticipation tasks in sports: evidence from microsaccade dynamics

Kato, Takaaki; Saijo, Naoki; Yoneya, Makoto; Kashino, Makio

Keio University; NTT Communication Science Labs, NTT Corp.; NTT Communication Science Labs, NTT Corp.; NTT Communication Science Labs, NTT Corp.

In interactive sports such as ball games and martial arts, superior performance critically depends on the speed and accuracy in predicting the next move of a ball or opponent players. How do skilled players acquire relevant visual information effectively? In contrast to extensive studies on the gaze direction of players in such sports, few studies have examined the span of visual spatial attention. Inspired by previous studies showing the link between microsaccades and visual spatial attention (e.g., Engbert & Kliegl, 2003), we developed a novel method to estimate the span of visual spatial attention, based on the dynamic properties of microsaccades (Yoneya, et al., 2017). Taking advantage of the method, we examined how the skill level of players affects the attention span in making a prediction about the next event in a sport scene. In the behavioral experiment, skilled and sub-skilled college soccer players were asked to answer the kicked ball direction presented on a screen by pressing the button as fast and accurate as possible. The scene was occluded immediately after the kicking. The results indicated that the skilled soccer players generally reacted more quickly and accurately than the sub-skilled ones. Moreover, they exhibited lower microsaccade rate and larger damping factor during the anticipation task. Such properties of microsaccades were similar to those obtained when the scope of attention was set to be narrowly focused in a cue-target task. These results suggest that the skilled players control their scope of attention effectively to get accurate prediction and quick decision-making.

Poster 09

The Influence of Culture: Perspective Taking and Scene Perception

Yuxuan Guo[1], Dale Barr[2] and Sebastian Pannasch[1]

1 Chair of Engineering Psychology and Applied Cognition, Technische Universität Dresden

2 Institute of Neuroscience and Psychology, University of Glasgow

Email: yuxuan.guo@tu-dresden.de

Some recent evidence suggested that visual-cognitive processes may differ across cultures. Individuals from East Asian cultures outperformed people from Western cultures in taking different perspectives into account when resolving ambiguous social information (Wu & Keysar, 2007; Wu & Barr, 2013). In addition, cultural differences were also reflected in perceptual attention (Chua, Boland & Nisbett, 2005). Cross-cultural research proposed that the "collectivist" character versus "individualist" character in Eastern and Western cultures underlies differences in visual-cognitive operations. To further understand the nature of these cultural effects, we conducted two eye-tracking experiments to examine the cultural variation in perspective-taking and scene perception processes. In the perspective-taking task, we monitored and compared both eye movement patterns and behavioural performances between different language users (English versus Mandarin) to investigate how cultural/language experiences may influence the way people take other persons' perspectives into account in socialization practices. Our results indicate that subjects from different cultures share a common pattern in resolving ambiguous information, no group showed greater ability in integrating others' perspectives with incoming referential expression. In the scene viewing task, we analysed attention allocation strategies and the time course of subjects' eye movements during the free scene exploration. The results demonstrate that subjects from different cultures did not allocate attentional resources differently as they perceived the scenes. Both cultural groups encoded more visual information on the foreground objects than on the background and revealed a systematic increase in fixation durations over the time course of scene perception.

Keywords: Eye movements, Perspective taking, Scene perception, Cultural difference, Fixations, Saccade amplitude

References

Chua, H., Boland, J. & Nisbett, R. (2005). From The Cover: Cultural variation in eye movements during scene perception. *Proceedings of the National Academy of Sciences, 102*(35), 12629-12633.

Pannasch, S., Helmert, J.R., Roth, K., Herbold, A.-K. & Walter, H. (2008). Visual Fixation Durations and Saccadic Amplitudes: Shifting Relationship in a Variety of Conditions. *Journal of Eye Movement Research, 2*(2):4, 1-19.

Wu, S. & Keysar, B. (2007). The Effect of Culture on Perspective Taking. *Psychological Science, 18*(7), 600-606.

Wu, S., Barr, D., Gann, T. & Keysar, B. (2013). How culture influences perspective taking: differences in correction, not integration. *Frontiers in Human Neuroscience, 7.*

Poster 10

Time-continuous eye movement analysis in a decision-making task

Federica Conti and Anna Montagnini

Institut de Neurosciences de la Timone (INT), Aix-Marseille University and CNRS

Motion direction discrimination of a random dot kinematogram (RDK) is a widely used experimental paradigm in the study of visual decision-making. We implemented a new variant of this decision task, whereby participants were not constrained to keep central fixation during the RDK display. The eye movements of eight healthy volunteers have been recorded during the whole duration of the decision task, both in equiprobable conditions and in the presence of a strong bias (90%) favoring one of the alternative outcomes. Task difficulty varied over four distinct coherence levels, namely, 0.05, 0.10, 0.20 and 0.40. Subjects' answers were communicated via a saccade towards one of two targets displayed at a 45° angle from the corresponding motion direction. Preliminary results show that, generally, in the early phase of RDK display, smooth eye movements velocity is directly proportional to motion coherence. Moreover, an analysis of the pre-saccadic epoch reveals that for many subjects (but not all) decision accuracy (correct vs incorrect trials) could be predicted above chance based on the average smooth velocity. Finally, quite unexpected is the overall effect of the direction bias, as demonstrated by a significantly higher error rate, and a frequently slower reaction time with respect to the unbiased condition. Very weak or no oculomotor anticipation was observed in the biased condition, whereas smooth eye movements during the RDK motion epoch were generally coherent with the behavioral performance, including in the frequent error trials.

Poster 11

Use-dependent plasticity in assistive interfaces: Gaze-typing improves inhibitory control

Souto, David ; Marsh, Olivia; Paterson, Kevin B.
University of Leicester

Gaze-controlled computing is a rapidly developing technology that is invaluable the context of assistive communication and may soon become mainstream in the entertainment industry (e.g. gaming). However, we know surprisingly little about the cognitive and oculomotor learning required for individuals to use gaze-controlled technology effectively, including for gaze-typing. In particular, users must adapt to the conflict between using gaze for normal looking and using gaze as a pointing device, often described as the “Midas touch” problem (i.e. turning everything you touch into gold). To assess the role of inhibitory control in solving this conflict we tested novice gaze-typists’ saccadic inhibition using the anti-saccade task at the beginning and end of five training sessions. During each session, participants wrote 100 high frequency six-letter words using a virtual keyboard, selecting letters sequentially by placing a cursor over each selection. One group (N=20) controlled the cursor with their eye gaze and the other with the mouse (N=20). The time required for the cursor to dwell on a selection was modified adaptively to equate letter-selection difficulty across participants and selection modes. The results showed that anti-saccade latencies were reduced more in the gaze-control compared to the mouse-control group by the end of the five sessions, while anti-saccade errors were maintained at the same level for both groups. The results therefore show improved inhibitory control in the gaze-typing group. We conclude that inhibitory control of saccades is implicated in solving the Midas touch problem during gaze-typing.

Poster 12

Using Gaze-Derived Measures to Contrast Mental and Physical Rotation Processes

Bertel, Sven; Wetzel, Stefanie
Flensburg University of Applied Sciences

Recent studies [e.g. 1,2] have provided novel, process-based analyses of mental and of physical (i.e., manual) rotation of Shepard and Metzler-type figures [3]. Similarities between the two include comparable angular disparity effects and comparable final angular offsets. Here, we advance the analysis by turning to gazed-derived measures obtained across the course of trials. In a within-subject design, students from STEM fields solved tasks as mental and as physical rotation problems. Tasks included both problems in which figures were the same and problems in which figures were different. Time course data on mean fixation duration and mean saccade amplitude was gathered. Our interpretations focus on mental loads and processes. While both measures provide evidence for higher loads in different-figure than in same-figure tasks, evidence for loads in mental and physical rotation tasks is less unambiguous. We further contrasted our data with what can be expected based on problem solving phases of classic procedural models of mental rotation. The results point to specific similarities and differences between mental and physical rotation that nicely add to previous findings.

[1] **Gardony, A. L., Taylor, H. A., & Brunyé, T. T.** (2014). What does physical rotation reveal about mental rotation? *Psychological Science, 25*(2), 605-612.
[2] **Bertel, S., Wetzel, S., & Zander, S.** (2016). Physical touch-based rotation processes of primary school students. *In Spatial Cognition X* (pp. 19-37). Springer, Cham.
[3] **Shepard, R. N., & Metzler, J.** (1971). Mental rotation of three-dimensional objects. *Science, 171*(3972), 701-703.

Poster 13

Viewers' Sensitivity to Camera Motion during Saccades in a Virtual Environment

Keyvanara, Maryam; Allison, Robert
Yok University

Gaze-contingent displays use real-time eye movement data to adjust the display content according to user's gaze. Display updates must happen fast enough to prevent the user from noticing them. Saccadic suppression helps hide these updates. The aim of this study was to investigate which image transformations are less perceptible and hence more applicable during saccadic suppression periods. We designed our experimental environments in Unity3D and used an Eyelink1000 to sample the participants' gaze in real time. Participants viewed 3D scenes in which the camera panned from left to right at a constant rotational velocity. During this motion they made a horizontal (lrightward) or vertical (downward) saccade during which a sudden movement of the camera transformed the image of the scene. Camera movements were one of 6 translation and 4 rotational directions. Following the trial participants indicated the direction of the change in a 2AFC task. Discrimination thresholds for each type of transformation were estimated using an adaptive procedure to fit a Weibull psychometric function. During both horizontal and vertical saccades, thresholds were higher for horizontal translational and rotational camera movements than for other transformations. Further experiments are being conducted to determine if this generalizes but the current results imply that the direction of camera motion affects the detectability of camera transitions during saccades. Understanding the relationship between on-going movements and the detectability of a sudden transsaccadic change can help provide a better user experience for users of VR that hide graphical updates when they generate a saccade.

Poster 14

Visual information sampling at the zebra crossing: Gaze behaviour in speed and time-to-arrival judgements

Jennifer Sudkamp[1], Mateusz Bocian[2], David Souto[1]

1 *Department of Neuroscience, Psychology and Behaviour, University of Leicester, UK*

2 *Department of Engineering, University of Leicester, UK*

Crossing the road safely requires an estimation of the speed and time-to-arrival of oncoming traffic. In naturalistic traffic scenes, gaze location within a vehicle influences its perceived speed (Clark et al., 2017). However, it is unclear how pedestrians spontaneously sample information when making time-to-arrival or speed judgements. We tested how information sampling interacts with perceptual judgements using video sequences that simulated an approaching vehicle from a pedestrian's view at a zebra crossing. In separate sessions, participants performed a 2IFC task, in which they identified the vehicle that was moving faster (speed task) or would arrive earlier (time-to-arrival task). After 3 s the approaching vehicle disappeared, while the scene remained visible for 2 s before participants responded. Gaze behaviour was similar during both tasks. While the vehicle was visible, gaze mainly pursued the centre of the vehicle's front plane. After the vehicle disappeared, visual strategies differed between participants. For half of the participants (n = 4) the number of fixations towards the crossing area increased. The other participants tracked the vehicle's extrapolated trajectory. The use of the two visual strategies were idiosyncratic rather than task-dependent, since only one participant alternated between strategies in different tasks. For time-to-arrival judgements, tracking the extrapolated trajectory resulted in a higher proportion of correct responses. For speed judgements, the choice of visual strategy showed no association with performance. The results suggest that observers do not adjust their gaze behaviour to different perceptual tasks, although spontaneous gaze strategies may impact perceptual judgements about oncoming traffic.

Poster 15

Visual scanning of places and non-places

Bałaj Bibianna, Strzałkowski Mateusz, Lewicka Maria, Lubiński Alex
Nicolaus Copernicus University in Torun

Within human geography and environmental psychology place is defined as a meaningful location and is often contrasted with locations that have no meaning, called non-places. Classic works on place define meaningfulness of places through: authenticity, uniqueness, enclosure, and historical continuity. Meaningful places have a fixed, stable identity and are described as “existential insidedness”, offering rest rather than movement. Contrarily, non-places are: uniform, commodified, and amorphous “spaces of flow”, without identity and history, as exemplified by highways, airports, degraded urban areas, and uniform commercial strips.
We predicted that people process meaningful scenes more deeply than scenes that are meaningless, and because of the focusing, meaningful elements scenes are explored more locally and their visual scan path is more focused. The research involved 34 participants watching set of 50 pictures of mostly urban scenes divided into meaningful and meaningless settings (places and non-places) on the basis of our preceding study in which the scenes were classified on a number of the meaningfulness-related dimensions such as historicity, enclosure, distinctiveness etc. The task was to click when participant caught the “gist” of the perceived scene (eye movements were recorded). No difference was observed between places and non-places in the time of catching the „gist” of the scene. As expected, we observed higher amplitudes of saccades and longer scan path for non-places comparing to places. No differences in the depth of processing measured by mean fixation time between places and non-places were observed, but it is worth testing more engaging task than “gist” assessment.
Key words: places and non-places, visual scanning, global-local scanning, meaningful scenes

Poster 16

Looking at Learning Spaces: Linking Space Properties and Visual Attention

Marco Rüth, Nicolas-Alexander Seiffert, Daniel Zimmermann, and Kai Kaspar
Department of Psychology, University of Cologne, Germany
marco.rueth@uni-koeln.de

Learning experiences in physical space are accompanied by an interplay of pedagogical, technological, and spatial features. Specifically, changes in the design of learning spaces are related to different learning performances of students (Ellis & Goodyear, 2016). The redesign of existing spaces is thus an important field of research for educational progress. However, the multitude of conceivable changes suggests to systematically relate the functional performance of learning spaces to their psychological and cognitive effects. Therefore, we aim at empirical guidelines for aesthetics, comfort, and amenity of learning spaces to estimate their functional performance.

In our study, we analyze how students observe and evaluate photographs of existing physical learning spaces in a controlled laboratory setting. First, the students evaluate key features of learning spaces and their friendliness and preference. Second, students select their seat in the room and rate the room again based on a photographs taken from the selected seat. By means of stationary eye tracking, we relate the spatiotemporal patterns of visual attention to the features and student evaluations to identify key learning space characteristics.

New insights in this regard can support decision-making at the design stage based on photographs to improve future learning spaces. In the long run, friendly and preferable learning spaces could support teachers and learners alike by triggering creativity, communication, engagement, and other environmental factors that support learning.

Ellis, R. A., & Goodyear, P. (2016). Models of learning space: integrating research on space, place and learning in higher education. *Review of Education, 4*(2), 149-191.

Poster 17

The Visual-spatial Attention's Characteristics of Chinese Developmental Dyslexia Children

Li Yutong, Sui Xue, Hu jinsheng
The School of Psychology, Liaoning Normal University, Dalian 116029, China

The oculomotor deficit was found in children with developmental dyslexia(DD) in alphabet writing system. Do Chinese DD have the same deficits? Is the visual-spatial attention affected by language specificity? The present study used the predictive saccades task, antisaccades task and visually guided saccades task.
The experimental materials included a black center cross (10 mm in diameter) and two solid circles (5 mm in diameter) of different colors, one red and one black. The solid circles as the target stimulus varied randomly in 5°, 10°and 15° from the center point.
Compared with the control group, in experiment 1, DD group had fewer predictive saccades, more regular and error-late saccades, and a longer overall saccades latency; in experiment 2, DD group have lower correct rate of antisaccades and longer overall saccades latency; in experiment 3, the overall latency between the two groups was relatively small, but the latency difference is obvious in each visual stimulus location, the larger the visual angle of the stimulus, the longer the latency.
In this study, three types of saccades tasks are used to compare the visual-spatial attention ability of Chinese children with developmental dyslexia from the perspective of implicit and explicit attention, and according to the results, our findings support the visual-spatial attention deficit on developmental dyslexia.
Keywords: Developmental dyslexia; Voluntary control of saccades; Reflexive saccades; Visuo-spatial attention deficit

Poster 18

Eye-tracking Applications in Aviation: A Selective Literature Review

Peißl, S, Wickens, C. D., and Baruah, R.
AUVA; University of Illinois and Colorado State University; Christ University Bangalore

Objective: The aim of this talk is to present a comprehensive review of eye-tracking measures and discuss different application areas of the method of eye tracking in the field of aviation.
Background: Psychophysiological measures such as eye tracking in pilots are useful for detecting fatigue or high workload conditions, for investigating motion sickness and hypoxia or for assessing display improvements and expertise.
Method: We review the uses of eye tracking on pilots and include eye-tracking studies published in aviation journals, with both a historical and contemporary view. We include 78 papers and assign the results to the following three categories: Human performance, aircraft design, health and physiological factors affecting performance. We then summarize the different uses of eye tracking in each category and highlight metrics which turned out to be useful in each area.
Results: On the basis of these analyses, we propose useful application areas for the measurement of eye tracking. Eye tracking has the potential to be effective in terms of preventing errors or injuries by detecting e.g. fatigue or performance decrements. Applied in an appropriate manner in simulated or real flight it can help to ensure optimal functioning of man-machine systems.
Conclusion: Further aviation psychology and aerospace medicine research will benefit from measurement of eye movements. An eye-tracking study on pilots applying the findings of this review is in preparation.

Poster 19

Attention Shifting during the Reading of Chinese Sentences

Weilin, Liu; Xingshan, Li; Albrecht Werner, Inhoff
Institute of Psychology, Chinese Academy of Sciences; Institute of Psychology, Chinese Academy of Sciences; Binghamton University

In Chinese reading, the perceptual span is one character to the left to three characters to the right of fixation, and it shows rightward asymmetry. In the current research, we proposed to investigate how was attention allocated during Chinese reading, and to further explore whether the asymmetry of perceptual span was closely related to the attention allocation. An eye-movement-contingent probe detection task was used to determine the allocation of visual attention during Chinese reading. On a subset of trials, a to-be-detected visual probe replaced visual text when the eyes crossed an invisible spatial boundary during reading. The probe was either near that fixated location or at a more distal location, and probes were shown to the right or left of fixation. Probe detection latencies were shorter for probes that were closer to fixation, and they were shorter when the probes were shown to the right rather than to the left of fixation. The right visual field advantage emerged, supporting the view that attention shifts account for asymmetries in the perceptual span during reading. However, the right visual field advantage also emerged when reading from right to left, although this advantage decreased over the course of the experiment. The results indicate that the direction of an attention shift during fluent reading is only partially determined by perceived word order. Instead, script-appropriate attention shifts appear to be executed by default and are difficult to change.
Key words: Reading; attention; eye movements; perceptual span

Poster 20

The pupil response to detection and storage of multiple targets in visual search

Christof Körner[1], Hannah Hiebel[1], Bettina Brückler[1], Nina Trausner[1], Iain D. Gilchrist[2] & Anja Ischebeck[1]
1 University of Graz/Austria, 2 University of Bristol/UK

Research has demonstrated that pupil size reflects processing effort in a visual search task which required the counting of targets (Porter, Troscianko and Gilchrist, 2007). Here, we were interested in whether and how memory for the spatial location of targets is reflected in pupil size. In a trial, participants searched a 30-item display for a varying number of 0 to 6 identical targets (search phase). The available search time varied between 5 and 8 s. Participants had to find and memorize the location of as many targets as possible during this period. After the end of the search phase, a 5 s interval followed during which participants did not move their eyes (retention phase). After this interval, participants performed a self-paced location task: with the mouse cursor, they marked the locations of memorized targets in the display (location phase). We measured eye movements and pupil size throughout the task. We found a clear effect of memory load for targets. Relative pupil size during the retention phase increased with the number of targets processed during the search phase. We also analysed pupil size during the search phase to track the moment-by-moment increase in memory load for targets, and we used pupil size in the retention phase to predict performance in the location phase. Our results demonstrate links between pupil size, target memory load and search performance.

Porter, G., Troscianko, T., & Gilchrist, I.D. (2007). Effort during visual search and counting: Insights from pupillometry. *Quarterly Journal of Experimental Psychology, 60,* 211 – 229.

Poster 21

Agents versus Avatars: Effects of manipulating the social relevance of gaze-cues on attention and eye-movements

Silver, Crystal; Scott-Brown, Kenneth
Abertay University

Research into gaze-cue effects has traditionally been conducted using computerised stimuli without human agency (i.e. social relevance). Recent studies have demonstrated that social appearance (i.e. anthropomorphic representation) alone is not adequate in eliciting truly social responses, social relevance is paramount. Therefore, we manipulated the perception of agency in a computerised target location task with integrated anthropomorphic gaze-cues in varying congruency. Participants believed gaze-cues were either computer-generated (agent) or dictated by a keyboard response of the experimenter (human-controlled; avatar). Behavioural (response times), physiological (heart rate, electrodermal activity) and eye-movement (number of fixations and initial fixation location) measures were recorded.

Human agency facilitated overall response times and exclusively elicited gaze-cue effects; response times significantly faster for congruently, compared to incongruently cued trials. Whilst participants were not required to look at the target for task completion, eye-movement recording revealed overt orienting in most trials, with significantly fewer fixations for human agency trials. There was no difference in the number of initial fixations towards the target or social (i.e. face, direction of gaze) locations between agency conditions. Heart rates were found to be lower in the human agency condition.

Our results support the automaticity of gaze-cue effects and the role of social relevance in eliciting social responses. Whilst it is widely established that gaze-cue effects do not necessarily engage overt orienting, results suggest that eye-movements may in fact play a role in the facilitation of response times associated with reflexive orienting; in human agency trials responses were faster and contained fewer fixations.

Poster 22

The role of cognitive mindset in physics problem solving.

Piotrowska-Półrolnik, Małgorzata ; Młyniec, Agnieszka; Krejtz, Izabela
SWPS University of Social Sciences and Humanities, Warsaw

The aim of the study is to analyse whether activation of cognitive mindset affects perception and efficacy of kinematics problem solving in physics (Kozhevnikov et al., 2007; Baron-Cohen et al., 2001).
According to Gollwitzer & Bayer (1999), cognitive processes activated by one task are automatically transferred to the next task. Büttner et al. (2014) demonstrated that activation of implementative mindset focuses attention on foreground object (goal-relevant information), whereas deliberative mindset focuses attention evenly on the whole scene (broadens visual attention). Kozhevnikov et al. (2007) demonstrated that holding multiple, uncoordinated representations of the same kinematics problem hinders integration of motion components and finding correct solution to the task in individuals of low-spatial abilities. High-spatial individuals are better able to focus their attention on task relevant information.
In the prescreening study individuals with the lowest (n = 45) and the highest (n = 45) spatial abilities were selected. In the main study each group was randomly allocated to three conditions: deliberative vs implementative vs control, followed by 31 kinematics tasks during which eye movements were recorded.
We hypothesize that activation of implementative mindset enhances, and activation of deliberative mindset hinders kinematics problem solving in both groups. The data collection is in progress. The preliminary analysis of scan paths suggests that high-spatial subjects in implementative condition direct their gaze to task relevant information compared to low-spatial subjects in deliberative condition. The results of this research might reveal implications for the development of specific mindset aids to enhance people’s abilities in solving kinematics problems.

Poster 23

Eye dominance modulates the spatial coupling between saccade and perception

Dorine Vergilino-Perez[1], Jérôme Tagu[1,2], Marla Karraz[1], & Karine Doré-Mazars[1]

1 *Laboratoire Vision Action Cognition, EA 7326, Institut de Psychologie, Université Paris Descartes, Boulogne-Billancourt, France*

2 *Icelandic Vision Laboratory, School of Health Sciences, University of Iceland, Reykjavík, Iceland*

The dominant eye is the one used to perform a monocular task. There is an advantage of the hemifield contralateral to the dominant eye for both motor (more accurate saccades) and perceptual (faster detections) performance. The current study examined the influence of eye dominance in a dual-task paradigm involving both a saccade and a discrimination task. The discrimination target (oblique line) appeared at one out of six possible locations on an imaginary circle and could coincide spatially or not with the saccade target. Distractors were presented at the five other locations. In two separate conditions (saccade vs. fixation), the participants' task was to indicate whether the discrimination target was oriented clockwise or counterclockwise Participants differed by their eye dominance (left/right) and eye dominance strength (strong/weak). The preliminary results in the saccade condition suggest that observers with strong eye dominance showed lower visual discrimination performance when the saccade target was in the hemifield contralateral to their dominant eye than in the ipsilateral one. This result was found whether the discrimination target coincided or not with the saccade target. Overall, this suggests that eye dominance modifies the coupling between saccade and perception in the hemifield contralateral to the dominant eye, inhibiting the locations of the contralateral hemifield. Theoretical implications will be discussed in light with neural correlates of eye dominance, oculomotor control and visual attention

Poster 24

Investigating EEG alpha and eye behavior in internally directed cognition

Simon Ceh, Sonja Annerer-Walcher, Christof Körner, Mathias Benedek
University of Graz

During the day, our eyes are open most of the time. However, oftentimes our attention is directed to internal processes. Such internal processes go in hand with specific eye behavior that can be described as visual disengagement. Here, we examined the relationship between visual disengagement and EEG alpha activity. This is an established neurophysiological indicator of internal attention, which is assumed to be involved in attenuating bottom-up information processing during states of internal attention. In our eye-tracking-EEG coregistration study, 42 participants worked on two tasks (anagrams and sentence generation), in which we manipulated the internal vs. external attention demands. The internally-directed cognition (IDC) condition was realized by masking the stimuli after 500ms in half of the trials, thus enforcing task performance without sensory input. In the other half of trials, stimuli were continuously available for participants to process (externally directed cognition; EDC). IDC produced greater pupil diameter, longer blink duration, fewer microsaccades, larger saccade amplitude and greater angle of eye vergence variance than EDC. These results replicated earlier eye-tracking findings and provide further evidence that eye behavior is indicative of internal attentional focus. Importantly, IDC involved relatively higher alpha activity (lower alpha desynchronization) especially in posterior and occipital areas, which is consistent with previous EEG research. In a final step, we are analyzing the temporal relationship between ocular parameters and EEG alpha activity to explore their mutual role in the modulation of visual perception. Together, these findings shed light on the neurophysiological mechanisms underlying sustained internally directed attention.

Poster 25

Individual differences in gaze patterns during risky financial decision-making

Milligan, Sara; Schotter, Elizabeth
University of South Florida

Risky decision making involves an interplay between characteristics of situations and characteristics of people. When given a choice between a low probability of gaining more money and a high probability of gaining less money, people tend to conservatively choose the higher probability/low payoff situation (Tversky et al., 1990). By studying deferential patterns of eye movements during the evaluation of information we can better understand how the decision-making process varies based on situational information and individuals' characteristics (i.e., riskiness, ambiguity aversion, and irrationality). To this end, we employ both eye tracking and behavioral tasks to investigate differences in how people allocate attention in risky situations.
In the current study, we manipulate the riskiness of a situation by increasing the range between potential positive and negative monetary payoffs and probabilities of each outcome. We use dwell time to determine the relative amount of cognitive resources spent evaluating different types of information. We find that people direct more attention (i.e., time) to probabilities than potential outcomes and more attention to negative versus positive outcomes. However, when we consider individual differences in risk preference, we see that people who are less risk averse increase attention to the positive outcome and reduce time on the negative outcome relative to those who are more risk averse. These findings suggest that personal traits influence not only the decisions that people make, but also the way in which they evaluate a situation.

Tversky, A., & Thaler, R. H. (1990). Anomalies: preference reversals. *Journal of Economic Perspectives, 4*(2), 201-211.

Poster 26

Self-focused attention in social anxiety: a naturalistic eye tracking investigation

Antolin, Jastine; Gregory, Nicola; Bolderston, Helen
Bournemouth University; Bournemouth University; Bournemouth University

Self-focused attention (SFA) is heightened in individuals with social anxiety (SA) which may cause inattention to others' positive external cues. As such, attentional training techniques have been developed to shift attention externally to reduce anxiety (Stot et al., 2013; Wells 1999). However, this has never been validated in a naturalistic setting. Therefore, we manipulated SFA whilst participants (N = 41) engaged in a Skype conversation. During the interaction, participants' own image was visible (self-focused condition) and then was later removed (external-focused condition), whilst eye-movements were recorded. Participants completed self-report measures of trait social anxiety and then self-focussed attention and state anxiety measures at three time points.

Whilst we expected that participants would report increased self-focus and anxiety and display increased self-gaze and less experimenter-gaze during the self-focussed condition, we predicted that this would be most pronounced in higher SA individuals. Conversely, we predicted that higher SA individuals would continue to report increased self-focus and anxiety and would show reduced experimenter-gaze during the external condition.

Our results suggest that self-focus increases for all participants when the self-image is visible but that those with high SA report increased state anxiety regardless of whether the self-camera is on, questioning the validity of attentional training aimed at increasing external-focus to reduce anxiety. Eye movement results will be discussed in terms of different viewing patterns emerging during self and external focused conditions in those higher and lower in SA. Implications for adapting attention-based therapeutic interventions will also be discussed.

References:

Stott, R., Wild, J., Grey, N., Liness, S., Warnock-Parkes, E., Commins, S. & Clark, D. M. (2013). Internet-delivered cognitive therapy for social anxiety disorder: a development pilot series. *Behavioural and Cognitive Psychotherapy, 41*(4), 383-397.

Wells, A., & Papageorgiou, C. (1998). Social phobia: Effects of external attention on anxiety, negative beliefs, and perspective taking. *Behavior therapy, 29*(3), 357-370.

Poster 27

Visual search in neurodevelopmental disorders

Canu, D., Ioannou, C., Fleischhaker, C. Biscaldi, M., Klein, C.
Department of Child and Adolescent Psychiatry, Psychotherapy and Psychosomatics, Medical Faculty, University of Freiburg

Introduction: Visual search has proven to be helpful in studying visuo-spatial information processing, but has been barely included in eye movement research on psychiatric conditions of childhood and adolescence.

Methods: Eye movements were recorded while patients with Early-Onset Schizophrenia (SZ), ADHD, Autism, controls (TD) searched a target in a 4x4 grid with 16 items, each showing a black outlined square with a gap on one of the sides. Participants were asked to locate the single target item as quickly as possible and to identify it by pressing a button on the keyboard.

Preliminary results: SZ patients showed normal mean latency but increased intra-subject variability (ISV) of the latency of the first saccade (initiation of search). Furthermore, the search process – defined as time between the onset of the first saccade and the onset of the last fixation on target – was slower and more variable in SZ than TD. Finally, patients were slower in identifying the target and slower and more variable than controls in deciding about the target.

Discussion and Conclusion: Results suggest that the slower search rate in schizophrenia cannot be fully attributed to a slowing in decision making, but rather to a less efficient serial search. Of particular interest is the finding of increased standard deviation in manual and all ocular-motor measures, suggesting that increased ISV highlights specific information processing deficits in patients with schizophrenia.

Poster 28

The development of learned hierarchical attention sets evident in gaze and choice

Watson, M.R.,[a] Voloh, B.,[b] Naghizadeh, M.,[a,c] Womelsdorf, T.[a,b]

a: *Centre for Vision Research, Department of Biology, York University, Toronto, Ontario*

b: *Department of Psychology, Vanderbilt University, Nashville, Tennessee*

c: *Canadian Centre for Behavioural Neuroscience, Department of Neuroscience, University of Lethbridge, Lethbridge, Alberta*

Learned hierarchically-structured representations enable task-relevant information at the level of contexts, feature dimensions and individual feature values to be efficiently extracted and acted upon. The effects of such hierarchies on gaze, however, are poorly understood. Here we present results from a dynamic 2-AFC object-selection task in which participants learned to choose multi-dimensional objects using context-dependent feature-based rules, e.g. "choose the red object in Context A". Rules were acquired suddenly, in one or two trials, but corresponding fixational biases only appeared several trials later, indicating a slower process gated by prior rule-learning.

Two related findings demonstrated learned attentional hierarchies. First, new rules were acquired faster if relevant dimensions had also been relevant in the previous block. Second, performance was better when contexts were the same across trials, as demonstrated in fixational biases to chosen objects, and also in choice accuracy and decision time. This was only apparent for correct trials after rule acquisition.

Two further results showed attention is preferentially allocated within single feature dimensions. First, immediately before acquiring a new rule, participants were more likely to chose objects with non-rewarded feature values on relevant feature dimensions. Second, after acquisition of one rule, performance in other contexts was better for objects rewarded in both contexts.

Taken together, we show uni-dimensional attentional allocations during the learning process that support sudden rule acquisition, with the later development of gaze biases reflecting learned hierarchical attentional sets.

Poster 29

Scene Layout Guides Visual Object Searching

Yong-Jie Li, Teng-Fei Zhan, Kai-Fu Yang*
MOE Key Lab for Neuroinformation, School of Life Science and Technology, University of Electronic Science and Technology of China, Chengdu 610054, China
**Corresponding author: yangkf@uestc.edu.cn*

Visual object searching is an important strategy of human visual system for fast scene perception. The guided search theory suggests that the contextual cues of scenes play a crucial role in guiding the effective object searching. In order to study how the scene layout contributes to the task of fast object searching, we executed psychophysical experiments using the eye-tracker to collect the human fixations from 500 indoor images with the tasks of searching for specific objects (including TV, paint and light) from a group of indoor scenes. The salient boundaries among regions (e.g., the boundaries between two walls of the indoor scenes) are extracted to roughly represent the scene layout. We analyzed the relations among the scene layouts, searching tasks and the fixation distributions. We found that most of the recorded fixations are concentrated around these boundaries, which suggests that the information of layout can be used to prime the presence or absence of the objects in the scenes and predict the fixation distribution. We speculate that these boundaries may help our visual system to quickly get a rough knowledge about the three-dimensional structure of the scenes, which could serve as a kind of prior to guide our visual system to explore the possible regions of the specific object we are searching for. This indicates that the scene layout can provide strong contextual cues for fast visual object searching. In addition, this study may also provide new ideas for designing efficient object detection methods in computer vision applications.

Acknowledgements
This work was supported by the Natural Science Foundations of China under Grant #61703075 and the Sichuan Province Science and Technology Support Project under Grant #2017SZDZX0019.

References

[1] **Bar, M., Kassam, K. S., Ghuman, A. S., Boshyan, J., et *al.*** *(2006). Top-down facilitation of visual recognition.* Proceedings of the National Academy of Sciences, 103, 449–454.
[2] **Eckstein, M. P.** (2017). Probabilistic computations for attention, eye movements, and search. *Annual Review of Vision Science, 3*, 319–342
[3] **Wolfe, J. M.** (2017). Visual attention: Size matters. *Current Biology, 27,* R1002–R1003.
[4] **Wolfe, J. M., Võ, M. L.-H., Evans, K. K., et al.** (2011). Visual search in scenes involves selective and nonselective pathways. *Trends in Cognitive Sciences, 15*, 77–84.
[5] **Wu, C.-C., Wick, F. A., and Pomplun, M.** (2014). Guidance of visual attention by semantic information in real-world scenes. *Frontiers in Psychology, 5*, 1–13.

Poster 30

Investigating the relation between gaze following and attentional disengagement

Y.J.R. de Kloe[1,2] , R.S. Hessels[1,2], C.M.M. Junge [1,2], I.T.C. Hooge[1], & C. Kemner[1,2,3]

1 *Experimental Psychology, Helmholtz Institute, Utrecht University, Utrecht, the Netherlands*

2 *Developmental Psychology, Utrecht University, Utrecht, the Netherlands*

3 *UMC Utrecht Brain Center , Utrecht, the Netherlands*

Background: In the YOUth study, potential early predictors of a child's social skill and behavioral control are investigated. The attentional shift that occurs due to a gaze cue is assumed to be a predictor for a child's social skill. This is operationalized in a gaze-cueing experiment as the difference in saccadic reaction time (SRT) between a congruent and incongruent gaze cue. Attentional (dis)engagement is assumed to be a predictor for the development of behavioural control. This is operationalized in a gap-overlap experiment as the difference in SRT between a gap and overlap condition. As these two operationalizations are seemingly similar, we investigate whether these measures are related.

Methods: Gap-overlap and gaze-cueing experiments were conducted in 104 children aged 8 to 16 years. The following measures were derived: in the gaze-cueing experiment the saccadic reaction times (SRT) in the (I) congruent condition, (II) in the incongruent condition, and (III) the gaze-cueing effect and in the gap-overlap the SRTs in the (I) gap condition, (II) the overlap condition and (III) the gap-overlap effect.

Results: Pearson correlations indicate that SRTs across experiments correlate positively (with correlation coefficients ranging from 0.29 – 0.80, all $p < .01$). The gap effect (overlap – gap SRT) and the congruency effect (incongruent – congruent SRT) were not significantly correlated ($p = 0.33$).

Conclusion: Attentional disengagement and attentional shifts due to a gaze cue are unrelated. This is a first prerequisite in the validation of these measures as predictors of diverging constructs: social skill and behavioral control.

Poster 31

Effects of disrupted attention in digital reading, an eye tracking study

Thierry Baccino & Véronique Drai-Zerbib
University P8; University Bourgogne Franche-Comté

During reading on digital displays, our attention is continuously disrupted by the irruption of numerous messages (email, ads,..) making our memorization and our comprehension more difficult. However, only few experiments have investigated the attentional availability during this digital reading especially using eye movements. Our study aims at analyzing the consequences of a series of interruptions occurring during a digital reading task using a whole text. 80 participants were required to read a long descriptive text (3 pages) in order to collect information on a specific topic. The text was displayed on 17 successive screens and contained relevant and irrelevant sentences related to the topic given at the start of the experiment (a kind of point of view). The disruption may occured either on relevant or irrelevant passages. The text reading was disrupted by pop-up message containing either emotional text or SMS conversations. We recorded the eye movements during reading before and after interruption as well as recall data (free recall and R/K responses) – Analyses are in progress.

Poster 32

Modeling Attention Shifts During Natural Scene Viewing

Lisa Schwetlick, Lars O. M. Rothkegel, Ralf Engbert
University of Potsdam

Human eye movements on natural scenes are guided by multiple classes of mechanisms. While image-dependent and viewer-dependent influences are the most frequently discussed, eye movement data also contains systematic tendencies which persist over viewers, images and experimental designs. These tendencies include the statistics of saccade and inter-saccade angles, in particular, the statistics of return saccades, and the dependencies of sequential eye movements with fixation durations. Existing dynamical cognitive models, such as the Scenewalk Model (Schütt et al, 2017), focussed on reproducing only some of these systematic tendencies (e.g., distributions of saccade amplitude). Here we propose a theory-driven extension of the basic model. In addition to the local saliency and inhibitory tagging streams in the original model, we implement pre- and postsaccadic attention shifts as well as facilitation of return via modified inhibitory tagging. These changes to the model are well founded in neuroscientific and cognitive theories of vision. Therefore, resulting model parameters have a clear biological interpretations and permit insights into the specific predictions of theoretical concepts of active vision. The model parameters were determined by a fully Bayesian framework using adaptive Markov Chain Monte Carlo (MCMC) simulations. Simulations based on the estimated model parameters indicate an improved agreement with the experimentally observed eye movement statistics.

Poster 33

Statistical learning of alternating priors in a random-walk eye-movement paradigm

André Krügel
University of Potsdam

Human sensorimotor control is the result of a Bayesian decision process, which is based on a combination of noisy sensory information and learned prior knowledge about the statistics of the environment (Körding & Wolpert, 2004, *Nature*). The range effect in eye-movement control, i.e., the systematic over- and underestimation of small and large target distances, is a signature of using prior knowledge for motor control (Engbert & Krügel, 2010, *Psychol. Sci*; Kapoula, 1985, Vis. Res.). However, most empirical studies employ very simple prior distributions while the statistical structure of the environment is much more complex. Here, I present data of a random-walk eye-movement experiment based on two alternating prior distributions. In this experiment, subjects had to make saccades towards targets that appeared either to the right or to the left of the current fixation position. Each trial consisted of a sequence of 80 consecutive target steps, with every second step drawn alternately from one of two partially overlapping Gaussian prior distributions. None of the subjects reported having noticed this manipulation. However, the eye movements show that the subjects implicitly learned the more complex structure of the task. Saccades over- or undershot equally distant targets depending on whether the targets originated from either the proximal or the distal prior distribution.

Poster 34

Boosting oculomotor plasticity through visuospatial attention.

Denis Pélisson[1,3], Judith Nicolas[1,2,3], Camille Métais[1,3], and Aurélie Bidet-Caulet[2,3]

1 *Integrative Multisensory Perception Action & Cognition Team (ImpAct), INSERM U1028, CNRS UMR5292, Lyon Neuroscience Research Center (CRNL), 69000 Lyon, France*

2 *Brain Dynamics and Cognition (Dycog Team), INSERM U1028, CNRS UMR5292, Lyon Neuroscience Research Center (CRNL), 69000 Lyon, France*

3 *University Claude Bernard Lyon 1, Université de Lyon, 69000 Lyon, France*

To what extent oculomotor and attention systems are linked remains strongly debated. Previous studies suggested that saccadic adaptation (SA), a model of oculomotor plasticity, and orienting of attention rely on overlapping networks in the parietal cortex (see references in[1]). Moreover, mounting evidence suggests a functional coupling between these two processes. Indeed, inducing SA in humans boosts attentional performances (e.g.[2]). Conversely, a study reported an effect of the attentional load on SA efficiency[3]. However, this study did not question the modality specificity of both saccades (either reactively or voluntarily triggered) and attention (endogenous and exogenous shifts).

We will present preliminary results of a study testing the impact of attentional load on SA efficiency in the different modalities. We designed a protocol in which the attentional load was increased by changing the proportion of trials necessitating a shift of covert attention[4] (three sessions per subject with 25%, 50%, and 75% of shift trials). The attentional task was coupled with an exposure to SA. The three sessions of increasing attentional load were coupled with SA under the following four modality configurations, separately in 4 experiments (18 subjects each): (1) endogenous / voluntary SA; (2) endogenous / reactive SA; (3) exogenous / voluntary SA; and (4) exogenous / reactive SA. SA efficiency was assessed by the relative saccadic gain change between the pre- and the post-exposure phases.

We predict modality specific effects (exogenous to reactive SA and endogenous to voluntary SA), with the bigger the attentional load, the stronger the SA.

References:

1. **Nicolas, J. et al.** Saccadic Adaptation Boosts Ongoing Gamma Activity in a Subsequent Visuoattentional Task. *Cerebral Cortex* (2018). doi:10.1093/cercor/bhy241
2. **Habchi, O. et al.** Deployment of spatial attention without moving the eyes is boosted by oculomotor adaptation. *Frontiers in Human Neuroscience 9*, (2015).
3. **Gerardin, P., Nicolas, J., Farnè, A. & Pélisson, D.** Increasing Attentional Load Boosts Saccadic AdaptationAttention Enhances Oculomotor Adaptation. *Investigative ophthalmology & visual science 56*, 6304–6312 (2015).
4. **Posner, M. I.** Orienting of attention. *Quarterly Journal of Experimental Psychology 32*, 3–25 (1980).

Poster 35

Which cues guide visual attention during a conversation?

Jessica Dawson
University of Essex

Previous research has established that we tend to look at the person speaking during a conversation, and that we shift our gaze in a tightly coordinated sequence along with the turn-taking of the speech. This series of experiments explores how manipulating aspects of the conversation affects the fixations to the speaker.
In the first experiment we tracked participants eyes while they watched clips of a natural group conversation. The clips were manipulated in terms of which visual and auditory cues were available, with 4 conditions: Control, Silent, Freeze-Frame (image stilled, and sound continued) and Blank (image removed, and the sound continued). Findings were that the general ocular motor movements and fixations to the speaker were similar in the Control and Silent condition. Furthermore, although reduced, the participants still appeared to follow the conversation with the removal of any additional visual cues in the Freeze-Frame condition. The second experiment expands upon this by manipulating the spatial location of the speakers, to explore why the sound promotes fixations to the image of the speaker when the image is stilled.
As an additional manipulation, further experiments within this series explore to what extent conversation following is affected by the ability to observe other people's eyes and how gaze following is affected in those with high ADHD-like traits. This research aims to represent real world scenarios with a combination of third party and live eye-tracking techniques.

Poster 36

Visual span, visual attention span and perceptual span on reading: differences and/or similarities.

Aline Frey, David Méary & Marie-Line Bosse
UPEC; LPNC; LPNC

It is well known that only a portion of available information is processed in a single fixation, which allowed characterizing three types of spans: the visual span – VS (O'Regan, 1990, 1991), the attention visual span - VAS (Bosse & Valdois, 2009) and the perceptual span – PS (McConkie & Rayner, 1975, 1976). Succinctly, the VS represents the number of adjacent letters arranged horizontally that can be recognised reliably without moving the eyes (Legge et al., 2007; Legge, Mansfield, & Chung, 2001); the VAS is defined as the number of distinct visual elements that can be processed simultaneously at a glance, in a multi-element configuration (i.e., in reading, the number of orthographic units; Bosse, Kandel, Prado & Valdois, 2014) ; and the PS is defined as the limited region from which useful information is acquired (Rayner, Slattery & Bélanger, 2010). While those three spans are clearly distinguished by their authors and differ from one another, either theoretically or methodologically, they are routinely used inconsistently in the literature.
In this experiment, we have measured those three spans, for the first time within the same participants and with the same paradigm characteristic (i.e., letter size, distance to the screen…), allowing a real comparison. Different general cognitive skills (i.e., short-term memory...) as well as eye-tracking characteristics (fixation time, reading speed…) have been also collected. Results allow to clarify the possible relationships and / or independence between those three spans, and to characterize their respective involvement in reading performance.

Poster 37

Early but not overwhelming: The effect of prior context on word segmentation of overlapping ambiguous strings during Chinese reading

Linjieqiong Huang[1, 2] **and Xingshan Li** [1 *]

1 *Key Laboratory of Behavioral Science, Institute of Psychology, Chinese Academy of Sciences, Beijing, China*

2 *Department of Psychology, University of Chinese Academy of Sciences, Beijing, China*

** Correspondence: Xingshan Li E-mail: lixs@psych.ac.cn*

Without inter-word spaces, Chinese readers need to use high-level information to segment words. The current study investigated how prior context influences word segmentation of overlapping ambiguous strings during Chinese reading. In two eye-tracking experiments, Chinese readers read sentences containing a 3-character overlapping ambiguous string (ABC) where both AB and BC were 2-character words. In the informative condition, prior contexts supported either the first word segmentation (AB-C) or the second word segmentation (A-BC), while didn't provide word-segmentation information in the neutral condition. The post-target contexts were also manipulated to favor either the first word segmentation (AB-C) or the second word segmentation (A-BC). Results showed that when prior context was informative, first-pass reading times were shorter in the AB-C condition than that in the A-BC condition, while no significant difference was found in the neutral condition. However, the effect in the informative condition was eliminated when word frequency of the words BC was higher than that of the words AB. In the informative condition, the difference between AB-C and A-BC conditions was also significant for regression-in probability. These results imply that during word segmentation process, prior context exerts its effect at an early stage but inefficiently. Readers still utilize word frequency information in situation of informative context. And the first word segmentation (AB-C) has processing advantages, suggesting a left segmentation priority.

Keywords word segmentation, prior context, Chinese reading

Reference

Brothers, T., & Traxler, M. J. (2016). Anticipating syntax during reading: Evidence from the boundary change paradigm. *Journal of Experimental Psychology: Learning, Memory, and Cognition, 42*(12), 1894-1906.

Ma, G., Li, X., & Rayner, K. (2014). Word segmentation of overlapping ambiguous strings during Chinese reading. *Journal of Experimental Psychology: Human Perception and Performance, 40*, 1046-1059.

Poster 38

Adult Age Differences in Word Frequency and Word Predictability Effects in Reading: Evidence from Eye Movements and Fixation-Related Potentials

Ascensión Pagán[1], Federica Degno[2], Richard Kirkden[1], Sarah White[1], Simon Liversedge[2] & Kevin Paterson[1]
University of Leicester[1], University of Central Lancashire[2]

Older readers are believed to (1) have greater difficulty identifying words and so produce larger word frequency effects, and (2) compensate for this difficulty by making greater use of context to predict words (Rayner et al., 2006). While numerous eye movement (EM) studies show larger word frequency effects for older compared to younger adults during sentence reading, the evidence that older readers make greater use of context is more limited (Choi et al., 2017). Moreover, ERP studies show larger word predictability effects for younger compared to older adults when sentences are presented one word at a time (Federmeier & Kutas, 2005). Given this contradictory pattern, we used co-registration of EMs and EEG to examine age differences in effects of word frequency (Experiment 1) and word predictability (Experiment 2) on reading times and fixation-related potentials (FRPs). Preliminary analyses show clear frequency and predictability effects in early EM measures (e.g., first-fixation duration) and trends towards larger frequency and predictability effects for older readers. We are currently completing data collection for older adults and will present EM and FRP data for 30 young and 30 older adult participants. The findings from both measures will be highly relevant to the debate concerning aging effects on word identification and use of contextual predictability during reading. Moreover, the FRP findings will be important in the context of previous research showing clear effects of word predictability (Kretzschmar et al., 2015), but not word frequency (Degno et al., 2018; Kretzschmar et al., 2015) in FRPs during sentence reading.

Poster 39

Processing of adapted texts by secondary schoolchildren with and without speech disorders: An eye-tracking study

Zubov, Vladislav; Petrova, Tatiana
Saint-Petersburg State University

Reading skills of the children with language disorders is one the main problems of correctional pedagogy. This study is one of the first eye-tracking experiments on the Russian language material, which explores how children read and comprehend texts with various types of adaptation, namely one and the same text in lexical adaptation (replacement of complex and rare words to more common ones), and grammatical adaptation (simplified syntactic structures and sentences). To examine this issue, 32 secondary school students with general speech underdevelopment and 32 secondary school students with normal speech development were involved in the experiment. The participants' eye movements were measured by Eyelink 1000+ during reading of 2 stimuli: a lexically- and a grammatically adapted text on two different stories. The complexity of the texts was equalized via http://readability.io/. We measured the total dwell time, the total fixation count, average saccade amplitude, average duration of the first pass and the number of regressions for each text. The text comprehension was controlled by after the text questions and scaling method, when the participants had to evaluate the level of text difficulty. The outcome has shown that there is no significant difference in processing and comprehension of lexically adapted and grammatically adapted texts in both groups of participants: each type of adaptation proved to be efficient for recalling the information. Nevertheless, we revealed the differences in the patterns of eye-movements for norm and different forms of general speech underdevelopment for both text types. Supported by RFBR grant No18-00-00640.

Poster 40

Examining the role of emoticons in sarcasm comprehension: Evidence from younger and older adults

Howman, Hannah; Filik, Ruth
University of Nottingham

Advancements in technology have seen an increase in the use of computer-mediated communication, with people using emoticons (or emojis) to enhance the meaning of their messages and to express their thoughts and emotions (Rezabek & Cochenour, 1998).We present an eye-tracking experiment examining the moment-to-moment cognitive processes underlying the comprehension of the wink emoticon; specifically, how it influences processing and interpretation of comments that are ambiguous between a literal and sarcastic interpretation. Younger (18-30 years) and older (65+ years) participants had their eye movements recorded while reading scenarios containing ambiguous comments that could be interpreted either literally or sarcastically (e.g., But you're so quick though), and ended with a wink emoticon or a full stop. Results showed that participants read earlier parts of the wink emoticon scenarios faster than those with full stops, but then spent more time reading the region of text containing the wink emoticon. Thus, readers moved more quickly to the end of the text when there was a device that may aid interpretation, but then spent more time processing the conflict between the superficially positive nature of the comment and the tone implied by the wink emoticon (i.e., that there is an additional hidden meaning behind the message). Interestingly, the wink emoticon increased the likelihood of a sarcastic interpretation in younger adults only, suggesting that perceiver-related factors play an important role in emoticon interpretation.

Rezabek, L., & Cochenour, J. (1998). Visual cues in computer-mediated communication: Supplementing text with emoticons. *Journal of Visual Literacy, 18*, 201-215.

Poster 41

Eye movement behaviour during reading: Effects of preceding text difficulty.

Faye O. Balcombe[1], Timothy J. Slattery[2] & Sarah J. White[1]
1 Department of Neuroscience, Psychology and Behaviour, University of Leicester, UK
2 Department of Psychology, Bournemouth University, UK

Experiments are presented that examine how the difficulty of preceding sentences modulates eye movement behaviour during reading of subsequent unrelated sentences. The study builds on previous work examining effects of preceding difficulty across sentences within connected text (Huestegge, & Bocianski, 2010; Slattery, Pollatsek, & Rayner, 2007). In the present experiments, the difficulty of preceding text was modulated by manipulating the difficulty of sentences within preceding trials (unrelated filler sentences). Difficult filler sentences included an object relative clause, nested relative clause, or a syntactic ambiguity. Easy filler sentences included control versions of these filler sentences. Experimental items included a critical word and the characteristics of the critical word were manipulated. Critical words were either high or low in predictability given the sentence context. Crucially, preceding text difficulty (the difficulty of preceding unrelated filler sentences) modulated first-pass eye movement behaviour for the experimental items, especially the likelihood of refixating words. Preceding text difficulty may also modulate the time course of effects of word predictability (for related findings in self-paced reading see Brothers, Swaab & Traxler, 2017). Importantly, the results of these new experiments indicate that the difficulty of preceding text can modulate eye movement behaviour during reading of subsequent unrelated sentences. Implications for models of eye movement behaviour during reading will be discussed, for example, the possibility that the difficulty of preceding text might modulate a global parameter for eye movement control. Methodological implications for experiments on eye movements during sentence reading will also be outlined.

References

Brothers, T., Swaab, T.Y., & Traxler, M.J. (2017). Goals and strategies influence lexical prediction during sentence comprehension. *Journal of Memory and Language, 93*, 203-216.
Huestegge, L., & Bocianski, D. (2010). Effects of syntactic context on eye movements during reading. *Advances in Cognitive Psychology, 6*, 79-87.
Slattery, T.J., Pollatsek, A., & Rayner, K. (2007). The effect of the frequencies of three consecutive content words on eye movements during reading. *Memory & Cognition, 35*, 1283-1292.

Poster 42

Preserved syntactic processing in older adulthood: Evidence from eye movements

Tami Kalsi[1], Kevin Paterson[2] and Ruth Filik[1]
1 University of Nottingham, 2 University of Leicester
tami.kalsi@nottingham.ac.uk

Previous research suggests that older adult readers (aged 65+ years) are more likely than young adult readers (aged 18-30 years) to engage in 'good-enough' processing in they assign a subjectively plausible analysis to a sentence instead of conducting a full syntactic analysis (Christianson et al., 2006). To investigate this issue further, we conducted an eye movement study to investigate age differences in the processing of temporarily ambiguous sentences like "While the gentleman was eating the burgers were still being reheated in the microwave.", and unambiguous versions that were disambiguated using a comma (e.g., after "eating"). After reading each sentence, participants were presented with a "yes/no" comprehension question that assessed whether the ambiguity was resolved correctly (e.g., "Was the gentleman eating the burgers?"), where incorrectly endorsing the question might indicate participants had assigned a plausible interpretation to the ambiguity. Both young and older adults had longer reading times at the critical phrase (e.g., "were still") of ambiguous compared to unambiguous sentences, indicating that the ambiguity caused similar difficulty for the two age groups. Both age groups were also equally likely to incorrectly endorse comprehension questions. The indication is that the young and older readers experienced similar syntactic processing difficulty and were equally likely to engage in 'good-enough' processing during reading. We discuss these findings in terms of evidence for preservation of syntactic processing in older adulthood.

Reference

Christianson, K., Williams, C.C., Zacks, R.T., & Ferreira, F. (2006). Younger and older adults' "good-enough" interpretations of garden-path sentences. *Discourse Processes, 42*, 205–238.

Poster 43

Undersweep-Fixations and Lexical Processing during Reading in Adults and Children

Parker, A. J., Kirkby, J. A., & Slattery, T. J.
Bournemouth University

Return-sweeps take a reader's fixation from the end of one line to the start of the next. Return-sweeps frequently undershoot their target and are followed by a corrective saccade towards the left margin. The pauses prior to correctives saccades are typically considered to be uninvolved in linguistic processing. However, recent findings indicate that these undersweep-fixations influence skilled adult reader's subsequent reading pass across the line and provide preview of line-initial words. This research examined these effects in children. A children's reading corpus analysis revealed that words receiving an undersweep-fixation were skipped more and received shorter gaze durations. A subsequent eye movement experiment directly compared the eye movements of children and adults. While undersweep-fixations were not terminated based on lexical-frequency of the fixated word, both groups acquired information that informed their subsequent pass. We argue that this information is acquired preattentively. Additionally, fixation times on line-initial words were shorter following undersweep-fixations than if they had been directly fixated, indexing parafoveal preview during undersweep-fixations. However, the fixation after the undersweep-fixation showed no signs of spillover of lexical-frequency. We interpret this as evidence that attention during an undersweep-fixation is on a line-initial word rather than the fixated word.

Poster 44

Return-sweep saccades in oral and silent reading

Victoria I. Adedeji, Adam J. Parker, Julie A. Kirkby, & Timothy J. Slattery
Bournemouth University, UK

Return-sweep saccades take reading fixations from the end of one line of text to the beginning of the next. Less is known about these large saccadic movements than is known about reading saccades within a line of text. During silent reading, the fixation prior to a return-sweep is shorter in duration than intra-line fixations (Parker, Slattery, & Kirkby, 2019). However, with reading aloud the eyes often lead the voice (Laubrock & Kliegl, 2015) and this coupling may impact return-sweep planning and execution. For instance, previous studies have reported that fixation durations and refixation rates increase while parafoveal preview benefit significantly reduces during oral reading (Ashby, Yang, Evan, & Rayner 2012). In the current study, participants read paragraphs, either aloud or silently, while their eye movements were recorded. Return-sweeps were launched from closer to the end of a line and landed closer to the beginning of the subsequent line when reading aloud compared to reading silently. Compared to intra-line fixations, line final fixations were shorter in duration while accurate line initial fixations were longer in duration. Fixations durations were also significantly longer when reading aloud compared to reading silently. Moreover, the time cost associated with reading aloud was significantly greater for line-final and accurate line-initial fixations in comparison to intra-line fixations. These findings suggest that when reading aloud, return-sweeps offer a natural pause in the uptake of new visual information which readers can use to prevent the eyes from getting too far ahead of the voice.

Ashby, J., Yang, J., Evans, K. H. C., & Rayner, K. (2012). Eye movements and the perceptual span in silent and oral reading. *Attention, Perception, and Psychophysics, 74*, 634–640. https://doi.org/10.3758/s13414-012-0277-0
Laubrock, J., & Kliegl, R. (2015). The eye-voice span during reading aloud. *Frontiers in Psychology, 6*, 1–19. https://doi.org/10.3389/fpsyg.2015.01432
Parker, A. J., Slattery, T. J., & Kirkby, J. A. (2019). Return-sweep saccades during reading in adults and children. *Vision Research, 155*, 35–43. https://doi.org/10.1016/j.visres.2018.12.007

Poster 45

Quantifier comprehension and the hypothesis of visual saliency: eyetracking data in a picture-identification task

Renê Forster[1], Erica dos Santos Rodrigues[2] e Letícia Maria Sicuro Corrêa[2]

1 Rio de Janeiro State University

2 Pontifical Catholic University of Rio de Janeiro

The comprehension of quantifiers by children has been traditionally investigated in the sentence-picture-matching paradigm (Crain et al., 1996; Roeper et al., 2004). A poor performance has been obtained, which may result from the visual properties of the stimuli (Minai et al., 2012). Given a sentence such as 'Is every cat kissing a turtle?', there is a tendency for children to give a "no" (wrong) answer against a picture containing three cats, each of them kissing a turtle, plus a single extra turtle, whereas their performance is better when multiple extra objects are presented (Sugisaki and Isobe, 2001). The visual saliency of the uniquely unpaired extra object has been claimed to capture children's initial attention to the point of preventing them from succeeding in the task (Minai et al., 2012). It should be noticed, however, that adult controls do not perform at ceiling (Minai et al., 2012). This study tested the visual saliency hypothesis with adults in a picture-identification task. Two pictures were simultaneously presented, in which the extra object was manipulated as a within-subject factor: single or multiple. A 120Hz TOBII eye-tracker was used. Fixation time and the number of correct responses were the dependent variables. Contrary to the prediction of the single-object salience hypothesis, longer fixations were obtained in the multiple-object condition, suggesting greater saliency. High error rates were still obtained for one third of the subjects even though no suggestion of single-object saliency was observed. An interaction between visual processing and quantifier interpretation is discussed.

Crain, S., Thornton, R., Boster, C., Conway, L., Lillo-Martin, D., & Woodams, E. (1996). Quantification without qualification. *Language Acquisition, 5*(2), 83-153.

Minai, U., Jincho, N., Yamane, N., & Mazuka, R. (2012). What hinders child semantic computation: children's universal quantification and the development of cognitive control. *Journal of Child Language, 39*(5), 919-956.

Roeper, T., Strauss, U., & Pearson, B. Z. (2004). The acquisition path of quantifiers: Two kinds of spreading. *Current Issues in Language Acquisition*, UMOP, 34.

Sugisaki, K.; Isobe, M. (2001). Quantification Without Qualification Without Plausible Dissent. In: *Proceedings of The Semantics of Under-represented Languages of the Americas*, eds. Ji-Yung Kim and Adam Werle, p. 97-100.

Poster 46

Influence of reading skill on word length effect in Fixation related potentials of school aged children.

Otto Loberg, Jarkko Hautala, Jarmo A. Hämäläinen & Paavo H.T. Leppänen
University of Jyväskylä; Niilo Mäki Institute; University of Jyväskylä; University of Jyväskylä

Word length has been shown to have greater influence on eye-movements (EM) if the individual has poor reading skill. Whether this difference co-occurs with electrophysiological brain activity effects is unknown. We examined influence of word length on fixation related potentials (FRP) and EMs of school aged (12 to 13,5 years) Finnish children. For this end, we used unexamined part of our dataset used in previous study. In this study, participants read sentences and provided sensibility judgements while their EMs and EEG were co-registered. We had two groups of participants: Slow readers (N=27) and Typical readers (N=65). FRPs were extracted with linear deconvolution with word length, fixation type (first fixation vs additional fixation) and previous saccade amplitude as predictors of the brain activity. FRPs were analyzed with nonparametric cluster based permutation tests and EM variables with LMMs. Pattern of stronger word length effect in eye-movements for slow reading was replicated, but effect of word length was not different between Slow and Typical readers in the FRPs. We found that for EMs word length had influence on first fixations, not on additional fixations. In FRPs word length had influence on brain activity only during additional fixations. We also found that the intercept FRP's, that contains the activity common to all fixations used in the estimation, were different between the two groups. Results suggest that stronger influence of word length on EMs of Slow readers is not due stronger direct modulation, but rather reflects accumulation of inefficient processing over multiple fixations.

Poster 47

LexiaD, the first Dyslexia-specific Cyrillic font, decreases mean fixation duration during sentence reading in Russian

Svetlana, Alexeeva; Golovatyuk Maria
St. Petersburg State University; Nimax

Dyslexia is a problem with the acquisition of reading decoding and/or spelling. There is evidence that the presentation of the text has a significant effect on a text's accessibility for people with dyslexia. This encourages designers to invent Dyslexic-specific fonts like Dyslexie or OpenDyslexic. However, most of them are Latin-based.

In this study, we designed and tested the first Dyslexic-specific Cyrillic font (LexiaD) that was based on reduction of inter-letter similarity of the Russian alphabet that was objectively measured by a pretest eye-tracking experiment (Alexeeva & Konina, 2016).

The *LexiaD* font. The font is proportional and sans serif. We enlarged the amount of the white space inside letters, inter-word and inter-line spacing, and leading. Its letters are made as different as possible from the visual similar counterparts by (a) emphasizing the individual characteristics of the letters; (b) extending ascender and descender parts; (c) thickening lines near the bottom of the letters.

Readability Testing. In the study, third or fourth grade children with (n=7) and without (n=23) dyslexia read the Children version of the Russian Sentence Corpus (Korneev et al., 2018) while their eye movements were recorded. The corpus comprised 30 sentences which were randomly divided for each participant in two halves. The first half was rendered in LexiaD, the other part was read in PT Sans, a sans serif Cyrillic font that is claimed to be one of best by font experts. Both participant groups showed the significant decrease in mean fixation duration while reading in LexiaD. Therefore, LexiaD proved faster to read and could be recommended to use by dyslexics.

References

Alexeeva, S. V., & Konina, A. (2016). *Crowded and uncrowded perception of Cyrillic letters in parafoveal vision: Confusion matrices based on error rates.* Perception, 45(2), 224–225.

Korneev A.A., Matveeva E. Yu., Akhutina T.V. (2018). *What we can learn about the development of reading process basing on eye-tracking movements?* Human Physiology, 44(2), 75–83.

Poster 48

Ambiguity Resolution in Natural Reading

Anastasiia Kaprielova, Anna Laurinavichyute
Center for Language and Brain, National Research University Higher School of Economics
Moscow, Russia
akaprielova@hse.ru

How can people effectively understand ambiguous messages? A possible answer is that understanding is not always effective, and ambiguity may not be resolved at all. Swets, B., Desmet, T., Clifton, C., & Ferreira, F. (2008) showed that syntactic ambiguities are read faster when sentence meaning was underspecified. We aim to find out whether similar meaning underspecification is possible with lexical ambiguities.

If no ambiguous word`s meaning is favored by the sentence context, any meaning that comes to reader`s mind will fit the sentence, and ambiguous words in such contexts might be read faster than in more restrictive ones. In a restrictive context, the reader will need time to activate the correct meaning of the ambiguous word and suppress the competing ones.

We tested 33 nouns with three meanings each (direct, metaphorical, and metonymic). For each noun, an experimental set with four conditions (contextual sentence favoring direct, metaphoric, metonymic or none of the meanings) was created. The contextual sentence was followed by the target sentence with the ambiguous word, which was constant across conditions.

80 people read experimental sentences while their eye-movements were tracked. We expected to see a speedup on the ambiguous word in the underspecified condition compared to the other conditions. But we found that it was read longer in the metaphoric sense condition instead: SFD – 95% CrI = [1, 25]ms, TT – 95% CrI = [1, 25]ms. These findings demonstrate that lexical ambiguities are processed in a different way than syntactical ones.

Poster 49

Eye movements indicate comprehension monitoring in silent and oral reading

Laura Schwalm, Chris Vorstius, Ehsan Ansari & Ralph Radach
University of Wuppertal

There has been little prior research on differences of eye movement control in silent vs. oral reading. Our study is focused on the issue of comprehension monitoring, a continuous (largely automatic) process of checking whether new linguistic input is coherent with prior information. We build on previous work using a two-sentence paradigm in which the *plausibility* (or typicality) of an agent, object or instrument in the second sentence is varied in relation to an action or event expressed by a verb in the first sentence (Connor, Radach, Vorstius, Morrison, McLean & Day, 2015).
Eye movements were recorded while fluent participants read German sentences in silent and oral mode. Replicating our earlier results with English materials, we found little effect of target word implausibility on initial fixation duration, but a substantial increase in gaze duration and total reading time, mainly due to an inflation of re-reading time. Critically, neither viewing times nor any spatial eye movement parameters (fixation probability, refixation rate) suggest any interaction between reading mode and plausibility effects. Saccade landing site distributions and the metrics of regressions back to the first sentence were also affected by typicality but not reading mode.
Why is there no effect? It might be argued that processing time advantages of slower reading aloud is offset by the concurrent mental workload required for working memory, language production and articulation. We favor an explanation in terms of a *comprehension monitoring* mechanism adjusting reading speed in both reading modes to meet a common criterion of optimal comprehension.

Poster 50

Effects of contextual constraints on word processing in reading

Veronika Prokopenya & Alisa Zhukova
Saint Petersburg State University

We investigate how context affects word processing during reading. It was shown that words highly predictable from the previous context are read faster than unpredictable words. However, the question of whether readers form predictions regarding an upcoming word or whether appropriate context information accelerates the integration of this word in the discourse remains unresolved. Two types of predictability were studied: (1) global, arising from the topic of the text; (2) local, conditioned by the words directly preceding the target. In Experiment 1 Russian speaking participants had to read entitled texts containing three types of target words: (1) globally predictable, (2) locally predictable, (3) unpredictable, but plausible in the given context. The results showed that local predictability has an advantage at the early stage of processing that is reflected in early eye-tracking measures (skip rate, 1st fixation duration, gaze-duration), while global predictability tends to be expressed in late measures (spillover, i.e. duration of the 1st fixation on the word following the target). In Experiment 2 we used the boundary paradigm and manipulated the semantic and orthographic relatedness of the parafoveal preview and target word. The effects of these manipulations were similar for (1) and (3) but differ for (2), supporting the idea that local predictability modulates the early stages of processing. Our results showed that contextual predictability influences word processing, however, mechanisms of local and global predictability differ: the former affects the lexical access, while the latter, tentatively, facilitates the integration of the target word in context. *Supported by RFBR #18-00-00640*.

Poster 51

Japanese Children's Effective Visual Field in Horizontal and Vertical Reading

Jincho, Nobuyuki; Mazuka, Reiko
Miidas Co, Ltd.; RIKEN Center For Brain Science

This study compared size of perceptual span and word identification span when 5th graders (N=19) read horizontal and vertical text in Japanese. In a moving window paradigm experiment, participants read two novel stories with one horizontal and one vertical reading. A gaze contingent moving window controlled the number of visible characters preceding and following the current fixation (1, 2, 4, or 6 characters for preceding region and 1, 2, 4, 6, 8, or 10 characters for following region). Results of the generalized linear models on forward fixation duration (FFD) and forward saccade length (FSL) revealed that FFD increased when the above and below 2 characters were perturbed in vertical text and the left 1 character and right 4 characters were perturbed in horizontal text, compared to when they were not. FSL was shorter when the above 2 characters and below 4 characters were perturbed in vertical text and the left and right 2 characters were perturbed in horizontal text than when they were not. These results suggest that the size of effective visual field is similar for vertical and horizontal text and the positions of them are more forward for horizontal text than for vertical text. This finding is consistent with the Japanese adults' effective visual field during reading (Jincho, 2017).
Jincho, N. (2017). Effective visual field of horizontal and vertical reading in Japanese. Poster presented at the 19th European Conference on Eye Movements, Wuppertal, Germany.

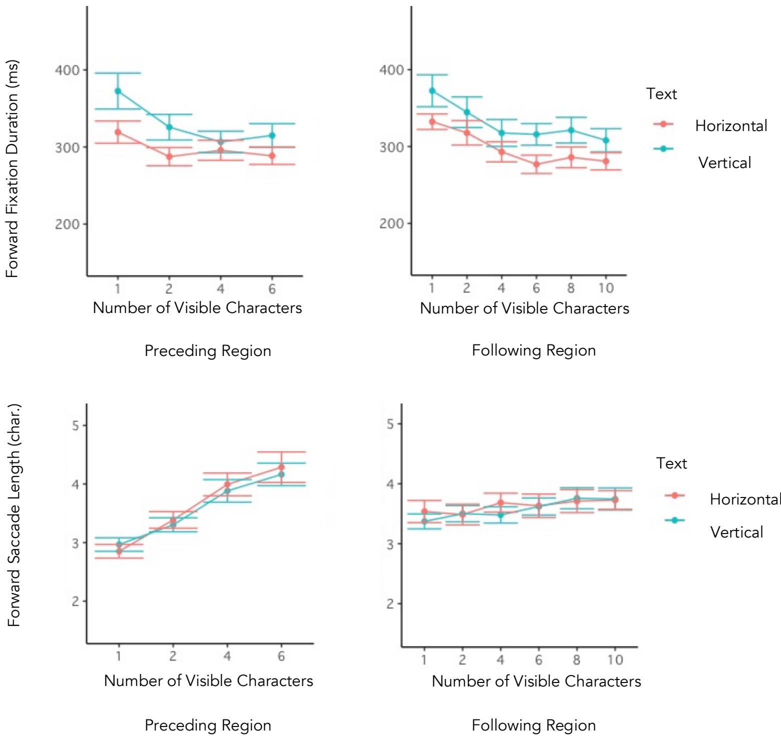

Figure. Forward Fixation Durations (top) and Forward Saccade Length (bottom) as function of text type and number of visible characters

Poster 52

Effects of Stimulus Quality and Word Frequency during Sentence Reading

Federica Degno[1], Fruzsina Soltész[2], Piril Hepsomali[2], Nick Donnelly[3], & Simon P. Liversedge[1]

1 University of Central Lancashire, 2 University of Southampton, 3 Liverpool Hope University

Consistent evidence both in the eye movement (EM) and event-related potential literatures has demonstrated that the quality of the stimulus in the fovea is essential for efficient reading. Much more limited is the evidence for an effect of quality while the stimulus is in the parafovea. In addition, while EM studies report robust foveal effects of word frequency, these effects are not observed when fixation-related potentials (FRPs) are recorded during sentence reading. In the present experiment, we simultaneously recorded participants' EMs and FRPs while they read sentences for comprehension. A target word was embedded in each sentence and manipulated for parafoveal stimulus quality (degraded vs. non-degraded preview) and foveal word frequency (high vs. low frequency target word). Initial analyses on first-pass reading times and amplitudes of FRP components showed effects of parafoveal and foveal stimulus quality in both the EM and FRP data, with foveal effects of word frequency in the EM data only. Deconvolution analyses of these data are currently underway and these analyses will disentangle FRP effects uniquely associated with specific, separate fixations on the pre-target and target words.

Poster 53

The influence of foveal lexical processing load on parafoveal preview extent in Chinese reading

Manman Zhang[1]**, Simon P. Liversedge**[2]**, Zhichao Zhang**[1]**, Xuejun Bai**[1]**, Chuanli Zang**[1, 2]

1 *Academy of Psychology and Behavior, Tianjin Normal University, Tianjin, China*

2 *School of Psychology, University of Central Lancashire, Preston, UK*

Recent research investigating the foveal load effect on parafoveal preview has failed to make a distinction between the depth and the spatial extent of parafoveal processing. The present study explored how foveal load influences preview extent in Chinese reading. Participants (N = 130) read sentences with embedded two-character pretarget words (high/low frequency - low/high load) and three-character target words while their eye movements were recorded. The boundary paradigm was used to provide previews of zero, one, two or three characters of target words (the remainder of the sentence was masked by pseudocharacters), or the full sentence. Pretarget word analyses showed robust first pass frequency effects, indicating an effective foveal load manipulation. First-pass reading times on the target showed robust preview effects (longest first pass times for zero, then one, two, three and full previews respectively). The smaller the preview window, the more disruption to processing occurred. Critically, interactive effects were apparent. Reading times were similar across load conditions for full sentence, three- and two-character previews. However, for one character and zero previews, reading times were shorter under high- than low-load conditions, indicating that target processing disruption due to restricted preview increased when foveal load was low relative to high. We conclude that in Chinese reading, foveal load modulates the extent of parafoveal processing, reducing the sensitivity to disruptive parafoveal information. However, such effects appear to be limited to a single parafoveal character and reflect a reduction in preview cost rather than an increase in preview benefit.

Keywords: Chinese reading, foveal load, parafoveal preview extent, preview cost, preview benefit.

SCENE PERCEPTION

Poster 54

Eye movements and additional psychophysiological markers of perceiving text and visual content

V. Anisimov[1,2], A. Kovaleva[3], K. Shedenko[2], N. Galkina[1], A. Latanov[1,2]
(1 - Joint-Stock Company 'Neurotrend', Moscow, Russia; 2 - Lomonosov State University, Moscow, Russia; 3 – Anokhin Research Institute of Normal Physiology, Moscow, Russia)

The study is dedicated to the analysis of psychophysiological correlates of interest in descriptions of travel tours (primarily eye movements). Participants (males, n=21, 18-26 years old) read different texts about some places of interest in Russia and then answered the questions about their interest and attractiveness of viewed examples. EEG and autonomic indices were registered synchronously (according to the method developed by authors) with eye movement data registration. The method allowed directly separate data for periods of text reading, viewing questions and choosing answers. Additionally, psychological status and functional asymmetry were assessed. Firstly, psychophysiological data were analyzed separately: eye movements, EEG, and autonomic measurements. Then, the complex predictive model was built.
It was revealed that some eye-tracker characteristics (fixation durations and saccade amplitudes) significantly differ for two situations: interest and no interest to travel tours. Several autonomic indices (heart rate, finger temperature, breathing parameters) also correlated with the issue. After all, a large data array (all parameters together) was used for mathematical analysis based on the predictive statistical model. Machine learning analysis was applied to the data. Results confirm the crucial role of eye movement parameters in interest prediction and also revealed the role of interhemispheric asymmetry of alpha-band power.The method shows the possibility of interest assessment based on several crucial physiological indices.
The study enriches view about efficient methods in research of human behavior in the situation of perceiving information during reading text and viewing illustrations (e.g., in the advertisement).

Poster 55

Joint and Self-Focused Attention in Computer-Supported Collaboration - the Role of Gaze Visualisation

Katarzyna Wisiecka[1*], Sven Mayer[2], Robin Schweigert[2], Izabela Krejtz[1], Radosław Nielek[3], Andreas Bulling[2], Krzysztof Krejtz[1]

1. *Psychology Department, SWPS University of Social Science and Humanities, Warsaw; *kwisiecka@st.swps.edu.pl*
2. *Institute for Visualisation and Interactive Systems, University of Stuttgart, Germany*
3. *Information Technology Department, Polish-Japanese Academy of Information Technology, Warsaw, Poland*

Following another person's gaze to a new focus of visual attention creates a situation of joint attention (Carpenter et al., 1998). Joint attention is possible to achieve if the conversation partners have the ability to inhibit their self-perspective (Samson et al., 2005). To fully engage in the process of collaboration, gaze communication enables a transition from a self-focused perspective to focusing on another person's gaze direction. Gaze visualisations provide solutions to attach directing attention to crucial information during computer-supported collaboration (Zhang et al., 2017). However, it remains unclear if gaze visualisation could reduce difficulties in reaching joint attention among self-focused people. In our study, participants pre-screened by Self-Consciousness Scale (SCS-R) solve two tasks requiring mutual problem solving and joint visual search in two conditions and in two experimental settings. We use a within-subjects experimental design 2x2 = Setting x GazeVis. The setting is divided into a co-located and a remote computer collaboration, while the collaboration is enhanced with or without the partner's gaze visualization. While the data collection is on-going, we hypothesise that (1) more self-focused attention is correlated with the level of achieved joint attention, (2) gaze visualisation enhances joint attention, as well as efficiency and (3) gaze visualisation, is more effective in remote setting compared to the co-located setting. Regardless of the outcome, our results will contribute to a greater understanding of the role of gaze communication in computer-supported collaboration within different settings and kinds of tasks.

Carpenter, M., Nagell, K., Tomasello, M., Butterworth, G., & Moore, C. (1998). Social cognition, joint attention, and communicative competence from 9 to 15 months of age. *Monographs of the society for research in child development, i-174*.

Samson, D., Apperly, I. A., Kathirgamanathan, U., & Humphreys, G. W. (2005). Seeing it my way: a case of a selective deficit in inhibiting self-perspective. *Brain, 128*(5), 1102-1111.

Zhang, Y., Pfeuffer, K., Chong, M. K., Alexander, J., Bulling, A., & Gellersen, H. (2017). Look together: using gaze for assisting co-located collaborative search. *Personal and Ubiquitous Computing, 21*(1), 173-186.

Poster 56

Inhibition of return across multiple tasks and scene flicker

Dovbnyuk Ksenia, Murzyakova Nadezhda, Merzon Liya and W. Joseph MacInnes
National Research University Higher School of Economics; National Research University Higher School of Economics; National Research University Higher School of Economics ; National Research University Higher School of Economics

Inhibition of return (IOR) is one of the processes believed to help guide visual tasks and facilitate visual search (Klein & MacInnes, 1999). Nevertheless, there are still debates on the mechanisms underlying IOR, its evolutionary meaning and the conditions in which IOR arises. For instance, for visual tasks other than search, there is evidence that facilitation of return and saccadic momentum could be alternative processes that guide the behavior (Dodd, Van der Stigchel, & Hollingworth, 2009). Our research investigates the presence of IOR in multiple tasks. The study consists of two sets of experiments. The first one compares visual search and memorization tasks to partially replicate Dodd et al. study. For the second one we test visual search, foraging (Jóhannesson, Thornton, Smith, Chetverikov, & Kristjánsson, 2016) and change blindness (Gusev, Mikhailova, & Utochkin, 2014) tasks. For both experiments we apply gaze-contingent paradigm where the target is presented at 1-back or 2-back fixation location or at a novel location. Instead of natural scenes a matrix of stimuli from Chetverikov and colleagues' study (Chetverikov, Kuvaldina, MacInnes, Jóhannesson, & Kristjánsson, 2018) will be applied. We expect that IOR will be presented in visual search and foraging tasks, but facilitation of return in other tasks in consistence with Dodd and colleagues' data. Additionally, search, foraging and change blindness tasks will be implemented with a flicker paradigm that allows us to test IOR with scene ongoing and scene removal.

References:

Chetverikov, A., Kuvaldina, M., MacInnes, W. J., Jóhannesson, Ó. I., & Kristjánsson, Á. (2018). Implicit processing during change blindness revealed with mouse-contingent and gaze-contingent displays. *Attention, Perception, & Psychophysics*, 1–16.

Dodd, M. D., Van der Stigchel, S., & Hollingworth, A. (2009). Novelty is not always the best policy: Inhibition of return and facilitation of return as a function of visual task. *Psychological Science, 20*(3), 333–339.

Gusev, A. N., Mikhailova, O. A., & Utochkin, I. S. (2014). Stimulus determinants of the phenomenon of change blindness. *Psychology in Russia, 7*(1), 122.

Jóhannesson, Ó. I., Thornton, I. M., Smith, I. J., Chetverikov, A., & Kristjánsson, Á. (2016). Visual Foraging With Fingers and Eye Gaze. *I-Perception, 7*(2), 2041669516637279. https://doi.org/10.1177/2041669516637279

Klein, R. M., & MacInnes, W. J. (1999). Inhibition of return is a foraging facilitator in visual search. *Psychological Science, 10*(4), 346–352.

Poster 57

Pupillary responses to differences in luminance, color and set size

Julia Oster, Jeff Huang, Ralph Radach & Douglas P. Munoz

University of Wuppertal; Centre for Neuroscience Studies, Queen's University; University of Wuppertal; Centre for Neuroscience Studies, Queen's University

The sympathetic and parasympathetic pathways of the pupil orienting circuit control the human pupil. The superior colliculus (SC) is a critical sensorimotor hub in the pupil control circuit for delivering orienting and cognitive signals to the pupil (Wang & Munoz, 2015). We know that manipulations of luminance, color and complexity of visual stimuli alter SC visual activity and so we hypothesized we should find correlates of these manipulations on the pupil. We presented arrays of visual stimuli in that differed in luminance, color and set size. Twelve healthy young adult participants viewed either a set size of 1, 4, 16 or 48 dots on the screen in front of them. These dots were white or black and lighter or darker than the background in the luminance condition, or the dots were isoluminant (to the background) blue, yellow, red or green. Participants looks at a central fixation point while chromatic or achromatic stimuli appeared in their periphery. The effects of set size, luminance and color were measured in terms of maximum pupil constriction velocity, maximum constriction magnitude and time of peak constriction.
Results showed that the set size effect could be confirmed in both conditions. Intrinsically photosensitive retinal ganglion cells might contribute to the remarkable pupillary constriction when subjects saw short-wavelength (i.e., blue) light. This constriction was substantially faster and greater compared to long-wavelength (i.e., red) stimuli. The findings of the present experiment are of considerable methodological importance for pupil research in general and should be considered when designing future pupil experiments.

Poster 58

Blink rate, -duration and -synchronization during conversation

Mareike Brych, Fabienne Schmid, Barbara Händel
University of Würzburg

A robust finding in eye research shows an increased blink rate if a person indulges in a conversation compared to quiet rest. Our recent findings suggest that this increase can be caused by motor activity during speaking. We therefore hypothesized a comparable effect on blink rate during speaking compared to listening. Additionally, we investigated other blink characteristics such as blink duration as well as synchronization between people during conversation.
Tracking the eyes of two conversation partners simultaneously, we found an increase of blink rate during speaking compared to quiet rest and listening, however, only the comparison between rest and speaking reached significance. Comparing blink durations revealed that we blink significantly longer during speaking compared to both listening and quiet rest.
In line with previous studies that investigated how a video of a speaker influences listeners' blinks (Nakano & Kitazawa, 2010), we found a higher blink probability in listeners in the time following the blink of a speaker. Contrary to the video-based results, we found a similar relationship when subjects only heard but did not see the speaker. If subjects saw each other without verbal interaction, no increased synchronization was present. Our results suggest that while blink rates might be more strongly influenced by non-cognitive factors like motor output, changes in blink timing indeed seems to underlie cognitive influences. Interestingly, this seems also true for social interactions over the phone.

Nakano, T., & Kitazawa, S. (2010). Eyeblink entrainment at breakpoints of speech. *Experimental brain research,* 205(4), 577-581.

Poster 59

Using eye-tracking to investigate differences in teachers' professional vision IN action and ON action

Zuzana Smidekova *(Department of Educational Sciences, Faculty of Arts, Masaryk University, Brno, Czech Republic; HUME lab, Faculty of Arts, Masaryk University, Brno, Czech Republic)*
Eva Minarikova *(Institute for Research in School Education, Faculty of Education, Masaryk University, Brno, Czech Republic)*
Miroslav Janik *(Institute for Research in School Education, Faculty of Education, Masaryk University, Brno, Czech Republic)*
Kenneth Holmqvist *(Department of Psychology, NKU, Torun, Poland; Department of Psychology, Regensburg University, Germany; Department of Computer Science and Informatics, University of the Free State, South Africa; Faculty of Arts, Masaryk University, Brno, Czech Republic)*

Classroom teaching places great demands on teachers as in each moment there are a number of stimuli competing for the teacher's attention. Teachers' perception, interpreting, and understanding of these situations guides their decisions and is thus of utmost importance in the teaching and learning process. A lot of teacher research has focused on phenomena such as teachers' professional vision (Sherin & van Es, 2009), noticing (Sherin, Jacobs & Philipp, 2011) or ability to notice (Star & Strickland, 2008). So far, most of our knowledge on professional vision has relied on verbal data or questionnaires that used classroom videos as prompts. This has been taken to tell us about teachers' professional vision. Recently, studies explore professional vision during the act of teaching through the use of mobile eye-tracking glasses. In our research, we recorded eye-tracking data through eye-tracking glasses in the act of teaching. After each lesson, we selected short clips from the lesson recorded by a static camera aiming at pupils and showed them to the same teacher (i.e. providing similar setting as traditional studies on professional vision) whilst recording eye-movements and gaze behavior data through screen-based eye-tracker. This gives us a unique opportunity to look at the same situation from two very different points of view - as professional vision IN action and ON action. The results aim to open a discussion about our understanding of professional vision in different contexts and about our existing research on this phenomenon.

Poster 60

Clustering eye movement transition reveals latent cognitive strategies

Kucharský, Šimon; Visser, Ingmar; Truțescu, Gabriela-Olivia; Laurence, Paulo; Zaharieva, Martina; Raijmakers, Maartje

University of Amsterdam; University of Amsterdam; Leiden University; Mackenzie Presbyterian University; University of Amsterdam; University of Amsterdam

In cognitive tasks, solvers can adopt different strategies to process information which may lead to different response behavior. These strategies might elicit different eye movement patterns which can thus provide substantial information about the strategy a person uses. However, these strategies are usually hidden and need to be inferred from the data. We present a relatively easy to apply unsupervised method to cluster eye movement recordings to detect groups of different solution processes that are applied in solving the task. The method uses transition between defined AOIs as an input and produces mean transition probability matrices for each cluster.

We tested the method's performance using simulations to find high classification accuracy. Possible post-hoc analyses, such as quantifying the representativeness of a particular scanpath to a particular group, and relating the groups to other variables are relatively easy to perform with standard statistical software, and their application is demonstrated using two empirical data sets. Our analyses are in line with presence of different solving strategies in a Mastermind game, and suggest new insights to strategic patterns in solving Progressive matrices tasks.

Our poster presentation presents the process and results of the simulated studies and summarises the results from the empirical data.

Poster 61

The horizontal and vertical extensions of the visual span during object search in real-world scenes

Schlachter, Tina Andrea ; Nuthmann, Antje
Kiel University

Using an object-in-scene search task, we investigated properties of the visual span, that is the area around the current point of gaze from which viewers can take in information. Previous studies have measured the visual span using circular gaze-contingent moving windows (e.g., Nuthmann, 2013). Given that visual acuity drops faster along the vertical than along the horizontal meridian (Weymouth, Hines, Acres, Raaf, Wheeler, 1928), we hypothesized that the visual span may have a smaller extension vertically than horizontally. To test this claim, we compared circular windows (radii: 10.5° and 13.2°) and symmetrical oval windows with a no-window baseline condition. Horizontal ovals had the same horizontal extent as the corresponding circle but were vertically compressed by either 20% or 40%. Vertical ovals were created by rotating the horizontal ovals by 90°. They were included to rule out that the size of the visual span may simply be determined by the absolute amount of information available to the observer. For the larger circular window, the mean search time did not differ significantly from baseline. This was also the case for the corresponding horizontal ovals, even with a 40% vertical compression. For the corresponding vertical ovals, however, search times were significantly prolonged. When using the smaller circular window, or any of the corresponding oval windows, search times were also significantly longer than in the no-window control condition. Taken together, the results suggest that the visual span during object search in real-world scenes is more closely approximated by a horizontal oval than a circle.

Poster 62

Eye-movement patterns during the perception of complex visual scenes: The comparison of Czech and Taiwanese samples

Cenek, Jiri ; Tsai, Jie-Li; Sasinka, Cenek
Masaryk University

In the presented research we compared the eye-movement patterns of Czech and Taiwanese university students during the perception of complex static visual scenes. We used a similar research design to designs previously used by Chua et al. (2005), Duan et al. (2016), or Evans et al. (2009). Real world pictures with one or two focal objects on complex backgrounds were presented to the participants, who were supposed to evaluate, how much they liked them, and their eye-movements were recorded. Number and duration of fixations, number of saccades, and focal object - background transitions were calculated. The results suggest the existence of systematic cross-cultural differences in focal object – background visual processing. The Taiwanese fixate the background for a relatively longer time, and make less focal object fixations in both one- and two focal object scenes.

Chua, H., Boland, J., & Nisbett, R. (2005). Cultural variation in eye movements during scene perception. *Proceedings of the National Academy of Sciences of the United States of America, 102*(35), 12629-12633.
Duan, Z., Wang, F., & Hong, J. (2016). Culture shapes how we look: Comparison between Chinese and African university students. *Journal of Eye Movement Research, 9*(6), 1-10.
Evans, K., Rotello, C., Li, X., & Rayner, K. (2009). Short Article: Scene perception and memory revealed by eye movements and receiver-operating characteristic analyses: Does a cultural difference truly exist? *The Quarterly Journal of Experimental psychology, 62*(2), 276--285.

Poster 63

Looking freely or not at bodies that evoke (or not) a gravity conflict: gaze pattern and effect on discrimination performance

Patrice Senot[1], Quentin Gallis[1], Alexandre Milisavljevic[1], Karine Doré-Mazars[1], Julien Barra[2]

1 *Laboratoire Vision Action cognition (EA 7326), Institut de Psychologie, Université Paris Descartes, France*

2 *Laboratoire de Psychologie et Neurocognition (UMR 5105), CNRS Université de Savoie, France*

The visual presentation of inverted bodies leads to poorer discrimination performance compared to upright bodies. This body inversion effect (BIE) (Reed et al., 2003) is taken as an evidence of a deterioration of a configural visual processing of bodies when the image of the body is presented upside-down on the retina. We previously proposed that the BIE could be in part explained by a gravity conflict induced by upside-down bodies (Barra et al., 2017). We indeed observed a reduction of the BIE when using bodies in a handstand posture. Such a posture is not in conflict with gravity direction contrary to usual inverted bodies. However, a recent study (Arizpe et al., 2017) suggested that gaze location is likely to also contribute to the BIE. To examine the possible effect of gaze location on the reduction of the BIE with handstanding bodies, we asked participants to discriminate pairs of body stimuli presented at the center of a screen while recording their eye movements. To more directly elucidate the role of gaze, we allowed the participants either to look freely at the stimuli or to fixate the center of the body during the whole trial. Our preliminary results tend to confirm that participants preferentially gazed at the upper part of the body when it is upright but at a much lower location when it is inverted. Moreover, this pattern tends to be even more pronounced for handstanding stimuli. The relation between this gaze pattern and discrimination performance is discussed.

Poster 64

Investigation of Change Blindness Phenomenon in Terms of High-Level Scene Perception

Türkan, Nilay; İyilikci, Osman; Amado, Sonia
Pamukkale University; Celal Bayar University; Ege University

Every day we come across various thematic scenes and we observe them to gather information about our physical environment. However, eye movements and some environmental occasions take place at any time while we are observing the visual scene. Previous research showed that observers often fail to notice even a large change in the visual scene if it is interrupted by a simultaneous occasion like eye blinks, saccades or a blank screen. This phenomenon is called change blindness. However recent research on change blindness phenomenon revealed contradictory findings regarding the influence of scene-object relations on eye movements and visual attention as well. One reason underlying these discrepant results might be the different procedures and task constraints. In the current study we therefore initially aimed to investigate the effect of the paradigm on change blindness phenomenon during the natural scene viewing. For this purpose, we compared, in the first experiment, two frequently used change detection tasks: flicker and one-shot paradigms. Our results suggested that change detection performance and eye movements varied across the different paradigms. Secondly, we investigated the influence of scene-object syntactic relationships. Finally, we examined the effect of low-level object properties and its interaction with high-level scene processing on the change blindness phenomenon. In three experiments, participants labelled the inconsistent objects commonly with more errors compared to the consistent ones. Our eye movement results emphasize that the attention hypotheses offered by Hollingworth and Henderson (2000) can explain the role of the scene-object relation on CD performance collaboratively.

Poster 65

Spontaneous eye blinks during ambiguous perception

Supriya Murali, Mareike Brych, Liyu Cao, Barbara Händel

Department of Psychology (III), University of Würzburg

Spontaneous eye blinks have been shown to be correlated with the rate of perceptual reversals in ambiguous stimuli. The current study investigated if this association is due to a direct influence of blinks on perception. We used two dynamic bistable stimuli, the ambiguous plaid and the ambiguous motion quartet, and compared the modulation of the perceptual switches (indicated by a key press) around a blink and around a control event (blanking of the display). We found an increase in switches, before a blink but after a blank, for both ambiguous stimuli. When analyzing the temporal modulation of the blink rate we found an increase in blinks during the time of the key press. However, there was no indication that a key press, which is not accompanied by a perceptual switch, would lead to such modulation. These findings indicate, that blinks have no direct influence on the perceptual switch but rather occur in response to perceptual changes. However, we found that not every visual sensory change leads to an increased blink probability. Neither a blank, when not followed by a perceptual switch, nor the switch to component motion in the ambiguous plaid stimulus had an effect on the blink rate. Our results show that spontaneous eye blinks can be a window to perceptual processes.

Reference

Laubrock, J., Engbert, R., & Kliegl, R. (2008). Fixational eye movements predict the perceived direction of ambiguous apparent motion. *Journal Of Vision, 8*(14), 13-13. doi: 10.1167/8.14.13

van Dam, L., & van Ee, R. (2005). The role of (micro)saccades and blinks in perceptual bi-stability from slant rivalry. *Vision Research, 45*(18), 2417-2435. doi: 10.1016/j.visres.2005.03.013

Poster 66

The model of psychophysiological mechanisms of vection illusion

Artem I. Kovalev
Lomonosov Moscow State University, Moscow, Russia

This research was aimed to develop the model of psychophysiological mechanisms of vection, which describes the sensation of ego-motion induced by moving visual stimuli that cover a large part of the visual field. The eye tracking was used to examine parameters of optokinetic nystagmus to identify vection periods which were provoked by the rotating optokinetic drum with black and white stripes. Velocities of rotation were 30, 45 and 60o/s. 14 volunteers passively observed rotating stimulation: 18 trials (3 velocities x 2 directions x 3 repetitions) were presented in pseudorandom order each for 2 min in HTC Vive virtual reality system. Pupil Labs Add-On eye tracker was also used. Participants pressed the keyboard button at the moment of vection perception. Subjective scaling and Simulator Sickness Questionnaire were used to vection strength estimation after each trial. It was found that for all stimuli rotation slow phases durations of nystagmus were significantly longer compared to non-vection periods ($F = 44,5$, $p < 0,01$). Vetion strength values were the highest during 60 o/s rotation. It is suggested that optokinetic nystagmus may be considered as an efferent part of the space orientation brain system. The increase in velocity made it difficult to perform the pursuit eye movements. The disturbances in nystagmus which occurred due to nystagmus habituation led to the sensory conflict manifestation and also to the increase of vection strength. The research was supported by the grant RSCF №19-18-00474.

Poster 67

Visual Search on a Flight-booking Site: Effects of Task Complexity on Search Time, Errors, Eye and Cursor Movements

Blinnikova I., Burmistrov I., Grigorovich S., Zlokazova T.

Lomonosov Moscow State University

This study simulated the search by specified parameters on flight-booking sites. The subject was asked to choose an outward flight and an inward flight according to the specified parameters. Task complexity exerting different working memory loads on two levels: (a) simple task: The two parts of each task (search for an outward and inward flight) are not connected; (b) complex task: Both parts of the task are connected: the subject has to keep in his working memory the results of the first part of the task in order to carry out the second part. Participants were 23 University students. They carried out 16 tasks - 8 simple and 8 complex (368 trials in total). The results demonstrated the influence the task complexity factor on the search time and the number of errors, as well as on the average duration of eye fixations. Increasing the complexity of the task adds about 20 ms to the average duration of fixations (347.14 vs 368.74 ms). In general, these results confirm the data obtained in other studies and represent an increased cognitive load. The complexity of the task determined the number of mouse movements and the amplitude of the cursor movement. In complex tasks the discrepancy between the eye and the cursor decreased in comparison to simple tasks. These results can be regarded as evidence of a significant role of the mouse cursor as an auxiliary tool in solving complex search tasks on sites. The research is supported by RFBR project 17-06-00652.

Poster 68

The Eye-Tracking Measurement and Cartographic Stimuli

David Lacko[a,b], Čeněk Šašinka[a], Stanislav Popelka[c], Zdeněk Stachoň[a]

a. *The Division of Information and Library Studies, Faculty of Arts, Masaryk University, Arna Nováka 1, 602 00 Brno, Czech Republic.*

b. *Department of Psychology, Faculty of Arts, Masaryk University, Arna Nováka 1, 602 00 Brno, Czech Republic.*

c. *Department of Geoinformatics, Faculty of Science, Palacký University Olomouc, 17. listopadu 50, 771 46 Olomouc, Czech Republic.*

The following poster presents an exploratory research of eye-tracking results conducted on 37 Czech participants. The poster introduces unique cartographic stimuli created on the basis of cognitive psychology research of categorization and clustering (Ji, Zhang & Nisbett, 2004; Norenzayan, Smith, Kim & Nisbett, 2002). The theoretical background of the developed cartographic tasks stems from the cross-cultural research of the cognitive style, especially analytic and holistic cognitive style (see Nisbett, Peng, Choi & Norenzayan, 2001), where the eye-tracking measurement is often performed (e.g. Chua, Boland & Nisbett, 2005). Therefore, the performance within the cartographic stimuli shall be understood as behavioural manifestation of the cognitive style in this case. These tasks are characterized by the categorizing (respectively clustering) of multivariate point symbols in the fictional cartographic maps with preselected analytic/holistic area (i. e. areas of interest in the eye-tracking analysis). The poster presents fixations and gaze points, saccades, heatmaps, average time spent and scanpaths with respect to preselected areas of interest. Data were gathered by Eye Tribe and SW Ogama. The contribution also discusses the limitations of the presented method and its potential for the deeper understanding of the cognition and perception differences in categorization processes.

References

Ji, L., Zhang, Z., & Nisbett, R. (2004). Is it culture or is it language? Examination of language effects in cross-cultural researchon categorization. *Journal of Personalityand Social Psychology, 87*(2), 57–65.

Masuda, T., & Nisbett, R. (2006). Culture and change blindness. *Cognitive Science, 30*(2), 381-399.

Nisbett, R., Peng, K., Choi, I., & Norenzayan, A. (2001). Culture and systems of thought: holistic versus analytic cognition. *Psychological Review, 108*(2), 291-310.

Norenzayan, A., Smith, E., Kim, B. & Nisbett, R. (2002). Cultural preferences for formal versus intuitive reasoning. *Cognitive Science, 26*(5), 653–684.

Poster 69

Detection of Pupillary Light Response in The Remapped Area

Mikheev, Vladimir; McInnes, Joseph
NRU HSE

Predictive visual remapping is an anticipating activation of retinotopic parietal neurons to the impending saccade and the pupillary light response is a reaction of pupil to changes in luminance. Mathot (Mathôt, 2015) and Binda (Binda, Pereverzeva, & Murray, 2014) described techniques to control pupil size and measure its reaction on light. Rolfs (Rolfs, Jonikaitis, Deubel, & Cavanagh, 2011) demonstrated short attentional benefit in remapped area in double-step saccade task. However, interaction of these two phenomena is not well-documented.

We hypothesize that attentional benefit in remapped area could affect pupil size if that area lands on the side with opposite brightness regarding last fixation in double-saccade task. In our study we use a split-screen condition with contrasting luminance of sides where participants should do double-saccade depending on an auditory cue. We expect to see pupillary response in remapped area (PRRA) in parallel saccade programming controlled by gaze-contingent demonstration of stimuli. We analyzed data from a pilot study that included gaze contingent luminance shifts, in addition to parallel and sequential saccade programming. Early results suggest sequential condition pupil size changes weaker than in parallel, but no differences for luminance shifts. Linear mixed-model demonstrates very robust effect of saccade programing order ($p < 1.23e-11$) during 400 msec. after audial cue.

References

Binda,P., Pereverzeva,M., & Murray,S. (2014). Pupil size reflects the focus of feature-based attention. *Journal of Neurophysiology, 112*(12), 3046–3052.

Mathôt,S. (2015). *The Pupillary Light Response Reflects Eye-Movement Preparation. 8.*

Rolfs,M., Jonikaitis,D., Deubel,H., & Cavanagh,P. (2011). Predictive remapping of attention across eye movements. *Nature Neuroscience, 14*(2), 252–256.

Poster 70

The effect of rhythmical violations on the pupillary response in unattended beat perception.

Ciesielski K.[1]**, Lovell G.**[2]**, Scott Brown K.**[3]
1,2,3 University of Abertay Dundee, Scotland
CiesielskiKry@gmail.com

It is far from resolved how unattended information operates in the multimodal attentional system. One of the aspects of this system is the perception of auditory stimuli as complex interactions between its elements and their subjective interpretation. For example, beat perception depends on various subdivisions of rhythm and listeners' ability to infer regular pulse from it. However, it is still unknown how beat perception operates in the absence of attention, and how it relates to the subjectively established hierarchy of salience or meter. The current study addressed those questions by use of pupillometry. We investigated the pupillary response to omissions with three different salience levels in drum beats (high salience, low salience and polyrhythmic change), while participants (n=20) attended to a filler vigilance task. It was hypothesised that there would be significant pupil dilation to all rhythmical violations and that there would be a significant difference between pupillary responses that would correspond to the levels of salience. The results demonstrated that pupils dilate as a response only to the high salience rhythmic violation. Contrary to Damsma and van Rijn (2017), the results suggest that there may be a threshold to extend the unattended auditory information being reflected by the pupillary response. Overall, the findings suggest that pupils dilate as a response to the violation of expectation and indicates unattentive auditory processing.

Poster 71

Re-evaluation of luminance evoked pupil response dynamics

Jonathan D Coutinho, Jeff Huang, Philippe Lefevre, Gunnar Blohm, Douglas P Munoz
Queen's University; Queen's University; Université catholique de Louvain; Queen's University; Queen's University

Pupil responses are commonly used in clinical assessments and as a proxy for cognitive processes in psychological research. Making accurate inferences from these measures requires precise knowledge of the underlying system dynamics. However, the precise system dynamics of pupillary responses are poorly quantified. As a result, there is a lack of consistency in the selection of pupil metrics and the preprocessing and analysis of pupil response data across the literature. Meanwhile, existing pupil models rely on simplistic assumptions of underlying control signals, resulting in poor generalizability. Thus, better quantification of the control system and neuromuscular properties of pupil response dynamics would substantially advance the utility of pupillometry in cognitive and clinical neuroscience research. Here we quantify pupil responses in healthy young adults (age range, participant #) to the simplest possible sensory stimulus, i.e. large-field changes in luminance, randomly selected between 1 and 43 cd/m2. We found large variability in baseline pupil sizes within and between subjects. Nevertheless, we found a linear relationship between average changes in pupil size and the difference in log-luminance during a luminance transition. Furthermore, we found covariance between the amplitude and peak velocity of pupil responses suggestive of a "main sequence" in the pupil control. We quantified aspects of dynamic pupil responses, including saturating non-linearities and asymmetries between constriction and dilation dynamics. These results provide a better foundation to link pupil dynamics with cognitive processes and clinical biomarkers.

Poster 72

Language models can outperform empirical predictability in predicting eye movement data

Markus J. Hofmann[1*], Steffen Remus[2], Chris Biemann[2], & Ralph Radach[1]
1 General and Biological Psychology, University of Wuppertal
2 Language Technology, University of Hamburg
** Email: mhofmann@uni-wuppertal.de*

In our talk at the last ECEM, we used n-gram models, topic models, and recurrent neural networks in generalized additive model (GAM) analyses to predict gaze duration data of the Potsdam sentence corpus (Kliegl, Grabner, Rolfs, & Engbert, 2004, European Journal of Cognitive Psychology, 16, 262-284). In the present poster, we extend our analyses to the eye-movement corpus of Schilling, Rayner, and Chumbley (1998, Memory & Cognition, 26, 1270-1281), as well as single-fixation and total-viewing-time data. Our results show that in many cases, language-model-based GAMs provide a significantly greater log likelihood than GAMs based on a set of 'classic' predictor variables relying on cloze-completion-based predictability. Because these algorithmic language models make encoding, consolidation and retrieval explicit, this opens up new levels of understanding semantic memory and its impact on reading performance beyond lab scenarios.

Poster 73

Font matters: efficient adaptation to monospaced vs. proportional fonts is accompanied by effect-size differences for word frequency and predictability

Julian Jarosch, Matthias Schlesewsky, Stephan Füssel & Franziska Kretzschmar
Johannes Gutenberg University Mainz; University of South Australia; Johannes Gutenberg University Mainz; University of Cologne

Monospaced fonts, though technologically obsolete, are present on all computers—and much reading research uses them (Hautala et al. 2011), since their constant letter width (achieved mostly through expansion) equates physical and orthographic length. Paterson and Tinker (1932) indicate that monospaced (opposed to normal, proportional) fonts decrease text reading speed, while Rayner et al. (2010) observe a constant single sentence reading rate.
To consolidate how these font types compare in eye tracking, 32 participants read 112 single sentences orthogonally manipulated for font style (serif typewriter vs. sans-serif Antiqua) and proportionality (monospaced vs. proportional). We analysed the entire sentence and a target word add itionally manipulated for lexical frequency and predictability (each low vs. high).
Linear mixed-model analysis reveals that at the sentence level, monospacing increases the number of fixations, while decreasing the mean fixation duration, resulting in an unchanged total reading time. Mean saccade length in pixels increases, conforming to monospacing's expansion, while saccade length in characters decreases: saccade planning does not wholly compensate the font expansion.
On the target word, monospacing decreases first fixation duration, go-past time, and skipping probability. As before, reading times decrease and fixation density increases, with constant total reading time. Additionally, proportionality interacted with frequency and predictability in reading time measures. The typical fequency effect (low > high) was larger in monospacing, whereas the predictability effect (low > high) was reduced.
Overall, this suggests that a lthough the oculomotor system adapts to monospaced fonts efficiently, generalizing from monospaced to proportional fonts may not be a simple quantitative scaling as regards effect size.

Hautala, J., Hyönä, J., & Aro, M. (2011). Dissociating spatial and letter-based word length effects observed in readers' eye movement patterns. *Vision research, 51*(15), 1719–1727.
Paterson, D. G., & Tinker, M. A. (1932). Studies of typographical factors influencing speed of reading. X. Style of type face. *Journal of Applied Psychology, 16*(6), 605–613.
Rayner, K., Slattery, T. J., & Bélanger, N. N. (2010). Eye movements, the perceptual span, and reading speed. *Psychonomic bulletin & review, 17*(6), 834–839.

Poster 74

Eye movements during reading and cognitive functions in elementary school students

Korneev A., Matveeva E., Akhutina T.
(Lomonosov Moscow State University)

An analysis of reading-related eye movements is helpful in uncovering the cognitive structure of the reading skill. There are different views on the role of various cognitive functions, such as executive functions or the processing of visual-spatial and auditory information when reading. In order to investigate how the relative contributions of these functions into the reading skill change in a course of development we analyzed eye movements during reading in schoolchildren of the second and third grades. A total 75 of second and 56 third graders participated in the study. Every child read a corpus of sentences, which has been developed for eye-tracking studies of reading in Russian children. All children were also assessed neuropsychologically in order to estimate their individual states of executive functions and functions of visuospatial and auditory information processing.

We correlated the number of fixations and their duration with the results of neuropsychological assessment in two age groups (second and third graders) separately. This analysis showed that (i) in both groups the executive functions scores correlated with numbers of fixations and their durations, (ii) the visuospatial information processing scores are statistically related to eye movements only in the group of second graders, but not in the group of third graders, and (iii) on the contrary, the auditory information processing correlated with the reading-related eye movements in the third graders, but not in the second graders.

The obtained results suggest that the cognitive structure of reading skill does change over the age period we studied.

Poster 75

Bayesian inference of the SWIFT model: Reading mirrored, scrambled, and normal texts

Maximilian M. Rabe, Johan Chandra, André Krügel, Stefan A. Seelig, & Ralf Engbert
Research Focus Cognitive Science and Department of Psychology, University of Potsdam, Potsdam, Germany

In dynamical cognitive modeling of eye movements during reading, SWIFT [1] has recently been among the first models to be proposed and implemented for Bayesian parameter estimation [2]. As presented here, the model was validated with data previously collected that were known to exhibit a broad range of reading behavior in response to the presentation of normal, mirrored and scrambled text [3]. After fitting SWIFT on a training subset on a by-participant basis, for the complementary test subset, statistical quantities such as saccade amplitudes, fixation durations and probabilities were derived by participant from empirical and simulated fixation sequences. High correlations and low deviation between predicted and empirical quantities indicate high goodness-of-fit. A statistical analysis of fitted model parameters between experimental conditions, as briefly presented herein, could serve as an explanation for a number of relevant experimental effects. As a result, the SWIFT model could be successfully validated as a viable model for describing, explaining and predicting eye movements in reading and corresponding interindividual differences under a broad range of experimental manipulations.

References:

[1] **Engbert, R. et al.** (2005). SWIFT: A dynamical model of saccade generation during reading. *Psychological Review, 112*, 777-813.
[2] **Seelig, S. et al.** (2019). *Bayesian parameter estimation for the SWIFT model of eye-movement control during reading*. Manuscript submitted for publication. arXiv:1901.11110.
[3] **Chandra, J. et al.** (2019). *Reading mirrored texts*. Manuscript in preparation.

Acknowledgments: This work was supported by Deutsche Forschungsgemeinschaft via Collaborative Research Center 1287 “Variability in Language” (Project no. 317633480 - SFB 1287, Projekt B03).

Poster 76

Ocular strategies analyses during reading through a text comprehension model

Etienne Langevin[1,2], Anne Guérin-Dugué[2], Aline Frey[3], Benoît Lemaire[1]

1 *Univ. Grenoble Alpes, Univ. Savoie Mont Blanc, CNRS, LPNC, 38000 Grenoble, France*

2 *Univ. Grenoble Alpes, CNRS, Grenoble INP*, GIPSA-lab, 38000 Grenoble, France*
** Institute of Engineering Univ. Grenoble Alpes*

3 *CHArt Laboratory, ESPE of the Créteil Academy, University of East-Paris Créteil, France*

Strategies controlling reading saccades throughout a text depend on many factors including semantic ones. The construction-integration comprehension model (Kintsch,1998) is used to simulate the time course of the semantic integration process of participants reading texts. This model is based on a Latent Semantic Analysis (Landauer et al., 2007) of a huge text corpus, enabling automatic semantic comparison of words.
The eye-tracking data of 28 participants reading 180 texts was used to characterize the optimal human way of text processing. Participants were instructed to read a text and to decide whether or not it was related to a given topic.
Using this model, the simulation of the progressive semantic integrations of each fixated word was compared to a fictional reader processing all words and to a fictional random reader processing words located at random. The simulation results show that the human way of performing ocular saccades saves time without altering the construction of the overall meaning of the text.
This fixation-based text comprehension model is a powerful tool to investigate the relationship between the saccade progression within a text and the gradual construction of its general meaning.

Poster 77

The Semantic Transparency Effect and Aging during Chinese Reading: An Eye Movements Study

Kuo Zhang[1], Min Chang[2], Shasha Pan[2], Lin Li[2], Jingxin Wang[2]
1. Nankai University, China.
2. Tianjin Normal University, China.

Most Chinese words are two-character compound words with each character conveying both semantic and phonological information, thus semantic transparency of compound words is potentially important with respect to the nature of word identification in Chinese. Several previous research showed there is a processing advantage for transparent words over opaque words via behavioral methods in which the words were presented in isolation. However, less is known about whether this kind of transparency effect exists during natural Chinese reading and how the effect change with aging. In the current study, eye movements of young or old adults were monitored while the participants read sentences with transparent or opaque two-character compound words. LMMs were used to analyze the data. The experiment found transparent target words were read faster compared with opaque words, and these effects emerged in first fixation duration, single fixation duration and total reading time. These results demonstrated the processing advantage for transparent words over opaque words during natural reading, thus provided evidence for the Multi-Level Representation Model via eye-tracking method. Moreover, the interactions between semantic transparency and age group were also obtained on first fixation duration, single fixation duration which indicated the transparency effect is more salient for old adults compared with young adults.

Zhou, X., Marslen-Wilson, W., Taft, M., & Shu, H. (1999). Morphology, orthography, and phonology in reading Chinese compound words. *Language and Cognitive Processes, 14*(5–6), 525–565.

Poster 78

The influence of context on the English polysemous word processing of Chinese-English L2 users: using visual world paradigm

How L2 readers understand the appropriate meaning of polysemous words is an important research topic. This study manipulated the domain-biased, secondary-biased, and neutral-context leading to explore how the English-as-L2 college students extract the domain- and secondary-meaning. This study collected the English vocabulary abilities of 28 college students through pronunciation-rule test, word-meaning test and speak-fluency test, and asked participants to read 10 leadings of each biased-type on the screen (a total of 30 leadings which stop at the target word) and to select the meaning of the target word in 4 words. Participants' eye movements in the reading stage and the visual search stage were recorded using eye tracker. The results show that: 1. In the pre-reading stage, the main words have the processing advantages and different English ability responses in different reading performance; 2. In the visual search stage, it was found that when biasing to domain-meaning, the English-as-L2 readers do not activate the secondary-meaning, and when the secondary-meaning was biased, readers suppressed the domain-meaning first, but then it was activated late. When the neutral context is presented, the domain- and secondary-meaning were both activated, but the domain-meaning had a higher fixed rate than secondary-meaning. Although this study provides the data of Chinese-English users on the processing of English polysemy, but still need further researches to provide native English user data or other English-as-L2 user data for having better understand the processing of polysemous words during reading.

Poster 79

Are L33T words processed as the original words in the parafovea?

Fernández-López, María; Marcet, Ana; Perea, Manuel
Universitat De València; Universitat De València; (1) Universitat De València (2) Universidad De Nebrija

Leet words are easily confusable with their base words (e.g., M4T3RI4L[illegible]MATERIAL). Using masked priming, Perea, Duñabeitia, and Carreiras (2008) found faster word identification times to pairs like M4T3RI4L-MATERIAL than to control pairs like M6T8RI6L-MATERIAL. Furthermore, leet primes behaved as identity primes. This pattern has been replicated in various labs (e.g., Lien, Allen, & Martin, 2014). However, in sentence reading, Duñabeitia, Perea, and Carreiras (2009) found a sizeable cost for sentences written with leet words relative to the original sentences in all eye movement measures.
To examine this discrepancy, we designed a sentence reading experiment using a gaze-contingent boundary change paradigm. The parafoveal previews were: 1) the same as the target (FAMILIA-FAMILIA); 2) a leet stimulus (FAM1L1A-FAMILIA); or 3) a control stimulus (FAM2L2A-FAMILIA). For both first-fixation durations and gaze durations, we found a small advantage of the identity condition over both leet and control conditions. Critically, there were no differences between the leet and control conditions. Thus, parafoveal (uppercase) leet words may not provide sufficiently fined-grained information to facilitate the processing of the target words.

References

Duñabeitia, J.A., Perea, M., & Carreiras, M. (2009). Eye movements when reading words with $YMβOL$ and NUM83R5: There is a cost. *Visual Cognition, 17*, 617–631.
Lien, M.C., Allen, P., & Martin, N. (2014). Processing visual words with numbers: Electrophysiological evidence for semantic activation. *Psychonomic Bulletin & Review, 21*, 1056–1066.
Perea, M., Duñabeitia, J.A., & Carreiras, M. (2008). R34D1NG W0RD5 W1TH NUMB3R5. Journal of Experimental Psychology: *Human Perception and Performance, 34*, 237–241.

Poster 80

Compound processing in second language speakers of Finnish

Rosa Salmela[1], Minna Lehtonen[2], Raymond Bertram[3]
[1]Åbo Akademi University, Finland, [2]University of Oslo, Norway, [3]University of Turku, Finland

Several studies have shown that compound processing is affected by lexical properties such as word length and morpheme boundary cues. For instance, the length of compound words modifies the extent to which constituents are used in processing (Bertram & Hyönä 2002). There is also evidence that compound parsing differs for native and nonnative speakers, as nonnative speakers are more likely to use orthotactic parsing cues than native speakers (Lemhöfer et al. 2011). In this study, we investigated to what extent relatively short (mean length 9 characters) and high-frequency compounds (merivesi 'sea water' or tyttöystävä 'girlfriend') are processed via constituents by beginner level L2 speakers in comparison to native speakers. Eye movements were registered during reading of compounds presented in sentence context. Several eye movement measures showed significant whole-word frequency effects for both L1 and L2 speakers. Moreover, the L2 speakers' processing was affected by constituent frequency, whereas L1 speakers' processing was not. Taken together, the results suggest that constituents have a bigger role in processing relatively short Finnish compounds for L2 speakers than for natives. This suggests that the developmental trajectory to process complex words moves from decomposition to whole word processing (cf. Portin et al., 2007) rather than from whole word processing to decomposition as suggested by the shallow structure hypothesis (e.g., Clahsen & Felser, 2006) or the declarative/procedural model of Ullman (Ullman, 2001).

References

Bertram, R., Hyönä, J. (2003). The length of a complex word modifies the role of morphological structure: Evidence from eye movements when reading short and long Finnish compounds. *Journal of Memory and Language*. Volume 48, Issue 3.

Clahsen, H., & Felser, C. (2006). Grammatical processing in language learners. *Applied Psycholinguistics*, 27(1), 3-42.

Lemhöfer, K., Koester, D. & Schreuder, R. (2011). When bicycle pump is harder to read than bicycle bell: effects of parsing cues in first and second language compound reading. *Psychon Bull Rev, 18*: 364.

Portin, M., Lehtonen, M., & Laine, M. (2007). Processing of inflected nouns in late bilinguals. *Applied Psycholinguistics, 28* (1), 135–156.

Ullman, M.T. (2001). The Declarative/Procedural Model of Lexicon and Grammar. *Psycholinguist Res* 30: 37.

Poster 81

Underlining words during sentence reading: To be or not to be

Manuel Perea [(1)], **Stéphanie Massol** [(2)], **Alberto Llorens** [(1)], **and Víctor Costumero** [(3)]
(1) Universitat de València
(2) Université Lumière Lyon 2
(3) Universitat Pompeu Fabra

Underlining is employed to add emphasis to some of the key words in a text. Here we designed an experiment to examine whether underlining influences eye movement control during reading. Recently, Gagl (2016) found similar first-fixation durations and gaze durations on a target word when presented underlined and when presented in the standard format. Critically, go-past times were longer for underlined target words than for non-underlined target words—this was interpreted in terms of extra allocation of attention. The lack of a word-frequency manipulation, however, makes it difficult to draw strong conclusions on the interplay between underlining and lexical access.
In the current experiment, a target word of high vs. low frequency was embedded in each sentence. This target word could be underlined or not. Results showed a sizeable word-frequency effect in all eye fixation measures. We found no signs of an effect of underlining in first-fixation durations or gaze durations, but we found longer go-past times for underlined words than for non-underlined words. Furthermore, skipping rates on the target word were lower for underlined words than for not-underlined words (see Fitz. Weal, & Drieghe, 2019, for a similar finding with colored words).
Taken together, these findings suggest that underlining has little effect during lexical access. Nonetheless, underlined words may induce more attentional resources at some stages of processing. This is probably related to the fact that highlighted words typically play an important role in the text.

Poster 82

Relating foveal and parafoveal processing efficiency with global eye movement parameters in reading

Timo T. Heikkilä and Jukka Hyönä
University of Turku, Finland

In the present study, we examined correlation between foveal and parafoveal processing efficiency and global eye movement parameters in reading. Foveal processing efficiency was assessed by the performance accuracy in a speeded lexical decision task, where 5-7 –letter words and pseudowords were presented backward-masked for identification using exposure times of 80, 60, 40 and 20 ms. Parafoveal processing efficiency was assessed by a lexical decision task, where 5-letter words and pseudowords were presented for 120 ms .91, 1.33, 1.83 and 2.24 degrees of visual angle to the right and left of the fixation point. Participants read silently for comprehension 8 popular science texts on different topics. Average forward saccade amplitude, forward fixation duration, forward number of fixations and regression rate were used as global indices of reading efficiency. Preliminary results showed a moderate positive correlation for forward saccade amplitude with foveal processing efficiency but no correlation with parafoveal processing efficiency. Average forward fixation duration displayed a weak positive correlation with parafoveal processing efficiency but not with foveal processing efficiency. Number of forward fixations demonstrated a weak negative correlation with foveal and parafoveal processing efficiency. Regression rate displayed a weak negative correlation with parafoveal processing efficiency but not with foveal processing efficiency. Finally, foveal processing efficiency correlated strongly with the efficiency of parafoveally recognizing words presented in the right visual field but weakly with the efficiency of parafoveally recognizing words presented in the left visual field. Before drawing firmer conclusions, the observed correlations will be confirmed with additional data.

Poster 83

Taipei Sentence Corpus of Mandarin Chinese: Analysis of an Eye-Tracking Corpus for Age Differences in Reading Chinese sentences

Jie-Li Tsai, Tzu-Hsuan Lin, I-Ju Chen
Department of Psychology, National Chengchi University, Taiwan
Research Center for Mind, Brain, and Learning, National Chengchi University, Taiwan

The present study introduced the TSC, Taipei Sentence Corpus, a corpus of eye movements of 243 participants with the age varied from 20 to 90 reading 160 Chinese sentences. For the TSC, the sentences contained 2246 words with the length ranged from one to four characters and the word predictability for each word was obtained in a contingent predictability rating study using the word cloze procedure. The fixed effects of word length, word strokes, word frequency, and word predictability, as well as the interactions with the age on single fixation duration, gaze duration and total viewing time, were estimated with the linear mixed models.
The results demonstrated the effects of word length, word frequency, and word predictability as the sentence corpora of other languages did. The interactions between word frequency and word predictability were significant on gaze duration and total viewing time. The properties of the previous and upcoming words also showed the strong influences on all measures and these effects were larger on the previous word. Regarding age differences, the interactions with the lexical effects were significant and most of the effects on the current and previous words were enhanced as a function of the age. For the upcoming words, the word predictability effect was increased as the age increased but an decrease of the word frequency effect as the age increased. The findings suggest that, comparing with young readers, the older readers were less efficient in lexical processing of words and relied more on the context in reading Chinese.

Poster 84

Phrasal Order and Ageing: Does the 'Wrong and Right' Order of Phrases Become Irreversible Over Time?

Barton, Hayley ; Paterson , Prof Kevin ; McGowan , Dr Victoria
University of Leicester

Previous research has shown that readers are highly sensitive to word order in common phrases, and show a strong processing preference for such phrases to be presented in a familiar (e.g., fish and chips), rather than an unfamiliar (e.g., chips and fish) order (Siyanova-Chanturia et al., 2011). These findings suggest that the order of words in many common phrases becomes fixed in memory through the experience of reading.
Within the present experiment, we used measures of eye movements to assess this word order effect for young adult readers (aged 18-30 years) compared to older adult readers (aged 65+ years). The rationale for this comparison is that older adults may have a stronger processing preference for the familiar over unfamiliar word order as a consequence of their greater experience of reading and, if compared to young adults, they make greater use of contextual knowledge to recognise words. The results show that unfamiliar word order disrupted both early and late processing of phrases relative to familiar word order. However, this effect did not differ across adult age groups. It therefore appears that, once learned, expectancies for words to appear in a certain order in phrases remain stable across the adult lifespan.

References

Siyanova-Chanturia, A., Conklin, K., & Van Heuven, W. J. (2011). Seeing a phrase "time and again" matters: The role of phrasal frequency in the processing of multiword sequences. *Journal of Experimental Psychology: Learning, Memory, and Cognition, 37*(3).

Poster 85

Scanning strategies of visual semantic search in Russian, Japanese and Chinese subjects

Rabeson M., Izmalkova A., Kirsanova S., Meliksetian A., Raevskiy A., Blinnikova I.
Lomonosov Moscow State University

The current study is aimed at identifying eye-movement patterns in readers of different native language backgrounds. Following K.Rayner (1998), we analyze whether the visual semantic search strategies are influenced by the reading skills, acquired earlier. The hypothesis was that the task scanning patterns are guided by the previous experience of reading in different writing systems. The participants (19 Japanese, 20 Chinese and 45 Russian students) had to look for meaningful English words in letter matrices (15*15). The task required the item-by-item analysis, letter string construction and lexical decision. The number of the found words and eye movements were recorded (with SMI RED 250 system). The results show that Japanese- and Chinese – speaking participants experience more difficulty in reconstructing words from non-ordered set of separate letters. The combination of intersaccadic angle and saccade direction measures (Blinnikova & Izmalkova, 2017) were used to differentiate between sequential and non-sequential patterns, applied by the subjects. Russian-speaking participants tended to use “horizontal sequential” pattern, which is characterized by horizontal saccades from left to right with little change of direction, whereas Chinese- and Japanese-speaking participants made more vertically-oriented saccades (although saccades with horizontal direction still dominated). $F(2;805)=30.4$; $p<0.05$ for intersaccadic angles 0°-45°, $F(2;805)=10.4$; $p<0.05$ for right-upward direction and $F(2;805)=10.2$; $p<0.05$ for upward-left direction. Japanese-speaking participants also showed significantly more backwards-directed saccades (135°-180°) than Russian and Chinese subjects ($F(2;805)=22.7$; $p<0.05$). This difference in visual search can be attributed to the previously formed strategies of verbal material processing. The research is supported by RFBR project 18-013-01240.

Poster 86

Prediction error costs in reading

Sarah Schuster[1], Klára Géciová[2], Florian Hutzler[1] & Stefan Hawelka[1]

1 *Centre for Cognitive Neuroscience & Department of Psychology, University of Salzburg*

2 *Department of Psychology, University of Surrey*

Recent evidence suggests that unpredictable words in constraining contexts do not result in slower reading times than words in neutral contexts. Thus, the presence of another, more predictable word does not inflict a *prediction error cost*. We probed this assumption by manipulating the visuo-orthographic similarity of high and low associated words in an adjective-noun paradigm. We used corpus-based association norms as a proxy of word predictability for target-nouns based on preceding adjectives. The pairs were then presented as either semantically compatible (e.g., *rotten wood*; base form), or semantically less compatible, but with visuo-orthographically similar (e.g., *rotten wool*), or dissimilar (e.g., *rotten cash*) noun versions of the base form. Participants silently read these pairs which they, subsequently, rated according to their meaningfulness of appearing together. Our findings demonstrate shorter fixation durations for high than low associated semantically compatible nouns. Semantically less compatible nouns received longer fixation durations than compatible nouns. Critically, high associated visuo-orthographic similar nouns elicited longer fixation durations than their low associated counterparts. We interpret our findings in terms of Bayesian belief updating: as previously suggested, the transition of a prior to a posterior belief as each word is encountered may already start when attention is allocated to the parafoveal word. In case of high associated compatible and incompatible, but visuo-orthographic similar nouns, a readers' prior belief might be supported by parafoveal information. When subsequently fixating visuo-orthographic similar, yet incompatible words, greater belief updating would have to be (re-)initiated, thus leading to a *prediction error cost*.

Poster 87

Is there any difference between processings of sortal classifiers and mensural classifiers in Chinese Reading?

Xuejun Bai, Siqi Feng, Manman Zhang

Academy of Psychology and Behavior, Tianjin Normal University, Tianjin, 300074

In Sino-Tibetan language (e.g., Chinese), when nouns are modified by numerals, classifier is essential (i.e., sortal classifiers or mensural classifiers). It has been provided some neuroimaging evidence that the quantity representations can be activated while processing Chinese classifiers. Sortal classifiers means one while mensural classifiers mean more than one. However, it is unclear about the difference between the processing of mensural classifiers and sortal classifiers, and how the numeral influences the processing of classifiers in natural Chinese reading.

In the present study 52 nouns were chosen with a preceding numeral word (means large or small) and classifier word (sortal or mensural). Eye movements of 40 Chinese native speakers were recorded by using the Eyelink 1000 plus while reading sentences with embedded numerals, classifiers and the nouns. Classifier word analyses showed that when the numeral means large, single fixation duration and total reading time on mensural classifiers were longer than those on sortal classifiers. However, there was no significant difference in reading times between mensural classifiers and sortal classifiers when the numeral means small. This suggests that longer processing is needed for mensural classifiers than sortal classifier with a “larger” meaning of numeral.

Keywords: Chinese reading, sortal classifiers, mensural classifiers, eye movements,

Poster 88

Context effects and spoken word recognition of Chinese: further eye-movement data

Michael C. W. YIP
Department of Psychology
The Education University of Hong Kong

An eye-tracking experiment, with a novel experimental design, was conducted to further examine the effects of sentence context and tonal information on spoken word recognition processes in Chinese. Sixty native Cantonese listeners were recruited to participate in the eye-tracking experiment. In this experiment, listeners were asked to listen carefully to a spoken sentence, ended with an ambiguous word (Chinese homophone), and look attentively at different Chinese characters or different pictures presented on the computer screen. The target ambiguous words were manipulated to either (a) match to the context (i.e. the correct tone that fitted the sentence context) or (b) not (i.e. the incorrect tone that did not fit the sentence context) in the experiment. The ambiguous Cantonese words that fitted and those did not fit the sentence context differed in the particular tone they carry: the former category carried the correct tone and the latter category the incorrect tone; for example, we put the ji6 instead of ji2 at the end of the sentence context, in which originally semantically biased ji2. By using the linear mixed-effects modeling, the results of eye-movement pattern revealed that (1) sentence context had an early effect on the disambiguation processes; (2) sentence context interacted with tone change of the ambiguous word during lexical access; and (3) phonological information of the distracters had a comparatively weak effect on the spoken word recognition processes. Finally, the patterns of eye-tracking results further support the interactive approach in spoken word recognition (Yip & Zhai, 2018a; 2018b).
Keywords: Context effects, Spoken word recognition, Chinese homophone, lexical access, eye-tracking

References

Yip, M. C. W. & Zhai, M. (2018). Processing Homophones Interactivity: Evidence from eye-movement data. *Scientific Reports, 8*, 9812.

Yip, M. C. W. & Zhai, M. (2018). Context Effects and Spoken Word Recognition of Chinese: An Eye-Tracking Study. *Cognitive Science, 42*, 1134-1153.

Poster 89

Can the solution strategy in different mental-rotation paradigms be identified via eye-movement patterns?

Claudia, Quaiser-Pohl; Mirko, Saunders
University of Koblenz-Landau

Mental-rotation is the ability to rotate three-dimensional objects in mind and to decide whether they are identical or different. It is one of the most often studied paradigms in cognitive psychology since Shepard and Metzler (1978) described it for the first time. Many studies revealed that the mental-rotation ability is crucial for being successful in the STEM field. Individual differences in mental-rotation performance, however, mainly depend on the strategies used for solving the test items. In two studies we used an eye-tracking approach in order to identify the gaze patterns of the particpants when they solved the mental-rotation tasks. The tasks either consisted of cube figures or of gender-stereotyped stimuli. The software Blickshift was used to analyze the gaze patterns. Results showed four different gaze patterns with the gender-stereotyped stimuli which were related to the characteristics of the items as well as to item difficulty and individual characteristics of the test persons (i.e. gender, experience, attention). We were also able to confirm important item characteriistics in the eyetracking data like egocentric and object-based mental rotation. These patterns were partially replicated with the cube-figure items, here mainly with regard to gender differences. However, further research is needed to verify the relatedness of the gaze patterns to the underlying cognitive processes.

Poster 90

The critical temporal delay for gaze-contingent multiresolutional displays to lose the sense of agency and controllability of gaze control

Kim, Junhui; Yoshida, Takako
Applied Brain Science Laboratory,Engineering, School of Engineering,Tokyo Institute of Technology

Para-foveal and peripheral visual information have different visual resolutions and thus play different roles in daily tasks, such as object recognition. A novel application of the isotropic nature of human central and peripheral vision is gaze-contingent foveated imaging windows that are rendered at high resolution in central vision and low resolution at the periphery.
However, temporal delay between gaze shift and the window is unavoidable. Authors investigated how much temporal delay is allowed for practical application while keeping the best user experience.
Here we performed a simple visual search for Chinese characters with gaze-contingent foveated imaging windows. The relative temporal delay of the window, with a size of a single letter, to the eye movement varied from 0 to 3000 ms. A recorded gaze behaviour condition was used as no control.
The sense of agency (SOA) score for the gaze-contingent display showed a decrease as the temporal delay increased, and the sense of delay score exceeded the SOA score at 250 ms, and then the SOA reached the floor at 500 ms.
The calculated controllability score reached the floor at 3000 ms where the score suddenly approached the lowest score, which was equivalent to the no control condition.
The temporal property of the sense of delay and SOA score in our study was roughly the same when users manually controlled visual object with a joystick and mouse. Our result suggests that information presentation delay should be within 3000 ms to keep the user's SOA and controllability when gaze-contingent display is designed.

Poster 91

Eye-tracking study of processing Russian polycode texts

Petrova Tatiana ; Riekhakaynen Elena
Saint-Petersburg State University

As visual representation of information is getting more and more popular nowadays, studies of so-called “polycode” texts integrating both verbal and non-verbal types of information are of high priority. This study investigates how readers integrate text-figure information when reading multimodal texts. Using the eye-tracking method, we compared the processing of different multimodal and verbal texts. Three experiments were carried out. In Exp.1, native speakers of Russian read infographics (graphic visual representation of information). In Exp.2, we studied the processing of different types of visual notes containing a handwritten text and drawings (‘path’ (trajectory), linear, and radial sketchnotes) by native speakers of Russian. In Exp.3, Chinese students learning Russian as a foreign language examined infographics and verbal texts. We measured the total dwell time, the total fixation count, average fixation duration for each verbal and non-verbal zone of the texts. Text comprehension was controlled by off-line methods (subjective scaling, answers to the after the text questions, key words). We revealed specific features of verbal text and polycode text perception and proposed a number of recommendations for creating effective polycode texts. The overall results of the study show that readers process the information better and faster while reading multimodal text of any format than a verbal text. The reading patterns in polycode text processing are text directed. The influence of the text type becomes crucial only to experienced readers: the better the reading skills are, the greater the influence of the factor “text type” is. Supported by RFBR grant No18-00-00640.

Poster 92

Grammatical gender in German: How do 6-10-year-old children process noun-external gender information in sentences?

Annika Bürsgens[1], Jürgen Cholewa[2], Thomas Günther[1,3]

1 *Child Neuropsychology Section, Department of Child and Adolescent Psychiatry, Psychotherapy and Psychosomatics, Medical Faculty, RWTH Aachen University, Germany*

2 *Institute of Special Education, Heidelberg University of Education, Germany*

3 *Faculty of Health, Zuyd University, Heerlen, the Netherlands*

Research in different languages suggests that gender information influences the decoding of subject and object. However, there has not been any study that addressed the online-processing of sentences in German. Thus, in the current study, we asked 6-10-year-old children to perform an eyetracking-paradigm. Participants were asked to listen to short sentences and to look at the corresponding picture. We varied the position of subject and object (canonical vs. topicalized sentence) as well as the gender of subject and object (equal vs. unequal). Our results revealed that children used gender information to identify the agent in canonical sentences and the patient in topicalized sentences.

Poster 93

Measuring ocular torsion using a low-cost head-mounted eye tracker

Annemiek Barsingerhorn, Demy van de Veen, Jesse Heckman, and John van Opstal
Radboud University, Donders Center for Neuroscience, Dept Biophysics, Nijmegen, The Netherlands

Video-based eye trackers are well-suited to measure horizontal and vertical eye movements. However, measuring torsional eye movements, i.e. rotations of the eye around the line of sight, with video-based eye trackers remains challenging due to the relatively low spatial resolution of video images, limited visibility of iris features under infrared illumination, and distortion of the iris pattern due to the optics of the eye. We assessed the feasibility of measuring torsional eye movements during passive whole-body rotations with the Pupil Labs eye tracker. Participants were placed in a vestibular chair and were passively rotated sinusoidally around the sagittal axis (roll rotation), evoking a torsional nystagmus. We used template matching of the entire iris pattern (Otero-Millan et al., 2015) on the raw video data (120 fps, 400 x 400 pixels) to estimate the amount of torsion. The analysis was performed offline using OpenCV functions in Matlab. Preliminary results demonstrate the feasibility to measure torsional nystagmus with the Pupil Labs eye tracker. We currently aim to measure ocular torsion in participants who actively look around making head-unrestrained gaze shifts. The possibility to measure torsional eye movements based on low-quality video images is promising for clinical applications, as well as for fundamental research on 3D gaze control.

Otero-Millan, J., Roberts, D. C., Lasker, A., Zee, D. S., & Kheradmand, A. (2015). Knowing what the brain is seeing in three dimensions: a novel, noninvasive, sensitive, accurate, and low-noise technique for measuring ocular torsion. *Journal of vision, 15*(14), 11-11.

Poster 94

Oculomotor action effect learning in gaze interaction: The role of effect contingency

Huestegge, Lynn; Riechelmann, Eva
Würzburg University

Efficient gaze control is known to be associated with the anticipation of its effects (ideomotor control), which requires the acquisition of learned associations between actions (i.e., saccades) and their (visual) effects in the environment. While contingency (extent of covariation between action and effect) is known to affect manual ideomotor action-effect learning, corresponding eye-movement studies are lacking. Given that social gaze interaction is a particularly interesting domain to study the anticipation of our eyes' effects on the environment, we here focused on displayed faces that respond to the participant's gaze with direct vs. averted gaze (in addition to a non-social baseline condition). Two experiments focused on the impact of contingency when acquiring action-effect associations. To examine the occurrence of anticipation, both experiments included congruency manipulations to prime or interfere with any anticipated representation of the subsequent effect signal. Importantly, we observed congruency effects (indicating effect anticipation) in both social and non-social contexts across experiments. In Experiment 1, action-effect learning was unaffected by contingency. In Experiment 2, however, where participants were explicitly instructed regarding contingencies, the congruency effect diminished for reduced contingency. This implies that oculomotor action-effect learning is modulated by contingency irrespective of (social vs. non-social) context. This observation is at odds with previous predictions that the impact of reduced contingency might be attenuated in social (vs. non-social) environments due to a generally reduced predictability in social interactions.

Poster 95

Power-Large and Precision-Small: Decoding Affordance Effects During Visual Search by Eye Movement Analysis

Matthias Hartmann
University of Bern / Swiss Distance Learning University

Perception and action are highly interlinked. It has been suggested that perception automatically triggers motor activity in order to prepare the observer to interact with the environment. An example for such an “affordance effect” is that people respond faster to large objects with a power grip response, and to small objects with a precision grip response. This effect has also been extended to visual search, where a reduced search time has been observed when the response grip (power vs. precision) was congruent with the size of the target. The nature of this effect still remains unclear: Does response preparation biases visual search by directing attention preferably to congruent objects, or is the reduced search time the mere consequence of faster response execution? The aim of this study was to disentangle these two mechanisms in a visual search task by means of eye movements analysis: A bias in visual search would be reflected in a difference in search behavior before the target is found (e.g., more fixations on large than on small distractor objects in the power grip conditions). On the other hand, a bias in response execution would be reflected in a difference in the latency between the fixation on the target (i.e., last fixation before response) and the motor response. Preliminary results suggest that affordance effects can be attributed to the response execution level. This study highlights the added value of eye tracking for decoding behavioral effects, and the results will advance the understanding of perception-action interactions.

Poster 96

Does priming affect the nasal-temporal free choice bias?

Ómar I. Jóhannesson, Jérôme Tagu and Árni Kristjánsson
University of Iceland

About 40 years ago Posner and Cohen (1980. Attention and the control of movements. *Tutorials in Motor Behavior*, 243–258.) showed that when two stimuli appear simultaneously to the left and to the right of the centre of a (computer) screen and people view the scene with the view of the one eye blocked, they prefer to saccade into the temporal hemifield (away from the centre of the body). This choice bias was later confirmed by Bompas and Sumner (2008. Naso-temporal asymmetry for signals invisible to the retinotectal pathway. *Journal of Neurophysiology*, 100(1), 412–421). When a property of a target during visual search is repeatedly shown, the time needed to respond to it is reduced, and this priming effect has also been shown to cause a bias in free-choice tasks (Brascamp, J., Blake, R. & Kristjánsson, Á., 2011. *Journal of Experimental Psychology: Human Perception & Performance*, 37, 1700-1707). But no one has tested whether this priming choice bias affects the nasal-temporal choice bias, which is what we do in our study. Odd-one-out visual search trials (2, 4 or 6 with the same target and distractor color in a row) were followed by choice trials. On choice trials, there was one stimulus in the nasal hemifield and another in the temporal hemifield randomly having the color of the primed target or the distractors. Our preliminary results suggest that the nasal temporal choice bias is reduced when it is in conflict with the priming choice bias.

Poster 97

Looking Near and Looking Far: Do humans monitor the exact locations of their actions´ future consequences?

Christina U. Pfeuffer
University of Freiburg, Germany

Our environment is governed by contingencies between our actions and their consequences. These contingencies allow us to learn which action causes which consequence. Crucially, this learning history also imprints on our eye movements and guides our attention based on the expectations we derive from it. Our eyes often already saccade towards the locations we expect the consequences of our actions to appear at later on. The resulting anticipatory saccades are thought to reflect a proactive monitoring process that prepares a later comparison of expected and actual action consequence. Here, we investigated whether participants anticipated the exact locations (near vs. far) their actions´ future consequences would appear at and proactively saccaded towards these locations or whether participants only anticipated their actions´ consequences to appear in a certain rough direction. In line with prior findings, participants looked significantly more often in the direction of their actions´ future consequences than in another direction. However, contrary to our assumptions, the length of participants´ manual responses (short vs. long movement) rather than the exact location of the respective action consequence had a strong influence on the amplitude of participants´ anticipatory saccades towards their actions´ future consequences.

Poster 98

Eye-gaze Behaviors in Natural Human-Human and Human-Robot Interactions

Tipporn Laohakangvalvit, Kristiina Jokinen
National Institute of Advanced Industrial Science and Technology, Tokyo, Japan
tipporn.laohakangvalvit@aist.go.jp, kristiina.jokinen@aist.go.jp

In human-human (HH) interaction, gaze plays an important role during such coordination as turn-taking in conversation. Mutual gaze is used to agree on the change of speaker (Kendon, 1967; Jokinen, 2012). Similarly, the use of gaze has been considered having a positive effective in human-robot (HR) interaction (Yu et al., 2012). Therefore, our study aims to explore eye-gaze behaviors in natural HH dialogues in order to develop HR applications that enable intuitive interaction with humans in daily life.
We asked participants to have free conversation with a human partner and a humanoid robot while their eye movements were being recorded using eye-tracking glasses. We analyzed experimental results by defining three AOIs (i.e. face, body, and other areas) and employing four eye movement indexes (i.e. number of fixations, fixation duration, number of first fixations, and number of last fixations) which indicate attentional focus of the participants. The results of all indexes were consistent that the participants tended to look at "face" area in HH interaction whereas "body" area in HR interaction. Our results supported the fact that that humans seem to monitor partner's reactions expressed through face and eyes, which provide necessary information for HH interaction. However, in HR interaction, the participants focused more on the robot's body rather than on its face possibly because the face did not provide "live" information about the robot's internal state like its emotion or intention. These results provide us a clue for further detailed study and development of robots that intuitively interact with humans.

Poster 99

How brightness affects pupil size and vergence in a VR setting

Anke Huckauf, Christoph Grabmaier, Sina Reininghaus*, Thiess Pfeiffer*

*Ulm University, Germany, * University of Bielefeld, Germany*

Video-based eye tracking is known to be prone to brightness changes. In fact, it had been shown that changing the brightness of the stimuli alters pupil dilation and leads to a change in the estimated fixation location. This is usually attributed to asymmetric movements of the pupil during dilation. We investigated this assumption in a stereoscopic virtual environment presenting bright or dark arrows at distances of 30cm, 60cm, and 120cm. A stimulus of medium brightness served as calibration baseline for vergence and for pupil diameter. Fourteen observers were tracked with an SMI RED tracker within a virtual reality headset. The brightness variations resulted in mean pupil sizes between bright and dark stimuli which differed about 2mm. Observed disparities fitted to the expected values. However and contrary to the hitherto reported findings, there were no changes in estimated fixation positions for dark versus bright stimuli. Rather, the estimated binocular gaze positions did not differ. Various assumptions regarding simultaneous changes in accommodation, pupil size, and binocular viewing will be discussed.

Poster 100

Gaze based Measurement of Cognitive Load from Multi-tasking with Human-Robot Interaction

Lucas Paletta, Martin Pszeida
JOANNEUM RESEARCH Forschungsgesellschaft mbH, Austria
lucas.paletta@joanneum.at

Dynamic coordination of attention refers to executive functions for goal oriented and flexible behaviors (Diamond, 2013). Stress can increase attention selectivity within a single task (Chajut & Algom, 2003) but decreases weight on complex cognitive processes (Hermans et al., 2014) whenever multi-task requirements surpass the regulatory capacity (Koolhaas et al., 2011).
This work investigated human-robot interaction where executive functions are fundamental for decision making (Bütepage & Kragic, 2017) and impact of stress on attention is relevant for safety, quality and performance. We measured the precision of selective attention for eye-hand coordination as part of a multi-tasking scenario under varying stress, including (i) a cognitively challenging N-back test (Jaeggi et al., 2010) to trigger a peg-in-hole task and (ii) a collision avoidance task related to a random walk of a robotic arm within the operation area. While pupil dilation scales with task difficulty in many cognitive domains (Klingner et al., 2011) we specifically focus on the impact on gaze and multi-tasking performance. We measured the precision of probing looks towards the goal position of eye-hand coordination (Flanagan & Johansson, 2003) with 6 participants and observed the dependence of cognitive arousal measured by electro-dermal activity (EDA) as well as variance in the gaze on the eye-hand goal position on the degree of task difficulty of the N-back test.
The results demonstrate that the gaze positioning error is positively correlated (r=.547) with task difficulty and indicate the measurement of executive function performance under cognitive arousal from eye movement features.

Poster 101

Eye-tracking in VR setting: implementation for cross-cultural research

Vojtech Jurik, Pavel Ugwitz Jiri Chmelik, Zuzana Smidekova, Cenek Sasinka
(Department of Psychology, Faculty of Arts, Masaryk University, Brno, Czech Republic; Division of Information and Library Studies, Faculty of Arts, Masaryk University, Brno, Czech Republic)
(Department of Geography, Faculty of Science, Masaryk University, Brno, Czech Republic; Division of Information and Library Studies, Faculty of Arts, Masaryk University, Brno, Czech Republic)
(Department of Visual Computing - Faculty of Informatics, Masaryk University, Brno, Czech Republic)
(Department of Educational Sciences, Faculty of Arts, Masaryk University, Brno, Czech Republic; HUME lab, Faculty of Arts, Masaryk University, Brno, Czech Republic)
(Division of Information and Library Studies, Faculty of Arts, Masaryk University, Brno, Czech Republic; HUME lab, Faculty of Arts, Masaryk University, Brno, Czech Republic)

Eye movement tracking represents royal pathway to understand human sensorimotor activity, especially with respect to specific environments. Human sensorimotor activity is an important factor by which we can infer cognitive processes, describe and predict human performance and also measure and compare individual differences among people. The most significant indicator of human sensorimotor activity is an analysis of one's areas of visual interest in his/her surroundings (Holmqvist, 2011). Regarding this, next to the real world settings, eye tracking finds huge potential in the context of virtual reality (VR). VR capabilities providing environmentally valid reality-approaching dynamic stimuli while maintaining high level of experimental control allow researchers to collect complex and accurate data about human behavioral and decision-making processes (Loomis et al., 1999). Virtual interfaces equipped with the functionality to track and record data about areas of human visual interest and virtual locomotion provide comprehensive evidence about users behaviors and may be applied in many research areas - e.g. navigation, perception, visual cognition (Stachoň et al., 2018) or cross-cultural research (Miellet et al., 2012). In the presented contribution we describe specific head-mounted-display-based experimental interface equipped with the ET technology, which we have designed for the research of information search activity and spatial navigation activity in immersive virtual environments. We discuss HW, SW and methodological implications, possibilities and limits of this ET implementation for VR and finally we propose its use for cross-cultural research of human perception and cognitive processes.

References:

Holmqvist, K. (2011). *Eye tracking: a comprehensive guide to methods and measures*. New York: Oxford University Press.
Loomis, J. M., Blascovich, J. J., & Beall, A. C. (1999). Immersive virtual environment technology as a basic research tool in psychology. *Behavior research methods, instruments, & computers, 31*(4), 557-564.
Miellet, S., He, L., Zhou, X., Lao, J. & Caldara, R. (2012). When East meets West: Gaze-contingent Blindspots abolish cultural diversity in eye movements for faces. *Journal of Eye Movement Research, 5* (2), 1-12.
Stachoň, Z., Šašinka, Č., Čeněk, J., Štěrba, Z., Angsuesser, S., Fabrikant, S. I., et al. (2018a). Cross- cultural differences in figure–ground perception of cartographic stimuli [Online]. Cartography And Geographic *Information Science, 46*(1), 82-94.

Poster 102

Transposed-Word Effects in Grammaticality Judgements: An Eye-Tracking Study in a Virtual Reality Environment

Jonathan Mirault, Agnès Guerre-Genton, Stéphane Dufau & Jonathan Grainger
Aix-Marseille University & Centre National de la Recherche Scientifique, Laboratoire de Psychologie Cognitive, Marseille, France.

We replicated the novel transposed-word effect in grammatically judgements described by Mirault et al. (2018), adding eye-tracking measures recorded in a virtual reality environment. We displayed five-word sequences that could be grammatically correct or not, and with two types of ungrammatical sequence: i) the Transposed-word condition where the ungrammaticality was created by transposing two adjacent words in a grammatically correct sentence (e.g., “The white was cat big”) and ii) the Control condition, matched to the transposed-word condition, but which could not generate a correct sentence by transposing two adjacent words (e.g., “The black ran dog fat”). The same number of grammatically correct sentences (e.g., “The little brown rabbit”) were intermixed with the ungrammatical sequences for the purposes of the grammaticality judgment task. We tested 40 participants, and the analysis of grammaticality judgments revealed a significantly higher error rate and longer response times in the Transposed-word condition compared to the Control condition. The eye-tracking measures further revealed more fixations (refixations and regressions) and longer reading times in the Transposed-word condition compared to the Control condition. We conclude, in accordance with Mirault et al. (2018), that the process of encoding word order information during sentence reading is noisy. Over and above the replication and extension of the Mirault et al. study, we demonstrate the possibility of using eye-tracking measures in a virtual reality environment for investigating reading.

Poster 103

Visual exploration serves to recapitulate details from autobiographical memory

Jennifer D. Ryan[1,2], **Michael J. Armson**[1,2], **Brian Levine**[1,2]

1 Rotman Research Institute, Baycrest

2 Department of Psychology, University of Toronto

Eye movements contribute to the encoding of memories by extracting information from the visual world (Yarbus, 1967). Research using “looking at nothing” paradigms has suggested that eye movements may also be functional for the retrieval of memories by aiding in the reinstatement of details (cf. Wynn, Olsen, Binns, Buchsbaum & Ryan, 2018). In this study, participants were guided through an art tour, which was divided into two parts, in order to create a staged event for which memory would be subsequently tested. A week later, participants were asked to recount their personal recollections of their art tour experience (autobiographical memory; Palombo, Sheldon & Levine, 2018) while their eye movements to a blank screen were recorded. Participants were asked to recall one part of the art tour under free viewing instructions, and were asked to recall the other part of the art tour while restricting fixation to a central location. When viewers were asked to maintain fixation, they had fewer gaze fixations and smaller saccade amplitudes compared to when they were engaged in free viewing. Regardless of viewing condition, participants who self-rated as high on autobiographical memory retrieval showed a relationship between the amount of visual exploration and the amount of details that were produced that referred specifically to the event itself (i.e., internal details). These findings suggest that visual exploration may be important for recapitulating details from memory, particularly for people who, subjectively, have rich re-experiences of prior events.

References:

Palombo, D.J., Sheldon, S. & Levine, B. (2018). Individual differences in autobiographical memory. *Trends in Cognitive Sciences, 22*(7), 583-597.

Wynn, J.S., Olsen, R.K., Binns, M.A., Buchsbaum, B.R. & Ryan, J.D. (2018). Fixation reinstatement supports visuospatial memory in older adults: an eye movement compensation effect. *Journal of Experimental Psychology: Human Perception and Performance, 44*(7), 1119-1127.

Yarbus, A.L. (1967). Eye Movements and Vision. New York: Plenum Press.

Poster 104

The interplay of eye movements and long-term memory: Using a novel combination of eye tracking and complex span tasks to reveal how eye movements draw on long-term memory

Lucas Lörch
University of Mannheim

Studies comparing experts and novices have shown that eye movements during domain-specific visual processing, such as the reading of notated music, are supported by long-term memory (LTM). Accordingly, if long-term memory information that is irrelevant for the visual processing task is activated, LTM support of eye movements is impeded. As a result, the amount of information that can be perceived with a single fixation decreases and the number of fixations increases given limited reading time.
I employed a novel combination of the complex span paradigm and eye tracking to test this assumption. Music students (n=75) were asked to memorize one note and then to play an unknown, simple melody on a piano. After twelve repetitions of this procedure, they were asked to recall the memorized notes in correct order. It was varied within participants if successive memory notes formed chords or did not form chords. Eye movements during the musical performance of the melodies were tracked and memory performance was measured with a recall test.
When memory notes formed chords, recall accuracy was higher and eye movements showed more fixations. Moreover, the number of fixations increased with each additional note that had to be held in memory. Both the more efficient storage of information when memory notes formed chords and the encoding of additional notes in the course of one trial led to more task-irrelevant activation in LTM. This, in turn, hindered LTM support for the eye movements and led to reading with more fixations.

Poster 105

Contextual Cueing Effect following Display Rotation: Evidence from Eye Tracking

Shiyi Li, Xuejun Bai, *Tianjin Normal University, China;*
Hong-Jin Sun, *McMaster University, Canada*
elevenny@hotmail.com

In classic contextual cueing paradigm, participants perform a visual search task in which they look for a target letter T among a random array of distractor letter Ls. Half of the scenes were repeated once over blocks while the other half were novel. Over time, RT for the repeated scenes became faster than that for the novel scenes, suggesting that participants learned the relation between repeated layout and target location. The present study was aimed to examine whether contextual cueing effect remained after the displays were rotated following learning. In the training phase, participants experienced 30 blocks of search trials in which the display monitor was rotated 22.5 degrees clockwise (or counter-clockwise) around the z-axis (in the frontal-parallel plane) from the normal landscape orientation. Participants then experienced 5 testing blocks in which the monitor was rotated 22.5 degrees counter-clockwise (or clockwise), thus creating a 45-degree display rotation from the learning to the testing phase. Significant contextual cueing was found in the training phase, with faster RTs in the repeated scenes than that in the novel scenes. The contextual cueing effect in the testing phase was comparable to that at the end of the training phase. By tracking eye movements during the search, we found fewer numbers of fixation, smaller scan pattern ratio for repeated scenes in both (late) training and testing phase. The results showed that the contextual cueing effect can be maintained following scene rotation, suggesting that the benefit of implicit learning of a spatial relation could be flexible.

Poster 106

The signature of semantic interference on eye-movement in a long-term visual memory task

[a]**Anastasiia Mikhailova** *(a.mikhailova@psicologia.ulisboa.pt)*
[a]**Ana Raposo** *(alraposo@psicologia.ulisboa.pt)*
[b]**Sergio Della Sala** *(sergio@ed.ac.uk)*
[a,c]**Moreno I. Coco** *(moreno.cocoi@gmail.com)*
a Faculdade de Psicologia, Universidade de Lisboa, Alameda da Universidade
b Human Cognitive Neuroscience, Psychology, University of Edinburgh
c School of Psychology, University of East London

The capacity of long-term visual memory is known to be susceptible to semantic interference (SI): the more scenes belonging to the same semantic category are viewed, the harder it is to recognize individual scenes. However, it is yet to be understood whether the allocation of overt attention is also mediated by SI, and how this reflects in recognition memory. In the current eye-tracking study, we asked participants to watch a stream of 372 photographs, where each photo belonged to a semantic category (e.g., a kitchen) in different concentrations (4, 20, 40, 60). Participants were then presented with the same 372 photographs plus 372 unseen photographs (in randomized order and equal category concentrations) and asked whether they recognized (or not) each photo (i.e., old/new recognition paradigm). We found that SI had an effect on recognition accuracy replicating previous literature, but only in new images, whereas the performance on old images was at chance level. When looking at the fixation duration and the entropy of fixation distribution across the scene, we found that successfully recognized scenes had shorter fixation duration and higher entropy. Moreover, the effect of SI on eye movements was only present during encoding. In particular, even though participants were at chance in old images, fixation duration increased and entropy decreased as a function of SI. These results suggest that SI modulates overt attention during encoding, even when recognition performance is at chance level. More broadly, our study highlights the interdependence between mechanisms of attention and memory.

Poster 107

Dynamic memory resource allocation to new saccade targets

Rob Udale, Masud Husain
School of Experimental Psychology, University of Oxford
Robert.udale@psy.ox.ac.uk

Encoding a memory representation of the environment requires integrating information from multiple saccades. How are memory resources re-distributed to the incoming perceptual information over these saccades? Previous research has indicated that items which are currently being saccaded towards, but not yet fixated, are more precisely represented in memory than previously fixated items. This is consistent with the view that saccades are preceded by a shift in covert attention to the saccade target location and reallocation of memory resources to the saccade target's features. We tested the effect of fixation order on memory recall precision. Participants sequentially fixated and remembered items in a memoranda display (locations or orientations) at their own pace. Once they initiated a saccade towards the final item, the display disappeared. After a brief interval, they were cued to recall the feature of one of the stimuli. We found greater precision f or the final saccade target than for previously fixat ed items. This occurred irrespective of whether they were instructed to saccade towards a particular item last, or whether to fixate the items in any order they chose. Finally, we ran an experiment in which the display would offset before they had viewed all of the items. In this case, the final item benefit disappeared, suggesting that it is dependant on knowing which item would be the final item in the saccade sequence. These results indicate that resources are flexibly and strategically allocated to the upcoming saccade target. However, in less predictable environments, observers only encode features during fixation.

FACE PERCEPTION & EMOTION

Poster 108

Musical expertise in a sight-reading task: an eye-tracking study

Maria Timoshenko*[1], Marcus Nyström[illegible][2], Halszka Jarodzka[illegible][3]
**Faculty of Education and Welfare Studies, Åbo Akademi University, Vasa, Finland*
[illegible] Lund University Humanities Lab Lund, Sweden
[illegible]Faculty of Educational Science, Open University of the Netherlands
[1]maria.timoshenko@abo.fi, [2] marcus.nystrom@humlab.lu.se, [3] halszka.jarodzka@ou.nl

The process of reading music has more obstacles than reading words at sight. Sight reading prima vista as a skill of performing unknown music score requires many years of deliberate training. In order to characterize expertise in singing prima vista task, the study investigated the performance and the eye-voice span (EVS) of musicians with different levels of expertise.

Eye movements and audio of 20 musicians of two levels of expertise (professional and semi-professional) were recorded while they sang 10 trials consisting of notes and lyrics in temporal restricted condition (metronome rate 60bpm). The software Melodyne-4 (Celemony 2016, www.celemony.com) quantified the accuracy of singing pitch. Expertise levels and complexity of the musical scores were used as independent variables. The eye-voice span and the accuracy of the singing performance were selected as dependent variables.

The results indicate that professionals performed significantly better than semi-professionals, where the former group made fewer errors in both musical notes and lyrics. The EVS did not correlate with the level of expertise. Moreover, when looking at different parts of the score, the beginning of a semi-phrases was associated with a larger EVS. In addition, no correlation was found between EVS and performance.

To conclude, the prima vista singing improves even on higher levels of expertise. However, our findings also show that EVS does not seem to be a predictor of this performance. Future research should investigate what underlies this performance difference on higher expertise levels and whether EVS play a role in performance on lower expertise levels.

Keywords: sight-reading, expertise, eye movement, signing prima visa, visual perception, sight-singing strategies, music

Reference:

Drai-Zerbib, V., Baccino, T. & Bigand, E. (2011). Sigh-reading expertise: Cross-modality integration investigated using eye tracking. *Journal Psychologi of Music 40*(2), p.216-235.

Holmqvist, K., Nyström, M., Andersson, R., Dewhurst, R., Jarodzka, H., & Van de Weijer, J. (2011). *Eye tracking: A comprehensive guide to methods and measures.* Oxford, UK: Oxford University Press.

Huovinen, E., Ylitalo, A., & Puurtinen, M. (2018). Early attraction in temporally controlled sight-reading of music. *Journal of Eye Movement Research, 11*(2).

Rayner, K. & Pollatsek, A. (1997). Eye Movement, the Eye-Hand Span, and the Perceptual Span During Sight-Reading of Music. *Journal The American Psychological Society.*

Truitt, F. E., Clifton, C., Pollatsek, A., & Rayner, K. (1997). The perceptual span and the eye-hand span in sight-reading music. *Visual Cognition, 4*(2), 143–161.

Poster 109

Saccadic reaction times in neurodevelopmental disorders

Klein, C., Canu, D., Ioannou, C., Fleischhaker, C., Biscaldi, N.
University of Freiburg

Background: The voluntary control of eye movements has appeared to be dysfunctional in psychiatric conditions of childhood and adolescence that share deficits in executive functions.
Methods: We tested 4 groups of adolescents aged 14-23 years old – Early-Onset Schizophrenia (SZ), ADHD, Autism, typically developing participants (TD), matched in age and full-scale IQ – on prosaccade (PRO), antisaccade (ANT) and memory-guided saccade (MEM) tasks while eye movements were recorded binocularly (Eye Link 1000 Plus).
Preliminary results: While SZ showed normal saccadic RTs in PRO and MEM tasks, they were slower in generating correct antisaccades and had a higher frequency of direction errors in ANT, but not in PRO and MEM, as compared to TD. Additionally, SZ showed a higher frequency of anticipatory saccades in all tasks. Finally, patients showed increased intra-subject variability (ISV) than controls in all tasks, although for both PRO and ANT significantly so only in GAP, but not overlap, conditions.
Discussion and Conclusions: Results confirm intact basic sensory-motor and memory-guided saccade control but impaired executive saccade control in patients with SZ. The anti-saccade task with its pronounced “prefrontal” load proofed particularly sensitive to the SZ condition. In contrast to delayed mean latencies of saccadic RTs, increased anticipations and ISV were task-nonspecific and thus potentially pointing to a global underlying deficit. Furthermore, normal mean RTs alongside increased intra-subject standard deviation of RTs show that increased ISV may not be a consequence of response slowing.

Poster 110

The sense of speed when watching video clips: Behavioural, pupillary, oculomotor and cortical responses.

Regina Gregori-Grgič, Claudio de'Sperati

Laboratory of Action, Perception and Cognition, Università Vita-Salute San Raffaele; and Unit of Experimental Psychology, San Raffaele Neuroscience Division

We have previously found a poor sensitivity to speed manipulations of video clips, and large speed biases [1-3]. None of 100 naïve observers spontaneously noticed speed alterations up/down to 12% when viewing a soccer match. When measured with a constant-stimuli discrimination task, speed sensitivity was still low (JND=9%). Also, we found a tendency to speed underestimation, with PSE errors up to [illegible]30%, depending on video content. Speed underestimation was larger in 6-years old than 10-years old kids, and was robust to display size and soundtrack manipulations. PSE was uncorrelated with estimated duration of the video clips, thus pointing to a distinct sense of event speed. To initially characterize the cortical and autonomic correlates of the sense of speed, we measured steady-state visual evoked potentials (SSVEP) and pupillary oscillations (PO) to sinusoidal speed modulations (10-50%, 1-4 Hz) of random-dot kinematograms (RDK) and naturalistic visual motion stimuli. We found clear SSVEPs but no comparable POs. Being evoked also by non-directional motion patterns (RDK with 0% coherence), these oscillatory cortical responses qualify as a new type of speed-driven SSVEPs, which were observed in all cortical regions. Also, we measured pupillary, oculomotor and cortical responses when watching videos showing repetitive human or mechanical motion reproduced at various speeds. We argue that global resonance mechanisms contribute to the sense of speed.

[1] **de'Sperati & Baud-Bovy** (2017). *Sci Rep 7*, 15379.
[2] **Rossi et al** (2018). *Front Int Neurosci 12*, 11.
[3] **Zuliani et al** (2019). *J Exp Child Psychol 179*, 190.

Poster 111

Surveilling the scene: Learning about the world from multiple 2D dynamic views

Sara Spotorno[1], Kenneth C. Scott-Brown[2], Sophie Shenton[3], Matthew J. Stainer[4], & Benjamin W. Tatler [3]

[1]Keele University, UK, [2]Abertay University, UK, [3]University of Aberdeen, UK, [4]Griffith University, AU

CCTV surveillance is an ideal model to understand the nature and time course of spatial learning when viewing the world indirectly through multiple simultaneous videos, as increasingly happens in our everyday life. We reproduced a six-scene CCTV multiplex display on a computer screen showing videos of different rooms of a public building. Participants tracked an actor walking across the rooms. We used eye movements as an index of spatial learning. After a training phase, with the same scene content and display arrangement repeated across a series of trials, we interrupted learning by changing the display arrangement (Expt. 1) and the viewpoint of each room (Expt. 2) or the walked path (Expt. 3), which then remained constant in the trials of test phase. We analysed the number of gaze transitions between scenes, comparing the real data in the test phase to what would be predicted from the training phase if no interruption had occurred. The interruption caused a significant learning disruption in each experiment, increasing the number of gaze transitions. However, this effect was short lasting (1-3 trials). The most disruptive manipulation was the path change, which tests understanding of the relationships between the scenes. Overall, our findings suggest that learning was not limited to the display arrangement or the content of each room, but extended to the layout of the whole 3D environment. Indeed, it included representation of how the rooms are connected to each other in the real space, even though this aspect appeared relatively harder to learn.

Poster 112

Narrative aesthetic absorption into audiobooks: Acoustics, cross-modal coupling and subjective states are related

Lange, E.B., Thiele, D., Fink, L. & Kuijpers, M.

Max Planck Institute for Empirical Aesthetics; Max Planck Institute for Empirical Aesthetics; UC Davis; University of Basel

We investigated narrative aesthetic absorption in the context of audiobook listening. Our study covers three aspects: (1) Acoustic features are used to structure speech and to communicate emotions. The role of acoustics for audiobook absorption has not been investigated so far. We extracted acoustic features of the audio files and explored how they related to absorption and eye parameters (microsaccade rate, blink rate, pupil dilation). Given the evidence on music listening, we investigated (2) cross-modal coupling between eye parameters and subjective states during listening to audiobooks, and (3) relations between acoustics and eye parameters. For our study, we selected excerpts of a wide range of authors (e.g., Tolkien, Goethe, Follett), literary genres (e.g., novels, fairy tales, mystery stories, drama, poetry, dada), and text-types (dialogue versus descriptions), with a length of 37 to 60 sec. After audio presentation, participants rated their subjective states. Acoustic analysis revealed strong dependencies between features. Excerpts were best described by pitch range, intensity, articulation rate, noise-to-harmonics ratio (nhr) and pitch variability. We related these features to absorption as well as to the eye parameters. State absorption was predicted by reduced blink rate, higher articulation rate, and individual differences in trait absorption. In addition, higher articulation rate predicted lower mean pupil dilation, and higher nhr was related to higher microsaccade rate. We show a complex pattern of relations between acoustics, cross-modal coupling and subjective states. Eye-tracking and audio analysis yield important predictions regarding auditory absorption.

Poster 113

Developing eye-tracking measures of self-regulation in infancy

Martina Zaharieva[1,2], Cristina Colonnesi[2], Roos Rodenburg[2], Ingmar Visser[1]

1 Research Institute of Developmental Psychology, University of Amsterdam, Netherlands

2 Research Institute of Child Development and Education, University of Amsterdam, Netherlands

During the first year of life, infants equip themselves with a number of attentional control mechanisms allowing them to become increasingly more proactive in selecting what information to process, thereby building a necessary foundation for complex self-regulatory capacities such as executive functions. Individual differences are likely to hold predictive value for later-life adjustment outcomes, yet the majority of early-life research describes group-average attentional capacities. We thus set out to develop novel eye-tracking measures to capture individual differences in the emergent attentional control. Crucially, tonic and phasic changes in arousal unfold dynamically with attentional states in early infancy; accounting for continuous changes in arousal can thus help us interpret eye-movement patterns believed to be attention-driven. In two eye-tracking tasks, we relate how 60 4-month-old infants differ in the speed of recovering attentional focus in a gaze-contingent sustained attention task (adapted from Wass, Porayska-Pomsta, & Johnson, 2011) to the speed of endogenous attentional shifts in the Gap-Overlap task. Eye-movement patterns from these tasks are then related to changes in pupil diameter and head-movement velocity measured concurrently. Using multilevel time-series modelling, we expect that infants faster at restoring attentional focus are also faster at shifting attention, while exhibiting attentional episodes more strongly correlated to corresponding arousal changes. The attentional measures will be validated with micro-coded measures of child-caregiver joint-interaction and caregivers' reports on the infant's level of self-regulation. This work will improve our understanding on whether early-life differences in online attentional control translate into differences at the behavioral scale of self-regulation.

Keywords: attention control, pupil dilation, head movement velocity, infant eye-tracking, self-regulation

References:

Wass, S., Porayska-Pomsta, K., & Johnson, M. H. (2011). Training attentional control in infancy. *Current biology, 21*(18), 1543-1547.

Poster 114

Oculomotor dominance among multitasking paradigms – the role of temporal overlap in inducing effector system prioritization

Mareike A. Hoffmann, Iring Koch, Lynn Huestegge

University of Würzburg; RWTH Aachen University; University of Würzburg

In multitasking situations performance is usually worse than in isolated single tasks. These performance decrements are often distributed asymmetrically among tasks, a phenomenon that, under suitable conditions, can be interpreted in terms of task prioritization. Among different multitasking paradigms, prioritization effects can be observed in different empirical markers, e.g., in asymmetrical dual-task costs (dual- minus single task performance) in paradigms involving simultaneous stimulus onset, or in PRP effects in the psychological refractory period paradigm. In the task switching paradigm – requiring subjects to switch between tasks on a trial-by-trial basis – performance typically declines in task switch (vs. repetition) trials. Again, these switch costs can be distributed asymmetrically among tasks. Since task switching does not involve temporally overlapping task demands, performance decrements here are merely based on cognitive representations of two competing task sets. Here, we present data demonstrating cognitive prioritization of oculomotor tasks over tasks involving other effector systems (pedal, vocal, manual) in a multitasking paradigm involving temporal task overlap (indexed by smaller oculomotor dual-task costs). In contrast, we did not find consistent evidence for oculomotor prioritization in the task switching paradigm, which does not involve temporal task overlap. The present results demonstrate the important role of temporally overlapping action demands in effector-based prioritization.

Poster 115

Inhibition of FEF increases errors in task-switching and anti-saccades

Gorina E, Ardasheva L, Feurra M, MacInnes J

National Research University Higher School of Economics; National Research University Higher School of Economics; National Research University Higher School of Economics; National Research University Higher School of Economics

Executive functions (EF) represent a set of cognitive processes involved in the control of information processing and goal-directed behavior. Different EFs are often task-specific and relate to different brain networks. For example, ”task switching” involves prefrontal regions while the “antisaccade task” occupies frontal eye field (FEF) and supplementary motor area. Our main goal was to test whether inhibition of the FEF significantly impacts the performance of an “antisaccadic task” while leaving unaltered the “task switching.”

Thus, we ran a study with 15 healthy adults involved in three experimental sessions spaced by 3-to-5 days apart. Subjects received three different stimulation conditions accordingly to the assigned day: Transcranial Magnetic Stimulation (TMS) of the FEF, Vertex (control site), or a no-TMS condition, each followed by two single-task (trials of either prosaccades or antisaccades) blocks and one mixed-task (trials of prosaccades and antisaccades randomly intermixed) block. As a TMS inhibitory protocol, we used a 40-second train of continuous theta-burst, which allows suppression of a target cortical brain area for about 30 to 40 minutes. For the behavioral indexes, reaction times, errors and saccadic reaction times were recorded using the Eyelink-1000 eye-tracker.

Contrary to our predictions, suppression of the right FEF had an impact only on the mixed-task: responses were faster but less accurate in the mixed blocks (speed-accuracy trade-off), while slower and less accurate in both single-task blocks. The accuracy cost of FEF stimulation was also higher in the mixed as opposed to the single-task blocks.

Poster 116

Cognitive control of saccadic eye movements. ERP investigation

Slavutskaya Maria[1,2], Karelin Stanislav[1]

Lomonosov Moscow State University, Russia 1; National Mental Health Research Centre, Moscow, Russia2

Saccadic eye-movements are an adequate model for studying the brain mechanisms of cognitive control. The «double step», «Go/no go» and «Go/no go delay» psychophysiology schemes were used in 59 healthy subjects. These paradigms include the different aspects of saccadic programming and inhibition, and allow us to reveal its correlation with attention and decision-making functions. The error numbers, saccadic latency, parameters and topography of averaged ERP components from 24 EEG points were studied. Analysis of parameters and topography of the P100, N150, P200, N250 and P300 to the signal stimulus has revealed the markers of stimulus evaluation, decision-making, attention shifting and inhibition stages. Different functional meaning of the same ERP components to the «Go» stimuli are depended on the individual saccade latency.

The obtaining data show that neurophysiological mechanisms of saccade programming and inhibition were reflected in the pattern of fronto-parietal neuronal networks and level of their activation with predominance of the frontal areas being leading in the decision-making and inhibition processes. It was shown that inhibition processes are involved into the different stages of stimulus analysis and they interconnected with attention processes, which are reflected in the ERP component foci predominantly localization in the right hemisphere areas regardless of the saccadic direction.

The study allows us to suppose that inhibition is an independent form of the cognitive control, and there are various types of inhibition, depending on the stage and form of adaptive behavior.

Poster 117

Designer's expert eye: Freshman and Senior Students comparison

Magalhães, Cláudio Freitas de; Welch, Leslie; Rodrigues, Erica dos Santos; Forster, Renê; Roque, Monique; Dantas, Sibelius Clausen

Pontifical Catholic University of Rio de Janeiro (PUC-Rio); Brown University; Pontifical Catholic University of Rio de Janeiro (PUC-Rio); Rio de Janeiro State University (UERJ); Pontifical Catholic University of Rio de Janeiro (PUC-Rio); Pontifical Catholic University of Rio de Janeiro (PUC-Rio)

Experiencing physical and digital configurations of objects is an essential part of Design education. This exploratory work investigates whether designers develop a particular way of seeing through training. In particular, it examines the extent to which college education gives designers a specific way of seeing a three-dimensional object. This study compares freshman and senior students' patterns of free visual exploration while looking at a rectilinear volume composition, designed according to visual relations proposed by Rowena Kostellow (Hannah, 2002). Students observed this object through eight different angles while they had their eyes. To measure the number and duration of visual fixations, the study considered four areas of interest: the external area (i.e., the empty spaces around the volume), the dominant form area, the subdominant form area, and the subordinate form area (i.e., the three forms joined in the rectilinear volume). Observations rendered significant different data, particularly when analyzing the external area through different angles (e.g., freshman students tend to look longer and more frequently for the empty space). Heat maps of visual inspection have shown a clear dispersion pattern, between freshman and senior students. Considering object front view inspection, the number and duration of fixations varied according to view angles, where subordinate form area presented higher values than the subdominant one. This work's results pave the way to the hypotheses in which the influence of expertise is decisive to eye movements (Gegenfurtner et al., 2011) and in which different form qualities do have decisive impact on perception.

Poster 118

Frequency Effects during Reading in Very Skilled Readers

Charlotte Lee, Hazel Blythe & Denis Drieghe

School of Psychology, University of Southampton

Eye movements during reading studies have focused primarily on either skilled readers, beginning readers or readers with reading difficulties. Comparatively little attention has been focused on the reading behaviour of very skilled readers. Both skilled and very skilled readers will be identified by traditional means, for example the WIAT-II UK reading subtests (Weschler, 2005) and the Nelson Denny reading and vocabulary tests (Brown, Fishco, & Hanna, 1993). We will examine eye tracking measures of word frequency effects during reading in these readers as a function of reading skill. All readers are expected to show high levels of word skipping and short fixation times for high frequency words compared to low frequency words, with very skilled readers also expected to show increased levels of word skipping and comparatively short fixation times for low frequency words compared to average skilled readers. This prediction is based on the observation that readers with a higher vocabulary display smaller frequency effects with differences mostly expressed in comparatively shorter fixation times for low frequency words (Cop, Keuleers, Drieghe & Duyck, 2015). Additionally, we will administer a test battery of reading and cognitive tests, including a lexical recognition task, the RAN letters and digits, a digit span test, spelling tests (see Andrews & Hersch, 2010), and the Author Recognition Task (Acheson, Wells & McDonald, 2008). The results of this test battery will be used to determine which tests provide measures which are sensitive enough to differentiate between skilled and very skilled readers.

Poster 119

Detecting pre-learnt target triplets in arrays under different word boundary demarcation presentations.

Mengsi Wang[1], Hazel I. Blythe[2], and Simon P. Liversedge[1]
1. University of Central Lancashire, UK
2. University of Southampton, UK

In two experiments, we investigated how participants learnt alphabetic pseudowords with different exposure frequencies in a learning session, and how exposure frequency affected eye movements during target detection in subsequent scanning of longer pseudoword strings. Strings were presented with different triplet boundary demarcation presentations (spaced strings, shadedstrings and unspacedstrings). In Experiment 1, we tested native English speakers and in Experiment 2 native Chinese speakers. Written English is spaced and alphabetic, whereas Chinese is unspaced and logographic. Thus, we wished to examine whether the native writing system modulated eye movement control during learning and scanning of alphabetic pseudowords.
We found consistent exposure frequency effects for English and Chinese participants during learning. Detection performance during string scanning was good, however, exposure frequency learning effects did not carry over. Interestingly, different demarcation effects occurred for fixation durations in Chinese and English readers. English participants' times on target triplets were longest in unspaced strings, shorter for shaded and shortest for spaced strings. By contrast, for Chinese participants' reading times did not differ between shaded and spaced strings. For initial landing positions, patterns were similar in both groups; most fixations landed towards the centre of a target in the spaced condition, left of centre in the shaded condition, and substantially towards word beginnings in unspaced conditions. These data demonstrate that word boundary demarcation through shading more effectively facilitated word identification in Chinese than in English readers, possibly due to their increased familiarity with an unspaced text format.

Poster 120

Inversion effect does not correlate with eye movement’s strategies in facial expression recognition

E. Luniakova, J. Gani-zada

Lomonosov Moscow State University

Face perception is mostly holistic process. It’s proved by inversion effect: inversion of a face impairs the recognition of its identity and facial expression (Yin, 1969; McKelvie, 1995). Holistic and feature-based face processing are associated with specific strategies of eye movements (Caldara et al., 2010). Long inspection of a single feature (mainly nose) and rare fixation shifts to external facial features could be related to configural processing. Numerous fixations distributed between internal facial features (eyes, nose, mouth) could be related to feature-based processing. The current project investigates if the number of visited facial areas and the number of transitions between different features during facial expression recognition correlate with the level of the inversion effect.

92 participants (46 M, 46 F) took part in experiment. Photos of 2 male and 2 female faces from WSEFEP (Olszanowski et al., 2015 doi: 10.3389/fpsyg.2014.01516) displaying seven facial expressions (neutrality, anger, fear, disgust, happiness, surprise, sadness) were presented randomly for 1300 ms each in three conditions: upright, inverted and Thatcherized inverted. The participant was to recognize facial expression. SMI RED 500 eye tracking technology was applied. Six AOIs were delimited on each face: right and left eyes, nose, mouth, “internal face” (forehead, cheeks and chin) and external features. The number of visited AOIs and the number of transitions were analyzed. The results showed great correlations between eye movement’s strategies subject used in different viewing conditions, but no correlation between the level of the inversion effect and participant’s eye movement parameters. Supported by RFBR, grant 18-013-01087A.

Poster 121

Eye movement strategies in holistic face processing

Pichugina Anna, *Moscow State University*
Menshikova Galina*, Moscow State University*

The role of holistic processing in face perception is actively discussed. The impact of holistic processing on eye movements during face perception is still not well understood. Our aim was to reveal eye movement strategies using the face inversion effect (FIE) as a well-established marker of holistic processing. Fifty seven participants (27 F, 30 M, age range 18-28) were tested. Stimuli were 10 photographs (5 female and 5 male faces). Nine modifications were created for each photograph to impair the facial attractiveness: the distance between eyes or between mouth and nose has been changed. Stimuli were presented in the upright or inverted orientation for 3 seconds. The task was to rate the facial attractiveness on a scale from 1 to 9. During the execution participant's eye movements were recorded. It was found that the dispersion of attractiveness scores was significantly higher for upright faces. AOI analysis for the first three fixations showed the same distribution patterns on main features regardless of the facial orientation. Then for each participant the ratio of average fixation durations to saccade amplitudes was calculated. Using this ratio the whole sample was divided into two groups: the holistic and analytic processors. When looking at upright faces holistic processors spent less time in the area of eyes and lips, and more time in the whole area of nose and nose bridge, than analytic processors. Thus, differences in analytic and holistic face processing can be revealed analyzing eye movement strategies in FIE effect.
Funded by RSF grant, project №19-18- 00474

Poster 122

Fixation-related potentials for refixated faces: Insights from (non)linear deconvolution modelling

Lisa Spiering, Werner Sommer, & Olaf Dimigen
Humboldt-Universität zu Berlin

During natural vision, the same object is often refixated multiple times. Similarly, in laboratory experiments measuring event-related potentials (ERPs), the initial stimulus presentation is often followed by several small saccades on the same stimulus. This raises the question of whether the fixation-related brain potentials (FRPs) produced by each of these refixations merely reflect the saccade-related low-level changes in retinal stimulation or also a (re)processing of the stimulus features at higher (that is, cognitive or affective) levels. To address this question, we recorded EEG and eye movements while participants saw images of neutral, happy, or angry faces for two seconds with the task to detect occasional gaze changes made by the stimulus face. A new (non)linear deconvolution toolbox (www.unfoldtoolbox.org) was then used to disentangle the ERP evoked by the stimulus presentation from the FRPs elicited by subsequent refixations. As expected, the stimulus-locked ERP showed rather typical emotion effects, with enhanced N170 and EPN components for emotional compared to neutral faces. Behavioral analyses confirmed that participants executed small saccades within the eye region in most (98%) trials. However, although these saccades generated strong visually-evoked responses, the FRPs were not modulated by facial emotion, neither for the first nor for subsequent saccades on the face. Results therefore suggest that the affective content of a static face is not necessarily reprocessed during refixations, at least under conditions in which facial emotion is easy to process and task-irrelevant. Methodologically, our results underline the advantages of deconvolution modelling for isolating neural responses during natural viewing.

Poster 123

Perceiving facial expressions in visual search

M. A. Kopachevskaia, G. Ya. Menshikova, O. A. Tikhomandritskaya
Lomonosov Moscow State University
mari.mariya97@mail.ru, gmenshikova@gmail.com, otihomandr@mail.ru

Emotions evolved to provide information about intentions of other people, so perceiving their messages efficiently confers an advantage for interpersonal interactions.In our study we explored the efficiency of facial expression perception using visual search paradigm, namely a search asymmetry effect. Forty participants (25 F, 15 M, age range 19-27) were tested. Ten real-face photographs (5 female and 5 male faces) were used bearing three basic expressions: neutral, happy and sad. The stimulus displays were constructed consisting of an emotional target face (happy or sad) among 11 neutral faces or vice-versa.The displays were presented until the participants responded. They were asked to search for a target face. During the execution participant's eye movements were recorded. The results showed the effect of search asymmetries for both expressions. Its strength was more pronounced in the situation of searching for happy faces among neutral faces compared to searching for sad faces. Gender differences in effect's strength were revealed: it was more pronounced for men regardless of the distinctive facial expression. The analysis of eye movements revealed longer average fixation durations for emotional faces surrounded by neutrals ones than vice versa. Our data showed the specificity of the search asymmetries on socially significant objects.

The study was funded by RFBR grant, project №18-013-01087

Poster 124

Eye-tracking and Dilatation of Sadness in relation to the level of Self-criticism – Pilot Study

Bronislava Strnádelová*[1], Júlia Halamová[1], Róbert Móro,[2] & Marek Mitašík[1]

1 *Institute of Applied Psychology, Faculty of Social and Economic Sciences, Comenius University in Bratislava, Bratislava, Slovakia*

2 *Institute of Informatics, Information Systems and Software Engineering*
Faculty of Informatics and Information Technologies
Slovak University of Technology in Bratislava

Even though self-criticism is considered to be a vulnerability factor for psychopathology, there is no research on dilatation patterns and a very scarce research on the eye-tracking patterns of self-critical people. This pilot research study analyses the relationship between the level of self-criticism, dilatation, and the fixation patterns of participants watching sadness´ and neutral facial emotions. Available sample of thirty-two participants (17 men and 15 women) completed a viewing task while their eye movements and dilatation were being recorded by Tobii X2 60 eye-trackers. They also completed the Forms of Self-Criticising/Attacking and Self-Reassuring Scale (Gilbert, Clarke, Hempel, Miles, & Irons, 2004) with two subscales measuring self-criticism-Hated Self and Inadequate Self. Twelve photos showing sadness and neutral were obtained from the Umeå University Database of Facial Expressions (Samuelsson, Jarnvik, Henningsson, Andersson, & Carlbring, 2012). According to the results, participants with higher Hated Self and Inadequate Self showed significantly higher average fixation time on sadness faces compared to the neutral faces and their dilatations lasted longer. Even though pupils of participants were generally narrowed watching sadness photos compared to neutral photos, participants with higher Hated Self scores had their pupils significantly extended while watching sadness photos compared to neutral photos. Being able to diagnose pathological form of self-criticism by the means of more objective tools than self-rating scales could be very beneficial for psychological and psychiatric practice. Further testing of the findings of this pilot study is highly recommended as well as encouraged.

Key words: eye-tracking, dilatation, facial emotion, sadness, self-criticism

Compliance with Ethical Standards

Disclosure of potential conflicts of interest

The authors declare that they have no potential conflicts of interests.

Funding

Writing this work was supported by the Vedecká grantová agentúra VEGA under Grant 1/0075/19, by Grant mladých UK (Grant number: UK/167/2017).

Ethical approval

All procedures performed in studies involving human participants were in accordance with the ethical standards of the institutional and/or national research committee and with the 1964 Helsinki declaration and its later amendments or comparable ethical standards.

Informed consent

Informed consent was obtained from all individual participants included in the study.

Author Contributions

BS and JH designed research. MM collected data. RM analysed data. BS, JH, RM, and MM wrote the article, interpreted the results, revised the manuscript and read and approved the final manuscript.

Poster 125

A novel perceptual trait: gaze predilection for faces during visual exploration

Guy, Nitzan ; Azulay, Hagar; Kardosh, Rasha; Weiss, Yarden; Hassin, Ran; Salomon, Israel; Pertzov, Yoni
The Hebrew University of Jerusalem; The Hebrew University of Jerusalem; The Hebrew University of Jerusalem; The Hebrew University of Jerusalem; The Hebrew University of Jerusalem; The Hebrew University of Jerusalem; The Hebrew University of Jerusalem

Humans are social animals and typically tend to seek social interactions. In our daily life we constantly move our gaze to collect visual information which often includes social information, such as others' emotions and intentions. Recent studies began to explore how individuals vary in their gaze behavior. However, these studies focused on basic features of eye movements (such as the length of movements) and did not examine the observer predilection for specific social features such as faces. We preformed two test-retest experiments examining the amount of time individuals fixate directly on faces embedded in images of naturally occurring scenes. We report on stable and robust individual differences in visual predilection for faces across time and tasks. Individuals' preference to fixate on faces could not be explained by a preference for fixating on low-level salient regions (e.g. color, intensity, orientation) nor by individual differences in the Big-Five personality traits. We conclude that during visual exploration individuals vary in the amount of time they direct their gaze towards faces. This tendency is a trait that not only reflects individuals' preferences but also influences the amount of information gathered by each observer, therefore influencing the basis for later cognitive processing and decisions.

Poster 126

The cognition of narcissistic aggression: physiological and behavioural correlates of error-monitoring

Smith, William; Lawrence, Claire; Filik, Ruth; Danielmeier, Claudia
University of Nottingham

There is a well-established link between narcissism and aggressive thoughts and behaviours. It has recently been suggested that this may be due to an oversensitive system of error detection in the brain, that raises an alarm when an observed outcome violates an expected outcome (Chester & DeWall, 2016). This alarm may trigger an automatic appraisal categorizing this violation as a potential threat, explaining the exaggerated sensitivity to threat observed in some narcissistic individuals. We investigate whether both grandiose and vulnerable narcissism influence error detection and affect pupil dilation amplitude - a physiological indicator of error-related cognition - during the detection of errors.

Participants (N = 100) completed assessments of grandiose and vulnerable narcissism, trait aggression, borderline personality traits, and self-esteem. They then completed a version of the Eriksen flanker task to assess error detection while pupil diameter was recorded by an EyeLink 1000 eye-tracker.

As vulnerable narcissism and trait aggression increased, changes in pupil dilation amplitude were enhanced during error detection. In contrast, as grandiose narcissism and trait anger increased, changes in pupil dilation amplitude were suppressed, whereas task accuracy was enhanced as grandiose narcissism, trait aggression and borderline personality traits increased.

This contrasting pattern of effects in individuals with otherwise similar traits may reflect different error-response strategies associated with vulnerable and grandiose subtypes of narcissism. Vulnerable narcissists may employ a strategy of avoidance in which an alarm response is useful, whereas grandiose narcissists may employ a strategy of approach in which alarm responses are suppressed to enhance task focus.

CLINICAL/TRANSLATIONAL

Poster 127

A comparison of pre- and postoperative eye movement behaviour in strabismus: challenges and opportunities

Magdalena Krieber-Tomantschger[1], Florian B. Pokorny[1,2], Martina Brandner[3], Susanne Lindner[3], Bianca Bizjak[3], Heike Gaugl[3], Daniela Frühwirth-Kaspar[3], Anna M. Lienhart[3], Karin Steinmair[3], Helena Wagner[3], Andrea Langmann[3], Christa Einspieler[1], Peter B. Marschik[1,4,5], Christof Körner[6]

1 *Research Unit iDN – interdisciplinary Developmental Neuroscience, Division of Phoniatrics, Medical University of Graz, Graz, Austria*
2 *Machine Intelligence & Signal Processing Group, Technical University of Munich, Munich, Germany*
3 *Department of Ophthalmology, Medical University of Graz, Graz, Austria*
4 *Child and Adolescent Psychiatry and Psychotherapy, University Medical Center Goettingen, Goettingen, Germany*
5 *Center of Neurodevelopmental Disorders (KIND), Department of Women's and Children's Health, Karolinska Institutet, Stockholm, Sweden*
6 *Cognitive Psychology & Neuroscience, Institute of Psychology, University of Graz, Graz, Austria*

Strabismus, a constant or intermittent misalignment of the visual axes, interferes with saccade performance and binocular vision. It has been shown to impact on basic oculomotor functions (such as vergence) and complex capacities (such as reading) in both children and adults. Evidence suggests that overall reading performance improves postoperatively, although some deficits in eye movement behaviour may outlast surgical realignment. The nature of postoperative functional improvement for different types of strabismus, however, is largely unknown. In our ongoing interdisciplinary study we use eye tracking to investigate basic oculomotor functions and reading processes for monocular and binocular viewing in individuals with constant vs. intermittent strabismus. To investigate the effects of surgical correction we use a repeated-measures design consisting of a baseline- and a 4-months-postoperative-assessment. Focusing on single cases of our sample, we highlight methodological challenges associated with eye tracking in individuals with strabismus (e.g., poor visual acuity as a result of amblyopia, gaze estimation accuracy in difficult-to-calibrate participants). Preliminary results from ophthalmologic assessments, basic oculomotor tasks and their association with reading processes (pre-/postoperative design) in constant vs. intermittent strabismus are converged for clinical decision making and add to the discussion of 'cosmetic and/or functional repair'.

Poster 128

Distinguishing between impairments of working memory and inhibitory control in cases of early dementia

Trevor J. Crawford
Centre for Ageing Research
Department of Psychology
Lancaster University
Lancaster, LA1 4YF, UK

Dementia (most notably, Alzheimer's Disease) is often associated with impairments of both working memory (WM) and inhibitory control (IC). However, it is unclear whether these are functionally distinct impairments. So far the eye-tracking studies of IC have relied heavily on studies that are based on the average scores from groups that were tested at a given time point. A detailed assessment of individual cases can address questions in relation to the dissociation of cognitive operations, which cannot be resolved by the average scores from a group of diverse patients. The patient group consisted of 18 patients with early dementia (13 males, 5 females). An old control group of 18 healthy participants (8 males, 10 females) were volunteers from the local community. All participants underwent a detailed neuropsychological assessment. Tests for the dissociations of neurocognitive inhibitory control (anti-saccade) and working memory span were conducted with reference to the control sample using the revised standardized difference tests. 33% patients from the original sample met the Crawford and Garthwaite (2005) statistical criteria for a "strong" dissociation. Some patients revealed a preserved working memory capacity together with poor inhibitory control in the anti-saccade task. A longitudinal follow-up revealed that the defective anti-saccade control emerged 12-months before the dementia was evident on the mini-mental state examination assessment. Other cases revealed a poor working memory together with a well-preserved level of anti-saccade errors. There is increasing evidence that people with early Alzheimer's disease have subtle impairments in cognitive IC that are often undetected by traditional cognitive assessments.

Poster 129

Double-step adaptation in 4 or less session for vergence in adults

Aurélien, Morize ; Botao, Peng; Dominique, Brémond-Gignac; Zoï, Kapoula
CNRS FR2022; CNRS FR2022; APHP; CNRS FR2022

Oculomotor neuroplasticity in adults is a well-known mechanism through saccades' adaptation paradigms, interested on robust and stereotype movements such as saccades. Alahyane and Pélisson (2005) shown that double-step paradigm induces recover within a few days. Respectively, studies were far less numerous to care about such time course effects when applied to binocular vergence, even since Takagi and Zee (2001) demonstrated the efficiency of such paradigm in healthy. Recently (2016) we objectivised in convergence insufficiency a monthly persistence, originality being the vergence double-step paradigm (REMOBI patent:US8851669). This has significant interests, mainly in clinical therapy. Traditional clinical practice requires 12-18 hours, including home-based exercises together with 6-12h of office-based, distributed along 12 sessions, a consensus that aim to elicit thousands of vergence movements. That practice aims to adapt vergence from artificial stimuli to an ecological environment. Alvarez (2010) focused on this kind of practice, mostly with artificial disparity vergence but together with fMRI, in order to objectivise involved substrates (mainly the brain stem, the cerebellum and the Frontal Eye Field); alternatively, we were rather interested by the time course that characterises binocular neuroplasticity along the double-step paradigm (i.e in ecological environment). We aim to substantiate the therapeutic curve and better understand the training duration that could be recommended to nurture efficiently and durably binocular neuroplasticity. Accessorily, we tested a new semi-automatic calibration method.

Subjects with vergence disorders (N=12, 20-77 y.) were recruited at Necker-Enfants malades, in order to compare patients' performances to healthy subjects (N=8), by coupling the assessment device with eye tracking (EyeSeeCam). We applied the REMOBI method in line with traditional clinical practice, to elicit thousands of movements, but within a reduced time course: the first group (N=8) performed only 4 training sessions that contained 720 double-step vergence and at least as much step vergence quantity. The second group participated to a reduce number of double-step convergence (2-4 sessions of same duration). Before and after training, we tested vergence with an overlap paradigm, which required 20 vergence movements in each direction. To summarize, a total of 5920 office-based vergence for the first group, which is twice or even thrice the quantity of vergence manually elicited in traditional clinical practice. The subjects did not perform any home-based additional exercise. After training, we performed follow-up tests at 1-8 months. And both groups were compared with controls (N=2), namely subjects whith vergence disorders who performed a traditional clinical training together with the same vergence tests. Finally, statistical analyses were performed with multiple ANOVA.

In line with our prior studies, we observed a significant increase of promptness (1/latency), amplitude and velocities that occurred in less than 4 sessions, both for convergence and divergence. Results provide additional investigation on subgroups and long or short terms repetition.

The study demonstrated the efficiency of the REMOBI method and more broadly the good receptivity of ocular neuroplasticity in subjects with vergence disorders. Also we validated for the first time both the calibration and the analysis methods. This facilitated and opened the way for additional studies on binocular neuroplasticity.

Alahyane, N., & Pélisson, D. (2005). Long-lasting modifications of saccadic eye movements following adaptation induced in the double-step target paradigm. *Learning & Memory, 12*(4), 433-443.

Takagi, M., Oyamada, H., Abe, H., Zee, D. S., Hasebe, H., Miki, A., ... & Bando, T. (2001). Adaptive changes in dynamic properties of human disparity-induced vergence. *Investigative ophthalmology & visual science, 42*(7), 1479-1486.

Kapoula, Z., Morize, A., Daniel, F., Jonqua, F., Orssaud, C., & Bremond-Gignac, D. (2016). Objective evaluation of vergence disorders and a research-based novel method for vergence rehabilitation. *Translational vision science & technology, 5*(2), 8-8.

Kapoula, Z., Yang, Q., Eggert, T., Daunys, G., & Orssaud, C. (2014). U.S. Patent No. 8,851,669. Washington, DC: U.S. Patent and Trademark Office.

Alvarez, T. L., Vicci, V. R., Alkan, Y., Kim, E. H., Gohel, S., Barrett, A. M., ... & Biswal, B. B. (2010). Vision therapy in adults with convergence insufficiency: clinical and functional magnetic resonance imaging measures. Optometry and vision science: official publication of the American Academy of Optometry, 87(12), E985.

Poster 130

Exploiting pupil and cortical oscillatory responses to flickering stimuli for decoding shifts of attention in depth.

Claudio de'Sperati, Regina Gregori-Grgič, Niccolò Zovetti, Tatiana Baroni
Laboratory of Action, Perception and Cognition, Università Vita-Salute San Raffaele; and Unit of Experimental Psychology, San Raffaele Neuroscience Division

The Locked-In Syndrome (LIS) is a very rare but devastating condition, characterized by the complete paralysis of the voluntary musculature, except for some eye and eyelid movements. Sometimes, however, the oculomotor control is so compromised, for example in an advanced phase of Amyotrophic Lateral Sclerosis (ALS), that these patients lose the possibility to communicate and interact with the external world. We have developed a prototype of Brain-Computer-Interface (BCI) based on the voluntary shift of attention (gaze) from a far target to a near target, which is associated to a decrease of pupil size, an automatic sympathetic response [1]. In a sample of healthy volunteers, we explored the possibility of exploiting this pupillary response (Pupillary Accommodative Response) in combination with the oscillatory response of the pupil (Pupillary Oscillatory Response) and the visual cortex (Steady-State Visual Evoked Potentials) to flickering stimuli, to establish binary communication (shifting attention to a near target meaning "yes", holding attention on a far target meaning "no"). These three signals (PAR, POR and SSVEP) discriminated when the observer's attention was on the far or the near target, and were decoded by means of a binary classifier (Support Vector Machine) to detect subject's voluntary response. Our results suggest that multiple signal decoding during a simple attention shift in the depth plane can be a robust strategy to communicate with LIS patients when oculomotor control is too poor to use traditional assistive aids based on eye tracking.

[1] **Porzio et al** (2019). *Int J Hum Comp Stu 126*, 53-63.

Poster 131

Eye movements characteristics as a potential indicator of psychopathology. Experiences from clinical practice.

Kroll, Aleksandra ; Danczura, Ewa; Szelepajlo, Michal; Mak, Monika
Pomeranian Medical University; Pomeranian Medical University; Pomeranian Medical University; Pomeranian Medical University

In the fields of medicine regarding mental health there is a need to search for diagnostic methods which would help remedy problems such as for example simulation or dissimulation, confounding learning effect, etc. Eye movement research are among the techniques which could be considered as helpful in alleviating these problems.
This report presents preliminary research results including clinical groups of participants with schizophrenia (SCH) and depression (DEP) in comparison to a control group (CTRL).
These 3 groups of participants (N=10 in each group) performed a CRTT (choice-reaction time task) where the eye-movements were recorded using Jazz-novo eye tracker. The test procedure was run in the psychoPy software.
We observed that SCH participants had longer saccades duration (M=755 ms) than CTRL (M=327 ms) group [$F(2,18) = 2.48$, $p = .058$] as well as DEP (M=645 ms). What is more fixation duration average time was shortest in SCH (M=354 ms), then in DEP (M=456 ms) and CTRL (M=464 ms). During the first 10 seconds of the experiment procedure the mean saccades count was highest in the SCH group (M=22.43) while it was the same for DEP (M=19.57) and CTRL (M=19.57) groups.
In our proposal we also sum up methodological problems which appeared during eye movement research in the clinical setting, like for example dealing with patients in different stages of illness.

Poster 132

Eye movements during reading on a computer screen with progressive power lenses

Concepcion,P; Gonzales,A; Chamorro, E; Cleva,JM; Alonso, J
IOT

Progressive addition lenses (PPL) are the most common solution for presbyopic subjects, who are unable to focus on near objects due to a physiological decrease of accommodation with age. These lenses are characterized by having an increase in spherical power from the upper to the lower portion of the lens. This geometrical change induces unwanted second order aberrations and variable distortion in the lateral sides of the lens, which negatively impacts vision. So, it is expected that eye movements may be affected when using PPLs. In the present study, an eye-tracker system (Tobii-X2-30) was used to register eye movements of 20 PPL users when reading on a computer screen. The number of fixations, complete fixation time, fixation duration mean, saccade duration mean, saccade distance mean and number of regressions were analysed at 6 different regions of the computer screen. Results showed a higher reading efficiency when reading in the central and inferior areas of the screen, matching when the subject is using the near area of the lens free of unwanted astigmatism. So, the results of this study suggest the importance of eye movement evaluation in presbyopic patients and using the results for PPL selection and adaptation.

Poster 133

Face scanning pattern of individuals with ASD changes after the treatment of sleep disorder

Kato, Masaharu; Toyoura, Makiko; Amagai, Kyoichi; Funabiki, Yasuko; Konishi, Yukuo
Doshisha University; Hyogo Rehabilitation Center Hospital; Kyoto University; Kyoto University; Doshisha University

Autistic spectrum disorder (ASD) is a developmental disorder in brain. The number of individuals with ASD exponentially increases but the causes are not clarified, and decisive treatments are not found yet. Recent research has shown that about 40-80% of individuals with ASD has sleep disorder (Fadini et al., 2015). Interestingly, it is reported that the symptoms of ASD was remitted by the medical treatment on sleep disorder. Previously we measured the face scanning pattern of typically developed (TD) adults and individuals with ASD and found that the degree of similarity of face scanning pattern between the same group participants (similarity index) was higher for TDs than for ASDs (Kato et al., 2015).

Thus, in the current study, we measured the similarity index for ASDs and that for non-ASDs (TDs) before and after the treatment to see whether the similarity index changes or not, which could be a qualitative evaluation of ASD symptoms. As predicted, we found that the similarity index increased after 2 months of sleep disorder treatment for ASDs, which supports the view that the sleep disorder treatment may have some effects on the characteristics of face looking behavior. In addition, neither the gaze distribution nor temporal distribution of looking did not change by the treatment, which indicates that the ability of collecting configural information rather than that of collecting featural information was influenced by the treatment.

References

Fadini, CC., et al. (2015). *Front Hum Neurosci*, 9, 347.
Kato, M., et al.(2015). *18th European Conference on Eye Movements*.

Poster 134

Free viewing in neurodevelopmental disorders

Ioannou, C., Canu, D., Fleischhaker, C., Biscaldi, M., Klein C.
University Hospital Freiburg; University Hospital Freiburg; University Hospital Freiburg; University Hospital Freiburg; University Hospital Freiburg, University Hospital Koeln

Background – Free viewing behaviour of participants with neurodevelopmental disorders like schizophrenia or autism may differ from that of neuro-typical controls in various regards such as constraint gazing or gaze preferences for certain elements of a scene. However, despite multiple ”pair-wise” clinical and biological relationships between the three neurodevelopmental disorders, there is, to our knowledge, no direct comparison between participants with autism, ADHD and schizophrenia. This head-to-head comparison is the primary objective of the present study.
Methodology – We tested four groups of participants aged 14-23 years old, matched for age and sex, namely early-onset Schizophrenia (SZ), ADHD, Autism, and typical controls. Our ocular-motor task battery included different free viewing tasks such as free viewing of social interactions under solely viewing and viewing-plus-speaking conditions, Rorschach items, emotional faces, and solving items from Raven’s progressive matrices. Eye movements were recorded with 1,000 Hz using an EyeLink 1000 Plus system.
Results – Preliminary analyses indicate that participants with early-onset SZ show a constraint gaze scatter when solely viewing at social interactions. This restriction was compensated while viewing and speaking about the scene. Participants with ADHD did not differ from typical controls.
Discussion and conclusions – The results of the present study have implications for our nosological understanding of neurodevelopmental disorders by showing commonalities and differences between these groups. Based on the results regarding the SZ patients, appropriate specific interventions can be developed to facilitate and improve their quality of life.

Poster 135

Impaired visual localization across saccades in a patient with parietal lesion

Alexis Cheviet, Laure Pisella and Denis Pélisson

Integrative Multisensory Perception Action & Cognition Team (ImpAct), Lyon Neuroscience Research Center (CRNL), INSERM U1028, CNRS UMR5292, University Claude Bernard Lyon 1, Université de Lyon, 69000 Lyon, France

Spatial remapping processes are elicited every time we scan our environment with our eyes in order to explore / act on it. These mechanisms use extra-retinal signals of eye motion to predict the sensory consequences of saccadic eye movements. Studies using double-saccade remapping tasks have emphasized a role of the colliculo-thalamo-frontal pathway[1] and of the parietal cortex[2] in keeping accurate visuomotor behaviour. However, the perceptual spatial remapping processes underlying our subjective experience of stability across saccades are less understood. A study of saccadic suppression of displacement (SSD) in thalamic patients[3] showed that the colliculo-thalamo-cortical pathway contributes to visual localization across saccades. A role of the parietal cortex in such perceptual remapping processes has also been suggested by theoretical studies[4] but has not been shown yet. Here, we address this question by testing a patient with a right posterior parietal lesion in a SSD-task and a localization task in which a bar briefly flashed just before a saccade has to be localized thereafter[5]. In the localization task, strong overestimations of the bar position were found in the patient's affected hemifield relative to healthy controls (n=4). This pattern disappeared in the patient's un-affected hemifield and tended to change direction opposite (underestimation) in an eye fixation condition as well as. In the SSD-task, preliminary analyses suggest that performances of the patient were lower within the affected hemifield as compared to the preserved hemifield. These findings suggest a role of parietal cortex in trans-saccadic visual localization, possibly in processing extra-retinal information of saccade execution.

References

(1) **Sommer MA,Wurtz RH**. 2002. A pathway in primate brain for internal monitoring of movements. *Science 296*:1480–82

(2) **Duhamel JR, Colby CL, Goldberg ME.** 1992a. The updating of the representation of visual space in parietal cortex by intended eye movements. *Science 255*:90–92

(3) **Ostendorf, F., Liebermann, D., and Ploner, C. J.** (2013). A role of the human thalamus in predicting the perceptual consequences of eye movements. *Front. Syst. Neurosci . 7*:10.

(4) **Ziesche, A., & Hamker, F. H.** (2014). Brain circuits underlying visual stability across eye move-ments converging evidence for a neuro-computational model of area lip. *Frontiers in Computational Neuroscience, 8*, 25.

(5) **Zimmermann, E., and Lappe, M.** (2009). Mislocalization of flashed and stationary visual stimuli after adaptation of reactive and scanning saccades. *J. Neurosci. 29*, 11055–11064.

Poster 136

Interaction between gaze perception and facial emotional processing in Post-Traumatic Stress Disorder Following Paris Terrorist Attacks in November 2015

S.Y. Coll, F. Eustache, D. Peschanski, C. Klein-Peschanski, F. Doidy, F. Fraisse, J. Dayan, P. Gagnepain, M. Laisney

University of Caen; University of Caen; University of Paris; University of Paris; University of Caen; University of Caen; University of Rennes; University of Caen; University of Caen

Terrorist attacks the 13th of November 2015 in Paris, have greatly moved and shocked people. In order to understand the psychological and neurobiological consequences of trauma, the 13-November program was launched. In this context, our study focuses on the social cognition abilities of participants having been directly exposed to these events. Several works show an impairment in some social skills in post-traumatic stress disorder (PTSD), that may be associated with an abnormal processing of emotions. For instance, a bias toward negativity has been shown, as well as emotion recognition difficulties. Although, evidence in the literature on anxiety disorders seem to highlight the importance of the eye area in these deficits, the interaction between gaze perception and emotional processing in PTSD remains largely unknown. To better understand the scope of this interaction, 159 participants, including 52 exposed to Paris terrorist attacks who developed a PTSD, 51 exposed to the same trauma without a PTSD, and 56 unexposed controls, participated in one study involving the recording of eye movements during a gaze-cueing attentional task using emotional faces. In this study, participants were asked to detect the location of a target correctly or incorrectly cued by the gaze of a face communicating sadness, happiness, or a neutral expression. In this poster, we will present and discuss eye movement data, which highlight how the processing of emotional faces is impaired in the PTSD and the importance of gaze detection in their deficits.

Poster 137

Placebo effects on visual food cue reactivity: an eye-tracking investigation

Jonas Potthoff*, Nina Jurinec, Anne Schienle
University of Graz, Department of Clinical Psychology, Graz, Austria
**jonas.potthoff@uni-graz.at*

Background: Enhanced visual food cue reactivity has been associated with overeating and weight gain [1]. Due to the increasing exposure to high-caloric food images, methods that are able to reduce the reactivity to these cues are urgently needed.
Method: In the present eye-tracking study it was investigated whether the visual bias for high-caloric food can be altered via placebo treatment. Fifty-two healthy women (mean BMI = 23.5) were presented with pictures depicting combinations of food (high-caloric, low-caloric) and non-food items, which were shown once with and once without a placebo (a pill introduced as a medication targeting peptide YY that is able to reduce appetite specifically for high-caloric food).
Results: The placebo reduced reported appetite, the percentage of fixations and the dwell time on food images. The placebo was not able to specifically reduce visual food cue reactivity to high-caloric stimuli.
Conclusions: The placebo was able to generally reduce visual food cue reactivity. This finding encourages future studies with other samples (e.g. patients with a binge-eating disorder) and also indicates the need of adapting placebo interventions to become more specific.

Reference:

[1] **Boswell, R. G., and Kober, H.** (2016). Food cue reactivity and craving predict eating and weight gain. A meta-analytic review. *Obesity reviews : an official journal of the International Association for the Study of Obesity 17*, 159–177. doi: 10.1111/obr.12354.

Poster 138

Temporal evolution of the objective fixation disparity

Sanchis-Jurado, Vicent; Pons, Álvaro
University of Valencia

In the clinical context fixation disparity (FD) is measured by means of subjective methods and reported as a single value as if it were a constant misalignment over time. The existence of the fixational eye movements implies that the angle formed by the visual axes is changing constantly, so the value of the objective fixation disparity (OFD) is changing constantly as well.
To analyse the dynamic behaviour of the OFD we recorded for 45 seconds the eyes of 15 volunteers while staring to a fixation point at 1 m in front of a chinrest. The position of each pupil centre was extracted and projected on the stimulus plane. The angular difference between each eye visual axis and the appropriate position of the axis were calculated in the horizontal plane.
For all the subjects the OFD values over time did not follow a normal distribution. Three linear regression models were fitted to the data. The first one was fitted to all the data to see if there was a positive, negative or zero trends in the OFD variation over time. The second and third models were fitted only to the maximum and minimum values, respectively, to check if the data was convergent or divergent in time.
Six subjects showed a zero trend, two a positive and seven a negative trend. Thirteen subjects showed a convergent and two a divergent trend. The average median was -4.68 ± 14.75 minutes of arc, compatible with values reported by other authors previously.

Poster 139

The effects of working memory load and ADHD-like traits on image viewing

Martinez Cedillo A. P.
Dent, K.
Foulsham, T.
Department of Psychology. University of Essex. United Kingdom.

Avoiding distractors is crucial for our daily lives. Load theory argues that high perceptual load facilitates distractor avoidance, while high cognitive load impedes distractor avoidance (see, Lavie, 2005). Difficulty in avoiding distractors is one of the key symptoms of Attention Deficit/Hyperactivity Disorder (ADHD; APA, 2013). In a series of experiments, we investigated visual attention during the viewing of complex scenes (featuring social and non-social objects), while manipulating Working Memory Load (WM) in a concurrent task. We also explored the relationship between these tasks and ADHD-like traits. Fixations during scene viewing were investigated with reference to objects of high and low saliency with social area (a person) also imbedded in the image. We tested the hypothesis that high WM load would lead to increased capture by the salient distractor. In contrast, attending to the social item might require more top-down resources and so be disrupted by WM load. The pattern of results suggests that during image viewing the social object was fixated to a greater degree than the other object (regardless of salience). While, there was a relationship between the degree of ADHD-like traits and performance on the memory task, WM load did not seem to affect scanning in scenes. Such findings suggest that top-down resources are not needed to attend to a social area in complex stimuli.

Poster 140

Treatment-induced eye movement responses for Parkinson’s disease

Zi H.Su[1], James J. FitzGerald [1,2] and Chrystalina A. Antoniades[1]

1 *NeuroMetrology Lab, Nuffield Department of Clinical Neurosciences, University of Oxford, Oxford, OX3 9DU, UK.*

2 *Nuffield Department of Surgical Sciences, University of Oxford, Oxford, OX3 9DU, UK.*

Parkinson’s disease (PD) is a slowly progressive neurodegenerative disease where the gradual death of dopaminergic cells in the substanta nigra and consequent loss of dopaminergic projections to the striatum disrupts the function of cortico-basal ganglia circuits. This results in motor symptoms such as rigidity, bradykinesia (slowness of movement) and tremor. The pathways affected by PD are also involved in the generation of eye movements. Although oculomotor pathways have been extensively studied, PD treatment-induced changes in their parameters are not well known.

There is currently no disease-modifying treatment for PD. Most cases are treated symptomatically with levodopa or Deep Brain Stimulation. One of the factors limiting the rate of development of new treatments, is the reliance on clinical rating scales which are subjective, insensitive and non-linear. Analysing the effect of current treatments on oculomotor response allows characterization of new treatment-assessment tools that is quicker and more sensitive. Previous studies have identified oculomotor parameters as sensitive markers due to their ability for rapid data collection and changes in de-novo PD patients. In addition, treatment-induced changes in gait parameters and manual responses will be used as covariates in analysis to further ensure accuracy. Eye movement, gait and manual responses of PD patients either On or Off medication or stimulation, depending on which treatment they are receiving, are measured along with the usual clinical assessment UPDRS. Objective and quantitative measurements revealed several parameters that are characteristic to each treatment response.

Poster 141

Visual Object Exploration Processing in Patients with Schizophrenia

Yeseul Kim, Taehyun Kim, Jinyeong Choi, Sunhyun Moon, Soo Rim Noh *

Chungnam National University; Gongju National Hospital; Seoul National University; Chungnam National University; Chungnam National University

Knowing the name of and recognizing objects accurately are very important functions in daily life. In the visual object exploration task, patients with schizophrenia show relatively less exploration of the relevant part for identification when asked to name the object. This can negatively affect patients with schizophrenia. In this study, 22 patients with schizophrenia and 24 healthy controls were monitored as they viewed 24 daily tools (ex: hammer, toothbrush) in performing the free-viewing and object-naming tasks. Overall, patients with schizophrenia and healthy controls fixated more and longer on the relevant part for identification when asked to name the object than other parts of the object. Additionally, their first fixation on the relevant part of identification was longer than that on the other parts of the object. There was the no difference between patients and controls in the accuracy of the object-naming task. Moreover, patients with schizophrenia fixated less and longer than normal in the relevant part for identification. This suggests that although there is the no difference in accuracy, patients have difficulty in knowing the object's name and recognizing it due to their reduced attention to the relevant part. This indicates that patients with schizophrenia and healthy controls have different exploration of object scan paths, and this difference can impair the object recognition and exploration abilities of patients with schizophrenia.

Reference

1. **Delerue, C., & Boucart, M.** (2012). The relationship between visual object exploration and action processing in schizophrenia. *Cognitive neuropsychiatry, 17*(4), 334-350.

Poster 142

Visual Scanpaths to Facial Emotions in Patients with Schizophrenia

Noh, Soo Rim; Kim, Yeseul; Moon, Sun Hyun; Kim, Taehyun

[1]*Department of Psychology, Chungnam National University, Daejeon, South Korea; [1]Department of Psychology, Chungnam National University, Daejeon, South Korea; [1]Department of Psychology, Chungnam National University, Daejeon, South Korea; Department of Psychiatry, Gongju National Hospital, Gongju, South Korea*

The visual scanpaths of 73 patients with schizophrenia and 97 healthy controls were monitored as they viewed happy, sad, angry, fearful, disgusted and neutral faces for identifying facial expression. Compared to controls, patients with schizophrenia performed poorly on recognizing the facial emotions of anger, fear and disgust, and neutral faces. For the analysis of visual scanpaths to salient features of faces, the eye, nose, and mouth areas were confined using the limited-radius Voronoi tessellation method. Overall, patients with schizophrenia fixated less frequently, but they fixated longer on the salient features of faces than controls. Moreover, their first fixation on the salient features of faces was longer than that of the controls, and their fixations were longer for eyes than mouth for angry and neutral faces. These results suggest that the lack of facial emotion recognition in patients with schizophrenia may be due to their reduced attention to the salient features of faces (as indexed by fewer fixations) and their difficulty in interpreting those features (as reflected in longer duration). Interestingly, among the patients, the frequency of fixation on the salient features of faces was positively correlated with the accuracy of facial emotion identification, indicating that directing their attention to the salient features of faces could enhance their emotion recognition accuracy.

This work was supported by the National Research Foundation of Korea (NRF) (Grant number NRF-2016R1E1A2A01953732 & 2018R1E1A2A02059043).

Poster 143

Modeling individual differences in fixational eye movements

Seelig, Stefan ; Engbert, Ralf; Sebastian, Reich;
University of Potsdam

Visual information processing is limited to fixations where the gaze is approximately stationary. Miniature (or “fixational”) eye-movements, however, are produced involuntarily. We developed a dynamical model of fixational eye movements (Engbert et al., 2011) that is based on a self-avoiding random walk in a potential. Additionally, the control of fixational eye movements is influenced by temporal delays (Herrmann et al., 2017). Here we present a fully Bayesian framework of parameter estimation using advanced Markov Chain Monte Carlo procedures (Vrugt et al., 2009). When simulating data by using parameter configurations from the sampled posterior distributions, we can reproduce the properties of fixational eye movements on the level of individual participants.

Acknowledgments:
This work was supported by Deutsche Forschungsgemeinschaft (Collaborative Research Center 1294 “Data Assimilation”, project B03).

References:

Engbert, R., Mergenthaler, K., Sinn, P., & Pikovsky, A. (2011). An integrated model of fixational eye movements and microsaccades. *Proceedings of the National Academy of Sciences, 108*(39), E765–E770.
Herrmann, C. J., Metzler, R., & Engbert, R. (2017). A self-avoiding walk with neural delays as a model of fixational eye movements. *Scientific Reports, 7*(1), 12958: 1–17.
Vrugt, J. A., Ter Braak, C. J. F., Diks, C. G. H., Robinson, B. A., Hyman, J. M., & Higdon, D. (2009). Accelerating Markov chain Monte Carlo simulation by differential evolution with self-adaptive randomized subspace sampling. *International Journal of Nonlinear Sciences and Numerical Simulation, 10*(3), 273–290.

Poster 144

Spiking neural network models of saccade generation

Anno Kurth[1], Mario Senden[2, 3], Sacha J. van Albada[1]

1 *Institute of Neuroscience and Medicine (INM-6) and Institute for Advanced Simulation (IAS-6) and JARA-Institut Brain Structure-Function Relationships (INM-10), Jülich Research Centre, Jülich, Germany*

2 *Department of Cognitive Neuroscience, Faculty of Psychology and Neuroscience, Maastricht University, 6201BC Maastricht, The Netherlands 3 Maastricht Brain Imaging Centre, Faculty of Psychology and Neurosience, Masstricht University, P.O. Box 616, 6200 MD Maastricht, The Netherlands*

Saccade generation is a fundamental example of eye-movement control and an integral part of attentional selection. In order to understand the latter two better one can try to gain a qualitative understanding of the former. Several models describing the process of saccade generation have been developed, taking anatomical and electrophysiological findings into account to varying degrees ([1]). Among these, the model of the saccade generator in the brainstem reticular formation by Gancarz and Grossberg ([2]) is able to reproduce observed functional aspects and, at the same time, respect neuron characteristics.

All these models, however, share a common ansatz: They collapse a number of neurons with similar characteristics into a population then studying their interactions and functions in terms of ordinary differential equations. While these models yield valuable insights regarding the population-level dynamics of saccade generation, their top-down approach neglects possibly rich internal dynamics and intricate interactions on the single-neuron level. In order to dissolve this limitation and to increase the biological realism, we compare different approaches to construct spiking neural networks incorporating the saccade generation mechanism of [2]. Starting with the Neural Engineering Framework ([3]), we show how spiking models can support the corresponding mechanism.

Acknowledgments: Funded by the European Union's Horizon 2020 Research and Innovation program under grants 720270 and 785907 (Human Brain Project SGA1, SGA2).

References:

[1] **Girard B, Berthoz A** (2005). From brainstem to cortex, Computational models of saccade generation circuitry. *Progress in Neurobiology 77* 215-251

[2] **Gancarz G, Grossberg S** (1998). A neural model of the saccade generator in the reticular formation. *Neural Networks 11* 1159-1174

[3] **Eliasmith C, Anderson CH** (2003). Neural Engineering: Computation, Representation, and Dynamics in Neurobiological Systems, *Cambridge, MA*: MIT press

Poster 145

A fully convolutional model of empirical saliency based on deep CNN features

David Dubray & Jochen Laubrock
University of Potsdam Germany

What is the best way to describe a visual scene? What aspects of a scene attract eye movements and attention? Inspired by Treisman's feature integration theory, early computational models used a number of relatively basic features such as maps of colors, intensity, and orientations, to model the distribution of attention. However, it has since become clear that object-level represenations play a major role over and above such basic features. Until recently, models including representations of features from mid-levels vision or higher-level processing in the ventral stream were not available. Deep convoluational features implemented in the new wave of computer vision models represent features at hierarchical stages, from elementary features via textures and motifs to proto-objects, and might thus provide an novel approach to the above questions. Whereas several such models have been presented in the computer science community, they have still been relatively unexplored in the community interested in eye movements. Here we present a fully convolutional model of empirical saliency based on deep CNN features. We had originally developed the model for an image segmentation task, namely, pixel-wise segmentation of speech balloons in comics. Retraining the model to kernel density estimates of empirical fixation distributions obtained from a large data base of eye movements during scene perception provided a good fit. An advantage of a model is that it allows one to look inside. An investigation of features at which level contributed most will be reported.

Poster 146

Using eye tracking to evaluate the learning perfor-mance in text reading

Gao, Hong; Appel, Tobias; Castner, Nora; Kasneci, Enkelejda
University of Tuebingen

Learning processes during reading text can be investigated with eye tracking. Gaze behavior is indicative of effective learning processes and learning per-formance. Once the relationship between eye movement and learning per-formance is clear, what influences our way of learning during text reading can be figured out, which is crucial to fostering Self-regulated learning (SRL) in modern education. 247 college students read a 450-word text for five minutes and answer questions after reading. We split subjects high text comprehen-sion and low text comprehension based on post-reading questions. Based on the gaze behavior, we recorded the time it took students to read through the text. We found that group with high comprehension (M=150.33s, SD:33.43s) read slightly faster than the group with low comprehension (M=168.82s, SD:39.7s). More interesting, after this first read through, i.e. gaze behavior during the remaining minutes/seconds, students with high comprehension looked again at the first and second paragraph (three total), showing similar gaze behavior to a second read through or scanning. Conversely, the low comprehension group showed less homogeneous gaze behavior not indica-tive of reading or scanning. Gaze-based AOIs (calculated in *EyeTrace*: Kübler, 2015) for the high comprehension group were on numbers, verbs, and adjectives and had fewer transitions between paragraphs. This gaze be-havior after the first read through could be reflective of effective text compre-hension as measured from their performance on the questionnaire.

Poster 147

Recurrence Quantification Analysis and Mental Imagery

Gurtner, Lilla; Bischof, Walter; Mast, Fred

Department of Psychology, University of Bern, Bern, Switzerland; Department of Psychology, University of British Columbia, Vancouver BC, Canada; Department of Psychology, University of Bern, Bern, Switzerland

Eye movements during mental imagery show a distinctive spatial and temporal pattern. While it has been known for a long time that fixation locations during mental imagery resemble those during perception, we showed in a recent study (Gurtner et al., 2019) that the temporal dynamics of fixations differ between perception and mental imagery. Using recurrence quantification analysis, we found that eye fixations return more often and sooner to previously inspected areas during mental imagery than during perception.
To further investigate this difference we analyzed the role of re-fixations for maintaining mental images. In two experiments, we tested the hypothesis that systematic re-visits of a limited number of locations can counteract fading of the imagined content. In the first experiment, we manipulated the demands of maintenance by increasing the complexity of the pattern that needed to be maintained. In a second experiment, dynamic visual noise interfered with the mental image. The results contribute to a better understanding of eye movements during mental imagery by including the temporal dynamics of fixations in addition to spatial congruence.

Gurtner, L. M., Bischof, W. F., & Mast, F. W. (2019). Recurrence quantification analysis of eye movements during mental imagery. *Journal of Vision, 19*(17), doi:10.1167/19.1.17

Poster 148

Microstimulation in V1 of behaving monkeys: spatio-temporal patterns in relation to evoked saccades

Roy Oz[1], Hadar Edelman-Klapper[1], Shany Nivinsky-Margalit[1], Hamutal Slovin[1]
1 The Gonda Multidisciplinary Brain Research Center, Bar-Ilan University, Ramat Gan, Israel

Intra-cortical microstimulation (ICMS) is a well-established technique to artificially stimulate the brain, enabling to explore the organization and function of the cortex. Previous studies have shown that electrical stimulation in the primary visual cortex (V1) of humans and monkeys can generate the perception of Phosphenes and can also evoke saccades directed to the receptive field (RF) of the stimulated neurons. However, little is known about the neural activity and the spatial patterns evoked by ICMS in V1 and how it relates to the evoked saccades. To investigate this, we performed microstimulation through a microelectrode placed in monkeys' V1 while the animals were performing a fixation task. Population response was measured at a high spatial (meso-scale) and temporal resolution using Voltage Sensitive Dye (VSD) Imaging.
ICMS evoked population responses extending over few mm within V1 and in some cases the neural response propagated to cortical interconnected areas: V2 and V4. Although the monkeys were trained to fixate, in several recording sessions, the animals performed saccades that were directed to the RF of the stimulated region. These saccades occurred within 400 ms after ICMS onset and had large latency variance. Analysis of the VSD signal aligned on ICMS onset, revealed increased population activity in the area surrounding the electrode for trials where the monkey performed saccades (to the stimulated RF site) compared with trials where it kept fixation or made saccades to other directions. In conclusion, ICMS in V1 evoked neural activity patterns that were related to the generated saccades and/or phosphenes.

Poster 149

Robust data smoothing method for fixational eye movements analysis

Doubrovski V.E., Garusev A.V., Luniakova E.G.
Lomonosov Moscow State University, Faculty of Psychology
E-mail vicdubr@mail.ru

Any eye tracker produces noisy recordings due to errors of a complex algorithm that calculates an instantaneous gaze direction. In most studies, this noise can be neglected, since the error is rather small. But in the studies of fixational eye movements the noise level of the equipment matches the amplitude of the useful signal. Data smoothing methods based on local polynomial regression with quadratic loss function (Savitzky, Golay, 1964) are commonly used to reduce the noise. Limitation of those methods is that the linear smoothers are very sensitive to outliers and leaps. To remove outliers a preliminary median smoothing is usually performed. It would be quite important to have one robust procedure as an alternative to this two-step smoothing process. We suggest a new implementation of a robust smoothing technique based on Huber M-smoother (Tsybakov, 1982a, 1982b, 1983). This method with a non-quadratic loss function is intermediate between median smoother and the ordinary Nadaraya-Watson kernel smoother (Härdle, 1990). An iterative algorithm is conventionally used to calculate the Huber estimate. Such smoothers are commonly not in use perhaps because eye movement data processing would take a lot of time for long records. However, A. Tsybakov and V. Doubrovski developed (unpublished report, 1990) a fast algorithm for calculating Huber's estimate in a finite number of steps for the statistical package XploRe (Härdle, 1990). The MATLAB/Octave implementation of this algorithm was tested on eye movements data containing microsaccades with amplitude up to 1°, and showed high efficiency.
Supported by RFBR grant 19-013-00784

Poster 150

Eye tracking during face-to-face interaction: Does speech affect eye-tracking data quality?

G.A. Holleman[1,2], R.S. Hessels[1,2], C. Kemner[1,3] & I.T.C. Hooge[1]

1 *Experimental psychology, Helmholtz Institute, Utrecht University, the Netherlands*

2 *Developmental psychology, Utrecht University, the Netherlands*

3 *Brain Center Rudolf Magnus, University Medical Center Utrecht, the Netherlands*

Background. High-quality eye tracking data is typically obtained when subjects are restrained with a chinrest or headrest while they passively perceive visual stimuli. However, during face-to-face interactions, people may talk, gesture, and move their head, body, and parts of their face. These conditions deviate from optimal eye-tracking conditions and may affect eye-tracking data quality, but have hitherto not been investigated or quantified. In this study, we are interested in how speech affects eye tracking data quality during face-to-face interaction.

Method. In a dual eye-tracking setup with live video-connection, we measured gaze of two subjects simultaneously. Subjects (n=20) were given several tasks to carry out in pairs: speaking, listening, turn-taking, staring, and chewing candy. We computed data loss (DL) and variable error (RMS-s2s deviation) of the eye-tracking data for each task.

Results. Data loss and variable error were highest when subjects were speaking (DL=31.6%, RMS=1.9°) and when subjects took turns speaking/listening (DL=33.1%, RMS=1.8°), and lowest when subjects were only listening (DL=12.3%, RMS=1.5°), staring (DL=17.9%, RMS=0.7°), or chewing candy (DL=16.3%, RMS=1.6°).

Conclusion. Researchers interested in the study of face perception during social interaction need to be aware that speech and turn-taking behavior may decrease eye tracking data quality, which affects eye-tracking data analysis.

Poster 151

Unfold: a toolbox to analyze combined EEG and eye-tracking data with non-linear deconvolution models

Ehinger, Benedikt V; Dimigen, Olaf
Donders Institute for Brain, Cognition and Behaviour, Radboud University; Department of Psychology, Humboldt-Universität zu Berlin

Vision is an active process, but in active tasks like free viewing we cannot experimentally control eye movements, which complicates research on the electrophysiological (EEG) correlates of natural vision. When combining eye tracking and EEG, four fundamental problems exist: (i) the synchronization of data streams, (ii) the removal of ocular artifacts, (iii) the systematically varying temporal overlap between the brain responses evoked by consecutive fixations, (iv) and the numerous and often nonlinear influences of low-level stimulus- and eye movement-properties (like saccade amplitude) on the neural responses (Dimigen et al. 2011). While effective solutions exist for the first two problems, the latter ones, overlap and non-linear confounds, continue to be largely unresolved. We recently published the unfold toolbox (www.unfoldtoolbox.org, biorXiv: https://doi.org/10.1101/360156!), which unifies the linear deconvolution framework (to disentangle overlapping potentials) and non-linear regression (to control for non-linear confounds). Here, we illustrate the advantages of this approach using data from two commonly studied eye-tracking/EEG paradigms: face perception and scene viewing. First, we demonstrate how deconvolution can be used to remove overlapping brain potentials produced by involuntary (micro)saccades in a typical ERP face recognition experiment. Then, we disentangle multiple nonlinear influences of saccade parameters on fixation-related potentials (FRPs) during natural scene viewing. Our results presented here show a principal way to measure reliable fixation-related ERPs during natural vision. The easy-to-use unfold toolbox, including extensive documentation, is open source and freely available.

Poster 152

A new comprehensive Eye-Tracking Test Battery concurrently evaluating the Pupil Labs Glasses and the EyeLink 1000

Groß, Katharina; Ehinger, Benedikt; Ibs, Inga; König, Peter

Institute of Cognitive Science, Osnabrück University; Donders Institute for Brain, Cognition and Behaviour, Radboud University; Institute of Cognitive Science, Osnabrück University; Institute of Cognitive Science, Osnabrück University

Eye-tracking experiments rely heavily on the quality of equipment. Unfortunately, it is often that only the spatial accuracy and precision values are available from the manufacturers. These alone are not sufficient for a reliable evaluation, but different experimental paradigms require a large scope of analysis.
Here, we developed an extensive eye-tracking test battery. In 10 different tasks, we evaluated with 15 subjects the decay of accuracy, fixation and saccade durations, smooth pursuit movements, microsaccade detection, blink detection, pupil dilation, and the influence of yaw and roll head motion. We use a remote EyeLink 1000 (EL) as a reference and benchmark the mobile Pupil Labs glasses (PL).
The average spatial accuracy of 0.57° decayed only slightly over time (EL) or dropped from 0.82° notably (PL). Furthermore, we detected fewer fixations and shorter saccade durations for the PL. Similarly, we found far fewer microsaccades using the Pupil Labs glasses. The pupil diameters differed between eye-trackers on the individual subject level but not on the group level. The influence of head movements was comparable in size, but different in quality as a function of type of head movement.
To conclude, our eye-tracking test battery offers a wide variety of tasks that allow benchmarking the many parameters of interest in typical experimental situations and evaluate whether an eye tracker is suitable for the requirements of a specific experiment.
We recently published data and a preprint (https://doi.org/10.1101/536243).

Poster 153

An error estimation of 3D-eye-models in eye-tracking protocols – undetected eye movements and the use of standards for cardinal points

Peter Wagner, Klaus Ehrmann, Fabian Conrad, Ravi Bakaraju
Brien Holden Vision Institute, Australia
University of New South Wales, Australia
p.wagner@brienholdenvision.org

Background / Purpose
Virtual and augmented reality applications rely on ocular vergence measurements to determine fixation point location and distance in 3D space. 3D-eye-models in eye tracking achieve lateral accuracies of 1.5-2° on a 2D plain, which is insufficient for clinically meaningful vergence measurements.
Methods
We compared 3D-eye-models with optometric schematic eyes with regard to location of cardinal points (the pupil- and eye rotation- center); their movements, and resulting effects during detection. We then modelled identified disparities to estimate influence on gaze and vergence measurements.
Results
Blink related retraction into the orbita account for up to 2° in monocular gaze errors, resulting in twice the error for vergence measurement. Common absolute pupil center shifts during constriction of 0.1 mm relate to 0.5° in gaze or 1° vergence error, respectively. 3D eye modeling assumes a pupil rotation sphere to calculate gaze direction. Dislocation between the true and assumed sphere center has multiple implications on gaze and vergence measurements (Figure 1).

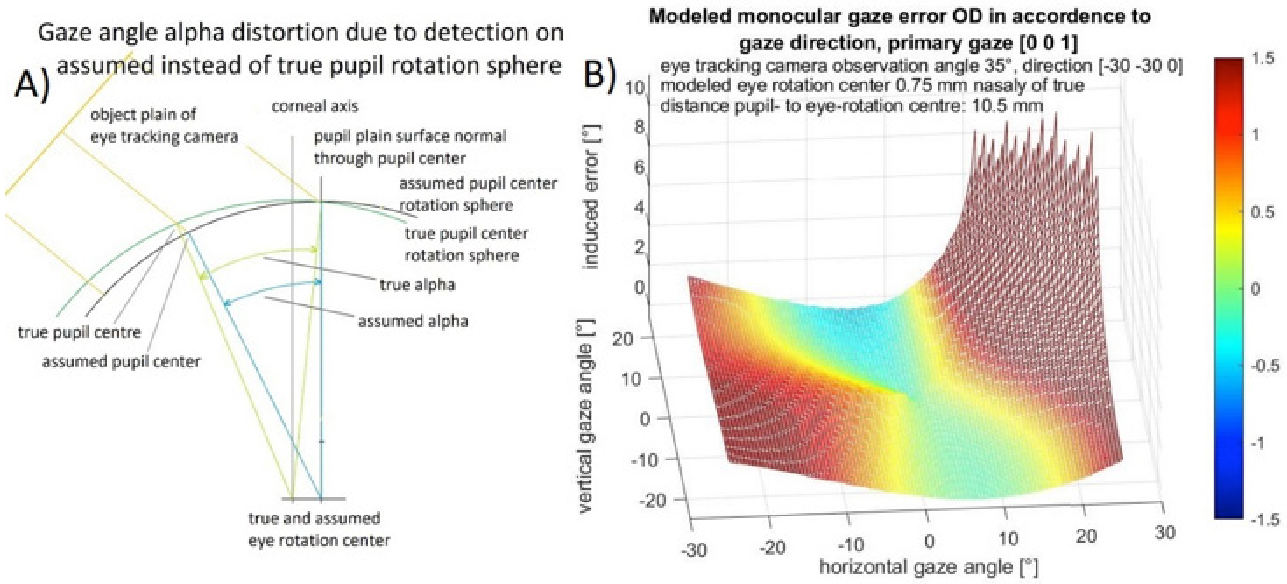

Figure 1 A) Principle of distortion of gaze angle B) influence on field of gaze right eye (OD).

Empirical observation of recordings support the effect of blink related retraction and pupil shift during constriction on vergence measurements.
Discussion
To reliably measure vergence and derive focus point distances, undesired blink-related eye movements need to be compensated for. Furthermore, customized determination of cardinal points in eye tracking protocols will improve the accuracy of such measurements.

Poster 154

Predictive signals in eye dynamics?

Miriam Reiner
Technion, Israel Inst. of Technology, & Mantis-Vision, Israel

Level of difficulty might be perceived through bottom up processes, i.e. conditioned by actual performance of the task, or top down processes, conditioned by expectations. The question we ask here is about timing - when is the recognition process of an expectation happen? Specifically, is the 'expectation' early enough to generate a signal before the actual task performance, suggesting that a potential predictive signal is not related to the actual difficulty level of the task but rather to memories associated with the context, that might, or might not be related to the task difficulty. This signal might change due to surprise of unexpected easy/difficult performance (Braem et al 2015). We hypothesized that the arousal response might also reflect an unexpected event, especially a threat e.g. threatening events during social threat, or driving. When a threat occurs, the brain-Hebbian calculations of visual cues are used to calculate probabilities of a threat, such as social violence or an accident. We hypothesized that this might result in an activation of the autonomous arousal system and ran an experiment in a simulator to test this hypothesis. Results show a consistent signal extracted from multiple factors of eye dynamics, using machine learning and visual processing. We further show that this signal is consistent, across same-category events, and across participants.

Braem, S., Coenen, E., Bombeke, K., Van Bochove, M. E., & Notebaert, W. (2015). Open your eyes for prediction errors. *Cognitive, Affective, & Behavioral Neuroscience, 15*(2), 374-380.

Poster 155

Integrating GlassesViewer and GazeCode: an open-source data analysis alternative for mobile eye-tracking.

Jeroen S. Benjamins[1,2], Roy S. Hessels[1,3], Diederick C. Niehorster[4,5]

1 *Experimental Psychology, Helmholtz Institute, Utrecht University, Utrecht, the Netherlands*
2 *Social, Health and Organizational Psychology, Utrecht University, Utrecht, the Netherlands*
3 *Developmental Psychology, Utrecht University, Utrecht, the Netherlands*
4 *Lund University Humanities Laboratory, Lund University, Lund, Sweden*
5 *Department of Psychology, Lund University, Lund, Sweden*

Mobile eye trackers often come with manufacturer software for data analysis, for instance Tobii Pro Lab for use with Tobii Pro Glasses 2. Tobii Pro Lab, however, is costly and a closed system, thus making it hard to expand or adjust to a researcher's data-analysis needs. Here, we present an alternative that combines the open-source packages GlassesViewer and GazeCode.

GlassesViewer automatically parses data files directly from the SD card of the Tobii Pro Glasses and displays azimuth, elevation, gaze velocity, pupil diameter, gyroscope and accelerometer data in a GUI alongside scene- and optionally eye-camera video images. Data can then be inspected for data quality, further annotated (manually or with classifier algorithms) and stored along with the original data. GazeCode takes the annotations from GlassesViewer, presents them in an interface for manual mapping to the visual stimulus and exports to text files for further analysis.

To demonstrate the flexibility and effectiveness of the integrated software, a simple experiment was performed. A subject looked for red pins on a message board while wearing the Tobii Pro Glasses 2. Using a custom button box, the subject indicated when a pin was found, the timestamps of which are fed straight into the eye-tracker data. Using GlassesViewer and GazeCode, fixations were classified in the eye-tracking data and manually mapped to the visual stimulus. The output of GazeCode can be used for further (statistical) analysis. Combining GlassesViewer and GazeCode offers an easy-to-use, open-source alternative to manufacturer software for mobile eye-tracking data analysis.

Poster 156

EMDAS: Eye Movement Data Analysis Service

Patrick Hynes *(phynes@cs.nuim.ie)* **and Ronan Reilly** ***(ronan@cs.nuim.ie)***
Department of Computer Science, Maynooth University, Ireland

Once an eye tracking experiment is complete, the task to review, clean and analyse the collected data begins. A researcher, depending on the hardware used to perform the experiment, may use a variety of tools to take their collected data and transform it into a working data set from which results relating to their experimental hypotheses can be obtained. This process can be time-consuming and complicated. EMDAS aims to improve how this process is carried out by leveraging the power of cloud computing. The platform allows data to be ingested, cleaned, analysed, transformed and allows results from the data to be obtained to either support or refute whatever experimental objective had been undertaken. Data from experiments is uploaded to the cloud-based platform, taking advantage of robust and highly available cloud storage services. Transformation of data facilitated using on-demand functions, storing the transformed artefact for analysis. Collected data is visualised within the browser, and can be cleaned in a manual or automated fashion. Areas of interest around words read by subjects are automatically determined using image recognition API's and presented alongside collected eye movement data. For data analysis, the platform provides a stable, browser-based, environment for analysis via platform containerisation technologies. Common statistical software is provided for data analysis. Results obtained from the statistical analysis can be stored securely using version control and downloaded for further use. By leveraging cloud computing, the platform provides a location for all elements of data analysis of an experiment.

Poster 157

Introducing GEETUP, a large database of mobile eye tracking in a urban environment

Valsecchi, Matteo; Akbarinia, Arash; Gegenfurtner, Karl

Justus-Liebig-Universität Giessen, Abteilung allgemeine Psychologie; Justus-Liebig-Universität Giessen, Abteilung allgemeine Psychologie; Justus-Liebig-Universität Giessen, Abteilung allgemeine Psychologie

The database consists of a large set of egocentric videos and eyetracking data captured as observers walked along two almost completely separate paths in the urban environment of Giessen (Germany), hence GEETUP (Giessen Egocentric Eye Tracking in Urban Paths) database. The data were acquired using a TOBII 2 mobile glasses system (eye tracking at 100 Hz, video recorded at 25 fps). 27 observers walked on the first route (3.6 km) and 23 observers walked on the second route (3.2 km), 15 of the observers did both routes.
The database was manually annotated to discard all recording episodes not pertaining to walking (consulting map, standing at traffic light etc.) and major problems with video recording (e.g. glare from direct sunlight). In total, out of the 50 recordings we extracted 27.2 hours of valid data, i.e. on average 32.6 minutes per recording. These are pre-segmented in 5500 smaller videos with a median duration of 14.7s.
General heatmaps confirm the presence of a very strong central bias in head-centered gaze position. Preliminary analyses using SP-Tool (Agtzidis, Startsev & Dorr ETRA 2016) identified 231.000 saccades.
The predominant direction of the saccades appears to be oblique, particularly on the NW-SE axis. A qualitative inspection of the saccadic events in the videos suggests that this is due to saccades following the dominant orientation of the walkway boundary.
We propose this database both as a testbed for egocentric saliency models and for research on the occurrence of smooth pursuit in an ecologically valid setting.

Poster 158

Guessing the Line Currently Being Read: Approaches for Offline Recalibration of Eye-tracking Reading Data

Borges Gamboa, John Cristian; Fernandez, Leigh; Allen, Shanley
University of Kaiserslautern

Eye-tracking data often contain noise due to poor calibration or tolow accuracy of the tracker. This becomes particularly problematic in studies for which participants are scarce and repeating the experiment is not an option. Procedures exist for the offline recalibration of eye-movement data (e.g., Vadillo et al., 2015), but they often rely on the identification of task-specific "Required Fixation Locations" or "Probable Fixation Locations" (cf. Zhang & Hornof, 2014). Approaches for guessing the line of text currently being fixated on have been proposed; however they either expect the reader to follow the order of the text sequentially (Yamaya et al., 2015), do not consider regressions into a previous line of the text (Sanches et al., 2016), or assume that the calibration error is independent of the horizontal position of the fixation (Martinez-Gomez et al., 2012). All of these assumptions are problematic for data in typical psycholinguistic eye-tracking studies. We present three new/alternative methods for improving the quality of the data by assigning fixations to a given line and moving them to a corrected position. The approaches are evaluated on existing data from a study where 19.29% trials were discarded due to not fulfilling certain quality criteria and 7.61% of fixations were not assigned to any word. Analyses using the proposed three methods show that, instead, 17.29%, 17.07% and 14.36% trials would be discarded by same criteria, and 4.28%, 3.75% and 5.63% fixations would be assigned to no words. We also developed a simple visualization tool to compare the different methods.

References

Martinez-Gomez, P., Chen, C., Hara, T., Kano, Y., & Aizawa, A. (2012, February). Image registration for text-gaze alignment. In Proceedings of the 2012 *ACM International Conference on Intelligent User Interfaces* (pp. 257-260). ACM.

Sanches, C. L., Augereau, O., & Kise, K. (2016). Vertical error correction of eye trackers in nonrestrictive reading condition. *IPSJ Transactions on Computer Vision and Applications, 8*(1), 7.

Vadillo, M. A., Street, C. N., Beesley, T., & Shanks, D. R. (2015). A simple algorithm for the offline recalibration of eye-tracking data through best-fitting linear transformation. *Behavior Research Methods, 47*(4), 1365-1376.

Yamaya, A., Topić, G., Martínez-Gómez, P., & Aizawa, A. (2017, June). Dynamic-programming–based method for fixation-to-word mapping. *In International Conference on Intelligent Decision Technologies* (pp. 649-659). Springer, Cham.

Zhang, Y., & Hornof, A. J. (2014, March). Easy post-hoc spatial recalibration of eye tracking data. In *Proceedings of the symposium on eye tracking research and applications* (pp. 95-98). ACM.

Poster 159

Top-down control of saccades requires inhibiting suddenly appearing stimuli

Christian Wolf & Markus Lappe
Westfälische Wilhelms-Universität Münster

Humans scan their visual environment using saccade eye movements. Where we look is influenced by bottom-up factors like stimulus salience and top-down factors like value information. For reactive saccades in response to suddenly appearing stimuli, it has been shown that short-latency saccades are biased towards salience and that top-down control increases with increasing latency (Schütz, Trommershäuser, & Gegenfurtner, 2012). Here we investigate whether this transition from bottom-up to top-down control is determined by inhibition of suddenly appearing stimuli.
Participants made consecutive saccades to three fixation crosses and a vertical bar consisting of a highly salient and a low salient region. The bar was foveated last and appeared left or right of the last fixation cross with the salient region pointing upwards or downwards. In selected conditions participants received a monetary reward for successfully looking at the low salient region.
Without reward, saccade endpoints were always biased towards the salient region. With reward and when the bar was displayed continuously from trial beginning, saccades were not biased towards salience. However, when the vertical bar appeared after successfully foveating the last fixation cross, short-latency saccades were biased towards salience. This was also true if the outline of the rewarded region was displayed from trial beginning, but the salient region suddenly appeared above or below. In this last condition, while there was enough time for saccade planning to integrate value information, there was no possibility to suppress salience. Altogether, these findings highlight the importance of inhibition for top-down eye movement control.

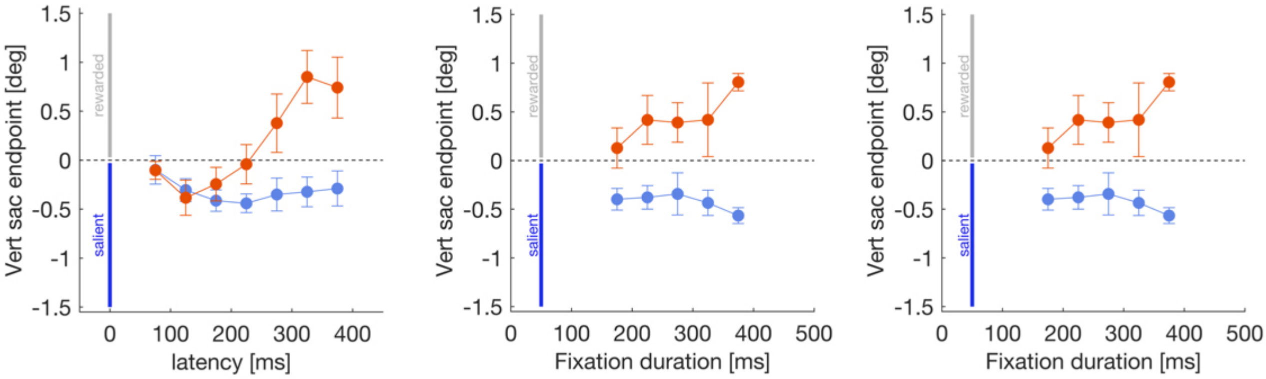

Fig. 1. Sudden onset condition. Vertical saccade endpoints as a function of saccade latency when participants received a reward (orange) or not (blue). Latency values denote saccade onsets relative to onset of the vertical bar. Data were collapsed so that positive vertical endpoints denote saccades towards the rewarded region. Error bars represent 95% confidence intervals.
Fig. 2. Continuous condition. Vertical saccade endpoints with reward (orange) and without reward (blue) as a function of the fixation duration before the bar was foveated. Error bars denote 95% confidence intervals.
Fig. 3. Reward outline condition. Vertical saccade endpoints with reward (orange) and without reward (blue) as a function of the temporal difference between saccade onset and target onset (t = 0). The outline of the rewarded region was displayed throughout the trial and could be foveated before target onset. In half of the trials, the vertical bar was vertically centered relative to the last fixation cross (saturated colors), in the remaining trials, the salient part was flipped to the other side (faint colors). Error bars denote 95% confidence intervals of between-participant variability.

Poster 160

Does number processing modulate kinematic parameters of spontaneous saccadic eye movements?

Doré-Mazars[1], K. & Pressigout[1], A. & Leroy[1], L.J. (soumis le 22/04/2019)

[1]*Laboratoire Vision Action Cognition, EA 7326, Institut de Psychologie, INC, Université paris Descartes, Sorbonne Paris Cité, Boulogne-Billancourt, France*

Numerous studies have shown that number processing affects response times and more recent studies have found that it could also impact the kinematic parameters of certain movements. Thus, both response times and amplitudes of swiping movements are modulated by number magnitude (Fischer, Fischer, Huber, Strau[illegible] & Moeller, 2018). The few studies on saccadic eye movements did not provide clear-cut conclusions. For instance, spatial-numerical associations (i.e. SNARC effect; Dehaene, Bossini & Giraux, 1993) have been found to modulate ocular drifts (Myachykov, Ellis, Cangelosi & Fischer, 2016) but not amplitudes of memory-guided saccades (Fischer, Warlop, Hill & Fias, 2004). Here we used a protocol similar to Fischer et al.'s (2018) to involve spontaneous saccades without visual target in order to optimize the emergence of a numerical effect. 24 participants were asked to perform a classification task by moving their eyes toward the left or the right side of an empty screen as response to the digit magnitude centrally presented. As expected, spatial-numerical associations were found on saccade latencies, reflected by faster responses congruent with the mental number line. In addition, a numerical distance effect was observed on latencies and more critically on amplitudes of the primary saccades: participants were faster and performed longer saccades for larger numerical distance to the standard 5. Overall, our study highlights interferences between number processing and saccadic programming beyond time responses.

Poster 161

The role of microsaccade frequency in peripheral vision

Lilit G. Dulyan, W. Joseph MacInnes, Igor S. Utochkin
National Research University Higher School of Economics, Russia

We proposed that microsaccades could be involved in the enhancement of spatial vision not only in the central (Rucci, Iovin, Poletti, & Santini, 2007) but also in the peripheral vision. It was hypothesized that in order to detect the stimulus edges in the periphery with large receptive fields it is necessary to move frequently the image projection such as the central part of the receptive fields will lie along the dark-light edge of the stimulus. Microsaccades could provide these movements. To test this idea, we asked our observers to report the orientation of a black Landolt ring at 7º eccentricity surrounded by (1) two white flankers or (2) two black flankers. The second condition should cause a stronger crowding effect than the first one due to increased target-flanker similarity (Kooi, Toet, Tripathy, & Levi, 1994). The QUEST procedure was used to estimate the critical spacing for crowding (Watson & Pelli, 1983). The ring size and prior intensity were calculated based on Song, Levi, & Pelli (2014). In both stages of the experiment, participants were asked to recognize the orientation of a central Landolt ring. The logistic regression analysis confirmed that the second condition causes greater crowding effect ($\chi 2$ (1) = 15.06, p = 0.000196). Although the trial type did not affect the overall frequency of microsaccades in the linear mixed effects model ($\chi 2$ (1) = 1.546, p = 0.214), the density distribution of microsaccades was elevated in the complex condition at the beginning of trials.

Poster 162

Rendering the invisible visible during microsaccades

Jan-Nikolas Klanke[1,2], Sven Ohl[1], & Martin Rolfs[1,2,3]

1 Department of Psychology, Humboldt-Universität zu Berlin, Germany
2 Berlin School of Mind and Brain, Humboldt-Universität zu Berlin, Germany
3 Bernstein Center for Computational Neuroscience Berlin, Berlin, Germany

During prolonged fixations, microsaccades occur at a frequency of 1–2 Hz, introducing brief discontinuities in the retinal image. Previous studies have shown that these discontinuities cause a reduction in visual sensitivity (microsaccadic suppression). Here we provide evidence that visual sensitivity remains intact during microsaccades. We devised a new paradigm in which a visual stimulus is moving rapidly (and, thus, invisibly) during fixation, but briefly stabilized on the retina during microsaccades when the velocity and direction of the eyes match the stimulus direction. We instructed participants to detect the stimulus and report the timing of its onset (i.e., the time on a clock face) in two different conditions. In the *active-microsaccade condition*, we displayed a stimulus—a vertical Gabor with a high-velocity phase shift—and assessed its visibility during microsaccades. Performance in this condition was compared to a *simulated-microsaccade condition* that caused comparable retinal input without microsaccade generation. Participants reliably reported the stimulus in the simulated-microsaccade condition. In the active-microsaccade condition, detection depended on microsaccade generation: Participants were most sensitive if the direction of a microsaccade matched the direction of the stimulus' phase shift. Temporal judgments of stimulus onset were comparable in both conditions, suggesting that the same retinal cues gave rise to detection. Our findings are in line with those of Deubel and Elsner (1986), who showed that microsaccades aid flicker detection. Indeed, visual perception remains largely unperturbed during microsaccades, provided the input is rendered static on the retina. Thus, microsaccade execution does not necessarily reduce visual sensitivity.

Deubel, H., & Elsner, T. (1986). Threshold perception and saccadic eye movements. *Biological Cybernetics*, 54(6), 351-358.

Poster 163

Perceptual segmentation of auditory streams revealed by involuntary eye movements during fixation

Rizzuto, Vincenzo; Kadosh, Oren; Bonneh, Yoram

School of Optometry and Vision Science, Bar-Ilan University; School of Optometry and Vision Science, Bar-Ilan University; School of Optometry and Vision Science, Bar-Ilan University

The involuntary eye movements during fixation (microsaccades, spontaneous eye-blinks) are known to be modulated by transient physical stimuli (oculomotor inhibition) as well as to affect visual processing, such as by counteracting fading or inducing perceptual alternations.

Here we asked whether these movements are modulated by the perceived or the physical stimuli in bi-stable auditory perception and whether they mark points of perceptual segmentation.

Observers (n=16) fixated and listened passively to periods of ~40s bi-stable auditory stimulation, with the classical ABA_ABA_ sequence (A=500Hz, B=1400Hz) presented at 2Hz cycle, while their eyes tracked. They were asked to voluntarily attend to the low, high or both tones, resulting in 3 different perceptual states in different runs.

We found that the observers' eyes were entrained to the frequency of the sound pattern and that microsaccades occured at different times or "perceptual positions", based on which perceptual stream the subject perceived as reflected by a difference in the rate modulation pattern, as well as average relative microsaccade times.

Moreover, spectral analysis of the total raw eye drift (including microsaccades) revealed significantly higher 2Hz and 4Hz power when perceiving the slow (high) and fast (low tone) streams respectively. We obtained a similar pattern of results when observers were free to perceive (shorter 6s trials) and with another type of stimulus (cycle of six tones).

The results suggest that the eyes are entangled by the perceptual (not physical) pattern and that microsaccades tend to occur in points of perceptual segmentation, possibly involved in a "cognitive punctuation" mechanism.

Poster 164

Interactions between microsaccades and quick-phases of torsional optokinetic nystagmus

Otero-Millan Jorge[1], Sadeghpour Shirin[1]
1 Johns Hopkins University, Department of Neurology

Many studies have characterized the eye movements that occur during visual fixation, including microsaccades. In most cases, however, only horizontal and vertical components of eye movements have been recorded and analyzed. Thus, little is known about the properties of the torsional component of microsaccades, in which the eye rotates around the line of sight. Here, we take advantage of newly developed software and hardware to record eye movements binocularly in three dimensions at 250Hz during fixation and during torsional optokinetic stimulus. We found that the average amplitude of the torsional component of microsaccades during fixation was 0.34 ± 0.07 degrees with velocities following a main sequence with a slope that was comparable to the vertical component but slower than the horizontal component. In the presence of an optokinetic stimulus the frequency of saccades/quick-phases increased, with torsional components growing larger with higher velocities. However, the horizontal and vertical components did not change with increase in velocity. Across subjects, we found that the rate of quick-phases occurring during torsional optokinetic stimulation was not correlated with the rate of microsaccades during fixation at a static stimulus. Our results show the feasibility of recording torsional eye movements using video eye tracking in a desktop mounted setup. We also show that while the torsional and vertical components are coupled during optokinetic stimulation the horizontal component behaves independently from them.

Poster 165

Eye-movement control in the Visual World Paradigm

Kromina, Anastasia; Lopukhina, Anastasiya; Laurinavichyute, Anna
Center for Language and Brain, National Research University Higher School of Economics, Moscow, Russia; Center for Language and Brain, National Research University Higher School of Economics, Moscow, Russia; Center for Language and Brain, National Research University Higher School of Economics, Moscow, Russia; University of Potsdam, Potsdam, Germany

One of the methods of experimental research of speech comprehension is the “Visual World Paradigm”. In a typical setup participants see several images and hear related linguistic input, while their eye movements are being recorded. The assumption behind the paradigm is that eye movements to the object that currently undergoes linguistic processing are automatic, but this assumption has not been tested directly. We conducted two experiments (with 40 participants each) that tested the automaticity of language-mediated eye movements by probing whether they could be canceled by volitional control. Each experiment had a special condition: in the eye-movement control condition, participants were asked to not look at the object that is currently being referred to. In the free viewing condition, participants had a classical ‘look and listen’ task. Both experiments included in each condition the same 32 short stories, each with 10 critical words. In both experiments participants had to answer comprehension questions after each story to ensure that they indeed processed the experimental materials.
Probability of fixating the target image is lower in the eye-movement control condition and lower for pronouns than for nouns. We also found an interaction: participants were less likely to fixate a noun’s referent in the eye-movement control than in the free viewing condition, which was not true for pronouns.
When participants fixated on the target image, the duration was greater in the eye-movement control condition (probably due to the impact of short fixations in the free viewing condition).

Poster 166

Saccadic motor command adapts to post-saccadic target representation in pre-saccadic coordinates

Jana Masselink & Markus Lappe
University of Muenster, Germany

The oculomotor system is hypometric: A saccade typically falls short 5-10% of the target and if an intra-saccadic target step is introduced, the amplitude adapts to a steady stage of 40-70% of the step size. Saccadic amplitudes and its adaptation are usually explained by minimisation of visual error or visual prediction error - in the latter assuming that in baseline and adapted saccades, the predicted visual error matches the actual post-saccadic visual error. Here we measured the predicted visual error by probing target-off saccades before, during and after adaptation, including pre- and post-saccadic localisation of a flash presented during fixation / shortly after target appearance. Adaptation was induced first with constant step size and second with constant visual error. We fitted three state space models which differed in the error that drives learning: visual error, visual prediction error and a motor prediction error computed as the error between the post-saccadic target representation in pre-saccadic coordinates and the saccadic motor command. Three gains were free to adapt: a visual gain to visually represent target distance, a motor gain to transform visual representations into motor commands and a forward model gain to simulate the visual consequences of motor commands. We show that minimisation of motor prediction error with plasticity in all three gains best explains saccadic amplitudes and localisations before, during and after adaptation. Our results suggest a new perspective on inter-saccadic prediction error computation for oculomotor control and adaptation.

Poster 167

Effects of motivation and dopamine on action selection and decision making

Hirschbichler, Stephanie; Ibánez, Jaime; Rothwell, John; Manohar, Sanjay

UCL Queen Square Institute of Neurology, Department of Clinical and Motor Neurosciences; UCL Queen Square Institute of Neurology, Department of Clinical and Motor Neurosciences; UCL Queen Square Institute of Neurology, Department of Clinical and Motor Neurosciences; UCL Queen Square Institute of Neurology, Department of Clinical and Motor Neurosciences, Nuffield Department of Clinical Neurosciences, University of Oxford

Objective

Studying eye movements can provide important insights into the pathophysiology of neurological disorders. Two experimental paradigms were used to quantify the differential effects of motivation by reward and loss and alterations of dopamine levels by drug manipulation on motor control and decision making.

Background

The speed-accuracy trade-off can be overcome when motivation simultaneously invigorates movements and improves response precision (Manohar et al. 2015). Dopamine may be a key neuromodulator in such motivational effects. We measured effects of monetary incentives (reward, loss) and drugs (Haloperidol, Levodopa) on oculomotor performance.

Methods

13 healthy controls performed two saccadic tasks recorded by an infrared eye tracker. Task 1: When we have to choose between a larger number of options, our responses become slower (Hick 1952) ; healthy volunteers were asked to make a saccade to one target indicated by an arrow, while monetary incentives and the number of possible targets varied. Task 2: we here studied how reward improves performance avoiding an early distractor in a prosaccadic task.

Both tasks involved three incentive levels indicated by an auditory cue before the start of each saccade. Reward was calculated as a fraction of the maximum depending on the performance (accuracy and speed).

Results:

We observed improved performance on incentivized trials (more so for reward than penalty) looking at peak velocity and reaction times. Higher dopamine levels led to faster simple reaction times but not choice reaction times and a trend towards higher reward sensitivity was observed. Data collection is currently ongoing.

References

Hick, WE. 1952. "On the Rate of Gain of Information in Children." *Quarterly Journal of Experimental Psychology 4* (1): 11–26. https://doi.org/10.1016/0022-0965(78)90002-4.

Manohar, Sanjay G., Trevor T.-J. Chong, Matthew A.J. Apps, Amit Batla, Maria Stamelou, Paul R. Jarman, Kailash P. Bhatia, and Masud Husain. 2015. "Reward Pays the Cost of Noise Reduction in Motor and Cognitive Control." *Current Biology 25* (13): 1707–16. https://doi.org/10.1016/j.cub.2015.05.038.

Poster 168

Age effects on task-related saccadic eye movements

Miroslava Stefanova, Nadejda Bocheva, Bilyana Genova, Simeon Stefanov
Institute of Neurobiology, Bulgarian Academy of Science; Institute of Neurobiology, Bulgarian Academy of Science; Institute of Neurobiology, Bulgarian Academy of Science; Institute of Neurobiology, Bulgarian Academy of Science

Recently several studies have shown that even though saccadic eye movements are highly stereotyped, their characteristics change depending on task. In the present study, we aimed to characterize the changes in saccade metrics in tasks of varying difficulty that pose different perceptual demands on performance. Four different conditions were used. In all of them, the task of the observers was to determine whether the shifted position of radial patterns was to the left or the right of the screen center and to make a saccade to its perceived center. The center was defined either by the orientation of dot pairs in a Glass pattern, by the orientation of pairs in a dynamic pattern, by the trajectory of moving dots and by both the trajectory and orientation of dot pairs. The subjects aged 23 to 72 years were divided in two age groups. The results show that in all experimental conditions the elderly group had longer saccade duration and lower peak velocity than the younger group. The differences in the experimental conditions significantly affected the saccade metrics for both age groups though the effects were not the same. The experimental conditions had different effects on the peak velocity and saccade duration and on their relationship with the saccade amplitude. The change in saccade characteristics is not correlated with the accuracy of performance or with the response time. The link between the saccade metrics and the underlying cognitive processes will be discussed.

This work was supported by the National Science Fund DN02/3/2016

Poster 169

Retention of saccadic adaptation induced by reinforcement learning

Céline Paeye

*Laboratoire Vision Action Cognition, Institut de Psychologie, Université de Paris,
71 avenue Edouard Vaillant, 92100 Boulogne-Billancourt, FRANCE*

We have previously shown that saccadic amplitude can be adapted via reinforcement learning and not only via the conventional double-step paradigm (Madelain, Paeye & Wallman, 2011). To disentangle post-saccadic visual error (known to induce saccadic adaptation) from reinforcement, we extinguished the target during saccades and provided rewarding tones whenever saccades met specific amplitude criteria. Such auditory reinforcement induced changes in saccadic amplitude similar to the changes obtained using the double-step paradigm. We proposed that saccadic adaptation might involve a general learning mechanism in which saccades are reinforced by the clear vision of the target.
The goal of the present study is to examine the retention of reinforced adaptation. Saccadic amplitude was decreased (backward adaptation) or increased (forward adaptation) using our reinforcement paradigm. Five days after the last reinforcement session, the amount of backward adaptation (n=7) was still significant (-20.1%, SEM=4.2, retention rate: 75.4%) and forward adaptation (n=4) kept increasing (27.3%, SEM=11.5, retention rate: 145%). Preliminary data also showed retention 12 and 19 days after the last reinforcement session. Overall, the retention was stronger than in conventional adaptation.
These long-lasting effects of reinforced adaptation are consistent with previous findings indicating that the effects of conventional backward adaptation can still be observed two months later (Wang et al., 2012). This provides further argument for the involvement of a general learning mechanism in saccadic adaptation. Presumably, auditory consequences of saccades provided during reinforcement sessions increased context specificity, crucial for sensorimotor adaptation.

Poster 170

Adaptation of voluntary saccades in young children

Christelle Lemoine-Lardennois, Anne Hillairet de Boisferon, Wissam Salem-Cherif, Karine Doré-Mazars, Nadia Alahyane

Université Paris Descartes, Institut de Psychologie, Laboratoire Vision Action Cognition, Boulogne-Billancourt, France

Sensorimotor adaptation is fundamental to maintain accurate saccadic eye movements for optimal perception and interaction with the environment throughout life. We previously showed that adaptation mechanisms for reactive (externally-triggered) saccades (RS) are in place early during development (Lemoine-Lardennois et al., 2016). Nevertheless, this cannot be automatically extended to voluntary (internally-guided) saccades (VS). Indeed, adaptation mechanisms are partially separate for RS and VS in adults (Pélisson et al., 2010), and brain regions involved in VS adaptation also show late maturation (e.g., Lenroot & Gieed, 2006).

Here we adapted our previous protocol to elicit VS, without verbal instruction, in toddlers (age < 3.5 years) and adults, using an overlap procedure. To prompt a decision, two identical peripheral stimuli appeared simultaneously at 10° eccentricity from a fixation point (140 trials). During the saccade toward the target, it systematically stepped in the direction opposite to the saccade to induce an adaptive decrease in saccade amplitude.

Preliminary results showed that toddlers were able to select and direct their gaze toward one target without any explicit task instruction. Analysis of baseline performance revealed longer saccade latency (M = 415ms, SD = 112ms) and lower accuracy (M = 9.2°, SD = 0.4°) in toddlers compared to adults (M = 205ms, SD = 111ms; M = 9.6°, SD = 0.6°). Crucially, both adult and toddler groups exhibited an adaptive decrease in saccade amplitude, with similar amount of adaptation.

Overall, our results suggest that the neural mechanisms underlying VS adaptation are in place early during development.

Poster 171

Time course of saccade programming during a saccadic choice task by deconvolution of EEG evoked potentials

Anne Guérin-Dugué[1], Léa Entzmann[2], Nathalie Guyader[1]

1 *Univ. Grenoble Alpes, CNRS, Grenoble INP*, GIPSA-lab, 38000 Grenoble, France*
** Institute of Engineering Univ. Grenoble Alpes*

2 *Univ. Grenoble Alpes, Univ. Savoie Mont Blanc, CNRS, LPNC, 38000 Grenoble, France*

The “saccadic choice task” is well-adapted to study the saccadic decision making. During this task, participants have to perform a saccade as fast as possible toward a target (i.e. a face) displayed simultaneously with a distractor (i.e. a vehicle). This paradigm allows to measure the time needed to detect a specific target and to program and execute a saccade. Previous experiments show extremely fast saccades toward faces. By recording both eye movements and electroencephalographic signals, the time course of saccadic decision making can be studied. Nevertheless, the concern is to isolate the evoked potentials to characterize the different cognitive processes which are closely intertwined. Recently, a methodology based on the “General Linear Model” has been successfully proposed to deconvolve overlapped potentials. The experiment was performed with 26 participants on 120 trials for each target condition (Face vs Vehicle). Different potentials were discriminated, as the evoked potential at the image onset, the first pre-saccadic component related to the saccade programming, the first saccadic response related to the saccade execution, the first lambda response related to the early visual processing, and the error potential in case of error which implies programming and execution of a next corrective saccade.

Poster 172

Accurate internal representation of eye position in saccade planning

Lau, Wee Kiat ; Maus, Gerrit W.; Collins, Therese
Nanyang Technological University; Nanyang Technological University; Université Paris Descartes

Fixation position changes slightly after each blink (Lau & Maus, 2019). We investigated whether these changes affect subsequent saccades. We tested if the oculomotor system uses an internal representation of eye position to plan a saccade. Naïve participants (N = 12) made 10° visually-guided (VG) and memory-guided (MG) saccades to a dot target presented to the left or right of fixation. Participants blinked once (blink) or remained fixated (no-blink) before an auditory cue instructed them to saccade to the target. We hypothesized that if participants had access to an eye position signal at the onset of their saccade, blink-induced position shifts should be corrected for. The alternative hypothesis was that without such an internal eye position signal, blink-induced position shifts should correlate with landing positions. This was not the case either in VG or MG saccades. Saccades started more forward from fixation for MG than VG saccades and landed more backward of the target for MG than VG saccades. Blinking did not contribute to these positional differences. Instead, blinks enlarged both saccade amplitudes. MG amplitudes were also smaller than VG amplitudes. We found no correlation between starting and landing errors across saccades. Hence, start position changes did not influence saccade landing errors. Our results suggest that to plan accurate saccades, the oculomotor system uses an internal representation of eye position that is updated after each blink. Although blinking was introduced to increase eye position changes, it did not influence saccade starting nor landing positions.

References

Lau, W. K., & Maus, G. W. (2019). Directional biases for blink adaptation in voluntary and reflexive eye blinks. *Journal of vision, 19*(3), 13-13.

Poster 173

Formation of world-centered perception of intra-saccadic motion streaks

Richard Schweitzer[1,2,3], Tamara Watson[4], Tarryn Balsdon[5,6], & Martin Rolfs[1,2]

1 *Bernstein Center for Computational Neuroscience Berlin, Berlin, Germany*
2 *Department of Psychology, Humboldt-Universität zu Berlin, Berlin, Germany*
3 *Berlin School of Mind and Brain, Humboldt-Universität zu Berlin, Berlin, Germany*
4 *School of Social Sciences and Psychology, Western Sydney University, Sydney, Australia*
5 *Laboratoire des systèmes perceptifs, Département d'études cognitives, École normale supérieure, PSL University, CNRS, Paris, France*
6 *Laboratoire de neurosciences cognitives computationelles, Département d'études cognitives, École normale supérieure, PSL University, INSERM, Paris, France*

Saccades give rise to so-called intra-saccadic motion streaks as they shift stimuli across our retinae at dazzling velocities. Although it is widely assumed that a dedicated extra-retinal suppression mechanism eliminates these blurry trajectories from visual processing, they can be readily detected when not masked by pre- and post-saccadic stable retinal images.

Capitalizing on this finding, we developed a paradigm to study the phenomenology of intra-saccadic vision: Using a projection system capable of displaying 1440 frames per second, we briefly presented stimuli moving at high speeds (for 12.5 – 25 milliseconds) during horizontal saccades. Importantly, resulting motion streaks were unpredictable for observers, as stimulus movements could start at nine screen locations and travel in twelve directions. After each saccade, observers reproduced the trajectory of the motion streak on the screen using a computer mouse, allowing us to compare perceived trajectories to their corresponding retinal and world-centered trajectories, respectively.

Although all stimulus movement occurred strictly while the eyes were moving, perceived trajectories were remarkably more similar to their world-centered than to their retinal trajectories. This effect was especially clear for motion streaks that extended further in space and time. In a second experiment, we corroborated these findings, showing that world-centered perception was robust to manipulations of saccade amplitude and global retinal velocity, which we manipulated by shifting a low spatial frequency noise background in or against the direction of the saccade.

Our results suggest that the visual system must have an accurate representation about eye position, even during saccades.

Poster 174

Saccade-related remapping shapes visual perceptual learning.

Pooya Laamerad, Daniel Guitton & Christopher C. Pack.
Montreal Neurological Institute, McGill University, Montreal, Canada
pooya.laamerad@mcgill.ca

A hallmark of visual expertise is the acquired ability to extract at a glance information that novices can only obtain after careful scrutiny [e.g., Rheingold & Sheridan, 2011]. This expertise has been associated with what psychologists call “chunking”: experts perceive larger patterns in a visual stimulus while novices inspect smaller elements individually. The mechanisms by which observers learn this integration is unknown but data suggest an important role for saccadic eye movements. Many neurons in visual cortex are able to integrate information across discrete retinal locations during saccades; a physiological mechanism called remapping because it manifests as a presaccadic shift of a receptive field across visual space [Duhamel et al. 1992]. There is strong psychophysical evidence that human subjects can use remapping to combine information across specific retinal locations, as would be required for the “chunking” of patterns in the image [e.g., Szinte et al. 2016, 2018]. These considerations led us to hypothesize that remapping facilitates the acquisition of visual expertise. To test this idea, we used a paradigm in which observers made saccades while evaluating a motion stimulus at a fixed location in retinal space. Consistent with previous reports on remapping, our observers integrated visual information across saccades. More importantly, through extensive training, observers acquired the ability to integrate such visual information without making saccades; i.e., during fixation. Because these effects are specific to the retinal locations that are involved in remapping, we suggest that remapping could serve as a mechanism for linking together distinct chunks of visual scenes.

Poster 175

Eye-tracking study on the apology text

Yunju Nam, Hyenyeong Chung, Soojeong Kim, Upyong Hong
Konkuk University

Lewicki & Polin (2012) suggested that an effective apology should consist of six components: Expression of Regret(R), Explanation(E), Acknowledgement of Responsibility(A), Declaration of Repentance(D), Offer of Repair(O), Request for Forgiveness(F), and among them, (A), (O) and (E) were the most effective components(Lewicki et al., 2016). We used Eye-tracker to investigate (1) whether these components are important to Korean too, (2) how can we confirm the effective components of apology during the online text processing and (3) how much attention is paid to unpleasant expressions in the apology.
8 apology texts received a score of 5 or higher on authenticity scale-test were selected. One of these texts is used as a Full condition and six texts were used in one component omitted condition. The other was manipulated in the condition including an unpleasant expression. As a results, Normalized Fixation Time (NFT) in (E)(49,758ms) and (R)(45,449ms) were the longest in the full condition. Moreover, results of the Normalized Fixation Count (NFC) showed that more fixations were occurred in (E)(255), (O)(217) and R(216). When one of factors are absent, similar tendency with NFT was observed in NFC. Additionally if an unpleasant expression appears in (A), NFT in (A) was getting longer(51,350ms) and NFC in (A) was the highest(249).
Our results demonstrated that it is important to give Korean rational information about the events and express emotional sincerity but the former is more critical unlike Lewicki et al.(2016). Also, it is also crucial not to include the expressions that seem to circumvent the responsibility.

Poster 176

Analyzing Japanese English Learners' Online English Research Skills Using Eye Tracking

Caleb Prichard & Andrew Atkins
Okayama University & Kindai University
prichard@okayama-u.ac.jp

Reading on the internet is an essential skill for a great variety of purposes. In the real-world, readers need to sift through and select multiple documents online, and then comprehend, evaluate, and synthesize the content. Nevertheless, most reading research has focused on the comprehension of single text documents, ignoring online research skills.

Survey results have suggested that participants who consider their objective and consider if the content fits their purpose tend to be more proficient readers (Zhang & Wu, 2009). However, research has suggested that second language readers often lack task awareness. Prichard and Atkins (2018) found that Japanese readers of English tended to read the whole text even though two-thirds of the text was irrelevant to their task. Second language research empirically examining strategies for searching multiple online documents is lacking.

Considering this gap in the research, this experiment utilizes eye tracking and other methods to evaluate the research strategies of Japanese university-aged English readers. The study focuses on the degree to which learners consider relevance when they view search engine results and the online articles. The relationship between the reading strategies and task performance is also examined.

The results suggest that the participants more often read task relevant pages. However, 20% of the pages read were not related to the research task, and many students clicked articles and read them without regard to their goal. Post-reading interviews suggested the participants had various reasons for their strategies, but strategic competence is often lacking.

Poster 177

Native vs. non-native processing of English number agreement: Eye-movement evidence on the attraction effect

Hyenyeong Chung, Yunju Nam, Upyong Hong
Konkuk university, Seoul, Korea

This study investigated (1) whether Korean L2 learners of English show the attraction effect observed in the English native speakers, i.e. the reading time in the main verb is longer when the number of the main verb and local noun are mismatched, (2) whether the attraction is modulated by the animacy of the local noun varying the conceptual meaning of the plural noun in Korean, and (3) what stage of processing does this effect appear. We manipulated 64 sentences by the animacy of local noun, the local agreement which means the number agreement between the local noun and the main verb, and the grammaticality ([Table 1]). Eye movements of 19 high-proficient learners were recorded while they read the experimental sentences.
In the first pass reading time reflecting the early stage of processing, there was no difference among the conditions. In the go-past time, the main effect of local agreement was marginally significant ($F(1,18)=3.969$, $p=0.062$). In contrast, in the total reading time which is the measurement for the later stage of processing, the interaction effect between the grammaticality and the local agreement was significant ($F(1,18)=5.935$, $p<.05$). Also, in the proportion of regression-out, the main effect of local agreement was significant ($F(1,18)=10.646$, $p<.01$). The results demonstrate L2 learners also show the attraction effect like English native speakers and it is reflected more clearly to later processing rather than early one. Ultimately, our results indicate high-proficient L2 learners did not show L1 transfer and they can process subject-verb number agreement in native-like way.

[Table 1] Examples of materials

Animacy of Local noun	Grammati-cality	Local agreement	Sentences
Animate	O	O	The instructors that trained the swimmers were very generous
	XThe instructors that trained the swimmer were very generous.		
X	OThe instructor that trained the swimmers were very generous.		
	XThe instructor that trained the swimmer were very generous.		
Inanimate	O	O	The girls that dressed the dolls were very lovely.
	XThe girls that dressed the doll were very lovely.		
X	OThe girl that dressed the dolls were very lovely.		
	XThe girl that dressed the doll were very lovely.		

Poster 178

EFL Learners' Narrative Reading Comprehension Processes on Illustrated Texts with Instructed Guidance: Evidence from Eye Movements

Ming-Yi Hsieh and Sunny S. J. Lin
Institute of Education, National Chiao Tung University, Taiwan

Narratives are authentic reading materials for English as a foreign language (EFL) learners. The emphasis on texts alone may implicitly introduce an avoidance of illustrations for EFL readers. Furthermore, post comprehension checks are commonly applied as assessments in language learning, and they rarely reveal the cognitive processes of reading. The current study investigated reading comprehension processes on 58 EFL struggling and proficient learners (Mage= 17.8) via eye-tracking techniques. Participants were systematically and randomly assigned into four groups, in which they were provided with/without guidance to read two different level narratives (simple/complex text) embedded with structures (prologue/climax/resolution) of illustrated texts. Guided groups were instructed to read illustrations before reading the experimental illustrated text, while non-guided groups were not provided instructions. The data analysis, fixation counts and dwell time, showed that struggling students read the complex text following the guidance to rely on illustrations. The proficient learners processed illustrations when reading the simple text. The findings suggest that with guidance, struggling learners relied on illustrations as a bridging mechanism to comprehend and integrate complex text whereas proficient learners processed simple text through illustrations to solve critical uncertain words. This study contributes to reading empirically and practically. Empirically, it demonstrates that illustrations have brought a better understanding of reading comprehension, especially when learners' native language is not English. Practically, for classroom teachers, with their instruction on reading illustrations, learners can have an effective understanding of simple and complex texts.

Poster 179

Exploring the effects of sound on students' e-book reading behaviors: An eye-tracking based study

Kao, Yi Ming; Hsieh, Ting Ru; Huang, Xing Yi
National Taiwan University of Science and Technology

E-books are commonly used to facilitate students' reading literacy. This study explored whether an e-book narrated by student peers could reduce students' cognitive load, raise their reading motivation, and enhance their reading comprehension. Eye-tracking technology was used to analyze the students' visual attention distributions during the reading process to understand the effects of sound on their reading behaviors. Participants were 75 junior high school students in Taiwan and were randomly assigned to one of four groups: text (picture + text), sound (picture + sound), mix (picture + text + sound), or peer voice (picture + text + peer voice). The results showed that the peer voice group had a higher reading motivation and fixated more on the main character, text and key areas of interest than the text group. Using peer voices as the narration could engage the students in observing the details in the pictures; therefore, the peer voice group performed better on the inference comprehension of the story. Also, the peer voice group's visual attention was more widely distributed on the e-book content, while the sound group fixated on the areas mentioned in the narration only.
Keywords: picture e-book, eye tracking, sound, narration, reading motivation

Poster 180

Impact of feedback variations in gaze controlled typing

Jens R. Helmert, Sven-Thomas Graupner, Sebastian Pannasch
Technische Universität Dresden

With the advent of faster, accurate and less expensive eye tracking devices, gaze based interaction becomes more and more important. This form of interaction crucially depends on a well set tradeoff between speed and accuracy. A major challenge is to ease the eyes' travel from one button to the next over the virtual keyboard. It is known that a disappearance of the current fixation target supports faster disengagement of visual attention (i.e. gap effect, Jin & Reeves, 2009); it should serve the idea of speeding up the departure to the next button. However, research on visual distractors (e.g. Pannasch & Velichkovsky 2009) would predict that the change induced by the disappearance should lead to the opposite effect—a prolongation of the current fixation. The latter aspect is also relevant in another design aspect: A visible gaze cursor provides feedback about the current gaze position, but at the same time imposes distracting movements at the foveal position.
In the current study, the gaze based interaction was varied in a 2 (gaze cursor visible vs. hidden) by 2 (button feedback bold vs. hide) design with both factors manipulated within subjects in a blockwise manner. After each block, a usability questionnaire was completed. All subjects participated in five consecutive sessions.
The results will be discussed against the theoretical background of distractor effect and gap effect. Especially, we will shed light on individual changes over time in the speed-accuracy tradeoff in the different conditions.

Jin & Reeves, 2009
https://www.sciencedirect.com/science/article/pii/S0042698909000674

Poster 181

Inferring problem-solving strategies through concurrent think aloud and eye tracking protocols

Aleksandra Kaszowska, Qianwen Wan, Nika T. Renshaw, Elizabeth McCall, Karen Panetta, & Holly A. Taylor
Tufts University

Solving ill-defined problems in real world settings is an inherently complex process that requires thinkers to integrate multiple knowledge domains and to continuously interact with the environment to maintain the most up-to-date definition of the problem space. In doing so, thinkers can divide the problem into a series of smaller, more manageable sub-problems, and address them in a stepwise fashion. Encountering difficulties when advancing between steps often results in impasses and forces the solver to readjust their approach.
In this work, we explore how people alter their thinking direction when working on a complex problem. We invited 45 engineering and human factors design students to solve complex problem: aid completion of a mundane sorting task by developing a sorting tool. Participants completed a concurrent think aloud and mobile eye tracking recording session. Exploratory analyses combining both data streams revealed two possible design strategies: a tendency to reject possible solutions early during the task ("shop around" strategy) and a tendency to switch direction when resolving an impasse ("cost-benefit" strategy). Semi-automatic processing tools developed for concurrent think aloud and eye tracking data will be discussed.

Poster 182

Interactive game-based picture e-books: An eye-tracking based study on problem-solving behavior

Not presented

Poster 183

Recording attention process in a natural lecture with mobile eye tracker: A case study

Li, Jerry N.C.; Lin, Sunny S.J.

Institute of Education, National Chiao Tung University, Taiwan; professor, Institute of Education, National Chiao Tung University, Taiwan

How to direct students' attention in the class is a basic but crucial skill for both expert and novice teachers because too many students have various difficulties to invest lengthy attention on teacher's lecture and teaching materials. This preliminary study explored the use of mobile eye trackers in a natural classroom for recording L2 students' sustainable attention toward the projection screen which was presenting peer- or self-works or attention toward teachers who were evaluating their works. Some cases in a 12th grade class were selected to show the effects of individual factor (ability) and teacher factor (gesture) on classroom attention. In the first session, the projection screen showed texts of self-introduction, while in the second session it showed slides with graphics and texts. Mobile eye tracker, Pupil Labs, were used to collect data. The projection screen and the position of teacher were set as AOIs. Scores of English test (TOEIC) and Digital information literacy test were also collected. We found, firstly, some teacher gestures easily lost students' focus on the contents (projector screen) and secondly there was at least one case who had poor English ability but actively paid attention in a lengthy duration. Though teachers urge to know students' attention process in the classroom, related eye movement research is still limited. Therefore, we provide several suggestions on how to conduct quality observation using mobile eye tracker, such as, how to set up AOIs to make heat maps useful and synchronize data with multiple devices for future researchers.

Poster 184

Similarities and differences in eye movements between professors and students during graph reading

Robbins, A., Pelnar, H., & Cameron, E.L.
Carthage College, Kenosha, WI, USA

Developing graphical literacy is a common struggle in undergraduate learning, making it a “bottleneck” in the language of *Decoding the Disciplines* (Pace, 2017). *Decoding* is a method to enhance student learning that utilizes introspection to make expert’s implicit knowledge and mental processes explicit for students. Using a tool employed by cognitive psychologists, namely eye-tracking, we are exploring ways of uncovering experts’ mental processes that move beyond introspection. In the present study, we examined eye movement patterns between professors (considered to be most adept at graph reading in an academic setting) and students. Participants viewed four graph types (bar, scatterplot, histogram, and factorial line) and verbally described the graphs while an eye tracker recorded eye movements. Performance of verbal description was measured. Both professors and students made fewer fixations and had more consistent scan paths in graph types with the highest verbal description scores. Though professors were better overall at describing graphs, both groups performed worst in describing factorial line graphs, and differences in eye movements between groups emerged for this graph type. Professors had longer dwell times and made more fixations to regions unique to the graph type and informative for interpretation. These data suggest that novice and expert graph readers have similar eye movements for most graph types, but patterns diverge with more computationally demanding graphs. These results complement related work in *Decoding the Disciplines* and may help instructors develop new strategies for teaching students how to interpret graphs.

Pace, D. (2017). *The Decoding the Disciplines Paradigm: Seven Steps to Increased Student Learning*, Bloomington, Indiana: Indiana University Press.

Poster 185

Assessment of Reading using Eye Tracking and Machine Learning

Gustaf Öqvist Seimyr and Mattias Nilsson Benfatto

The Marianne Bernadotte Centre, Department of Clinical Neuroscience, Karolinska Institutet, Sweden

The earlier reading deficits are discovered, the more effective interventions become. We have developed a method that quickly and accurately can assess reading difficulties with an accuracy of 95 %. We use machine learning of eye movements during reading to find children with reading problems, relative to expectations typical for the age and grade level. Based on results from a large population-based study performed in schools, we can now show that the method works for grades 1-3. In total, 3,444 screenings were performed in 21 schools. This is likely the largest eye tracking study ever performed on early readers. The results show that we achieve a binary classification accuracy of 86% with a good balance between sensitivity (85 %) and specificity (88 %). However, as reading deficits exist along a continuum, it is more appealing to predict the standard score. We find that a regression model gives a high correlation (R2 = .82) between observed and predicted standard scores. Although deviating eye movement patterns during reading are symptomatic rather than causal, the data from just a short passage yields a lot of information. In this talk, we will give an overview of the rationale for using eye tracking to assess reading ability. We see dyslexia as a language problem, and we will demonstrate how eye movements while reading reflect the cognitive processes involved. We will also present the research behind our method and discuss how it may be used for benchmarking as well as monitoring in response to intervention.

Poster 186

3D smooth pursuit: Comparison of two detection algorithms adapted for three-dimensional virtual environments

Zechner, Roland ; Freytag, Sarah-Christin
Technical University of Berlin

Smooth pursuit is a common input method for gaze-based interactions in 2D. 3D environments provide additional depth information that can enhance detection algorithms. A correlation-based (CBA) and a movement vector and angle-based algorithm (VABA) were adapted for smooth pursuit movement in 3D by integrating depth information. After implementation they were compared regarding their performance for different movement patterns and object distances.

12 participants gaze-followed one object moving on different circular and linear paths in 0.4m or 1.4m distance within a VR environment, shown via Head-Mounted Display. Each condition contained a comparatively high number of possible paths (26), chosen to avoid potential ceiling effects. As expected the overall detection rate was comparably low (CBA: 29%, VABA: 49%). Inferential statistical analysis for the CBA suggests a better detection performance for close objects (39%) than for distant objects (19%), as well as for movements on circular paths (33%) than on linear paths (26%). The VABA achieved a significant result only in terms of distance (close: 67%, far: 31%). Analyses of individual object movements suggest a better detection rate for movement in multiple dimensions. Preliminary results from a follow-up study suggest that detection rates increase with a lower number of object paths.

The results demonstrate that the type of algorithm plays a vital role in detection of smooth-pursuit movements in 3D spaces, with the vector/angle-based approach providing more robust detections across all conditions. Furthermore, closer distances enhance detection rates for both algorithms.

Poster 187

A Retina Inspired Model for High Dynamic Range Images Displaying

Xian-Shi Zhang, Kai-Fu Yang, Yong-Jie Li*
University of Electronic Science and Technology of China
liyj@uestc.edu.cn

Here we propose a retina inspired model to render the high dynamic range (HDR) images for displaying on the limited dynamic range devices. Despite its limited bandwidth in the visual pathway, Human visual system (HVS) allows human beings to perceive the details simultaneously in the darkest as well as the brightest areas of HDR scenes. Inspired by the processing mechanisms in the retina, the first HVS stage that is far smarter than scientists have believed, the proposed model shows competitive performance in terms of improving details in both the dark and bright areas.
The main novelty of the proposed model lies in the adaptive adjustment of the receptive field (RF) size of horizontal cells (HCs) based on the local brightness, which simulates the dynamic gap junction between HCs based on the physiological evidence. This local adaptation enables the brightness of distinct regions to be recovered into clearly visible ranges with the operation of negative feedback from HC to cone, while largely reducing the halo artifacts that are commonly produced by other methods. Another novelty of the proposed model is the introducing of the center-surround interaction of the bipolar cells' difference-of-Gaussian-shaped RF, which serves to eliminate the redundant information and enhance the local contrast. In addition, in order to work well on more HDR images of diverse scenes, the proposed model adaptively adjusts the responsive curves of the cone photoreceptors based on the statistical information of each image.

Poster 188

Decoding User's Task From Eye Movement Behavior In Virtual Reality

Keshava, A.[1], Aumeister, A.[1], Izdebski, K.[2], König,P.[1,3]

1. *Institute of Cognitive Science, University of Osnabrück, Wachsbleiche 27, 49090 Osnabrück, Germany*
2. *SALT AND PEPPER, Software GmbH & Co.KG., Kaffee-Partner-Allee-5, 49090 Osnabrück, Germany*
3. *Department of Neurophysiology and Pathophysiology, University Medical Center Hamburg-Eppendorf, Martinistraße 52, 20246 Hamburg, Germany*

INTRODUCTION: With the advent of state-of-the-art eye trackers, capturing human eye movement behavior in virtual environments has become markedly simplified. In this study, we take inspiration from the inverse Yarbus process, i.e., that eye movements can predict the user's task. Our aim was to explore whether and how well we can predict a user's instructed task based on their eye movements in an immersive virtual environment.
METHODS: We designed a simple task wherein participants were asked to align two cubes based on one of the four task conditions. Using raycasting, we calculated the point-of-regard (POR) on each cube from the position of the cyclopean eye for each time frame. Each POR was then labeled based on its location on the region-of-interest i.e. either the face or the edge of the two cubes. Finally, with 5-fold cross validation, we used logistic regression classifiers to predict the alignment task for a given trial.
RESULT: Our analysis showed the number of POR correlated strongly with the size of the two cubes, low for small cubes as compared to large and medium sized cubes. The number of POR was also high for the cube that was being manipulated. Taken together, these values also differed based on the type of alignment task. Our model performed above chance in predicting the four different alignment tasks with an accuracy of 39.05 (±1.8)% over the 5 folds.
DISCUSSION: Our results suggest that decoding the type of task based on the number of POR can be done above chance and sets an exciting roadmap for how to design decoding experiments in immersive virtual reality.

Poster 189

Different visual explorations between free-viewing and target finding tasks in websites: evidence from temporal analyses of ambient and focal modes

Milisavljevic, Alexandre[1, 2, 3] ; Le Bras, Thomas[1]; Abate, Fabrice[1]; Gosselin, Bernard[2]; Petermann, Coralie[3]; Mancas, Matei[2]; Doré-Mazars, Karine[1]

1Psychology institute, VAC EA7326 team, Paris Descartes University, Paris, France

2Numediart institute, University of Mons, Mons, Belgium

3Research and Development department, Sublime Skinz, Paris, France

alexandre.milisavljevic@etu.parisdescartes.fr

The human gaze during visual scenes exploration is influenced by many bottom-up and top-down factors such as the low-level visual characteristics or the given task to observers. It is also important to note that gaze behavior is intrinsically dynamic and thereby can be characterized by two visual exploration modes with switches from one to the other during the exploration. Fixation duration and saccade amplitude parameters define a mode as ambient with short fixation durations and large amplitudes or focal with long fixation durations and small amplitudes (Unema et al., 2005). A quantification of the do minant mode was introduced with the “K coefficient” (Krejtz, et al., 2016). Here, eye movements of 130 participants were recorded while carrying out free-viewing and target finding tasks on 18 fully scrollable webpages (for 35 seconds each). The K coefficient, the number of mode switches and the duration in each mode were analyzed. The main effect of the task (free-viewing vs. target finding) was significant on the switch number and the mode duration but not on the K coefficient. Indeed, gaze exploration in a website seems more focal and stable, with fewer mode switches and longer mode durations, when we are looking for a target than we explore it freely. However, a dynamical analysis (each trial was divided in 15 bins of 2.33 sec) has highlighted significant differences between visual tasks on all variables but only for the first part of the exploration.

Poster 190

LibreTracker: A Free and Open-source Eyetracking Software for head-mounted Eyetrackers

André Frank Krause[1] and Kai Essig[2]
[1]Mediablix IIT GmbH, Germany; [2]Rhein Waal University, Kamp Lintfort, Germany
post@andre-krause.net

LibreTracker is a free and open-source eyetracking software, licensed under the GPL-3. It is targeted at low-cost and do-it-yourself head-mounted Eyetrackers like the Pupil Labs headset, but works with other headsets as well. Implemented in modern C++, it has only minimal dependencies (OpenCV and the Fast Light Toolkit) and runs on many platforms (e.g. Windows, Linux and MacOS). Due to its small performance footprint, LibreTracker scales from powerful desktop workstations down to embedded devices. All USB video class compliant cameras can be used, with camera focus and exposure being adjustable for many of them. Using AR markers, the distortion of the scene camera can be evaluated, and, if necessary, calibrated. Further, we use on-screen AR markers to implement easy to use n-point calibration and scene-camera pose-estimation, such that the on-screen gaze point can be calculated regardless of head movements. For pupil-center estimation, we use a highly improved version [Krause and Essig, ETRA, 2019] of gradient based dark pupil tracking [Timm and Barth, Visapp 11, p. 125–130, 2011] that can be scaled to require less than 15ms per frame on a Raspberry Pi or less than 1ms on a Workstation. As an application scenario, we have implemented an eyetracking speller with a full virtual keyboard. The source code can be found at: https://github.com/afkrause/libretracker backup site: andre-krause.net/publications/ecem2019

This work was supported by the European Regional Development Fund (ERDF, Grant No.: GE-1-1-047) and the State Government of North Rhine-Westphalia (Grant No.: IT-1-2-001).

Poster 191

On coupling of EyeStalker algorithm with USB3.0 camera for affordable eye tracking

Mikushin, Dmitry; Korotaev, Kirill; MacInnes, William Joseph
Glazomer; Glazomer; HSE, Vision Modelling Lab

The price of professional eye-tracking systems for scientific and clinical applications starts at $10,000, making investigations of visual perception and attention along with diagnostics of neurological disorders prohibitively expensive. This situation is driven by two factors: the cost of complex and often excessive vendor-specific software and the cost of high-frequency camera. In our study, we explore the possibility of reducing the costs by means of coupling open source eye-tracking framework with recently emerged 800+ FPS conventional USB 3.0 cameras.
We have developed a test suite to evaluate the novel EyeStalker method based on recursive Haar-like features, Canny edge detection and segmentation pipeline (Brouns, 2017). The original eye tracking application has been modified to deploy a conventional high-speed USB 3.0 camera. We also show how the camera performance could be affected by the host system and discuss the optimal system configuration. The resulting setup has been intensively evaluated in the daily research work. We present the collected statistics and valuable lessons learned from this study.

Poster 192

SMITE and Titta: toolboxes for creating Psychtoolbox and PsychoPy experiments with SMI and Tobii eye trackers

Marcus Nyström[1], Richard Andersson[2], Diederick C. Niehorster[1,3,]

1 Lund University Humanities Lab, Lund University, Lund, Sweden

2 Tobii Pro AB, Stockholm, Sweden

3 Department of Psychology, Lund University, Lund, Sweden

Eye tracker manufacturers often provide a software development kit (SDK) or application programming interface (API) to allow users to interface their stimulus presentation software with the eye tracker. However, these tools are often bare bone and do not include examples that can easily be transformed into actual experiments. We present SMITE and Titta, two toolboxes implemented in PsychToolbox and PsychoPy to communicate with eye trackers from SensoMotoric Instruments and Tobii AB. The toolboxes contain a graphical interface for participant setup and calibration, integration with manufacturers' own visualization and data export software, as well as several examples that demonstrate the functionality of the toolboxes and make it easy to get started with your own eye tracker experiment.

Poster 193

Validation of a new tablet-based eye-tracker for oculomotor research

Aylin König[1,2], Stefan Dowiasch[1,2,3] & Frank Bremmer[1,2]
1) Dept. Neurophysics, Universitiy of Marburg, Germany
2) Center for Mind, Brain and Behavior – CMBB, Universities of Marburg and Giessen, Germany
3) Thomas RECORDING GmbH, Giessen, Germany

Eye-movements offer a reliable and non-invasive way to detect and quantify brain functions and their neural correlates. Typically, such studies are performed with highly sophisticated stationary eye-trackers in laboratory setups. Unfortunately, they often require a lengthy training in their handling by qualified personnel and set high demands on the environment and the compliance of the subjects. A novel, fully integrated tablet device, the Thomas Oculus Motus - research mobile (TOM, Thomas RECORDING), aims to overcome these limitations to allow for an easy, fast and natural use with low compliance demands. It records head-unrestrained eye movements with 30Hz in almost any environment, while simultaneously providing visual (and auditory) stimulation.
Here, we compared the data output of this mobile eye-tracker with a well-established system, an EyeLink 1000 (SR Research) with a sampling rate of 1000Hz. Ten healthy volunteers performed a mixed pro and anti-saccade task and a free viewing task while eye-movements were recorded with both systems simultaneously. Despite huge technical differences, common eye-movement parameters such as fixation durations and locations as well as frequency, direction and amplitude of saccades showed no significant difference between both systems. Hence, typical gaze sequence analyses in the free viewing task and performance in the pro-/anti-saccade task showed equal results for both devices.
We conclude that the TOM is well suited to complement classical laboratory-based eye-trackers in a wide range of psychophysical experiments. The mobility, versatility and low compliance demands might be especially useful for basic experiments in large populations or patient studies in clinical settings.

Supported by: BMBF-DIADEM

Poster 194

An EEG marker of the intentional smooth pursuit in human-machine interaction

Darisy G. Zhao[1], Anatoly N. Vasilyev[1,2], Bogdan L. Kozyrskiy[1], Evgeniy V. Melnichuk[1], Andrey V. Isachenko[1,3], Boris M. Velichkovsky[1,4], Sergei L. Shishkin[1]

1 National Research Center «Kurchatov Institute», Moscow, Russia;

2 M.V. Lomonosov Moscow State University, Moscow, Russia;

3 Moscow Institute of Physics and Technology, Moscow, Russia;

4 Technische Universität Dresden, Dresden, Germany

E-mail: chzhao@phystech.edu

Detection of the smooth pursuit eye movement can be used to select an object moving on a computer screen (Vidal et al., 2013). However, moving objects may also become pursued without intention to select and become incorrectly selected. We hypothesized that intentional and spontaneous pursuits can be accompanied by different EEG patterns. We coregistered EEG and eye movement under two conditions: for intentional pursuit, participants were asked to select moving objects with gaze (pursuit caused target highlighting); spontaneous pursuit was provoked by an instruction to count dots on the moving objects. Both intentional and spontaneous pursuits were featured with an EEG lambda wave well-known for static gaze fixations. The intentional, but not spontaneous selection was accompanied, in all of the 20 participants, by an EEG wave, presumably related to the expectation of the feedback. This wave was similar, by time course and topography, to the EEG marker described for static object selection (Shishkin et al., 2016). The selection-related EEG marker did not differ between moving objects selection with smooth pursuit and static objects selection with dwells. Using a deep neural net (EEGNet, Lawhern et al., 2018), we successfully classified intentional vs. spontaneous pursuits. These results support the proposal of enhancing gaze interaction with an expectation-based passive brain-computer interface (Protzak et al., 2013), showing high robustness of the EEG marker related to the expectation of the interface feedback.

The study was partially supported by the Russian Science Foundation, grant 18-19-00593 (development of techniques for data preprocessing and classification).

Participants list
20th European Conference on Eye Movements

"İyilikci, Osman" 265
Abate, Fabrice 390
Abrahamson, Dor 66
Abramczyk, Marta 51
Acarturk, Cengiz 192
Achtziger, Anja 170
Adedeji, Victoria I. 245
Agtzidis, Ioannis 208
Aguilar, Carlos 117, 137
Ahn, Seoyoung 124
Aiple, Franz 169
Akbarinia, Arash 358
Akhutina T. 275
Akthar, Farah 116
Alahyane, Nadia 25, 371
Alarcón-Osollo, Adrián 65
Albrecht Werner, Inhoff 220
Albrengues, Claire 137
Alexa, Marc 173
AlJassmi, Maryam A. 143
Allen, Shanley E.M. 129, 359
Allison, Robert 214
Alonso, J 333
Amado, Sonia 265
Amagai, Kyoichi 334
Anderson, Nicola C. 74, 75, 59
Andersson, Richard 393
Angele, Bernhard 187
Anisimov, Victor 176, 255
Annerer-Walcher, Sonja 225
Annika, Wallin 194
Ansari, Ehsan 250
Anschuetz, Lukas 204
Antal, Caitlyn 80, 148
Antolin, Jastine 227
Antoniades Chrystalina A. 60, 341
Appel, Tobias 202, 347
Ardasheva L. 316
Armson, Michael J. 304
Armstrong, Thomas 50
Arruda de Souza, Brenda 147
Arsalidou, Marie 153
Atkins, Andrew 377
Aumeister, A. 389
Aurélien, Morize 330
Aust, Elisa 203
Azulay, Hagar 326
Baccino, Thierry 164, 232
Bachirina, Valentina 153
Backhaus, Daniel 85
Bai, Xuejun 186, 254, 288, 306
Bakaraju, Ravi 354
Bakker, Arthur 66
Balagué, Marta 152
Ba_aj Bibianna 216
Balcombe, Faye O. 242
Balsdon, Tarryn 374
Barach, Eliza 161, 180
Baroni, Tatiana 331
Barr, Dale 210
Barra, Julien 264
Barrett, Doug JK 198
Barsingerhorn, Annemiek 294
Barton, Hayley 285
Baruah, R. 219
Barza, Lydia 64
Belopolsky, Artem V. 111
Ben-Shakhar, Gershon 199
Benedek, Mathias 225
Benjamins, Jeroen S. 356
Bertel, Sven 213
Bertram, Raymond 141, 166, 281
Bidet-Caulet, Aurélie 150, 235
Biemann, Chris 273
Biscaldi, M. 59, 228, 335, 310
....
Bischof, Walter F. 74, 75, 348
Bishof, W. 59
Bizjak, Bianca 328
Blinnikova I. 268, 286
Blohm, Gunnar 272
Blumberg, Julie 169
Blythe, Hazel I. 319, 320
Blythe, I. 131
Boccignone, G. 59
Bocheva, Nadejda 369
Bolderston, Helen 227
Bonneh, Yoram 189, 364
Borkowska, Olga 63
Boshra, Rober 140
Bosse, Marie-Line 237
Botao, Peng 330
Bouamama, Sana 158
Bourgois, Jan 190
Bourrelly, Clara 118
Brandner, Martina 328
Brandt, Armin 169
Brandt, Silke 144
Brawn, James 177
Bremmer, Frank 394
Brémond-Gignac, Dominique 159
Brennan Payne 22
Breteler, Monique M.B. 160
Brinkman, Hanna 134
Brückler, Bettina 221
Brych, Mareike 259, 266
Buchanan, Tim 60
Buchsbaum, Bradley R. 46
Bulling, Andreas 256
Burmistrov I. 268

Bürsgens, Annika ... 293
Bush, Charlotte ... 195
Butorina, Anna V. ... 155
Byrne, Ruth ... 83
Cajar, Anke ... 77
Calabrèse, Aurélie ... 117
Calvo, A ... 61
Cameron, E.L. ... 385
Canu, D. ... 228, 310, 335
Cañal-Bruland, Rouwen ... 193
Cao, Liyu ... 266
Cara, Michel ... 68
Cardoso Pereira, Norberto ... 58
Carrasco, Marisa ... 87
Carvalho Ribeiro, Antonio João ... 136
Caspi, Avi ... 99
Cassanello, Carlos ... 168
Casteau, Soazig ... 86, 195
Castelhano, Monica S. ... 78
Castet, Éric ... 117, 137
Castner, Nora ... 202, 347
Caversaccio, Marco ... 204
Ceh, Simon ... 225
Cenek Sasinka ... 302
Cenek, Jiri ... 263
Chalkley, Mary ... 195
Chamorro, E ... 333
Chan Seem, Rob ... 126
Chandra, Johan ... 138, 276
Chang, Min ... 106, 278
Chaturvedi, Akshay ... 43
Chen, I-Ju ... 284
Chen, Yupei ... 124
Cheviet, Alexis ... 336
Chmelik, Jiri ... 302
Choi, Jinyeong ... 342
Cholewa, Jürgen ... 293
Chumachenko, Dmitry ... 165
Chung, Hyenyeong ... 376, 378
Ciesielski K. ... 271
Claudia, Quaiser-Pohl ... 290
Cleva,JM ... 333
Clifford, Colin ... 208
Coco, Moreno I. ... 79, 307
Coll, S.Y. ... 337
Collins, Therese ... 373
Colonnesi, Cristina ... 314
Concepcion,P ... 333
Conesa, Natàlia ... 152
Cong, Fengjiao ... 107
Connolly, John ... 140
Conrad, Fabian ... 354
Conti, Federica ... 211
Coors, Annabell ... 160
Costela, Francisco M ... 115
Costumero, Víctor ... 282
Coutinho, Jonathan D ... 272
Crawford, Trevor J. ... 329
Creaser, G. ... 101
Cristian, John ... 359
Cui, Lei ... 107
Cutsuridis, Vassilis ... 177
Cutter, Michael ... 71, 181
Dalmaijer, Edwin S ... 50
Damania, K. ... 59
Danczura, Ewa ... 332
Daniel, Francois ... 43
Danielmeier, Claudia ... 327
Dantas, Sibelius Clausen ... 318
Dare, Zoya ... 134
David Souto ... 215
Davidenko, Nicolas ... 53
Dawson, Jessica ... 236
Dayan, J. ... 337
Dayan, Oryah chen ... 199
de Almeida, Roberto G. ... 80, 148
de Kloe, Y.J.R. ... 231
de'Sperati, Claudio ... 61, 311, 331
Degno, Federica ... 239, 253
Deitcher, Yishai ... 114
Della Sala, Sergio ... 307
Demeter, Gyula ... 81
Dempere-Marco, Laura ... 152
Dent, K. ... 340
Deubel, Heiner ... 88, 125
Di Nardo, Julia ... 80
Dillon, Brian ... 145
Dimigen, Olaf ... 323, 352
Dimigenc, Olaf ... 79
Dithurbide, L. ... 101
Doidy, F. ... 337
Dominique, Brémond-Gignac ... 330
Donkers, Jeroen ... 98
Donnelly, Nick ... 55, 253
Donovan, Tim ... 97
Doré-Mazars, K. ... 224, 264, 361, 371, 390
Dorr, Michael ... 135, 208
Dos Santos Rodrigues, Erica ... 136, 246
Doswell, Michael T. ... 75
Doubrovski V.E ... 350
Dowiasch, Stefan ... 394
Drai-Zerbib, Véronique ... 164, 232
Dreneva, Anna ... 165
Drieghe, Denis ... 126, 158, 319
Du, Hong ... 186
Dubray, David ... 346
Dubynin, Ignat A. ... 155
Duchowski, Andrew T. ... 51, 56, 63
Dufau, Stéphane ... 303
Duke, Robert A ... 62

Dulyan, Lilit G. 362
Dunn, Matt J. 177
Ebner-Priemer, U. 59
Ebrahim, Pishyareh 57
Edelman-Klapper, Hadar 349
Eder, Thérése 202
Eekhof, Lynn S. 37
Ehinger, Benedikt V 54, 352, 353
Ehrmann, Klaus 354
Einspieler, Christa 328
Elizabeth R. Schotter 22
Elschner, Sophie G. 82
Engbert, Ralf 77, 85, 91, 138, 233, 276, 344
Entzmann, Léa 372
Erichsen, Jonathan T. 177
Erlinghagen, Charlotte 166
Espino, Orlando 83
Essig, Kai 391
Ettinger, Ulrich 160
Eustache, F. 337
Faber, Myrthe 37
Fal, Mehmetcan 192
Fechino, Marion 37
Fehringer, Benedict C.O.F. 133
Feldman, Laurie B. 180
Feng, Siqi 288
Fennell, Anna Marie 184
Fernandes, Eunice G. 171
Fernandes, Natália 136
Fernández-López, María 280
Fernandez, Leigh 129, 359
Feurra M. 316
Filik, Ruth 241, 243, 327
Fink, Lauren 95, 313
FitzGerald, James J. 341
Fleischhaker, C. 228, 310, 335
Folk, Jocelyn R 40
Forster, Renê 246, 318
Foulsham, Tom 59, 120, 340
Fraisse, F. 337
Fran_k, Marek 205
Freitas Pereira Toassi, Pâmela 187
Frens, Maarten 28
Frey, Aline 237, 277
Freytag, Sarah-Christin 387
Frühwirth-Kaspar, Daniela 328
Fuchs, Philippe 76
Funabiki, Yasuko 334
Füssel, Stephan 274
Gagnepain, P. 337
Gaissmaier, Wolfgang 82
Galiana, H.L. 175
Galkina, N. 255
Gallis, Quentin 264
Gamboa, Borges 359
Gani-zada, J. 321
Gao, Hong 347
Garcia Moreno-Esteva, Enrique 191
García-Madruga, Juan A. 83
Garusev A.V. 350
Gaugl, Heike 328
Géciová, Klára 287
Gegenfurtner, Karl 358
Gehrer, Nina A. 56
Geng, Joy 95
Genova, Bilyana 369
Gert, Anna Lisa 54
Ghasia, Fatema F. 46
Giannini, Marcello 157
Gidlöf, Kerstin 194
Gilchrist, Iain D. 109, 221
Giusiano, S. 61
Gloriani, Alejandro H. 110
Gloskey, Leah 161
Godfroid, Aline 67
Goffart, Laurent 118
Golovatyuk Maria 248
Gonzales, A. 333
Goossens , J. 102
Gopikrishna, D. 128
Gorina E 316
Gosselin, Bernard 390
Grabmaier, Christoph 300
Grainger, Jonathan 303
Graupner, Sven-Thomas 203, 381
Gregori-Grgi_, Regina 311, 331
Gregory, Nicola 227
Grigorovich S. 268
Groß, Katharina 353
Gubitz, G. 101
Guérin-Dugué, Anne 277, 372
Guerre-Genton, Agnès 303
Guerreiro, Manuela 58
Guitton, Daniel 28, 375
Günther, Thomas 293
Guo, Yuxuan 210
Gurtner, Lilla 348
Guy, Nitzan 123, 326
Guyader, Nathalie 372
Haghgoo, Hojjat Allah 57
Häikiö, Tuomo 127
Halamová, Júlia 325
Hämäläinen, Jarmo A. 247
Hammar, Björn 172
Händel, Barbara 259, 266
Hanning, Nina M. 125
Hannula, Markku S. 191
Hansen, Dan Witzner 193
Hartmann, Matthias 296
Hassin, Ran 326

Hausamann, Peter 156
Hautala, Jarkko 183, 247
Hawelka, Stefan 188, 287
Hazel 131
Heckman, Jesse 294
Heikkilä, Timo T. 283
Heinrich, Amelie 193
Helmert, Jens R. 151, 381
Hepsomali,Piril 253
Hermann, Andreas 203
Hermena, Ehab 158
Hessel, Annina 132
Hessels, Roy S. 30, 96, 231, 351, 356
Hicken, Laura K. 62
Hickey, E. 101
Hiebel, Hannah 221
Hill, H. 59
Hillairet de Boisferon, Anne 25, 371
Hirschbichler, Stephanie 368
Hoffmann, Mareike A. 315
Hofmann, Markus J. 273
Holleman, G.A. 351
Hollingworth, Andrew 112
Holmberg, Nils 194
Holmqvist, Kenneth 173, 260
Hong, Upyong 376, 378
Hooge, Ignace T.C. 96, 231, 351
Howman, Hannah 241
Hsieh, Ming-Yi 379
Hsieh, Ting Ru 380
Hu jinsheng 218
Huang, Jeff 258, 272
Huang, Linjieqiong 238
Huang, Xing Yi 380
Huckauf, Anke 300
Huestegge, Lynn 295, 315
Hurley, Brian 95
Hurtado, Álvaro 68
Husain, Masud 308
Hüttig, Fabian 202
Hutzler, Florian 287
Hynes, Patrick 357
Hyönä, Jukka 107, 283
Ibánez, Jaime 368
Ibs, Inga 353
Inhoff, Albrecht 130
Ioannou, C. 59, 228, 310, 335
Isachenko, Andrey V. 395
Ischebeck, Anja 221
Izdebski, K. 389
Izmalkova A. 286
Jacobs, Arthur M. 37
Jainta, Stephanie 119
James, FitzGerald 60
Janata, Petr 95
Janik, Miroslav 260
Jarodzka, Halszka 98, 309
Jarosch, Julian 274
Jaudas, Alexander 170
Jennifer Sudkamp 215
Jincho, Nobuyuki 252
Johandera, Eerika 142
Jóhannesson, Ómar I. 297
Johansson, Roger 34
Jokinen, Kristiina 299
Joos, Markus 203
Junge, C.M.M. 231
Jurik, Vojtech 302
Jurinec, Nina 338
Jusyte, Aiste 56
Kaakinena, Johanna K. 37, 142
Kadosh, Oren 364
Kalsi, Tami 243
Kao, Gloria YI-Ming 383
Kao, Yi Ming 380
Kapoula, Zoi 43, 159
Kaprielova, Anastasiia 249
Kardosh, Rasha 326
Karelin, Alexander 176
Karelin, Stanislav 317
Karraz, Marla 224
Kasatkin, Vladimir 176
Kashino, Makio 209
Kasneci, Enkelejda 202, 347
Kaspar, Kai 217
Kaszowska, Aleksandra 382
Kato, Masaharu 334
Kato, Takaaki 209
Kawabata, Hideaki 134
Kekunnaya, Ramesh 100
Kemner, C. 231, 351
Kenneth, Holmqvist 94
Kern, Markus 169
Keshava, A. 389
Keutel, Constanze 202
Keyvanara, Maryam 214
Kharlamov, Nik 73
Kietzmann, Tim 54
Kim, Junhui 291
Kim, Soojeong 376
Kim, Taehyun 342, 343
Kim, Yeseul 342, 343
Kingstone, Alan 59, 74, 75
Kirkby, Julie A. 40, 244, 245
Kirkden, Richard 239
Kirsanova S. 286
Kirtley, Clare 69
Klanke, Jan-Nikolas 363
Kleberg, J.L. 25
Klein-Peschanski, C. 59, 228, 310, 335, 337

Kleinsmith, Abigail L. 163
Koch, Iring 315
Kolodziejczyk, K.Z. 128
König, Aylin 394
König, Peter 54, 353, 389
Konishi, Mahiko 154
Konishi, Yukuo 334
Kopachevskaia, M. A. 324
Korneev A. 275
Körner, Christof 221, 225, 328
Korosteleva, Anastasia N. 151
Korotaev, Kirill 392
Kovalev, Artem I. 267
Kovaleva, A. 255
Kozyrskiy, Bogdan L. 155, 395
Krasovskaya, Sofia 207
Krause, André Frank 391
Krejtz, Izabela 51, 223, 256
Krejtz, Krzysztof 51, 63, 256
Kretzschmar, Franziska 274
Krichevets, Anatoly 165
Krieber-Tomantschger, Magdalena 328
Kristjansson, Arni 121, 207, 297
Kroll, Aleksandra 332
Kromina, Anastasia 366
Krügel, André 138, 234, 276
Ksenia, Dovbnyuk 257
Kucharsk_, _imon 261
Kuijpers, Moniek M. 37, 313
Kuperman, Victor 140, 166, 178
Kurth, Anno 345
Kuzmi_ová_, Anezka 37
Laamerad, Pooya 375
Lacko, David 269
Laisney, M. 337
Lange, Elke 95, 313
Langevin, Etienne 277
Langmann, Andrea 328
Laohakangvalvit, Tipporn 299
Lappe, Markus 208, 360, 367
Latanov, Alexander 176, 255
Lau, Wee Kiat 373
Laubrock, Jochen 40, 77, 346
Laurence, Paulo 261
Laurinavichyute, Anna 179, 249, 366
Lavigne, Frédéric 137
Lawrence, Claire 327
Le Bras, Thomas 390
Leder, Helmut 134
Lee, Alex 50
Lee, Charlotte 319
Lee, Joy Yeonjoo 98
Lefevre, Philippe 272
Lehtonen, Minna 281
Lemaire, Benoît 277
Lemoine-Lardennois, Christelle 371
Lencer, Rebekka 208
Lenoir, Matthieu 190
León, J. A. 72
Leppänen, Paavo H.T. 183, 247
Leroy, L.J. 361
Levine, Brian 304
Lewicka Maria 216
Li , Hsin-Hung 87
Li, Yutong 218
Li, Jerry N.C. 384
Li, Liang 122
Li, Lin 105, 106, 278
Li, Sha 106
Li, Shiyi 306
Li, Xingshan 103, 238
Li, Yong-Jie 230, 388
Liebscher, Daniel 203
Lienhart, Anna M. 328
Lieven, Elena 144
Lin, Sunny S.J. 379, 384
Lin, Tzu-Hsuan 284
Lindner, Susanne 328
Ling, Wei 52
Linse, Katharina 203
Litchfield, Damien 97
Liu, Min 185
Liu, Yanping 104
Liu, Zhong-Xu 198
Liversedge, Simon P. ....
.........55, 106, 131, 158, 185, 186, 239, 253, 254, 320
Liya, Merzon 257
Llorens, Alberto 282
Loberg, Otto 183, 247
López-Nájera, Abraham 65
López-Orozco, Francisco 65
Lopukhina, Anastasiya 179, 366
Lörch, Lucas 305
Lorenzo Villalobos, A. E. 61
Lovell G. 271
Loya-Sánchez, Carlos Esteban 65
Lu, Zhongjiao 60
Lubi_ski Alex 216
Lüdtke, Jana 37
Ludwig, Casimir J.H. 91
Ludwig, Jonas 170
Luna, B. 25
Luniakova E.G. 321, 350
“Młyniec, Agnieszka” 223
“Małgorzata” 223
Macdonald, Ross 144
MacInnes, William Joseph .. 207, 257, 316, 362, 392
MacKenzie, D.E. 101
Macknik, Stephen L. 46
MacNeilage, Paul R. 156

Magalhães, Cláudio Freitas de 318
Magyari, Lilla 37
Mak, Marloes 37
Mak, Monika 332
Mäkinen, Tiina 127
Malakhov, Denis G. 155
Malyutina, Svetlana 179
Mancas, Matei 390
Mangen, Anne 37
Mannan, Sabira 206
Manohar, Sanjay 368
Marcet, Ana 280
Marcum, Travis 62
Marschik, Peter B. 328
Marsh, Olivia 212
Martin-Arnal, L. A. 72
Martin, Andrea E. 71, 181
Martinez Cedillo A. P. 340
Martinez-Conde, Susana 46, 115
Martins da Costa, Maria Armanda 58
Masselink, Jana 367
Massol, Stéphanie 282
Mast, Fred 204, 348
Mateusz Bocian 215
Mathôt, Sebastiaan 149
Matonti, Frédéric 117
Maturi, Kinnera S. 162
Matveeva E. 275
Maus, Gerrit W. 373
Mayer, Erika 145
Mayer, Sven 256
Mazuka, Reiko 252
McCall, Elizabeth 382
McCloy, Rachel 109
McGowan, Victoria A. 128, 143, 182, 285
McInnes, Joseph 270
McIntyre, Nora 64
McSorley, Eugene 109
Méary, David 237
Medendorp, Pieter 28
Meghanathan, Radha Nila 93
Meliksetian A. 286
Meli_, Honza 205
Melnichuk, Evgeniy V. 395
Menshikova, Galina 322, 324
Merritt, B.K. 101
Merten, Natascha 160
Métais, Camille 235
Meyhöfer, Inga 208
Meza-Garcia, Eduardo Ariel 65
Michael C. W. YIP 289
Mikhailova, Anastasiia 307
Mikheev, Vladimir 270
Mikuni, Jan 134
Mikushin, Dmitry 392
Milisavljevic, Alexandre 264, 390
Milledge, Sara V. 131
Miller, S.G. 101
Milligan, Sara 226
Minarikova, Eva 260
Mirault, Jonathan 303
Mirko, Saunders 290
Mita_ík, Marek 325
Moglia, C. 61
Montagnini, Anna 211
Moon, Sunhyun 342, 343
Morize, Aurélien 43, 159
Móro, Róbert 325
Morris Moscovitch 197
Munoz, Douglas P 25, 28, 258, 272
Münzer, Stefan 133
Murali, Supriya 266
Murray, Christopher 69
Musso, L. 61
N. Teixeira, Elisangela 147
Nadezhda, Murzyakova 257
Naghizadeh, M. 229
Nahari, Tal 199
Nam, Yunju 376, 378
Neveu, Pascaline 76
Neyedli, H.F. 101
Nicolas, Judith 150, 235
Niederhauser, Laura 204
Niehorster, Diederick C. 356, 393
Nielek, Rados_aw 256
Nikolaev, Andrey R. 93, 157
Nila Meghanathan, Radha 157
Nilsson Benfatto, Mattias 386
Nisbet, Kelly 166
Niu, Lili 122
Nivinsky-Margalit, Shany 349
Nogueira Teixeira, Elisangela 187
Noh, Soo Rim 342, 343
Nuthmann, Antje 79, 262
Nuzhdin,Yuri O. 155
Nyström, Marcus 96, 172, 309, 393
Ohl, Sven 363
Olkoniemia, Henri 142
Olmos, R. 72
Olszanowski, Micha_ 51
Öqvist Seimyr, Gustaf 386
Oralova, Gaisha 140
Orenes, Isabel 83
Ossadtchi, Alexei E. 155
Ossandon, Jose 100
Ostendorf, Florian 168
Oster,Julia 258
Otero-Millan, Jorge 365
Oz, Roy 349
P. Dantas, Antonio 147

Pack., Christopher C. 375
Paeye, Céline 370
Pagán, Ascensión 239
Pajkossy, Péter 81
Paletta, Lucas 301
Palva, Satu 154
Palvaa, Matias 154
Pan, Jasmine 87
Pan, Shasha 278
Panetta, Karen 382
Pannasch, Sebastian 151, 203, 210, 381
Parker, A. J. 244
Parker, Adam J. 245
Paterson, Kevin B. ....
.......105, 106, 128, 139, 143, 182, 212, 239, 243, 285
Peißl, S 219
Pélisson, Denis 150, 235, 336
Pelnar, H. 385
Peng, Botao 159
Perea, Manuel 280, 282
Pertzov, Yoni 123, 199, 326
Peschanski, D. 337
Petermann, Coralie 390
Petrova Tatiana 240, 292
Petru_álek, Jan 205
Pfeiffer, Thiess 300
Pfeuffer, Christina U. 298
Phillips, Louise 171
Pichugina, Anna 322
Picketts, L. 101
Pieczykolan, Aleksandra 166
Pieter, Blignaut 94
Pilling, Michael 198
“Piotrowska-Półrolnik,” 223
Pisella, Laure 336
Pokorny, Florian B. 328
Pons, Álvaro 339
Popelkac, Stanislav 269
Potthoff, Jonas 338
Povlsen, Alexander F. 73
Pressigout, A. 361
Prichard, Caleb 377
Priot, Anne-Emmanuelle 76
Prokopenya, Veronika 251
Pszeida, Martin 301
Quinet, Julie 118
Quiñonez-Domínguez, César 65
Rabe, Maximilian M. 276
Rabeson M. 286
Racsmány, Mihály 81
Radach, Ralph 130, 250, 258, 273
Raevskiy A. 286
Rahne, Mar-kus 172
Raijmakers, Maartje 89, 90, 261
Raposo, Ana 307
Ravnkilde, Kasper H. 73
Regnath, Franzi 149
Reichle, Erik D. 104
Reilly, Ronan 40, 357
Reinacher, Peter 169
Reiner, Miriam 355
Reininghaus, Sina 300
Remus, Steffen 273
Renhao Liu 52
Renshaw, Nika T. 382
Richter, Juliane 202
Riechelmann, Eva 295
Riekhakaynen Elena 292
Rizzuto, Vincenzo 364
Roatta, S. 61
Robbins, A. 385
Rodenburg, Roos 314
Röder, Brigitte 100
Rodrigues, Erica dos Santos 318
Rogerson, Natalie 195
Rolfs, Martin 168, 363, 374
Roque, Monique 318
Rosenbaum, R. Shayna 198
Rosenberg, Raphael 134
Rosengren, William 172
Rosenzweig, Gal 189
Rosner, Agnes 34
Rosser, Anne 177
Rothkegel, Lars O. M. 85, 233
Rothwell, John 368
Roumes, Corinne 76
Rüth, Marco 217
Ryan, Jennifer D. 197, 198, 304
Sachar, Yaron 114
Sadeghpour, Shirin 365
Saijo, Naoki 209
Sainan, Li 185
Salasc, Charles-Antoine 76
Salem-Cherif, Wissam 371
Salmela, Rosa 141, 281
Salminen-Saari, Jessica F. A. 191
Salomon, Israel 326
Sanchis-Jurado, Vicent 339
Sasinkaa, Cenek 263, 269
Scheepers, Christoph 129
Scheiter, Katharina 202
Schienle, Anne 338
Schlachter, Tina Andrea 262
Schlesewsky, Matthias 274
Schmid, Fabienne 259
Schmidtke, Daniel 140
Schmitt, L.M. 25
Schönenberg, Michael 56
Schotter, Elizabeth R. 184, 226
Schroeder, Sascha 92, 132

Schulze-Bonhage, Andreas ... 169
Schuster, Sarah ... 287
Schütz, Alexander C. ... 108, 110, 167
Schwalm, Laura ... 130, 250
Schweigert, Robin ... 256
Schweitzer, Richard ... 374
Schwetlick, Lisa ... 233
Scott-Brown, Kenneth C. ... 222, 271, 312
Sebastian, Reich ... 344
Seelig, Stefan A. ... 276, 344
Seernani, D. ... 59
_efara, Denis ... 205
Seiffert, Nicolas-Alexander ... 217
Semin, Gun R. ... 192
Senden, Mario ... 345
Senot, Patrice ... 264
Serratrice, Ludovica ... 144
Sharaev, Maxim G. ... 151
Shareef, Idris ... 100
Shedenko, K. ... 255
Shenton, Sophie ... 312
Sheridan, Heather ... 161, 162, 163, 180
Shishkin, Sergei L. ... 155, 395
Shiva, Janmohamadi ... 57
Shurupova, Marina ... 176
Shuwei Xue ... 37
Shvarts, Anna ... 66, 165
Sicuro Corrêa, Letícia Maria ... 246
Sidhu, K. ... 128
Siegelman, Noam McMaster ... 178
Silver, Crystal ... 222
Simola, Jaana ... 154
Sinnott, Christian ... 156
Sjödahl, Linnéa ... 172
Skuballa, Irene ... 64
Slattery, Timothy J. ... 113, 187, 242, 244, 245
Slavutskaya, Maria ... 317
Slessor, Gillian ... 171
Slovin, Hamutal ... 349
Smallwood, Jonathan ... 154
Smidekova, Zuzana ... 260, 302
Smith, Daniel T. ... 86, 195
Smith, Heather LE ... 175
Smith, William ... 327
Soltan, M. ... 128
Soltész, Fruzsina ... 253
Sommer, Werner ... 323
Souto, David ... 212
Spichtig, Alexandra ... 40
Spiering, Lisa ... 323
Sponagle, K. ... 101
Spotorno, Sara ... 312
Stacho_, Zden_k ... 269
Stainer, Matthew J. ... 312
Startsev, Mikhail ... 135
Staub, Adrian ... 145
Stefanov, Simeon ... 369
Stefanova, Miroslava ... 369
Steinmair, Karin ... 328
Stewart, Emma E. M. ... 167
Stridh, Martin ... 172
Strnádelová, Bronislava ... 325
Stroganova, Tatyana A. ... 155
Strza_kowski Mateusz ... 216
Sturt, Patrick ... 71, 181
Su, Zi H. ... 341
Sui Xue ... 139, 218
Sun, Hong-Jin ... 306
Svetlana, Alexeeva ... 248
Svirin, Eugeny P. ... 155
Sylvester, Teresa ... 37
Szelepajlo, Michal ... 332
Sz_ll_si, Ágnes ... 81
Tagu, Jérôme ... 121, 224, 297
Tas, Caglar ... 112
Tatler, Benjamin W. ... 69, 171, 312
Taylor, Holly A. ... 382
Taylor, Jack ... 146
Theakston, Anna ... 144
Thiele, D. ... 313
Tikhomandritskaya, O. A. ... 324
Timm, Silja ... 54
Timoshenko, Maria ... 309
Toivanen, Miika ... 191
Toyoura, Makiko ... 334
Trausner, Nina ... 221
Trawinski, T. ... 55
“Truțescu, Gabriela-Olivia” ... 261
Trukenbrod, Hans A. ... 85, 91
Tsai, Jie-Li ... 263, 284
Türkan, Nilay ... 265
Udale, Rob ... 308
Ugwitz, Pavel ... 302
Ushakov, Vadim L. ... 151, 155
Utochkin, Igor S. ... 362
Vakil, Eli ... 114
Valsecchi, Matteo ... 358
van Albada, Sacha J. ... 345
van de Veen, Demy ... 294
van den Hoven, Emiel ... 37
Van der Stigchel, Stefan ... 100
van Ede, Freek ... 46
van Heuven, Walter J. B. ... 146
van Leeuwen, Cees ... 93, 157
van Leeuwen, Jonathan ... 111
Van Merriëboer, Jeroen ... 98
van Opstal, John ... 28, 294
van Renswoude, Daan ... 89, 90
Vansteenkiste, Pieter ... 190
Vasilev, Martin R. ... 113

Vasilyev, Anatoly N. 155, 395
Vaughan, Phillip 69
Velichkovsky, Boris B. 151
Velichkovsky, Boris 196
Velichkovsky, Boris M. 151, 155, 395
Vergilino-Perez, Dorine 224
Visser, Ingmar 89, 90, 261, 314
Vitu, Françoise 137
Voloh, B. 229
Von Suchodoletz, Antje 64
Vorstius, Chris 40, 250
Wagner, Helena 328
Wagner, Ilja 108
Wagner, Peter 354
Walker, Robin 116
Wan, Qianwen 382
Wang, Jue 107
Wang, Jingxin 105, 106, 278
Wang, Mengsi 320
Wang, Ping 174
Wang, Xi 173
Wang, Xiaotong 139
Ward, David 160
Ward, Lindsey 43
Warrington, K.L. 128, 182
Warrington, Kayleigh L. 139, 143
Watson, M.R. 229
Watson, Tamara 208, 374
Weibel, David 204
Weilin, Liu 220
Weiss, Anna Fiona 70
Weiss, Yarden 326
Welch, Leslie 318
Westwood, D.A. 101
Wetzel, Stefanie 213
White, S.J. 128, 182
White, Sarah J. 139, 143, 239, 242
Wickens, C. D. 219
Willems, Roel M. 37
Wimmer, Wilhelm 204
Wisiecka, Katarzyna 51, 256
Wolf, Christian 108, 360
Wollenberg, Luca 88
Womelsdorf, T. 229
Woods, Russell L 115
Wu, C.C. 383
Wynn, Jordana S. 197
Xin Gao 37
Xingshan, Li 220
Xu, Qianqian 105
Yacoub, Abraam 204
Yan, Guoli 185, 186
Yang, Dongsheng 174
Yang, Haibo 122
Yang, Kai-Fu 230, 388
Yates, Mark 113
Yilmaz, Efecan 192
Yima, Esteban 68
Yoneya, Makoto 209
Yongsheng, Wang 185
Yoshida, Takako 291
Youyi Yu 52
Yu, Lili 104
Zaharieva, Martina 261, 314
Zang C. 55
Zang, Chuanli 186, 254
Zechner, Roland 387
Zelinsky, Gregory 124
Zerr, Paul 100
Zhan, Teng-Fei 230
Zhang, Kuo 106, 278
Zhang, Manman 254, 288
Zhang, Wenxin 107
Zhang, Xian-Shi 388
Zhang, Xue 106
Zhang, Yingliang 107
Zhang, Zhichao 254
Zhao, Darisy G. 155, 395
Zhao, Sainan 105
Zhou, Junyi 103
Zhu, Meng 185
Zhukova, Alisa 251
Zimmermann, Daniel 217
Zimmermann, Eckart 84
Zlokazova T. 268
Zoï, Kapoula 330
Zovetti, Niccolò 331
Zubov, Vladislav 240

20th ECEM 2019
European Conference on Eye Movements
18-22 August 2019, Alicante, Spain

www.ecem2019.com
